# Post‐Modification of Tripyrenylenes to Enantiopure π‐Extended *D*_3_‐Symmetric Propellers

**DOI:** 10.1002/chem.71239

**Published:** 2026-06-13

**Authors:** Dennis Popp, Melanie Bruttel, Sebastian Möbert, Anusha Sounder Rajan, Christoph Keck, Darius Boehm, Frank Rominger, Sven M. Elbert, Michael Mastalerz

**Affiliations:** ^1^ Organisch‐Chemisches Institut Ruprecht‐Karls‐Universität Heidelberg Heidelberg Germany

**Keywords:** chirality, *N*‐heteropolycycles, optical properties, polycyclic aromatic hydrocarbons, pyrene

## Abstract

A palladium‐catalyzed trimerization of a pyrene‐based Kobayashi aryne precursor and post‐functionalization of the obtained tripyrenylene was used to synthesize polycyclic aromatic hydrocarbons with multiple helicene subunits. The isomerization of the obtained *C*_2_‐isomers to the corresponding propeller‐shaped *D*_3_‐isomers was investigated and the enantiomers of a *D*_3_‐pyrenylene as well as a *D*_3_‐trisphenanthrophenazine were isolated. Their chiroptical properties as well as the crystalline packings in comparison to the corresponding racemates were studied and the isomerization as well as racemization processes were investigated kinetically and by quantum chemical calculations.

## Graphical abstract

The post‐modification of tripyrenylenes to π‐extended enantiopure propellers with tris‐phenanthrophenazines is presented and the chiroptical properties as well as the solid‐state packings investigated in comparison to the corresponding racemic mixtures.

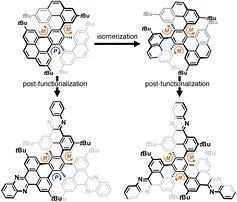

## 1 Introduction

**FIGURE 1:**
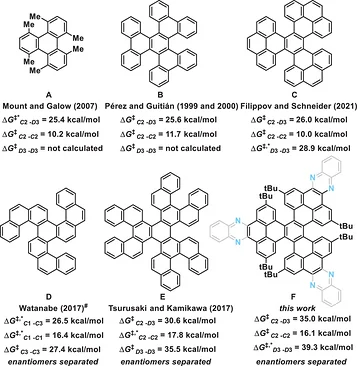
Structures of literature known triphenylene‐based PAHs **A–E** with isomerization barriers. The values marked with an asterisk have been calculated by methods described in the corresponding papers. # *Note*: Compound **D** was first described by Pérez and Guitián [25] and later investigated by Watanabe [38].

Since the first synthesis of [4]‐ and [5]‐helicenes by Weitzenböck, the scientific interest in helicenes remains high and even experienced a renaissance in the last decades [1, 2, 3, 4, 5, 6]. Enantiopure and configurationally stable helicenes show very good optical and electronic properties, such as large absolute absorption dissymmetry factors *g*_abs_ [3, 7, 8, 9]. In recent years, polycyclic aromatic hydrocarbons (PAHs) with multiple helicene substructures have been synthesized [10, 11, 12, 13] with triple helicenes as a prominent subgroup of such [10]. While several different methods toward such compounds have been developed [14, 15, 16, 17], the palladium‐catalyzed trimerization of arynes [18, 19, 20, 21, 22] derived from Kobayashi aryne precursors [23] proved to be a versatile tool and structures such as a bay‐methylated triphenylene (**A** in Figure 1) [24], two regioisomers of trinaphthylene [25], regioisomers of triphenanthrylene (e.g. **B** and **D** in Figure 1) [25, 26], substituted dibenzopicene [27], trimers of bi‐ and triphenylene [28, 29], tricorannulene [30], unsubstituted and *tert*‐butylated tripyrenylenes (**C** and **F** in Figure 1) [31, 32], an overcrowded triply fused carbo[7]helicene [33] and even hexapole [5]helicenes (**E** in Figure 1) [34] were realized by this method.

Empirically, it became obvious that in contrast to some other reactions toward triple helicenes [15, 35], the palladium‐catalyzed trimerization of arynes gives *C*_2_‐symmetrical products exclusively, when the steric demand of the formed helicenes hindered fast interconversions [30, 31, 32, 33, 34]. This phenomenon was later elucidated by quantum chemical calculations of the underlying mechanism and the geometry of the insertion of a third aryne into a five‐membered palladacycle intermediate by a kinetically controlled reaction was the driving force favoring the *C*_2_‐symmetrical products [31, 32, 36, 37]. As exception, in the case of an overcrowded triply‐fused carbo[7]helicene, the *C*_2_‐symmetric conformation was locked because this isomer was thermodynamically more stable than the *D*_3_‐ or *C*_3_‐isomer, excluding isomerization studies [33].

The interconversion to the corresponding higher symmetric and thermodynamically more stable propeller‐shaped *D*_3_‐ or *C*_3_‐isomers was realized for several PAHs by thermal treatment (Figure 1) [32, 34, 39]. In all cases, the *C*_2_‐*C*_2_ isomerization barriers were between 10.0 and 17.8 kcal mol^−1^, preventing the isolation of the *C*_2_‐symmetric enantiomeric pairs. For the bay substituted hexamethyltriphenylene **A** [24], triphenanthrylene regioisomer **B** [25, 39], and tripyrenylene **C** [32] isomerization barriers of 25.4–26.0 kcal mol^−1^ for the *C*_2_‐*D*_3_ conversion were found. However, the separation of the corresponding *D*_3_‐symmetric enantiomers was not reported. To the best of our knowledge, only for triphenanthrylene **D** reported first by Pérez and Guitián [25] and later investigated by Watanabe [38] and the hexapole [5]helicenes **E** [34] described by Tsurusaki and Kamikawa the propellers of threefold symmetry were separated by chiral HPLC and studied in their enantiopure forms [34].

Herein, propeller‐shaped PAHs based on the *C*_2_‐symmetric *tert*‐butylated pyrenylene reported before [31] were investigated for their *C*_2_‐*D*_3_ conversions. Chiral *D*_3_‐symmetric propellers were isolated and their chiroptical properties investigated. By post‐functionalization through oxidation, the chiral propeller π‐systems were enlarged by condensation reactions and studied as well.

## 2 Results and Discussion

### 2.1 Synthesis

**SCHEME 1:**
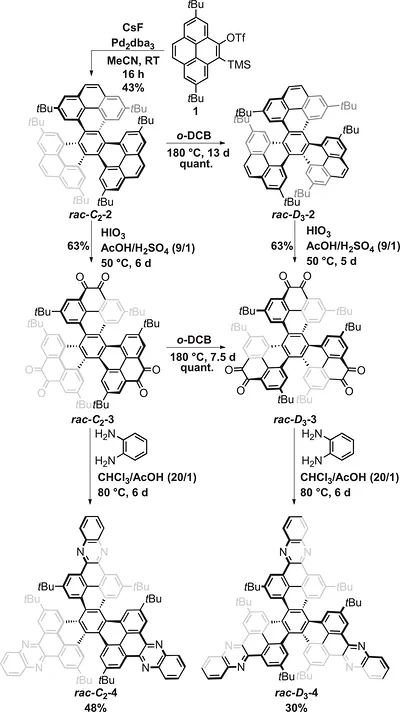
Pd‐catalyzed trimerization of **1** to *C*_2_‐symmetric tripyrenylene rac‐*C*_2_‐**2** and subsequent post‐functionalization. For simplicity only one enantiomer of racemic compounds is depicted.

The *tert*‐butylated pyrene aryne precursor **1** was generated in 43% yield over four steps from readily available precursors [40] as recently reported [31]. By increasing the catalyst loading of Pd_2_dba_3_ from 5 to 25 mol% in the cyclisation reaction, the formation of higher oligomers was suppressed and the yield of *C*_2_‐symmetrical tripyrenylene ***rac*‐*C*_2_‐2** was improved from 9% [31] to 43% (Scheme 1). Heating ***rac*‐*C*_2_‐2** for 13 days at 180 °C and removing the solvent under reduced pressure gave ***rac*‐*D*_3_‐2** in quantitative yield. Both, ***rac*‐*C*_2_‐2** and ***rac*‐*D*_3_‐2** were further oxidized at the pyrene *K*‐regions (HIO_3_, glacial AcOH, H_2_SO_4_ (0.18 M), 50 °C) [41], giving the corresponding *C*_2_‐symmetrictripyrenylene hexaone ***rac*‐*C*_2_‐3** and *D*_3_‐symmetric tripyrenylene hexaone ***rac*‐*D*_3_‐3** in yields of 88% and 87%. Hexaone ***rac*‐*D*_3_‐3** was also accessible in quantitative yield by isomerization of ***rac*‐*C*_2_‐3** under similar conditions as described above (*o*‐DCB, 180 °C, 7.5 days). Hexaones ***rac*‐*C*_2_‐3** and ***rac*‐*D*_3_‐3** were subsequently condensed with *o*‐phenylenediamine (CHCl_3_, AcOH 20/1 (v:v), 80 °C) [42] to give the *C*_2_‐ and *D*_3_‐symmetric tripyrenylene triphenazines ***rac*‐*C*_2_‐4** and ***rac*‐*D*_3_‐4** in 48% and 81% yield, respectively.

**FIGURE 2:**
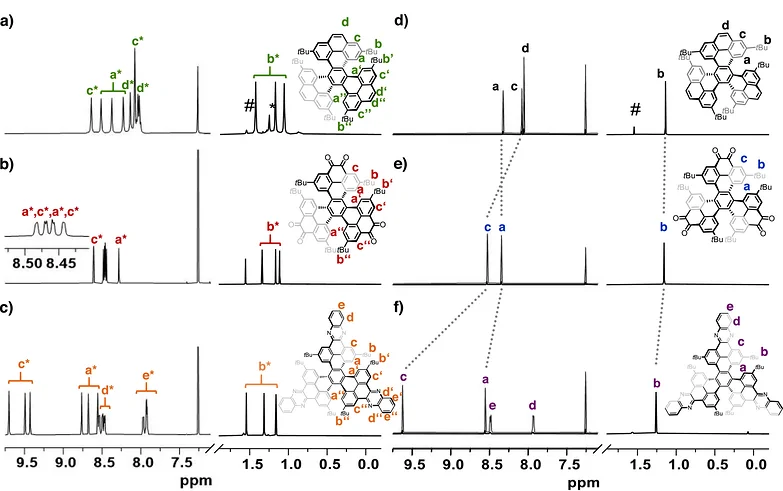
^1^H NMR spectra of (a) tripyrenylene ***rac*‐*C*_2_‐2** (CDCl_3_, 500 MHz) [31], (b) hexaone ***rac*‐*C*_2_‐3** (CDCl_3_, 700 MHz), (c) trisphenanthrophenazine ***rac*‐*C*_2_‐4** (CDCl_3_, 700 MHz), (d) tripyrenylene ***rac*‐*D*_3_‐2** (CDCl_3_, 700 MHz), (e) hexaone ***rac*‐*D*_3_‐3** (CDCl_3_, 600 MHz), (f) trisphenanthrophenazine ***rac*‐*D*_3_‐4** (CDCl_3_, 700 MHz). The aromatic regions are shown in threefold intensity. Due to the molecular symmetry of ***rac*‐*C*_2_‐2‐4** it is not possible to distinguish the protons between the different aromatic “wings” by 2D NMR experiments. Therefore, protons at positions a, a’ and a″ cannot be assigned and are marked with asterisks (e. g. a*).

All compounds have been fully characterized by common analytical methods (see Supporting Information). The different conformations can be clearly distinguished by ^1^H NMR spectroscopy as for example only three signals of aromatic protons are found in the ^1^H NMR spectrum of ***rac*‐*D*_3_‐2** at *δ*  =  8.47, 8.12, and 8.00 ppm, while ***rac*‐*C*_2_‐2** shows nine signals for aromatic protons instead, due to its lower symmetry (see Figure 2a,d).

**FIGURE 3:**
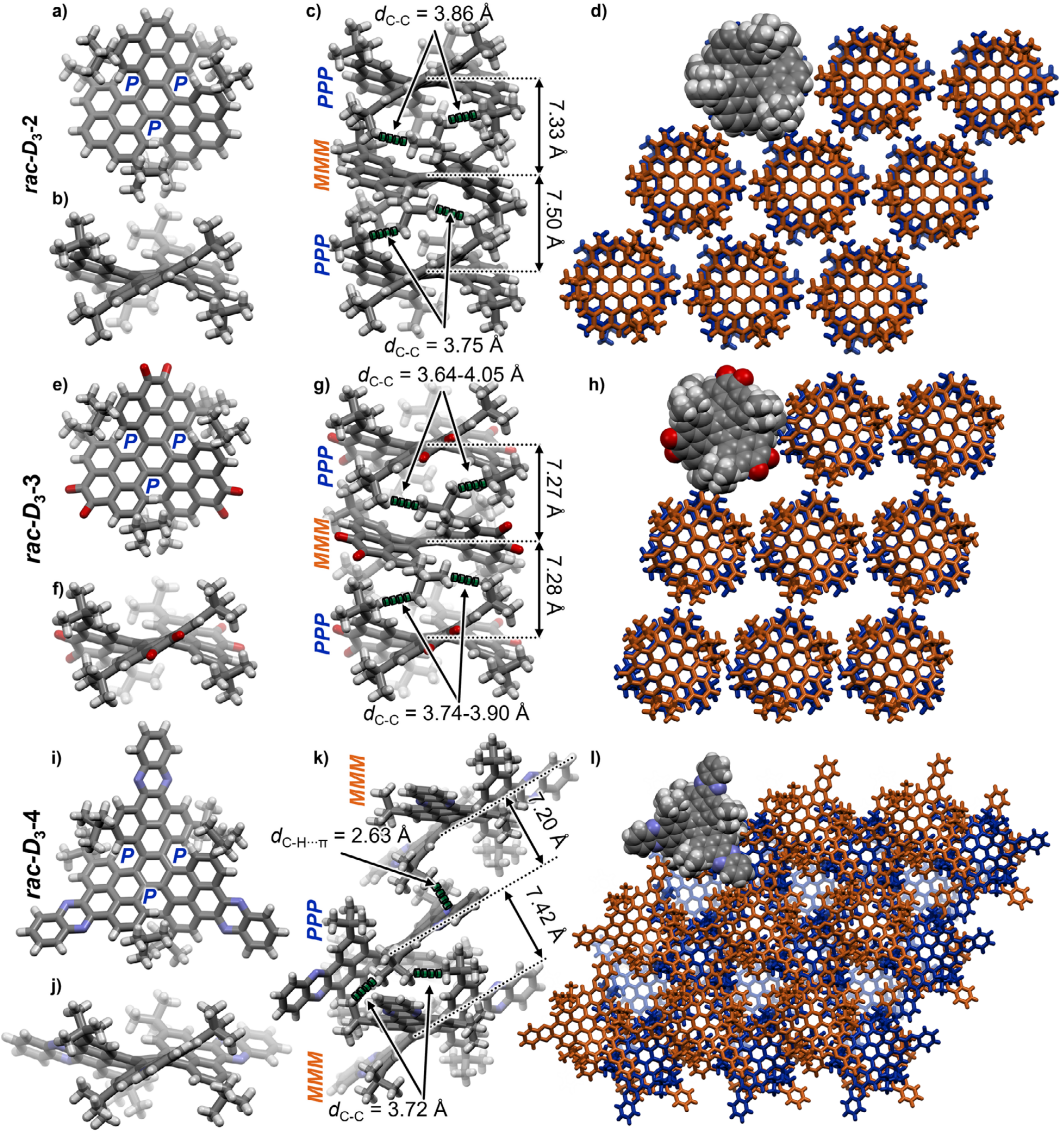
Single crystal Xkcal mol−1‐ray structures of **rac‐*D*_3_‐2** (a–d), **rac‐*D*_3_‐3** (e–h) and **rac‐*D*_3_‐4** (i–l) as capped sticks models. In the first column only one enantiomer each is shown from top view (top) and side view (bottom). Column three shows racemic column formation. Column four represents a top view on the packing motifs with the *M*,*M*,*M*‐isomers in orange and the P,*P*,*P*‐isomers in blue. In the packing representations, one molecule each is shown as space filling model in element colors. Grey: carbon; white: hydrogen; red: oxygen; blue: nitrogen.

Crystals suitable for single crystal X‐ray diffraction analysis (SCXRD) were obtained for pyrenylene ***rac*‐*D*_3_‐2**, hexaones ***rac*‐*C*_2_‐3** and ***rac*‐*D*_3_‐3** as well as trisphenanthrophenazine ***rac*‐*D*_3_‐4**, confirming their conformations (Figure 3).

Pyrenylene ***rac‐D*_3_‐2** crystallizes in the trigonal space group $P\bar{3}$ with *Z*  =  2 by slow evaporation of a CDCl_3_‐solution. The propeller‐like shape of the molecules is caused by three [5]‐helicene units of identical chirality where the pyrene moieties are equally bent out of the mean plane of the central benzene ring by ∡  =  27° (Figures 3a and b). Racemic columns (Figure 3c) are formed by interactions with enclathrated, disordered chloroform as well as by intermolecular dispersion interaction [43] of the *tert*‐butyl groups (*d*_C‐C _ =  3.86 Å and *d*_C‐C _ =  3.75 Å measured as the shortest distance between two carbon atoms of the *tert*‐butyl groups), which is comparable to dispersion interactions between *tert*‐butyl groups for instance in crystals of structurally similar quinoxalinophenanthrophenazines (*d*_C‐C _ =  3.62‐4.06 Å) [44]. The latter also mainly contributes to the formation of enantiopure sheets in the crystallographic *ab*‐plane that alternate along the crystallographic *c*‐axis with a distance of 7.33 Å and 7.50 Å (measured as the distance of mean planes of the central benzene units).

Hexaone ***rac‐D*_3_‐3** crystallizes in the triclinic space group *P* $\bar{1}$ with *Z*  =  2 and shows a comparable contortion (∡  =  27°, Figures 3e and f), non‐planarity value (0.13 Å) as well as packing as pyrenylene ***rac‐D*_3_‐2**. It also forms alternating enantiopure planes along the crystallographic *a*‐axis with a distance of 7.27 Å and 7.28 Å (Figure 3g). Similar to ***rac‐D*_3_‐2**, the racemic columns of ***rac‐D*_3_‐3** (Figure 3g,h) are formed by interactions with enclathrated, disordered chloroform molecules as well as dispersion interactions of the *tert*‐butyl groups with each other, which are with *d*_C‐C _ =  3.74‐3.90 Å and *d*_C‐C _ =  3.64‐4.05 Å in the same range as for ***rac‐D*_3_‐2** (*d*_C‐C _ =  3.89 Å). Crystals of trisphenanthrophenazine ***rac‐D*_3_‐4** were obtained by slow evaporation of solvent from a concentrated chloroform solution of it. It crystallizes in the trigonal space group *P* $\bar{3}$ with *Z*  =  6. With a non‐planarity value of 0.10 Å and a contortion out of the central mean plane by ∡  =  28° (Figure 3i,j), the key conformational features match those of its precursors ***rac‐D*_3_‐2** and ***rac‐D*_3_‐3**. In contrast to ***rac‐D*_3_‐2** and ***rac‐D*_3_‐3,** racemic dimers are formed instead of columns by cdispersion interactions of the *tert*‐butyl groups (*d*_C‐C _ =  3.72 Å). Enantiopure sheets are found in the crystallographic *ab*‐plane and two sheets stack along the crystallographic *c*‐axis, connected by C─H∙∙∙π‐interactions (*d*_C‐H∙∙∙π_  =  2.63 Å) between *tert*‐butyl groups and the other part of the π‐systems of adjacent molecules (Figure 3k). Along the crystallographic *c*‐axis, the stacked molecules of each double sheet are shifted to create an ABC‐like pattern (Figure 3l).

**FIGURE 4:**
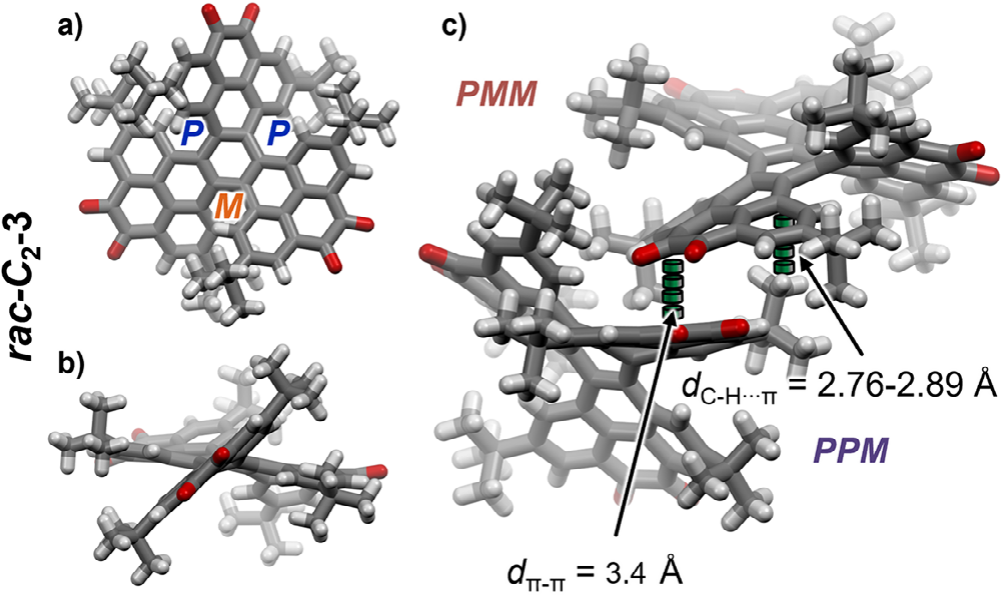
Single crystal X‐ray structure of **rac‐*C*_2_‐3** In the first column only one enantiomer each is shown from top view (a) and side view (b). c) shows racemic dimer formation. Grey: carbon; white: hydrogen; red: oxygen; blue: nitrogen.

Crystals of hexaone ***rac*‐*C*_2_‐3** were obtained by layer‐to‐layer diffusion of methanol into a concentrated DCM solution. ***rac*‐*C*_2_‐3** crystallizes in the triclinic space group *P* $\bar{1}$ with *Z*  =  4 (Figure 4). The pyrene unit along the molecular *C*_2_‐axis is almost planar with a non‐planarity value [45, 46] (defined by the average distance of the 16 carbon atoms of the corresponding pyrene subunits from their mean‐plane) of 0.12 Å and is bent out‐of‐plane by 41° in respect to the central benzene unit (Figure 4a,b). The remaining two pyrene units are less planar (non‐planarity value of 0.27 Å) and less contorted relative to the central benzene with 12°. The packing of ***rac*‐*C*_2_‐3** is mainly driven by π‐stacking (*d*_π‐π_  =  3.4 Å; measured as shortest distance between two carbon atoms of carbonyl groups of adjacent molecules) as well as CH∙∙∙π‐interactions (*d*_C‐H∙∙∙π_  =  2.76‐2.89 Å) of the *tert*‐butyl‐groups with the extended aromatic systems of adjacent molecules, leading to a distorted layer‐like packing of enantiopure sheets. In contrast to that, the packing of the isomeric hexaone ***rac*‐*D*_3_‐3** almost resembles the one of pyrenylene ***rac‐D*_3_‐2**.

### 2.2 Optical Properties and Quantum Chemical Calculations

**FIGURE 5:**
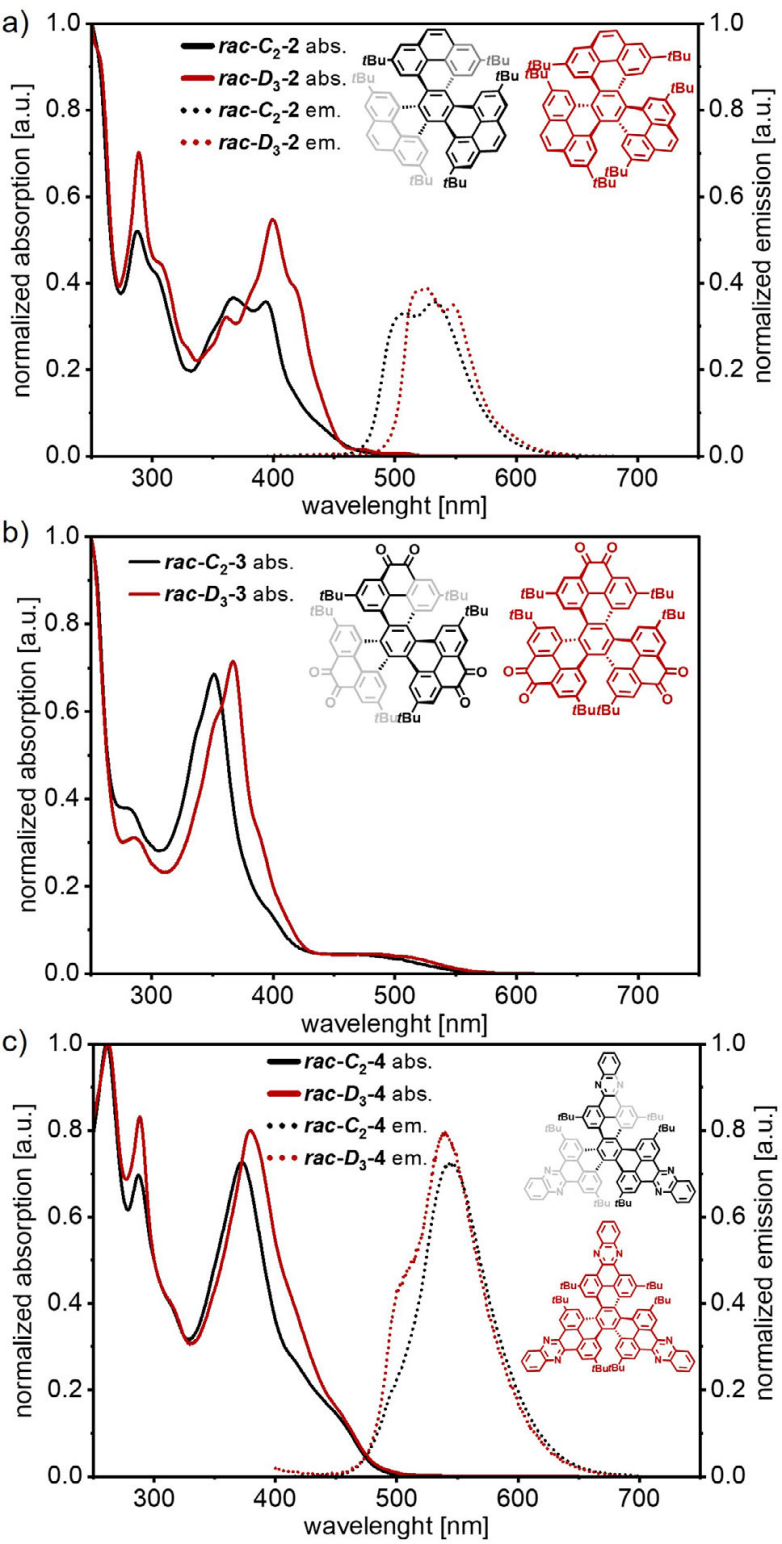
UV/vis absorption (solid lines) and emission (dotted lines) spectra of (a) ***rac*‐*C*_2_‐2** (black) [31] and ***rac*‐*D*_3_‐2** (red) (b) ***rac*‐*C*_2_‐3** (black) and ***rac*‐*D*_3_‐3** (red) and (c) ***rac*‐*C*_2_‐4** (black) and ***rac*‐*D*_3_‐4** (red) in dichloromethane (***rac*‐*C*_2_‐2**, ***rac*‐*D*_3_‐2**) or chloroform (***rac*‐*C*_2_‐3, *rac*‐*D*_3_‐3**, ***rac*‐*C*_2_‐4** and ***rac*‐*D*_3_‐4**) at room temperature.

The optical properties of all compounds were investigated by UV/vis absorption and fluorescence spectroscopy in dichloromethane or chloroform at room temperature and compared with the previously reported ***rac*‐*C*_2_‐2** [31]. Corresponding bands were assigned using TD‐DFT calculated spectra (calculated at the B3LYP/6‐311G(d,p) level of theory). ***rac*‐*C*_2_‐2** has a maximum at *λ*  =  394 nm, which is attributed to the HOMO‐2 to LUMO transition according to TD‐DFT calculations (see Supporting Information). Two shoulders at *λ*  =  420 nm and *λ*  =  440 nm are assigned to the HOMO‐LUMO transition with an oscillator strength of *f* = 0.1245. In comparison, ***rac*‐*D*_3_‐2** has a pronounced maximum at *λ*  =  400 nm and two shoulders at *λ*  =  420 nm and *λ*  =  440 nm, which correspond to transitions with lower oscillator strengths (Figure 5a and Supporting Information). The corresponding weak emission (*Φ*  =  2%–4%) is bathochromically shifted by Δ*λ * =  16 nm from *λ*_em_  =  509 and 533 nm (***rac*‐*C*_2_‐2**) to *λ*_em_  =  525 and 549 nm in ***rac*‐*D*_3_‐2**.

Hexaone ***rac*‐*C*_2_‐3** only shows one distinct band at *λ*_abs_  =  351 nm. Again, for the higher symmetric isomer ***rac*‐*D*_3_‐3** a bathochromic shift of Δ*λ*  =  16 nm to *λ*_abs_  =  367 nm is observed. Additionally, both isomers show broad bands between *λ * =  450‐550 nm that correspond to the HOMO‐LUMO transitions and have comparably low oscillator strengths of *f* = 0.0213 (***rac*‐*C*_2_‐3**) and 0.0379 (***rac*‐*D*_3_‐3**). In contrast to the parent pyrenylenes, ***rac*‐*C*_2_‐3** and ***rac*‐*D*_3_‐3** are non‐fluorescent at room temperature.

Trisphenanthrophenazines ***rac*‐*C*_2_‐4** and ***rac*‐*D*_3_‐4** (*λ*_abs_  =  379 nm) show bands of higher intensity at *λ*_abs_  =  373 nm (***rac*‐*C*_2_‐4**) and *λ*_abs_  =  379 nm (***rac*‐*D*_3_‐4**), which are slightly bathochromically shifted in comparison to analogous peaks of hexaones ***rac*‐*C*_2_‐3** and ***rac*‐*D*_3_‐3**. Here also shoulders at *λ* ∼ 420 nm and *λ* ∼ 450 nm are detected for both isomers attributed to HOMO‐LUMO‐transitions according to TD‐DFT (see Supporting Information). Mirror image emissions can be found at *λ*_em_  =  545 nm (***rac*‐*C*_2_‐4**) and *λ*_em_  =  541 nm (***rac*‐*D*_3_‐4**) and as for the pyrenylenes, the *C*_2_‐isomer ***rac*‐*C*_2_‐4** shows a higher PLQY of 12% as ***rac*‐*D*_3_‐4** (3%). The overall spectroscopic features differ noticeably from unsubstituted phenanthrophenazine for which the absorption (*λ*_abs_  =  435 nm) as well as emission (*λ*_em_  =  471 nm) are hypsochromically shifted [47] suggesting an electronic communication of the phenanthrophenazine units over the central six‐membered ring in ***rac*‐*C*_2_‐4** and ***rac*‐*D*_3_‐4**. Furthermore, the PLQY of unsubstituted phenanthrophenazine is about ten times higher (*Φ*  =  35%) indicating additional degrees of freedom of the non‐planar and twisted ***rac*‐*C*_2_‐4** and ***rac*‐*D*_3_‐4** in comparison to simple phenanthrophenazines [47].

In contrast to the observed spectroscopic differences of the *C*_2_‐ and the *D*_3_‐isomers, quantum chemical calculations (B3LYP/6‐311G(d,p)) [48, 49, 50, 51, 52] only show minor differences of orbital energy levels and the distribution of orbital coefficients based on the different symmetries (Table 1 and Supporting Information).

**TABLE 1:** Experimental and quantum‐mechanically calculated optical properties of all discussed compounds.

Compound	*λ*_abs_ (nm)	*λ*_em_ (nm)	PLQY	$\tilde{\nu }$ (cm^−1^)^a^	*λ*_onset_ (nm)	$E_{g}^{\mathrm{opt}}$ (eV)^b^	$E_{\mathrm{HOMO}}^{\mathrm{DFT}}$ (eV)^c^	$E_{\mathrm{LUMO}}^{\mathrm{DFT}}$ (eV)^c^	$E_{g}^{\mathrm{DFT}}$ (eV)^c^
***rac*‐*C*_2_‐2** ^d^ [31]	287, 303, 367, 394	509, 533	0.04	5747	462	2.7	−5.2	−2.0	3.2
***rac‐D*_3_‐2** ^d^	289, 361, 400, 414	525, 549	0.02	5906	487	2.6	−5.2	−2.0	3.3
***rac*‐*C*_2_‐3** ^e^	351, 473	—	—	—	587	2.1	−6.2	−3.4	2.9
***rac‐D*_3_‐3** ^e^	286, 367, 480	—	‐–	—	591	2.1	−6.2	−3.3	2.9
***rac*‐*C*_2_‐4** ^e^	261, 288, 373	545	0.12	8461	502	2.5	−5.7	−2.4	3.3
***rac‐D*_3_‐4** ^e^	262, 289, 379	541	0.03	7901	507	2.5	−5.7	−2.4	3.3

^a^Calculated from the most bathochromic absorption maximum and the most hypsochromic emission maximum.

^b^Calculated by the measured absorption onset in the UV/vis spectrum.

^c^Calculated at the B3LYP/6‐311G(d,p) [48, 49, 50, 51, 52] level of theory.

^d^Measured in dichloromethane at room temperature.

^e^Measured in chloroform at room temperature.

**FIGURE 6:**
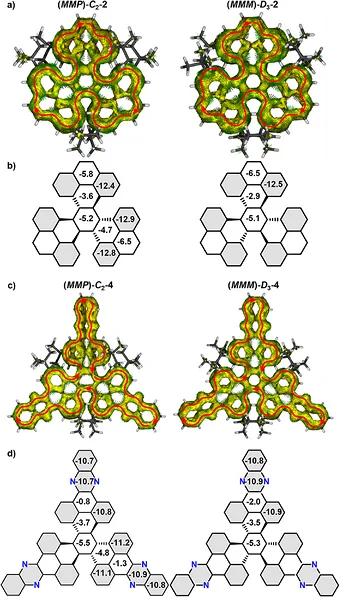
ACID‐plots (a and c) and NICS(1)_av_‐values (b and d) of the pyrenylenes **(*M,M,P*)‐*C*_2_‐2** and **(*M,M,M*)‐*D*_3_‐2** (a and b) as well as the trisphenanthrophenazines **(*M,M,P*)‐*C*_2_‐4** and **(*M,M,M*)‐*D*_3_‐4** (c and d) calculated at the HF/631+G(d) level of theory.

ACID‐plots as well as NICS(1)_av_‐values were calculated (HF/6‐31+G(d)) [53, 54, 55, 56, 57] (Figure 6) [58, 59, 60, 61, 62], revealing only minor differences between the *C*_2_‐ and the *D*_3_‐isomers of pyrenylenes ***rac*‐*C*_2_‐2** and ***rac*‐*D*_3_‐2** as well as trisphenanthrophenazines ***rac*‐*C*_2_‐4** and ***rac*‐*D*_3_‐4**.

The central benzene rings have similar NICS values (*δ*  = ‐5.1 ppm to *δ*  = ‐5.5 ppm) as in triphenylene (*δ*  = ‐5.1 ppm) but differ from larger starphenes (*δ*  = +0.4 ppm) [63]. The values obtained for the pyrene substructures of the trisphenanthrophenazines match the aromatic properties calculated for unsubstituted pyrene with the K‐region as only exception [64]. The calculated ACID‐plots show a global ring current [65] which explains the bathochromic shifts observed for trimers in comparison to monomeric units.

### 2.3 Isomerization Studies

**FIGURE 7:**
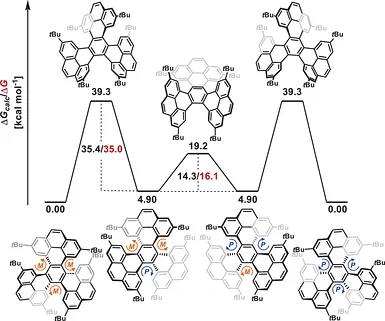
Energy diagram for the racemization of pyrenylene **rac‐*C*_2_‐2** and the isomerization to **rac‐*D*_3_‐2** (B3LYP/6‐311G(d,p)) [52] (black values) and determined experimentally by kinetic ^1^H NMR studies in o‐DCB‐d_4_ (red values, for details see Supporting Information).

As discussed above, ^1^H NMR spectra of pyrenylene **rac‐*C* *_2_*‐2** at room temperature showed more complex patterns than those of **rac‐*D*_3_‐2** (Figure 2a). When heated up to 140 °C in *ortho*‐dichlorobenzene‐d_4_ only three signals are detected (see Supporting Information), which is expected due to the threefold symmetry. For example, the signals at *δ*  =  8.72 and 8.53 ppm merge at 50 °C to one signal at *δ*  =  8.67 ppm, which clearly distinguishes from **rac‐*D*_3_‐2**, where this peak is resonating at *δ*  =  8.47 ppm. Cooling to room temperature gave the initial spectra of *C*_2_‐symmetric **rac‐*C*_2_‐2** (see Supporting Information), revealing that the spectroscopic observations correspond to a *C*_2_‐to‐*C*_2_ racemization. The experimental inversion barrier of 16.1 kcal mol^−1^ (derived from kinetic studies at variable temperatures) is close to the theoretical inversion barrier of $\Delta G_{C_{2}-C_{2}}$=  14.3 kcal mol^−1^ (B3LYP/6‐311G(d,p) with GD3BJ dispersion correction [66] and the SMD solvent model using o‐DCB as solvent) [67] (Figure 7). These energies correspond to inversion half‐lives in the millisecond regime at room temperature, which makes a separation of the *C*_2_‐symmetric enantiomers not possible [68], similar to the hexapole [5]helicenes [34].

**FIGURE 8:**
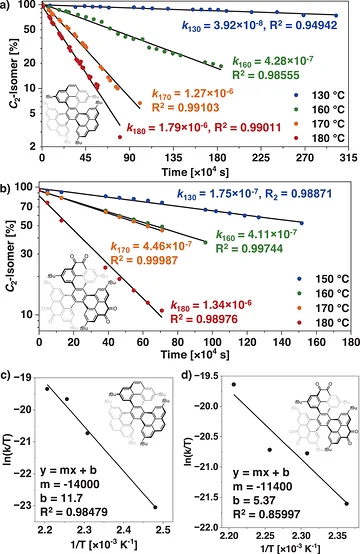
(a,b) Logarithmic plots of the amount of *C*_2_‐isomer of **rac‐*C*_2_‐2** (a) and **rac‐*C*_2_‐3** (b) to determine rate constants by the slope of the respective linear fits. c,d) Eyring‐plots of the symmetry change of **rac‐*C*_2_‐2/rac‐*D*_3_‐2** (c) and **rac‐*C*_2_‐3/rac‐*D*_3_‐3** (d).

The transition state for a *C*_2_‐to‐*D*_3_ isomerization is with $\Delta G_{C_{2}-D_{3}}^{‡}$  =  39.3 kcal mol^−1^ much higher in energy. Please note, that by the additional *tert*‐butyl groups, these values are increased by ∼ 10 kcal mol‐1 in comparison to the parent compound without any substituents [32]. The theoretical activation barrier of 35.4 kcal mol^−1^ is in good agreement to the experimental one of 35.0 kcal mol^−1^_,_ obtained by kinetic ^1^H NMR studies at even higher temperatures of 130 to 180 °C (for details see Figure 8). This explains why the *C*_2_‐to‐*D*_3_‐conversion takes longer reaction times of up to 13 days even at 180 °C. The *D*_3_‐symmetric pyrenylene is thermodynamically more stable than the *C*_2_‐isomer by 4.90 kcal mol^−1^, which is comparable to other trimeric compounds with three [5]helicene units [32, 37, 38, 39]. Since a potential racemization of the *D*_3_‐isomers proceeds through the *C*_2_‐isomers as intermediates (Figure 7), the half‐life is about 1000 years at room temperature, which enables the separation of the corresponding enantiomers (see discussion below). Similar experimental investigations and calculations were done for **rac‐*C*_2_‐3** and **rac‐*D*_3_‐3** and comparable barriers of $\Delta G_{C_{2}-C_{2},\mathrm{ex}}$=  19.5 kcal mol^−1^ ($\Delta G_{C_{2}-C_{2},\mathrm{theo}}$=  10.4 kcal mol^−1^) and $\Delta G_{C_{2}-D_{3},\mathrm{ex}}$=  33.4 kcal mol^−1^ ($\Delta G_{C_{2}-D_{3},\mathrm{ex}}$=  35.0 kcal mol^−1^) have been obtained (for details see Supporting Information). This demonstrates that the peripheral modification of the trimeric pyrenylene scaffold is barely influencing the isomerization behavior.

### 2.4 Chiral Resolution and Chiroptical Properties

**FIGURE 9:**
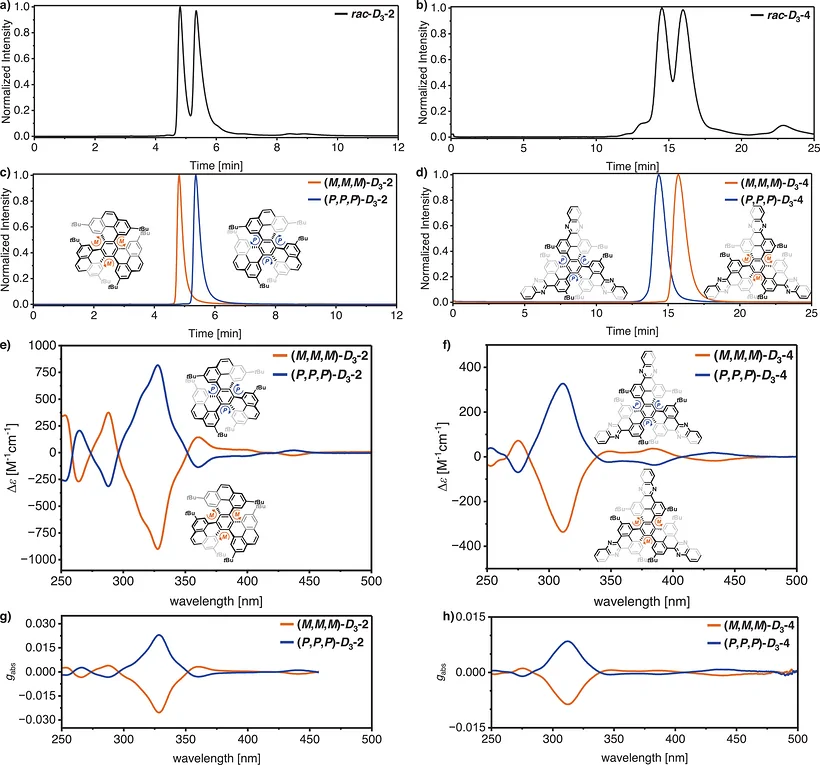
(a) and (b) HPLC‐chromatograms of ***rac*‐*D*_3_‐2** (left) and ***rac*‐*D*_3_‐4** (right) on CHIRALPAK‐IA columns (left: 4.6×250 mm, *n*‐heptane, 1 mL/min; right: 20×300 mm, *n*‐heptane/*iso*‐propanol 99.5:0.5, 6 mL/min). (c) and (d) Overlaid HPLC‐chromatograms of the isolated enantiomers. (e) and (f) CD spectra of ***D*_3_‐2** (left) and ***D*_3_‐4** (right) in dichloromethane at room temperature. (g) and (h) Absorption dissymmetry factors of the corresponding enantiomers of ***rac*‐*D*_3_‐2** (left) and ***rac*‐*D*_3_‐4** (right) in dichloromethane at room temperature.

The separation of the enantiomers of ***rac*‐*D*_3_‐2** as well as ***rac*‐*D*_3_‐4** was realized by chiral HPLC using a CHIRALPAK‐IA column and pure *n*‐heptane (***rac*‐*D*_3_‐2**) or *n*‐heptane with 0.5 vol.‐% isopropanol as eluents and the single enantiomers were isolated in >99% enantiopurity as determined by integration of the respective peaks in the chromatograms (Figure 9a–d). It has to be noted that attempts to separate the enantiomers of hexaone ***rac*‐*D*_3_‐3** failed due to stability issues. The enantiomers of ***D*_3_‐2** show complementary mirror‐image circular dichroism (CD) spectra with a weak Cotton effect of **(*M*,*M*,*M)*‐*D*_3_‐2** in the low energy regime (*λ*  =  438 nm) and vice versa. The absolute configuration was assigned by comparison of experimental and theoretical (TD‐DFT calculated) CD spectra (for details see Supporting Information).

Experimental dissymmetry factors (*g*_abs,_) up to |*g*_abs_|  =  1.1∙10^−3^ for the local maximum at *λ*  =  438 nm and |*g*_abs, max_|  =  2.4∙10^−2^  for the global maximum at *λ*  =  329 nm were found in dichloromethane, which are in the same order of magnitude as dissymmetry factors of other multiple helicenes [8, 69].

Quantum mechanical calculations suggest, that the local minimum constitutes the S_0_→S_2_ transition, comprised of 71% HOMO‐LUMO transition, the S_0_→S_1_ transition is dark (*f*  =  0). In contrast, the global maximum at *λ*  =  329 nm corresponds to the S_0_→S_15_ transition, in which the electric and magnetic transition dipole moments have an angle of *θ*  =  169° (see Supporting Information), which is beneficial for the high experimental *g*_abs_‐value (|*g*_theoretical_| = 4·cos(*θ*)·|µ_m_|/|*µ*_e_|) [8, 10]. The enantiomers of ***D*_3_‐4** show a comparable behavior in CD spectroscopy (Figure 9f and h). Again, a weak negative Cotton effect is found for the low energy regime (*λ*  =  432 nm) of (*M,M,M*)‐***D*_3_‐4** with dissymmetry factors of |*g*_abs_|  =  8∙10^−4^. The dissymmetry factor at the global maximum of phenazine ***D*_3_‐4** (*λ*  =  313 nm) is |*g*_abs_|  =  8.4∙10^−3^, which is lower in comparison to the unsubstituted ***D*_3_‐2**. Here the S_0_→S_39_ transition is the main contributor and shows an angle of 180° between the magnetic and electronic dipole moments (see Supporting Information).

***D*_3_‐2** and ***D*_3_‐4** show no circularly polarized luminescence (CPL) signals in dichloromethane at room temperature, which is attributed to the low PLQYs of 2–4% of ***D*_3_‐2** and ***D*_3_‐4** (Table 1).

**FIGURE 10:**
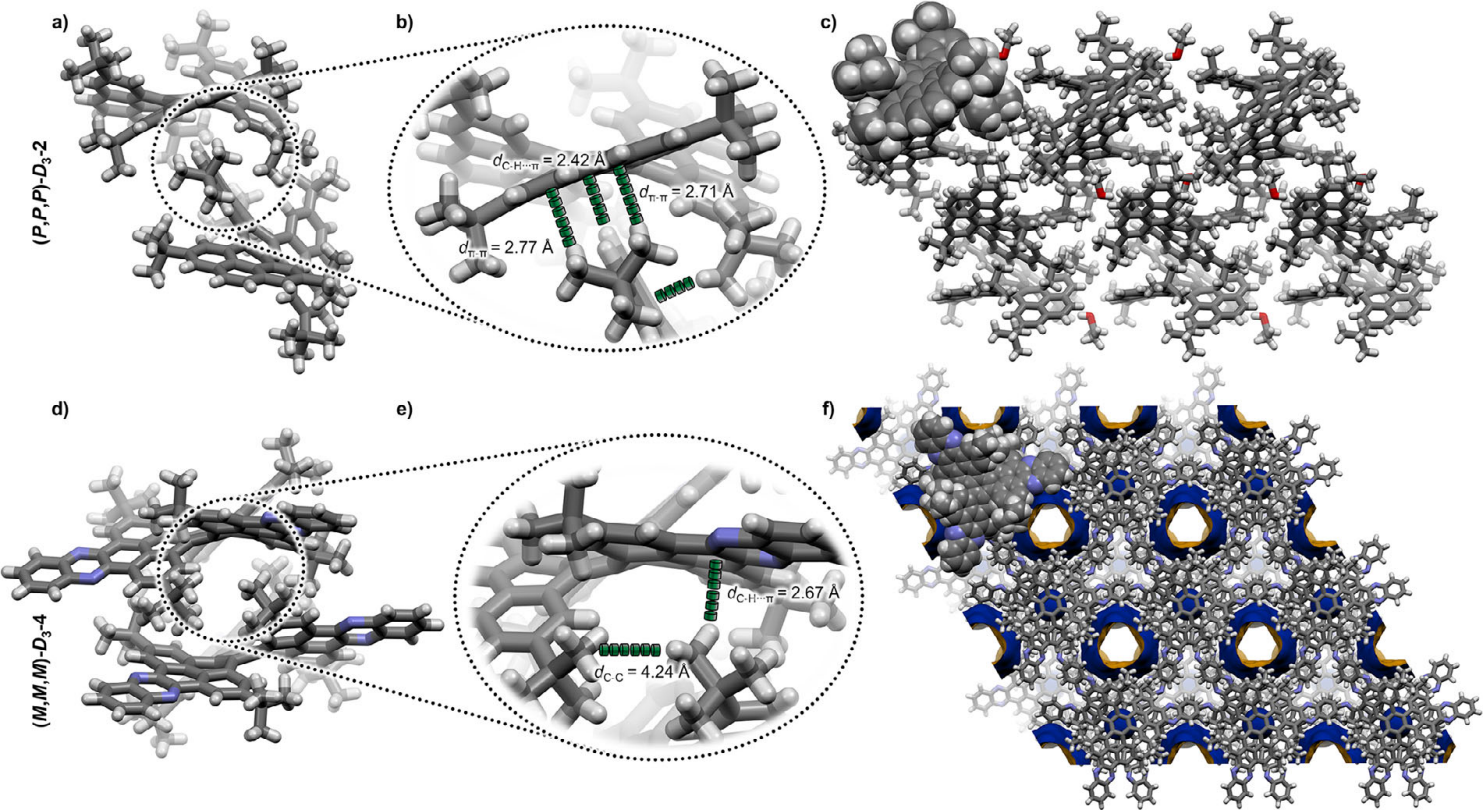
Single crystal X‐ray structures of (a–c) enantiopure **(*P*,*P*,*P*)‐*D*_3_‐2** and (d,e) enantiopure **(*M*,*M*,*M*)‐*D*_3_‐4** as capped stick models. First column: Dimeric alignments. Second column: Zoom‐ins of the intermolecular interactions within the dimeric alignments. Third column: crystal packing. In the packing representation one molecule each is shown as space fill models in element colors. (f) Contact surface with 1.82 Å probe radius and an grid spacing of approx. 0.3 Å. Grey: carbon; white: hydrogen; blue: nitrogen; red: oxygen.

By vapor diffusion of ethanol into a concentrated chloroform solution of enantiopure **(*P*,*P*,*P*)**‐***D*_3_‐2** crystals suitable for SCXRD were obtained (Figure 10). In contrast to ***rac*‐*D*_3_‐2** (triclinic space group *P* $\bar{1}$) **(*P*,*P*,*P*)**‐***D*_3_‐2** crystallizes in the orthorhombic space group *P*2_1_2_1_2_1_ with *Z*  =  4. The nonplanarity of the pyrene moieties of **(*P*,*P*,*P*)**‐***D*_3_‐2** matches the one of ***rac*‐*D*_3_‐2** with 0.10 but this time the molecules are less contorted against the central benzene unit with ∡  =  21° (***rac*‐*D*_3_‐2**: ∡  =  27°).

In contrast to the crystal structure of ***rac*‐*D*_3_‐2**, in which most of the intermolecular interactions were promoted by disordered solvent molecules, for **(*P*,*P*,*P*)**‐***D*_3_‐2** edge‐to‐face π‐stacking (*d*_π‐π_ *=* 2.71‐2.77 Å) and CH∙∙∙π‐interactions (*d*_C‐H∙∙∙π_ *=* 2.42 Å) of *tert*‐butyl‐groups with the π‐systems of adjacent pyrene subunits were found (Figures 10a and b). In combination with dispersion interactions and interactions with enclathrated ethanol molecules, sheets in the crystallographic *ab*‐plane are created and the overall packing is denser than for the racemic crystal (Figure 10c).

Similar to the racemic mixture ***rac*‐*D*_3_‐4**, crystals of enantiopure **(*M*,*M*,*M*)**‐***D*_3_‐4** were obtained by slow evaporation of a chloroform solution. **(*M*,*M*,*M*)**‐***D*_3_‐4** crystallizes in the chiral space group *P*6_3_22 (Figure 9d‐f). The packing is again driven by dispersion interactions of *tert*‐butyl groups of adjacent molecules (*d*_C‐C _ =  4.24 Å) (Figure 10e) in addition to CH∙∙∙π‐interactions with *d*_C‐H∙∙∙π _ =  2.67 Å of the *tert*‐butyl groups with the aromatic planes of close molecules.

**FIGURE 11:**
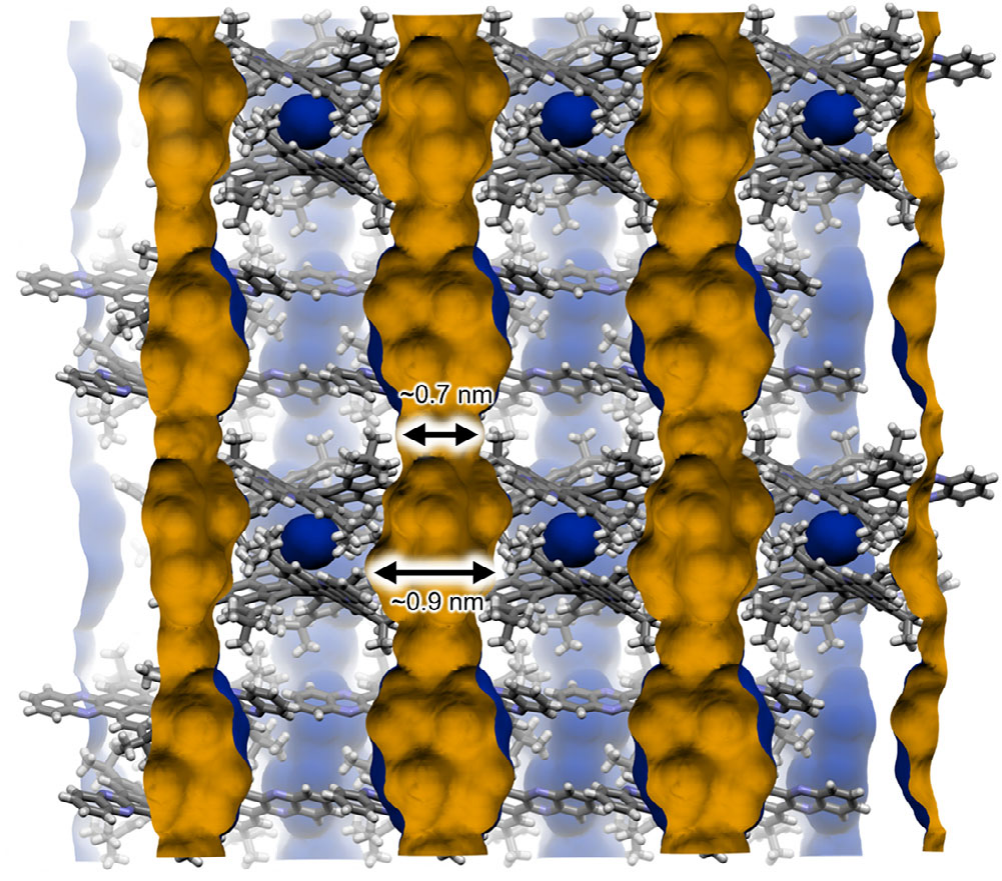
Crystal packing of **(*M*,*M*,*M*)‐*D*_3_‐4** with a contact surface with 1.82 Å probe radius and an approx. grid spacing of 0.3 Å.

While in the packing of ***rac*‐*D*_3_‐4** no connected pores are formed, **(*M*,*M*,*M*)**‐***D*_3_‐4** shows one‐dimensional channels along the crystallographic *c*‐axis in addition to spherical pores inside the dimers with a calculated accessible specific surface area of 846 m^2^ g^−1^ (Figures 10f and 11). The voids represent 33.6% of the unit cell volume and have a diameter of *d* ∼ 0.7 nm at the smallest and *d* ∼ 0.9 nm on the widest sections, which is in the range of the ion radius of solvated Li^+^‐cations (*r*_Li_ = 0.59‐0.76 nm) [70]. Therefore, this material is interesting to study lithium‐ion transport coupled with electron‐transport, which will be studied in the near future.

## 3 Conclusion

In this study, the post‐functionalization of previously reported *C*_2_‐symmetrical tripyrenylene ***rac*‐*C*_2_‐2** was achieved. Sixfold oxidation of the K‐regions and subsequent condensation with *o*‐phenylene diamine resulted in *C*_2_‐symmetrical π‐extended nitrogen containing PAHs. Both tripyrenylene and hexaone underwent a symmetry change to *D*_3_ and consequently enabled the isolation of threefold propeller shaped PAHs. The racemization barrier of *D*_3_‐symmetrical PAHs was calculated to be high enough for chiral resolution. Separation of the enantiomers of the *D*_3_‐tripyrenylene as well as *D*_3_‐trisphenanthrophenazines was achieved and the isolated enantiomers investigated by chiroptical methods and dissymmetry factors up to *g* = 0.024 have been obtained. Furthermore, the investigation of the isolated enantiomers by single crystal X‐ray diffraction revealed a porous crystalline lattice of *D*_3_‐trisphenanthrophenazine **(*M*,*M*,*M*)**‐***D*_3_‐4** with a pore‐size potentially enabling the application in solid‐state lithium‐ion batteries.

## Conflicts of Interest

The authors declare no conflicts of interest.

## Supporting information



The authors have cited additional references within the Supporting Information [31, 41, 42, 48, 49, 50, 51, 52, 53, 54, 55, 56, 57, 58, 59, 60, 61, 62, 66, 67, 71, 72, 73, 74, 75, 76, 77, 78, 79, 80, 81, 82, 83, 84, 85, 86, 87, 88, 89, 90, 91, 92, 93, 94, 95, 96, 97, 98, 99, 100, 101]. The supplementary crystallographic data for this paper are provided free of charge by the joint Cambridge Crystallographic Data Centre and Fachinformationszentrum Karlsruhe [102]. Parts of this article are adapted from the Ph.D. dissertation of Dr. Dennis Popp [103].

## Data Availability

The data that support the findings of this study are available from the corresponding author upon reasonable request.
