# Supplementary material for: Post‐Modification of Tripyrenylenes to Enantiopure π‐Extended D 3‐Symmetric Propellers
Source: Chemistry. 2026 Jun 13;32(36):e71239. doi: 10.1002/chem.71239 (PMC13602892; doi:10.1002/chem.71239)
Supplement: Supplementary file 1 [file CHEM-32-e71239-s001.pdf]

Post-Modification of Tripyrenylenes to Enantiopure π-Extended D_3-Symmetric Propellers

Dennis Popp,[a],§ Melanie Bruttel,[a],§ Sebastian Möbert,[a] Anusha Sounder Rajan,[a] Christoph Keck,[a] Darius Boehm,[a] Frank Rominger,[a] Sven M. Elbert,[a] Michael Mastalerz*[a]

[a] D. Popp, M. Bruttel, S. Möbert, A. S. Rajan, C. Keck, D. Boehm, Dr. F. Rominger, Dr. S. M. Elbert, Prof. Dr. M. Mastalerz
Organisch-Chemisches Institut, Ruprecht-Karls-Universität Heidelberg
Im Neuenheimer Feld 272, 69120 Heidelberg
E-mail: michael.mastalerz@oci.uni-heidelberg.de

§ these authors contributed equally.

-Supporting Information-

Table of Content

1. General Remarks S2

2. Synthetic Procedures S4

3. NMR Spectra S9

3.1 RT-NMR .... S**Fehler! Textmarke nicht definiert.**

3.2 VT-NMR S24

4. IR Spectra S26

5. UV/Vis Absorption & Fluorescence Spectra S28

6. CD Spectra S31

7. Mass Spectra S32

8. Crystallographic Data S35

9. Computational Details S41

9.1 General Remarks S41

9.2 Coordinates – Ground States S41

9.3 Coordinates – Transition States S65

9.4 Frontier Molecular Orbitals S76

9.5 TD-DFT S80

10. References S83

1. General Remarks

Materials: **1** was synthesized as previously reported.[S1] All chemicals and solvents used were purchased from abcr, Acros Organics, BLDpharm, Carbolution, Carl Roth, Deutero, Fisher Scientific, Honeywell, Sigma-Aldrich, Grüssing, Merck, TCI or VRW Chemicals and were used without further purification unless otherwise noted. Anhydrous acetonitrile, dichloromethane, diethyl ether or toluene were dispensed by a Solvent Purification System MB SPS-800, tetrahydrofuran was either dispensed by SPS-800 or dried over freshly prepared sodium wire, distilled and stored over molecular sieve 4 Å, hexane was dried using activated molecular sieve 4 Å. Unless noted otherwise, all reactions were performed under standard conditions (25°C, 1023 mbar). Inert conditions were achieved by evacuating the flasks, bake out and flooding with Argon (three cycles). Complete removal of solvents was achieved by high vacuum (10^{-2} mbar). Unless otherwise noted, room temperature (r.t.) denotes a temperature of 20 °C or 293.15 K.

Thin layer and flash column chromatography: POLYGRAM® SIL G/UV_{254} gel plates sold by Machery-Nagel were used for analytical normal phase thin layer chromatography (TLC). Detection was accomplished using UV-Light (254 nm or 360 nm). Silica gel 60 (40-63 µm / 230-400 mesh ASTM) sold by Machery-Nagel was used for flash column chromatography.

High performance liquid chromatography (HPLC): Analysis of compounds with an analytical HPLC was achieved using an LC-40D pump unit, a SIL-40 autosampler, a CTO-40C column oven and a DGU-405 degassing unit sold by Shimadzu. Separation was achieved using silica gel column amylose-based column (CHIRALPAK® IA) with tris-(3,5-dimethylphenylenecarbamate) as residue.

Complex mixtures of products were separated and purified using a preparative HPLC consisting of a LC-20AP pump unit, an FCV-$20AH_2$ valve unit and a SPD-M20A PDA detector unit by Shimadzu. Separation was achieved by a Restek ultra silica 5 µm (250 x 21.2 mm) column, in which the compound mixture was solved in HPLC-grade solvents and injected using a 500 µL, 1000 µL or a 5 mL injection loop.

Chiral high performance liquid chromatography (chiral HPLC): Chiral HPLC-analysis was achieved by using a combination of a LC40D pump unit, a SIL-40 autosampler unit, a CTO-40C column oven, a SPD-M20A detector and a DGU-405 degassing unit of Shimadzu. The column used had an amylose (CHIRALPAK® IA) backbone and Tris-(3-5-dimethylphenylcarbamat)-residue. Solvents used were HPLC-grade heptane, *iso*-propanol or DCM.

Nuclear magnetic resonance spectroscopy (NMR-spectroscopy): A Bruker Avance III 300 (^{1}H: 300 MHz, ^{13}C: 75 MHz, ^{19}F: 283 MHz), a Bruker Avance DRX 300 (^{1}H: 300 MHz, ^{13}C: 75 MHz), a Bruker Avance III 500 (^{1}H: 500 MHz, ^{13}C: 126 MHz), a Bruker Avance III 600 (^{1}H: 600 MHz, ^{13}C: 151 MHz) and a Bruker Avance Neo 700 (^{1}H: 700 MHz, ^{13}C: 176 MHz) NMR machines were used to measure NMR-spectra. If not noted otherwise NMR spectra were recorded in $CHCl_3$-d_1 at room temperature.

Low temperature NMR spectra were measured in THF-d_8, high temperature NMR spectra were measured in *o*-DCB-d_4. Chemical shifts δ are given in ppm in relation to non-deuterated residues of solvents ($CHCl_3$: δ_H = 7.26 ppm, δ_C = 77.2 ppm, THF-d_8: δ_H = 3.58 ppm, 1.72 ppm, o-DCB: δ_H = 7.19 ppm, δ_H = 6.93 ppm), coupling constants J are given in Hz. The following abbreviation are used for multiplicities: s = singlet, d = doublet, t = triplet, q = quartet, m = multiplet and combinations thereof.

Mass spectroscopy (MS): For the measurement of mass spectra a Bruker Auto-Flex speed MALDI-TOF mass spectrometer, and a Bruker tims-TOF mass spectrometer was available. For MALDI measurements, *trans*-2-[3-(4-*tert*-butylphenyl)-2-methyl-2-propenyliden]malo-nonitril DCTB was used as matrix material.

Melting points: For the measurement of uncorrected melting points a Büchi B-540 or a Büchi m-565 melting point device was used.

Infrared spectroscopy (IR): A Bruker Lumos Fourier-Transformations spectrometer in combination with a ZnSe ATR crystal was used to measure infrared spectra. Intensity of bands are given with the following abbreviations: s (strong), m (medium), w (weak), br (broad), v (very).

UV-Vis/Fluorescence Spectroscopy (UV-Vis): For the measurement of UV-Vis and fluorescence spectra, a JASCO UV-Vis V670 spectrometer and a Jasco FP6500 spectrofluorometer was used. Scattered light of the excitation was cut and offsets were compensated by subtracting a calculated baseline in Origin (see Software).

Circular Dichroism Data (CD): For the measurement of CD spectra, a JASCO J-1500 spectral polarimeter with a Julabo F250 cooler and FLMS-526 N_2 gas flow was used. To compensate offsets

Crystal Structure Analysis: A Bruker APEX II ($\lambda_{MoK\alpha}$ = 0.71073 Å) or a Stoe Stadivari ($\lambda_{CuK\alpha}$ = 1.54186 Å) diffractometer was used to obtain reflexes for crystal structure. Intensities were corrected for Lorentz and polarization effects, an empirical scaling and absorption correction was applied using SADABS[S2] X-Area LANA 1.70.0.0 (STOE, 2017). Based on the Laue symmetry of the reciprocal space, structure solved with SHELXT-2014[S3] or XM 2013/2[S3] and refined against F^2 with a Full-matrix least-squares algorithm using the SHELXL-2018/3 (Sheldrick, 2018) software[S4], hydrogen atoms were treated using appropriate riding models.

Photoluminescence Quantum Yields (PLQY): Quantum yields were determined by the absolute method using an Ulbricht sphere (6 inch, band widths excitation: 3 nm; band widths emission: 3 nm; integration time 0.5 s) and a PTI QuantaMaster 40 equipped with a HAMAMATSU R928P Photomultiplier. Or Absolute photoluminescence quantum yields (Φ_{fl}) were measured in chloroform solution (OD < 0.1) at room temperature using a JASCO FP-8500 fluorescence spectrometer with a JASCO ILF-835 (100 mm) integrating sphere.

2. Synthetic Procedures

Unless otherwise noted, starting material used and obtained products were racemic mixtures. Only one enantiomer is depicted for simplicity reasons.

2,5,10,13,18,21-Hexa-*tert*-butyltripyrenylene *rac-C*$_2$-2

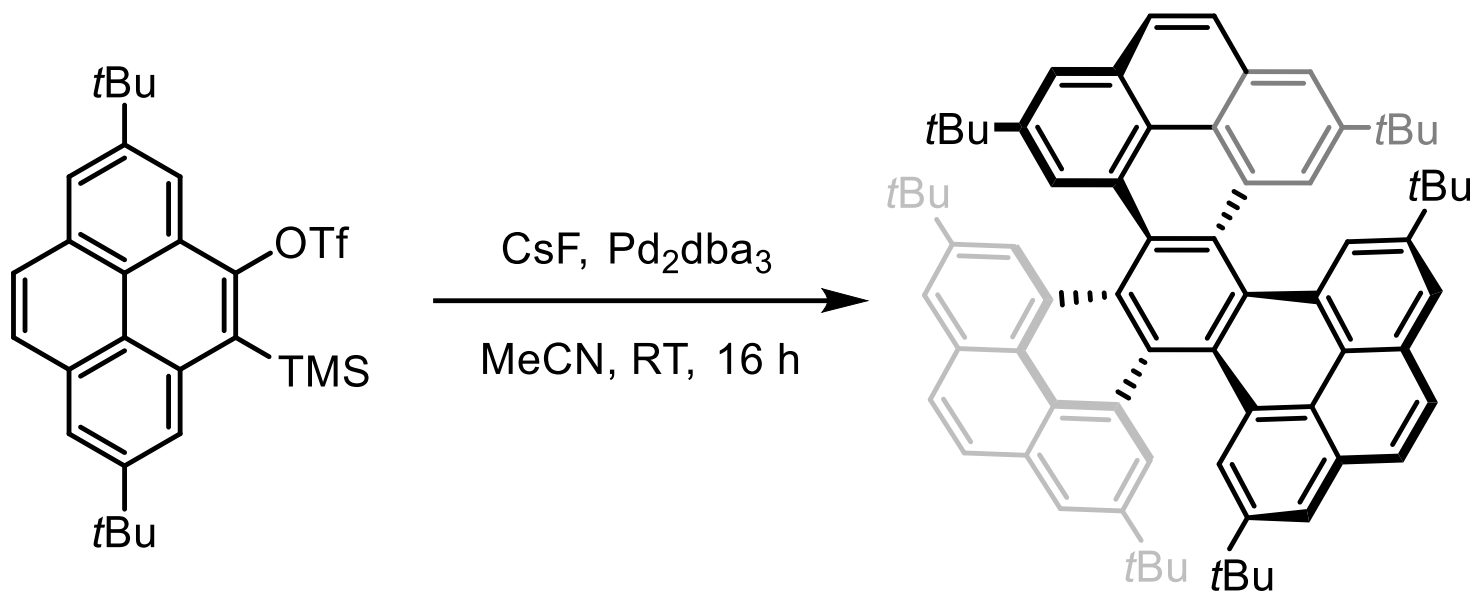

1 **rac-C$_2$-2**

Pyrene TMS-triflate **1** (985 mg, 1.84 mmol) and Pd_2dba_3 (421 mg, 460 µmol) were suspended in dry MeCN (37 mL) in an inert atmosphere and CsF (1.84 g, 12.1 mmol) was added under argon flow. The resulting dark grey suspension was stirred for 16 hours at room temperature, filtered through a Celite® pad, flushed with DCM (250 mL) and the solvent was removed under reduced pressure. The crude product was purified by flash column chromatography (SiO_2, petroleum ether/DCM 20:1) (R_f = 0.3) to give 245 mg (43%) of ***rac-C*$_2$-2** as pale-yellow powder.

M.p. > 338 °C (dec.). ^{1}H NMR ($CDCl_3$, 300 MHz) δ[ppm]: 8.62 (s, 2H), 8.50 (s, 2H), 8.36 (s, 2H), 8.21 (s, 2H), 8.12 (s, 2H), 8.06 (s, 4H) 8.02 (d, J = 3.66 Hz, 4H), 1.43 (s, 18H), 1.17 (s, 18H), 1.06 (s, 18H). Analytical data are in accordance with the literature.[S1]

2,5,10,13,18,21-Hexa-tert-butyltripyrenylene *rac-D*$_3$-2

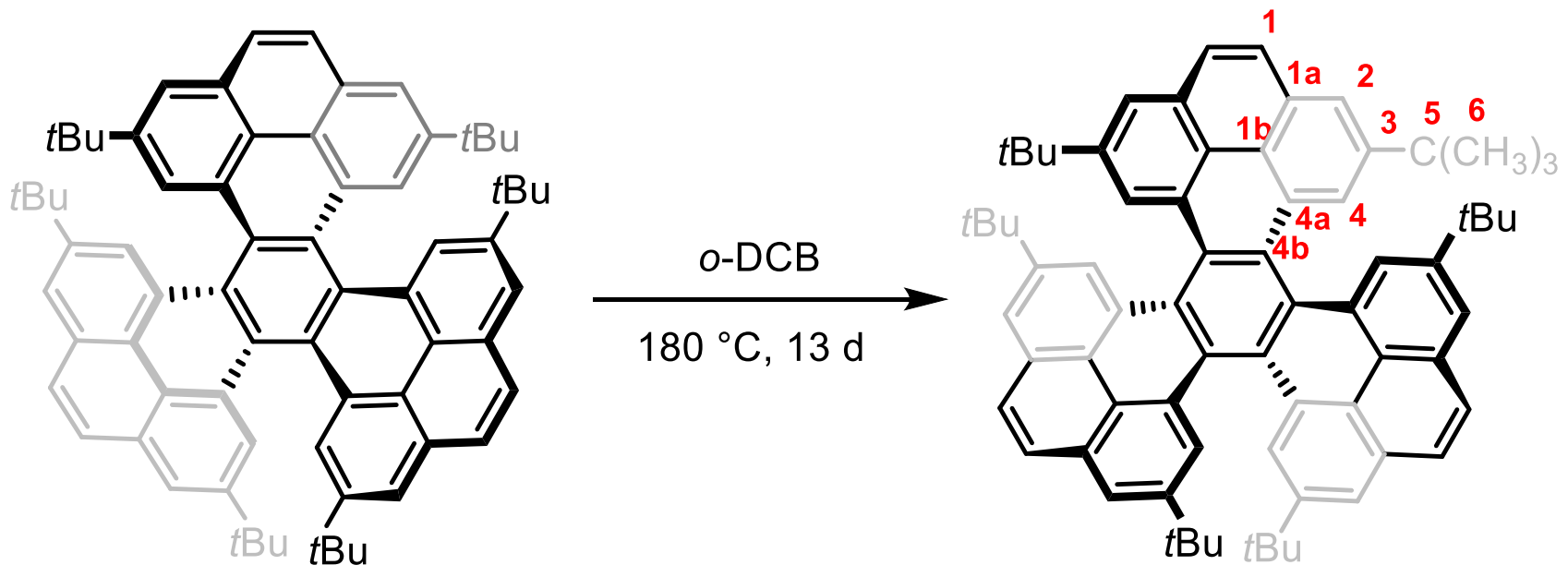

rac-C$_2$-2 **rac-D$_3$-2**

A solution of ***rac-C*$_2$-2** (173 mg, 184 µmol) in *o*-DCB (15 mL) was stirred at 180 °C for 13 days. After cooling to room temperature, the solvent was removed under reduced pressure to give 172 mg (quant.) of ***rac-D*$_3$-2** as yellow powder.

M.p.: decomposition >300 °C. ^{1}H NMR ($CDCl_3$, 700 MHz) δ [ppm]: 8.33 (d, J = 2.10 Hz, 6H, H-4), 8.08 (d, J = 2.10 Hz, 6H, H-2), 8.06 (s, 6H, H-1), 1.14 (s, 54H, H-6). ^{13}C NMR ($CDCl_3$, 176 MHz) δ

[ppm]: 147.0 (C-3), 130.6 (C-1a), 130.5 (C-4a), 130.1 (C-4b), 127.4 (C-1), 126.0 (C-4), 122.2 (C-1b), 122.2 (C-2), 35.1 (C-5), 31.7 (C-6). IR (RT, ATR) $\tilde{\nu}$ [cm^{-1}]: 3029 (w), 2959 (m), 2901 (w), 2866 (w), 1908 (vw), 1747 (w), 1601 (w), 1585 (w), 1549 (w), 1463 (m), 1429 (w), 1362 (m), 1328 (w), 1294 (w), 1263 (w), 1241 (w), 1229 (w), 1215 (w), 1198 (w), 1178 (w), 1147 (w), 1058 (w), 1026 (vw), 973 (vw), 933 (w), 905 (w), 881 (vs), 863 (w), 825 (w), 796 (w), 770 (w), 739 (w), 732 (m), 706 (vs), 688 (w), 666 (vw), 648 (w), 630 (vw). UV/Vis (DCM) λ [nm] (*log* ε): 288.8 (4.94), 360 (4.60), 399 (4.83) Fluorescence (DCM, λ_{Ex} = 395 nm) λ_{Em} [nm]: 522, 548. Φ (DCM) = 2%. HR-MS (MALDI$^+$, DCTB): calcd for $C_{72}H_{72}$ ([M]$^+$): 936.5629, found: 936.5609.

2,5,10,13,18,21-Hexa-*tert*-butyltripyrenylene-7,8,15,16,23,24-hexaone *rac C*$_2$-3

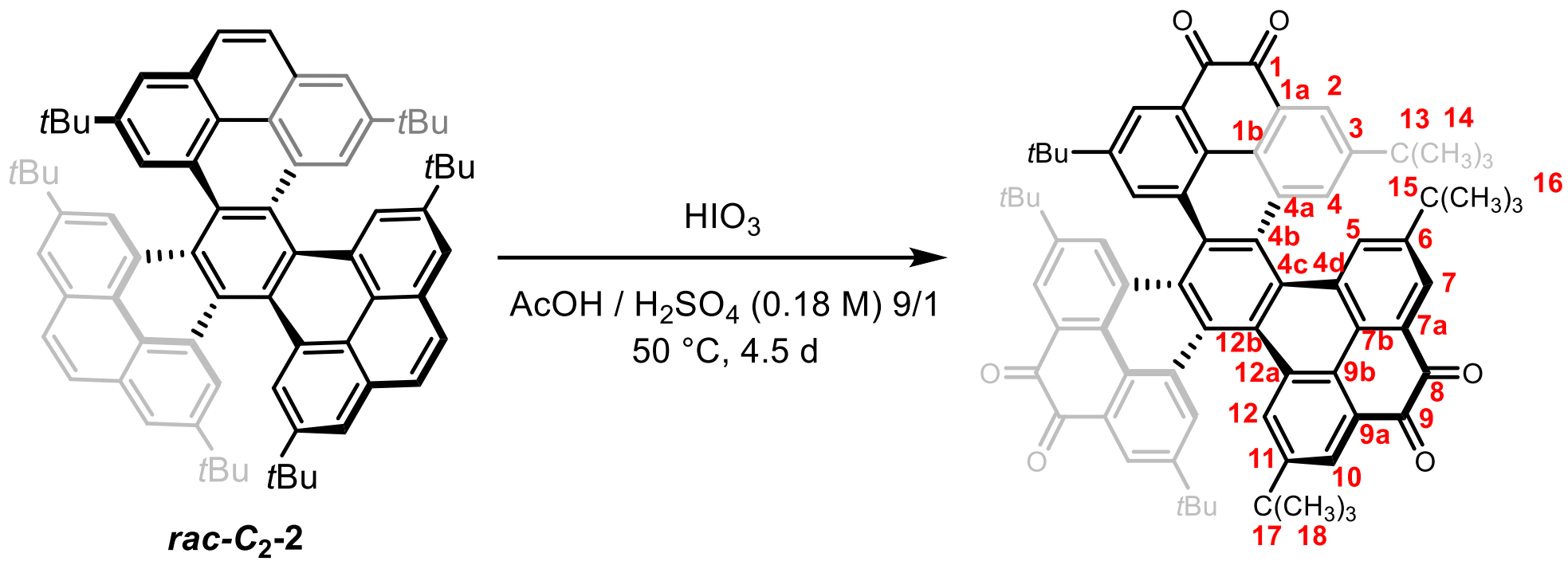

***rac-C*$_2$-2** ***rac-C*$_2$-3**

A mixture of ***rac-C*$_2$-2** (20 mg, 21 µmol) and HIO_3 (40 mg, 227 µmol) was suspended in a mixture of glacial AcOH (0.30 mL) and diluted aqueous H_2SO_4 (0.18 M, 0.04 mL).[S5] The orange suspension was stirred for 4.5 days at 50 °C. After cooling to room temperature, H_2O (1 mL) was added. The resulting dark red suspension was extracted with DCM (3×2 mL). The combined extract was washed with $Na_2S_2O_3$-solution (1 M, 2×2 mL), H_2O (2×2 mL) and brine (5 mL), dried over anhydrous $MgSO_4$ and the solvent was removed under reduced pressure. Crude ***rac-C*$_2$-3** was purified by filtration through a plug of silica (EA) and normal phase HPLC (SiO_2 column, 10 mL/min) using *n*-heptane/EA (4:1) as eluent to give after drying 19 mg (88%) of ***rac-C*$_2$-3** as orange-red powder.

M.p. >350 °C (dec.). ^{1}H NMR ($CDCl_3$, 700 MHz) δ [ppm]: 8.61 (d, *J* = 1.82 Hz, 2H, H-10), 8.48 (d, *J* = 2.03 Hz, 2H, H-4), 8.47 (d, *J* = 1.96 Hz, 2H, H-7), 8.46 (d, *J* = 1.96 Hz, 2H, H-2), 8.44 (d, *J* = 1.89 Hz, 2H, H-12), 8.28 (d, *J* = 1.89 Hz, 2H, H-5), 1.34 (s, 18H, H-18), 1.17 (s, 18H, H-16), 1.11 (s, 18H, H-14). ^{13}C NMR ($CDCl_3$, 176 MHz) δ[ppm]: 181.6 (C-9), 180.9 (C-8), 180.8 (C-1), 150.8 (C-11), 150.1 (C-3), 149.9 (C-6), 135.1 (C-5), 132.6 (C-12b), 132.2 (C-4), 132.1 (C12), 131.3 (-C$_q$), 130.9 (C$_q$), 130.2 (C2), 130.2 (-C$_q$), 129.8 (C$_q$), 129.5 (C-4c), 129.4 (C-7), 128.8 (C-1b), 128.4 (C10), 127.9 (-C$_q$), 127.6 (C7b), 127.3 (-C$_q$), 126.7 (C-4b), 125.9 (C-9b), 35.6 (C-17), 35.3 (C-13), 35.1 (C-15), 31.2 (C-18), 30.9 (C-16), 30.8 (C-14). IR (RT, ATR) $\tilde{\nu}$ [cm^{-1}]: 2962 (m), 2908 (m), 2878 (w), 2870 (w), 1734 (w), 1682 (vs), 1601 (m), 1543 (w), 1518 (w), 1475 (m), 1460 (m), 1418 (m), 1396 (w), 1366 (m), 1329 (w), 1315 (m), 1302 (w), 1283 (m), 1248 (m), 1229 (s), 1205 (m), 1188 (m), 1163 (w), 1144 (w),

1136 (w), 1086 (m), 1047 (m), 1028 (m), 1001 (w), 980 (w), 968 (w), 924 (m), 899 (m), 868 (m), 852 (m), 831 (m), 822 (m), 802 (m), 785 (m), 752 (m), 731 (m), 698 (m), 687 (m), 663 (m), 646 (m), 621 (m), 611 (m). UV/Vis- (DCM) λ [nm] (*log* ε): 282 (4.38), 337 (4.55), 351 (4.64), 399 (3.93). HR-MS (MALDI$^+$, DCTB): calcd. for $C_{72}H_{67}O_6$ ($[M+H]^+$): 1027.4933, found: 1027.4895.

2,5,10,13,18,21-Hexa-*tert*-butyltripyrenylene-7,8,15,16,23,24-hexaone *rac-D*₃-3

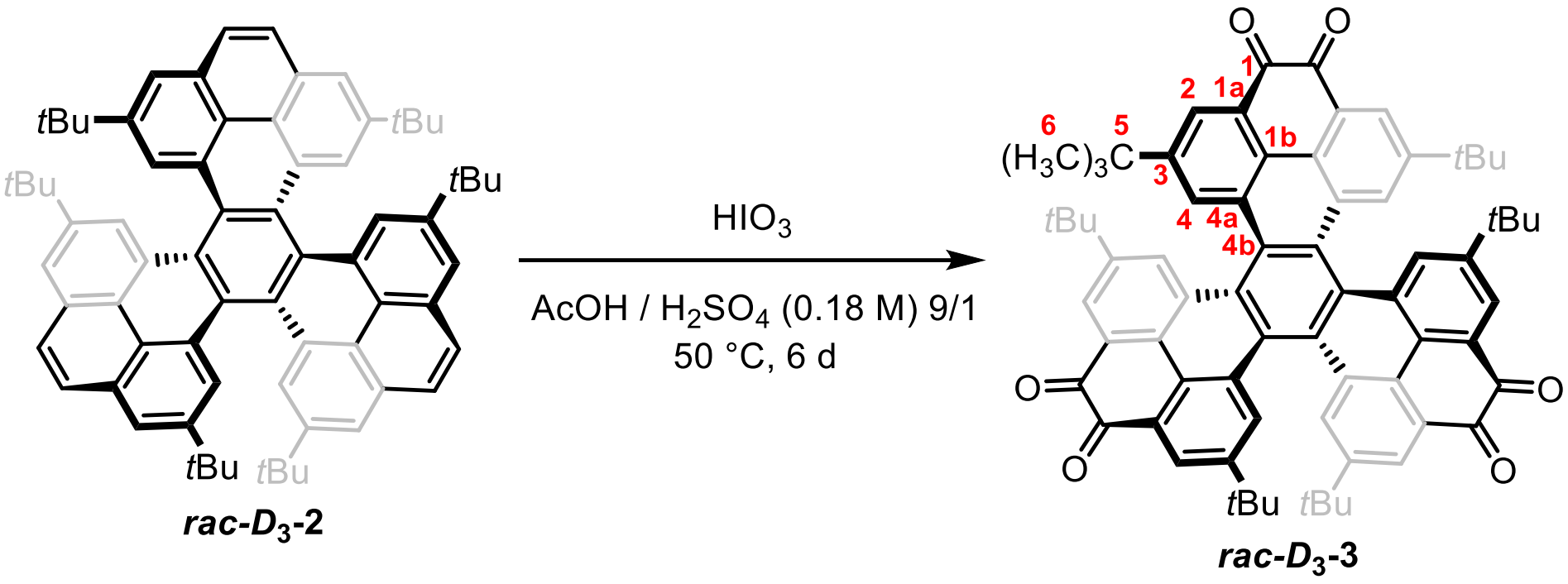

A mixture of ***rac-D*$_3$-2** (40 mg, 42 µmol) and HIO_3 (80 mg, 455 µmol) was suspended in a mixture of glacial AcOH (0.6 mL) and aqueous H_2SO_4 (0.18 M, 0.08 mL, 10 vol%). The dark red suspension was stirred for 6 days at 50 °C. After cooling to room temperature H_2O (2 mL) was added. The suspension was extracted with DCM (3×2 mL). The combined extract was washed with $Na_2S_2O_3$-solution (1 M, 3×3 mL), H_2O (3×3 mL) and brine (8 mL), dried over anhydrous $MgSO_4$ and the solvent was removed under reduced pressure. Crude ***rac-D*$_3$-3** was purified by filtration through a plug of silica (EA) to give after drying in vacuum 38 mg (87%) of ***rac-D*$_3$-3** as orange-red powder.

M.p. >354 °C (dec.). ^{1}H NMR ($CDCl_3$, 600 MHz) δ [ppm]: 8.53 (d, J = 1.86 Hz, 6H, H-2), 8.35 (d, J = 1.86 Hz, 6H, H-4), 1.16 (s, 54H, H-6). ^{13}C NMR ($CDCl_3$, 151 MHz) δ [ppm]: 180.7 (C-1), 150.5 (C-3), 134.0 (C-4), 130.9 (C-1/4a), 129.6 (C-1a/4a), 128.6 (C-2), 128.5 (C-4b), 126.5 (C-1b), 35.3 (C-15), 31.0 (C-16). IR (RT, ATR) $\tilde{\nu}$ [cm^{-1}]: 2959 (m), 2937 (m), 2918 (m), 2895 (m), 2868 (m), 1738 (m), 1730 (m), 1722 (m), 1711 (m), 1703 (m), 1680 (s), 1649 (m), 1643 (m), 1599 (m), 1477 (m), 1468 (m), 1462 (m), 1452 (m), 1441 (m), 1416 (m), 1366 (m), 1312 (m), 1302 (m), 1283 (m), 1229 (m), 1202 (m), 1153 (m), 1105 (m), 1086 (m), 1049 (m), 1022 (m), 901 (m), 852 (m), 735 (m), 704 (m), 681 (m), 665 (m), 511 (m), 476 (m), 459 (s), 444 (s), 434 (m), 417 (m). UV/Vis- ($CHCl_3$) λ [nm] (*log* ε): 286 (4.43), 367 (4.79), 480 (3.59). HR-MS (MALDI$^+$, DCTB): calcd. for $C_{72}H_{67}O_6$ ($[M+H]^+$): 1027.4933, found: 1027.4867.

2,5,10,13,18,21-Hexa-*tert*-butyltripyrenylene-7,8,15,16,23,24-hexaone *rac-D*$_3$-3

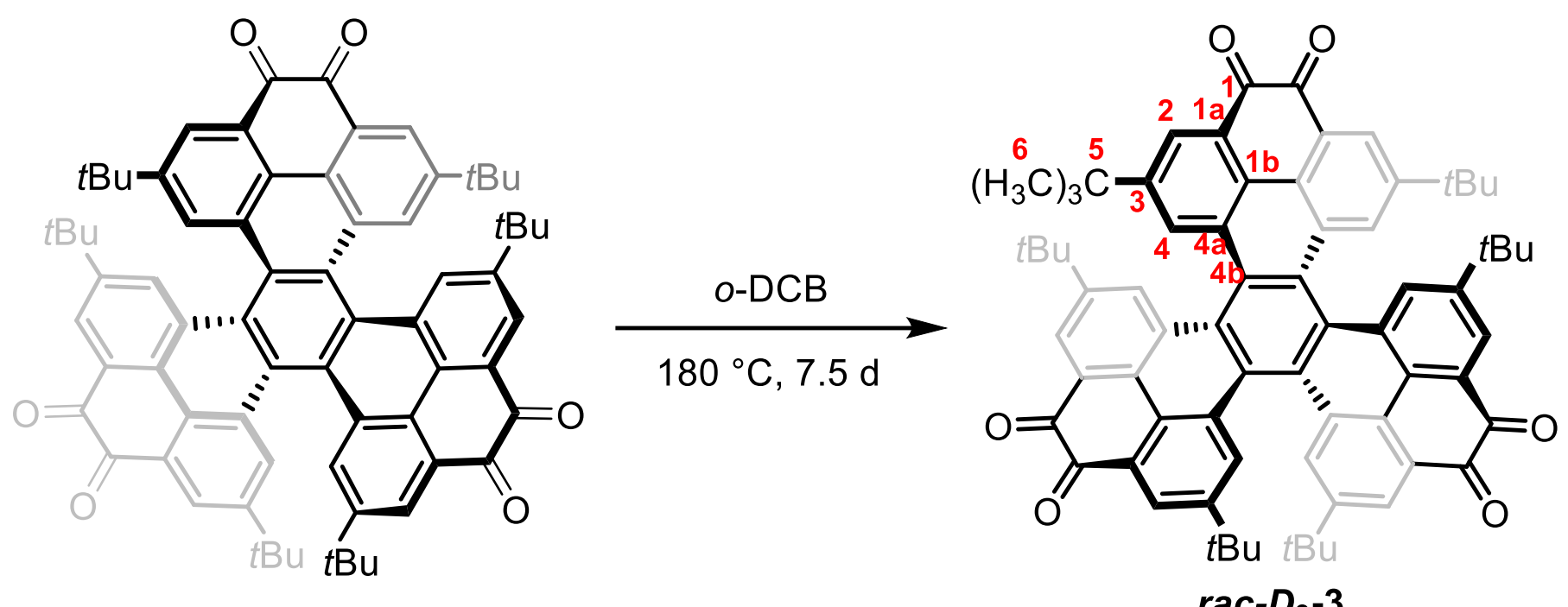

A solution of ***rac-C*$_2$-3** (4.0 mg, 3.89 µmol) in *o*-DCB (0.5 mL) was stirred at 180 °C for 7.5 days. The solvent was removed under reduced pressure to give ***rac-D*$_3$-3** as red solid in quantitative yield. Analytical data is in accordance to the one reported above.

Trisphenanthophenazine *rac-C*$_2$-4

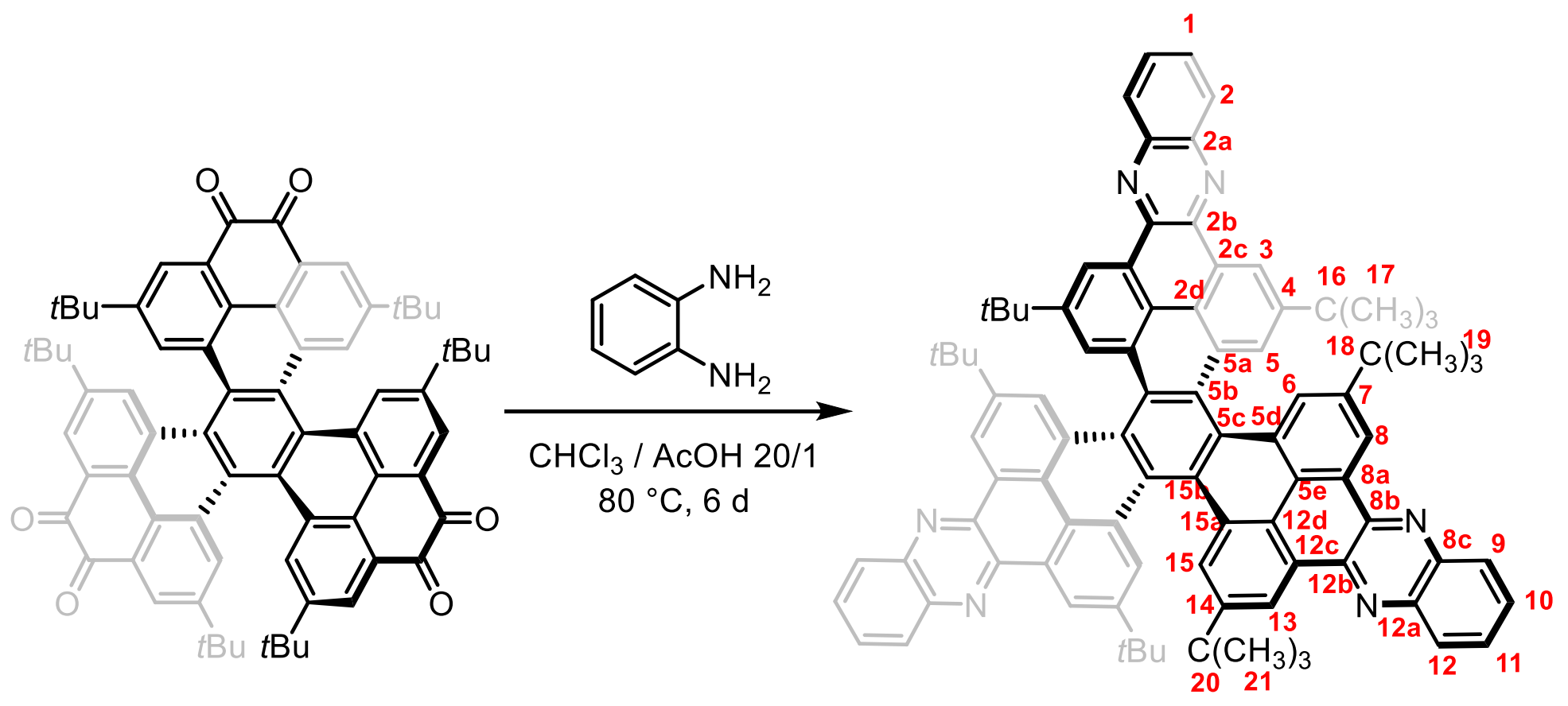

***rac-C*$_2$-3** (10 mg, 10.0 µmol) and 1,2-diaminobenzene (6.5 mg, 60.1 µmol) were dissolved in a mixture of $CHCl_3$/AcOH (20:1, 0.27 mL) and stirred for 6 days at 80 °C. After cooling to room temperature, MeOH (1 mL) was added to the yellow to brown mixture and the resulting grey solid filtered and washed with MeOH (3×3 mL) and *n*-pentane (3 mL). The crude product was dried under reduced pressure to give 6 mg (48%) of ***rac-C*$_2$-4** as yellow solid.

M.p >360 C (dec.). ^{1}H NMR ($CDCl_3$, 600 MHz) δ [ppm]: 9.70 (d, J = 1.82 Hz, 2H, H-13), 9.49 (d, J = 2.03 Hz, 2H, H-3/8), 9.43 (d, J = 1.89 Hz, 2H, H-3/8), 8.76 (s, 2H, H-15), 8.67 (s, 2H, H-5/6), 8.55--8.53 (m, 4H, H2,5/6-), 8.51-8.49 (m, 2H, H-9/12), 8.48-8.46 (m, 2H, H-9/12), 7.98-7.96 (m, 2H, H-1), 7.94-7.91 (m, 4H, H-10, H-11), 1.54 (s, 18H, H21), 1.31- (s, 18H, H17/19), 1.15- (s, 18H, H-17/19). ^{13}C NMR ($CDCl_3$, 151 MHz) δ [ppm]: 148.8 (C-14), 148.1 (C-4/7), 147.9 (C-4/7), 144.4 (C-2b/8b), 144.3 (C-2b/8b), 144.2 (C-12b), 142.7 (C-2a/8c/12a), 142.6 (C-2a/8c/12a), 142.5 (C-2a/8c/12a), 134.1 (C15b), 131.2 (-C$_q$), 130.5 (C5/6/15), 130.0- (C-1/2/9/10/11/12)-, 129.8 (2C, C1/2/9/10/11/12), 129.6- (2C, C1/2/9/10/11/12), 129.4 (-C$_q$), 129.2 (C$_q$), 128.8 (C$_q$), 128.7 (C$_q$), 127.9 (C5/6/15),

127.5 (-C_q), 127.3 (C15), 126.7 (-C_q), 126.5 (C_q), 125.5 (C-2d/5e), 124.5 (C3/8), 123.3- (C-12d), 123.1 (C-3/8), 121.6 (C-13), 35.9 (C-20), 35.3 (2C, C-16, C-18), 31.9 (C-21), 31.6 (C-17/19), 31.4 (C-17/19). IR (RT, ATR) $\tilde{\nu}$ [cm^{-1}]: 3063 (m), 2955, 2907 (m), 2870 (m), 1605 (m), 1512 (m), 1477 (s), 1460 (m), 1425 (m), 1410 (m), 1393 (m), 1364 (s), 1337 (s), 1285 (m), 1263 (s), 1227 (s), 1202 (m), 1155 (m), 1128 (m), 1113 (s), 1022 (m), 1007 (m), 974 (m), 949 (m), 922 (m), 916 (m), 891 (s), 862 (m), 827 (m), 808 (m), 791 (m), 777 (s), 754 (vs), 735 (s), 702 (m), 689 (m), 671 (m), 648 (m), 635 (m), 611 (m), 582 (s), 573 (s), 554 (m), 534 (m), 519 (m), 492 (m), 474 (m), 449 (m), 426 (m). UV/Vis- ($CHCl_3$) λ [nm] (*log* ε): 261 (5.12), 287 (4.97), 373 (4.99), Fluorescence ($CHCl_3$, λ_{Ex} = 372 nm) λ_{Em} [nm]: 545. Φ (DCM): 12%. HR-MS ($MALDI^+$, DCTB): calcd. for $C_{90}H_{78}N_6$ ($[M]^+$): 1243.6317, found: 1243.6264.

Trisphenanthrophenazine *rac- D*$_3$-4

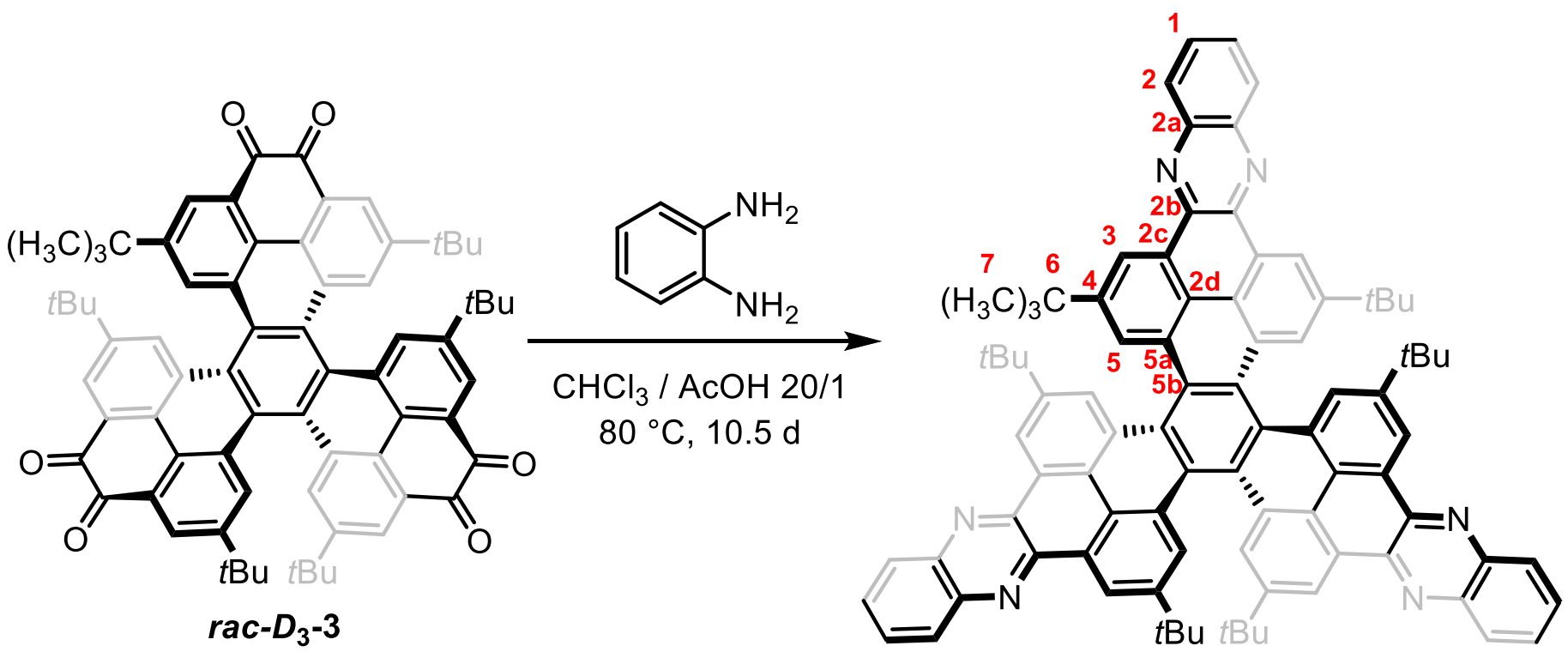

***rac-D*$_3$-3** (39 mg, 38 µmol) and 1,2-diaminobenzene (25 mg, 228 µmol) were dissolved in a mixture of $CHCl_3$/AcOH (20:1, 0.4 mL) and stirred for 10.5 d at 80 °C. After cooling to room temperature, MeOH (1 mL) was added to the yellow to brown mixture and the resulting yellow solid filtered and washed with MeOH (3×4 mL) and *n*-pentane (4 mL). The crude product was dried under reduced pressure to give 38.2 mg (81%) of ***rac-D*$_3$-4** as yellow solid.

M.p. >356 °C (dec.). ^{1}H NMR ($CDCl_3$, 700 MHz) δ [ppm]: 9.63 (d, *J* = 1.93 Hz, 6H, H3), 8.56 (d, *J* = 1.93 Hz-, 6H, H-5), 8.50-8.48 (m, 6H, H-1), 7.94-7.93 (m, 6H, H-2), 1.26 (s, 54H, H-7). ^{13}C NMR ($CDCl_3$, 176 MHz) δ [ppm]: 148.5 (C-4), 144.0 (C-2b), 142.5 (C-2a), 130.8 (C-2c), 129.9 (C-2), 129.7 (C-1), 129.6 (C-5b), 129.4 (C-5), 129.0 (C-5a), 123.8 (C-2d), 121.7 (C-3), 35.5 (C-6), 31.6 (C-7). IR (RT, ATR) $\tilde{\nu}$ [cm^{-1}]: 2959 (s), 2924 (s), 2903 (s), 2866 (s), 1605 (s), 1475 (s), 1464 (s), 1458 (s), 1394 (s), 1366 (s), 1340 (s), 1325 (s), 1265 (s), 1256 (s), 1227 (s), 1202 (m), 1161 (m), 1155 (m), 1130 (s), 1117 (s), 1107 (s), 1084 (s), 1067 (s), 1036 (s), 1026 (s), 962 (m), 926 (m), 895 (vs), 858 (m), 827 (m), 808 (s), 766 (vs), 737 (s), 706 (s), 687 (m), 669 (m), 648 (m), 619 (m). UV/Vis- ($CHCl_3$) λ [nm] (*log* ε): 262 (4.97), 289 (4.89), 379 (4.87), Fluorescence ($CHCl_3$, λ_{Ex} = 379 nm) λ_{Em} [nm]: 345, 358, 504, 540. Φ (DCM): 3%. HR-MS ($MALDI^+$, DCTB): calcd. for $C_{90}H_{78}N_6$ ($[M]^+$): 1243.6317, found: 1243.6303.

3. NMR Spectra

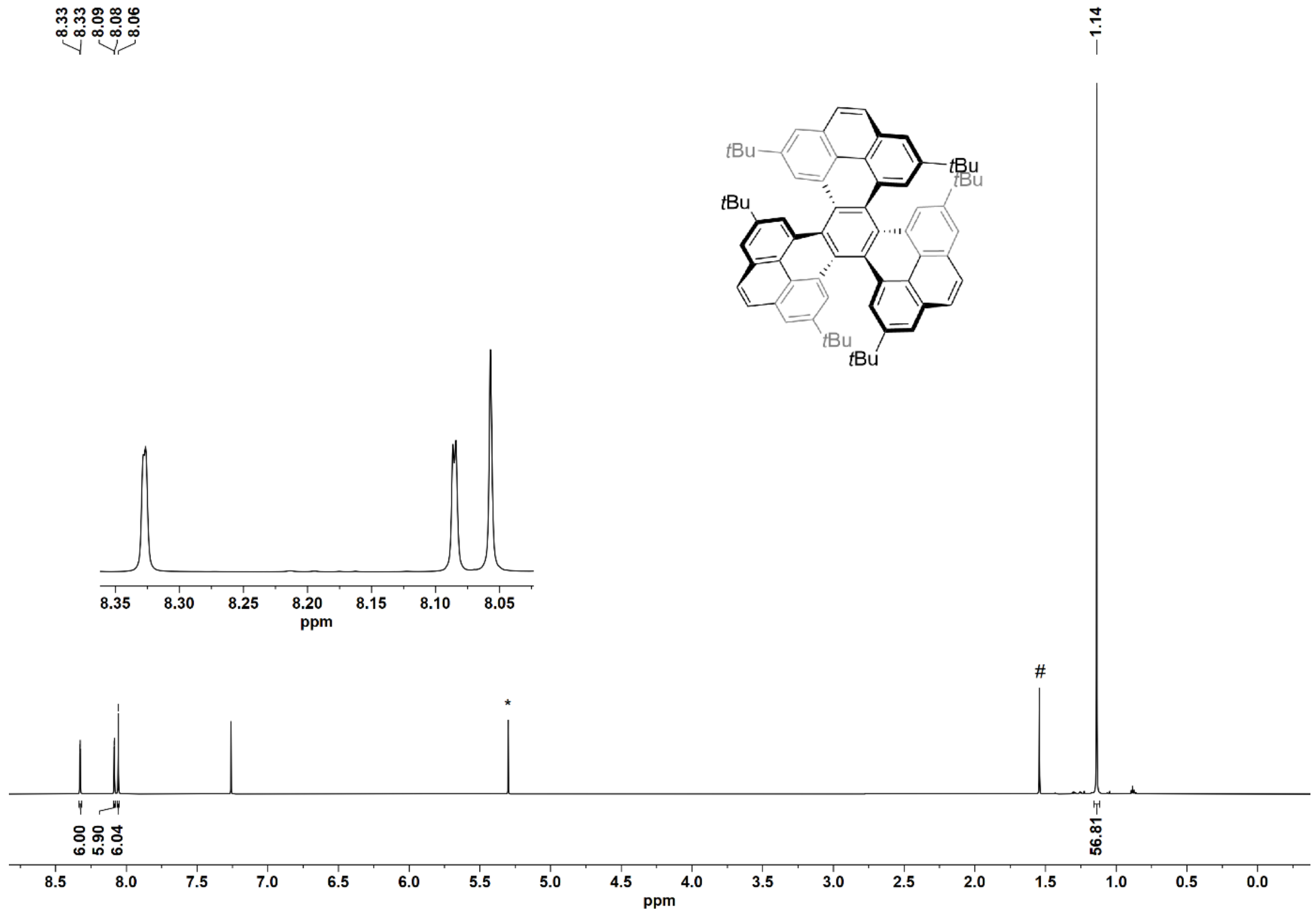

Figure S1: ^{1}H NMR spectrum ($CDCl_3$, 700 MHz, r.t.) of ***rac*-*D*$_3$-2**. #: Water. *: DCM.

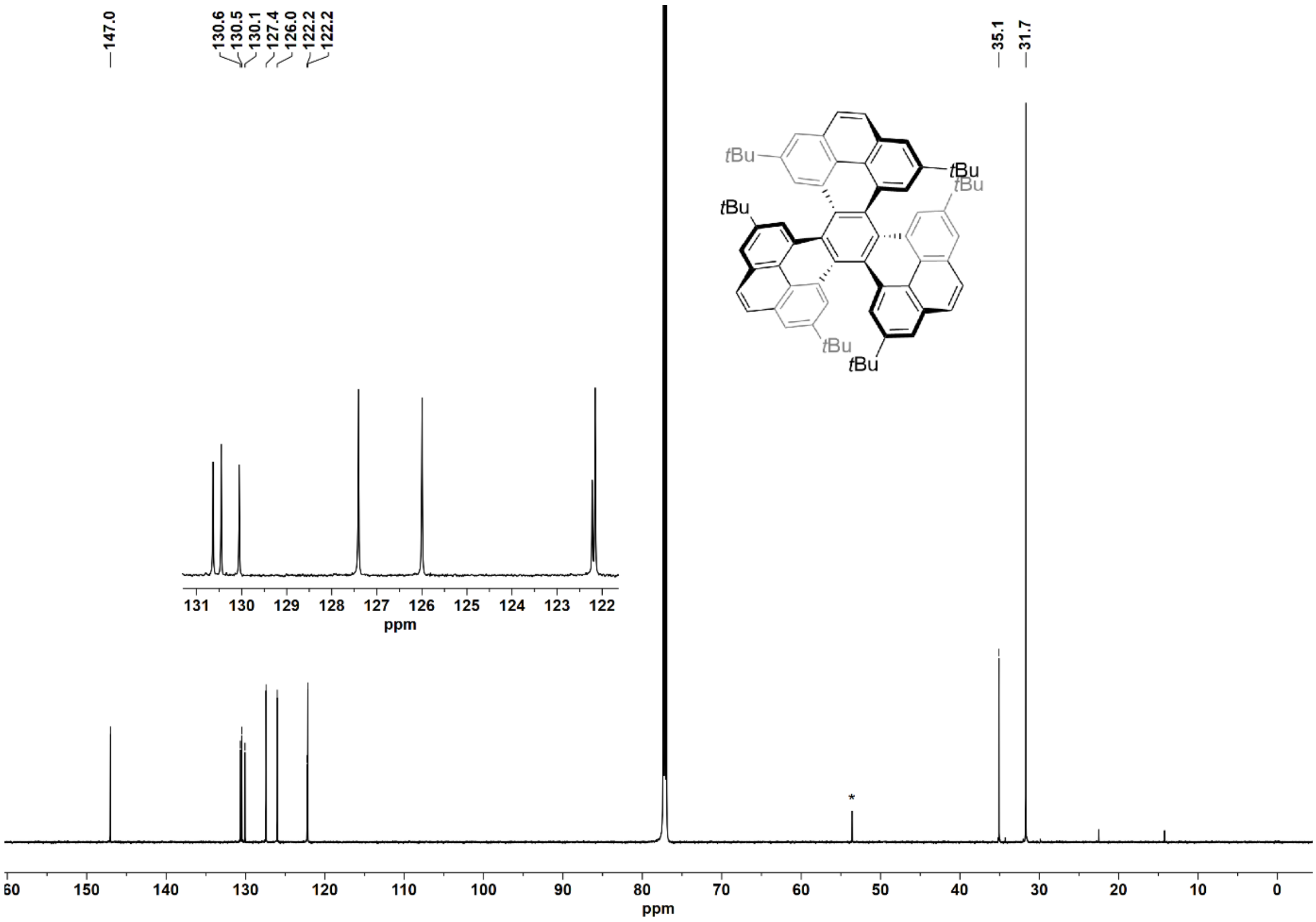

Figure S2: ^{13}C NMR spectrum ($CDCl_3$, 176 MHz, r.t.) of ***rac*-*D*$_3$-2**. *: DCM.

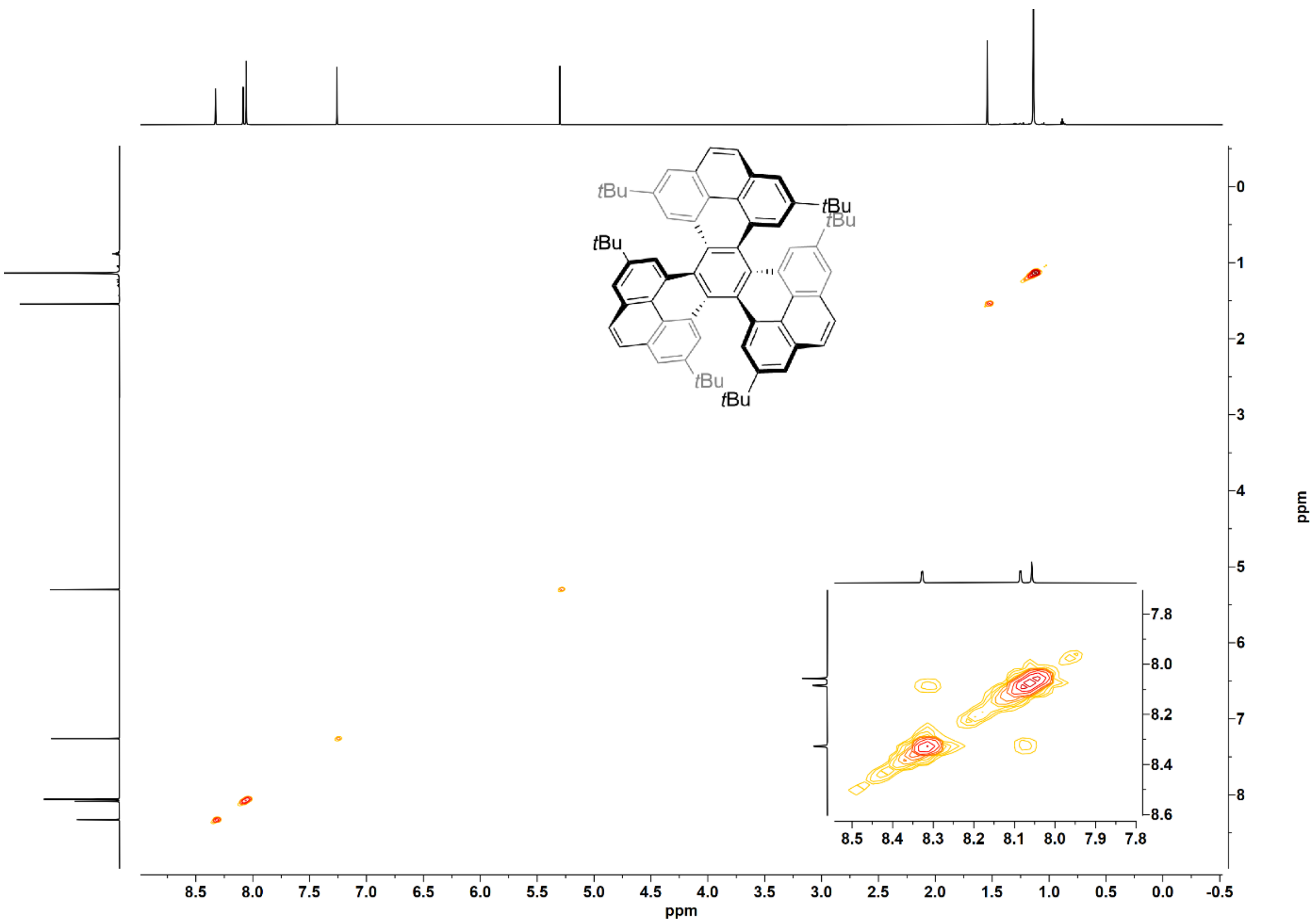

Figure S3: ^{1}H-^{1}H COSY NMR spectrum ($CDCl_3$, 700 / 700 MHz, r.t.) of ***rac-D*₃-2**.

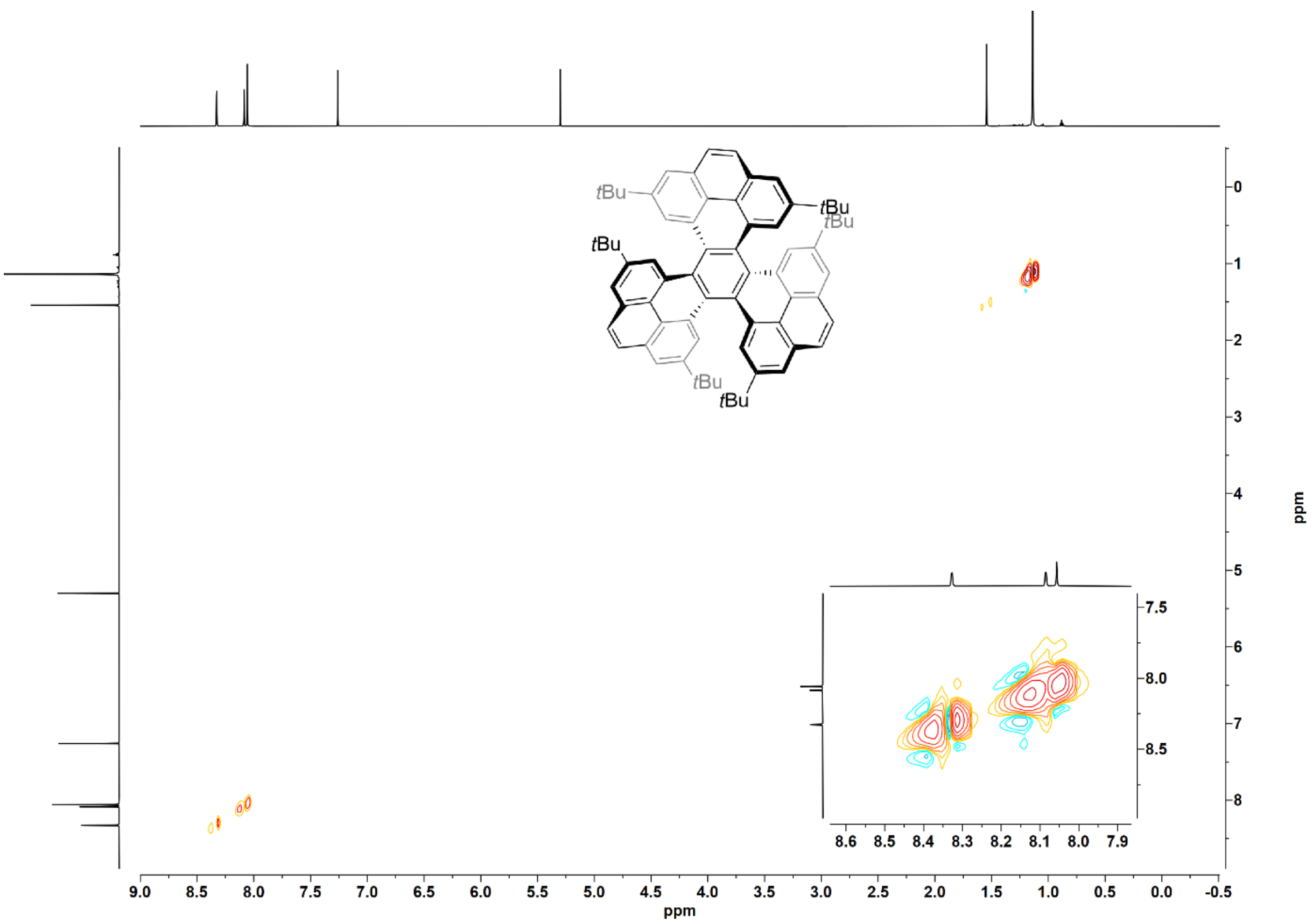

Figure S4: ^{1}H-^{1}H NOESY NMR spectrum ($CDCl_3$, 700 / 700 MHz, r.t.) of ***rac-D*₃-2**.

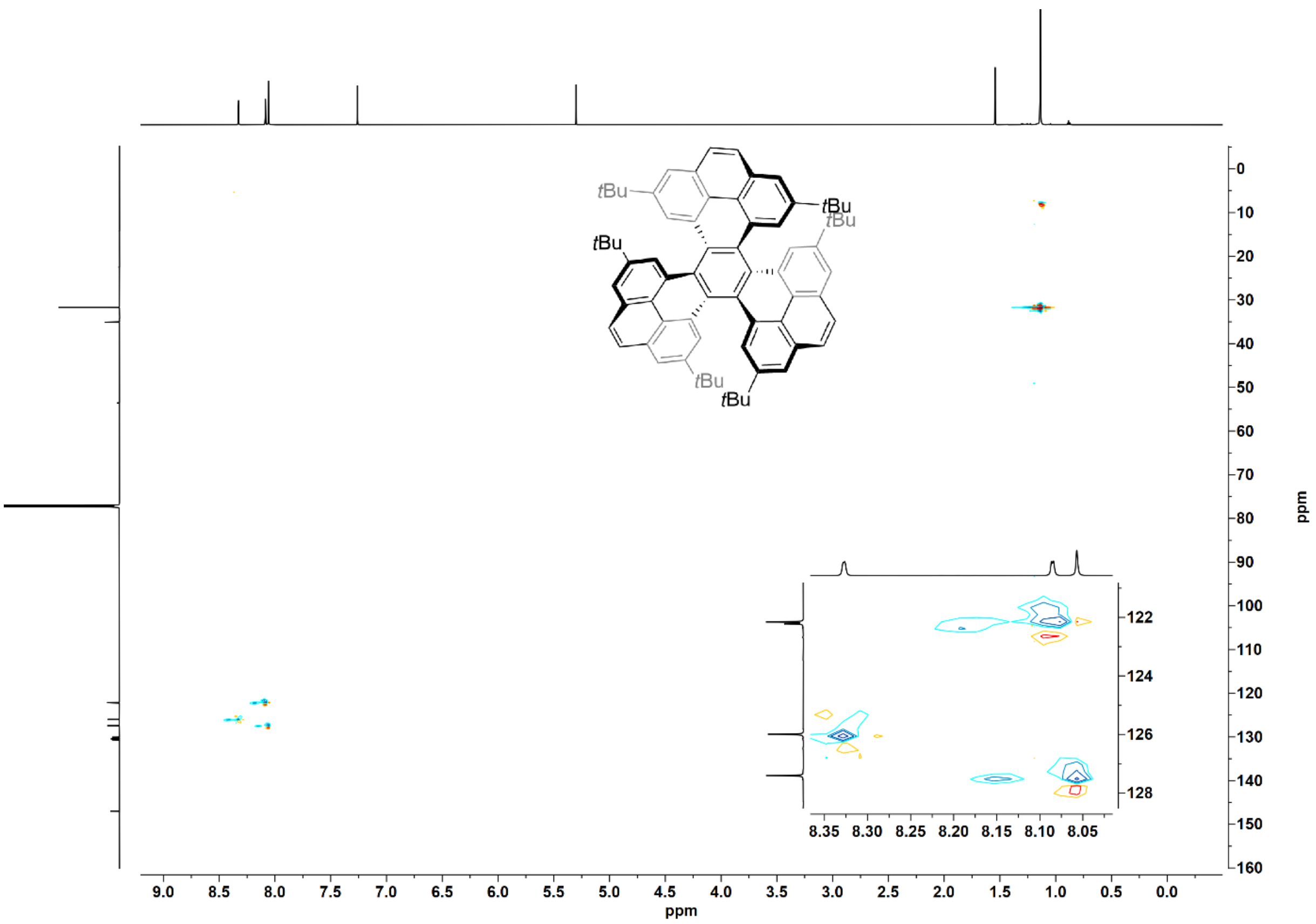

Figure S5: ^{1}H-^{13}C HSQC NMR spectrum ($CDCl_3$, 700 / 176 MHz, r.t.) of ***rac-D*$_3$-2**.

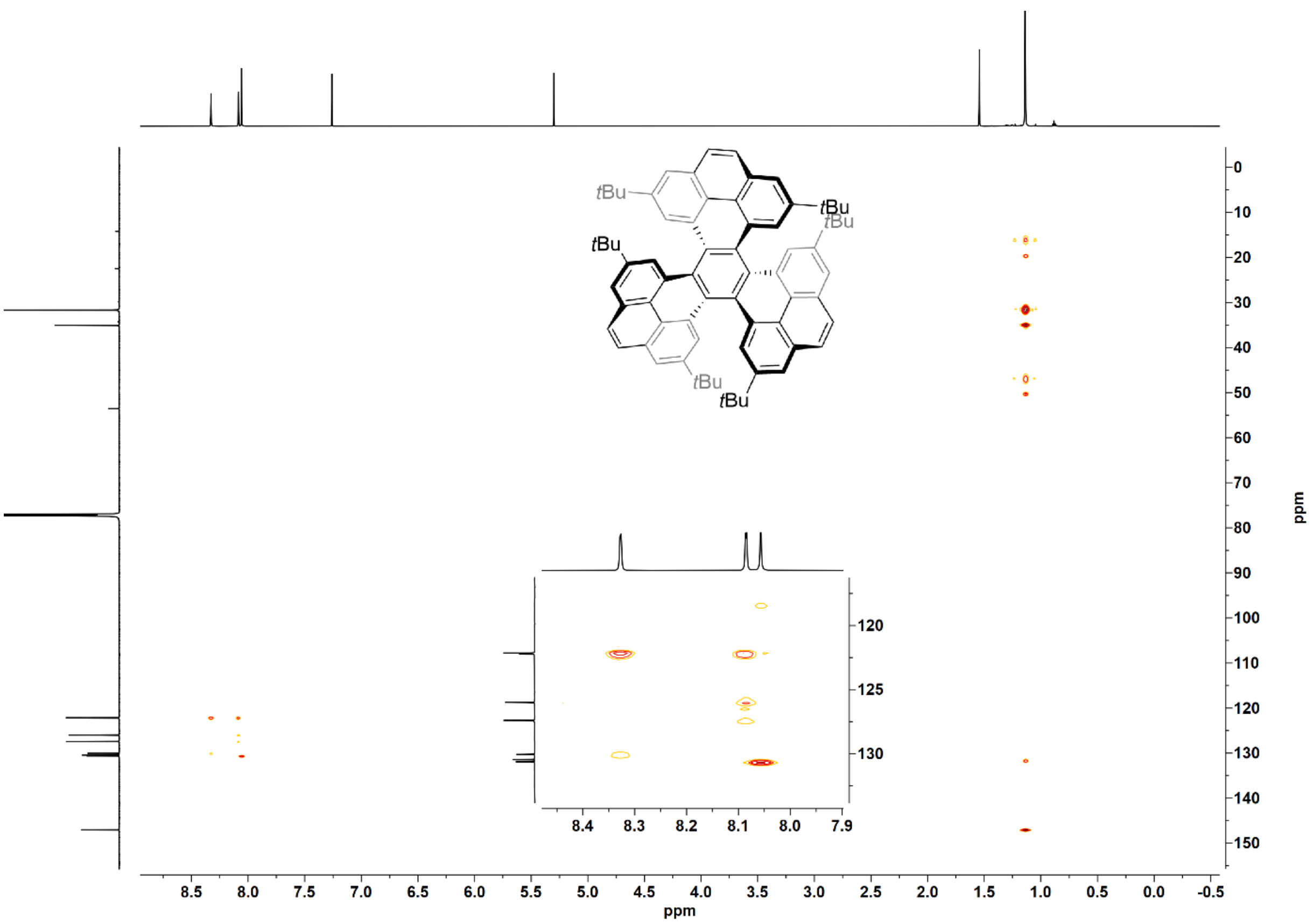

Figure S6: ^{1}H-^{13}C HMBC NMR spectrum ($CDCl_3$, 700 / 176 MHz, r.t.) of ***rac-D*$_3$-2**.

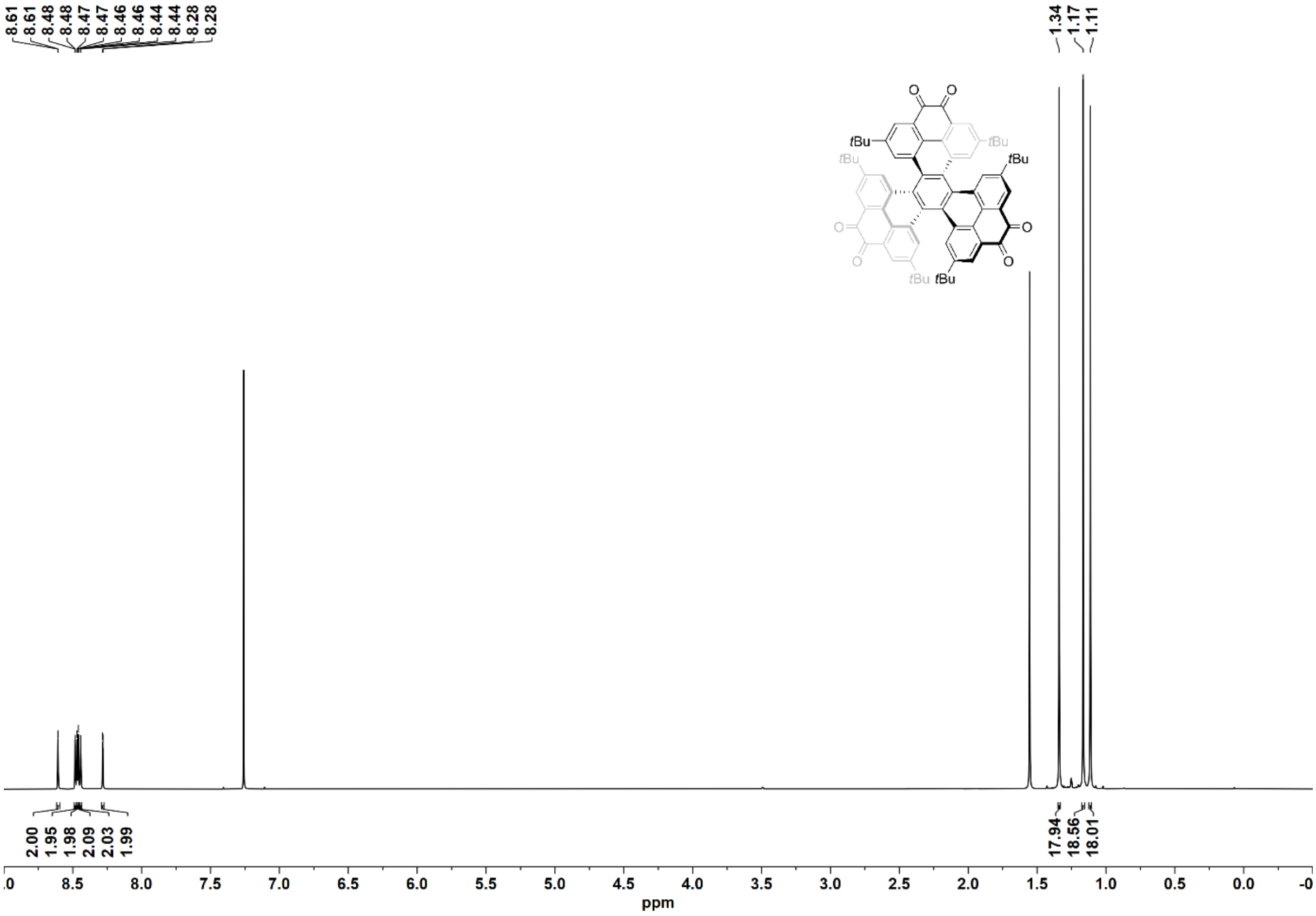

Figure S7: ^{1}H NMR spectrum ($CDCl_3$, 700 MHz, r.t.) of ***rac*-*C*$_2$-3**.

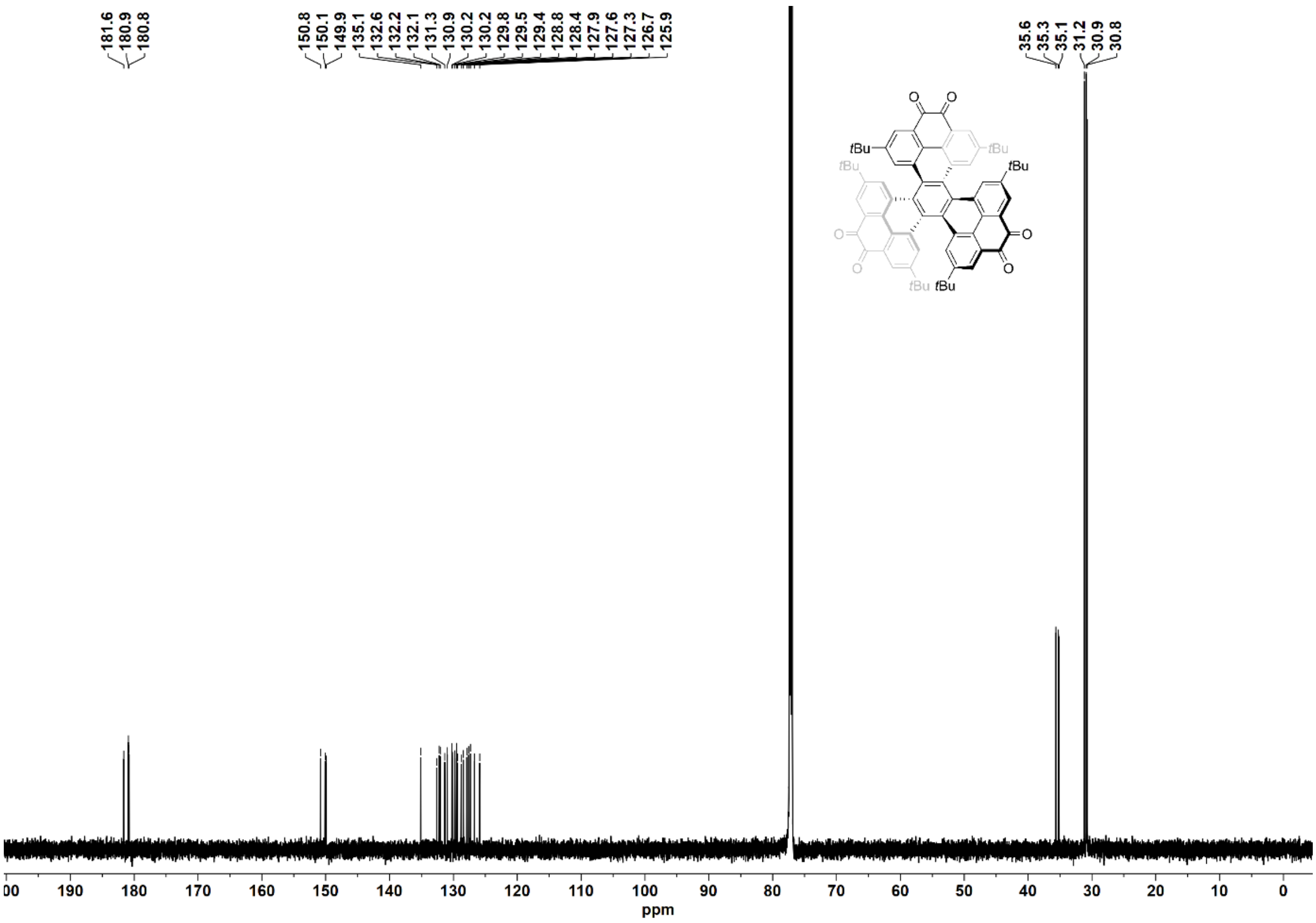

Figure S8: ^{13}C NMR spectrum ($CDCl_3$, 176 MHz, r.t.) of ***rac*-*C*$_2$-3**.

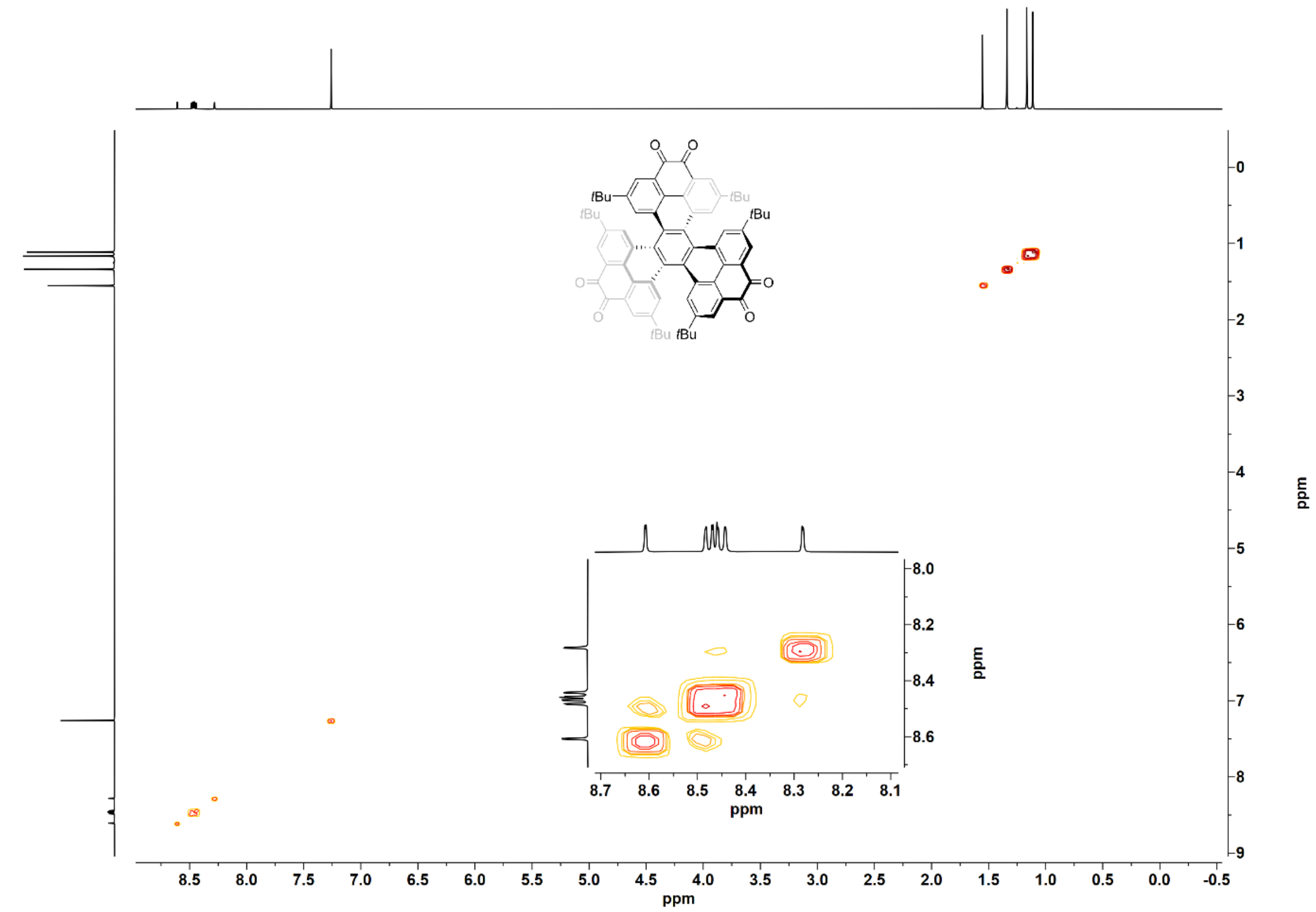

Figure S9: ^{1}H-^{1}H COSY NMR spectrum ($CDCl_3$, 700 / 700 MHz, r.t.) of ***rac*-*C*$_2$-3**.

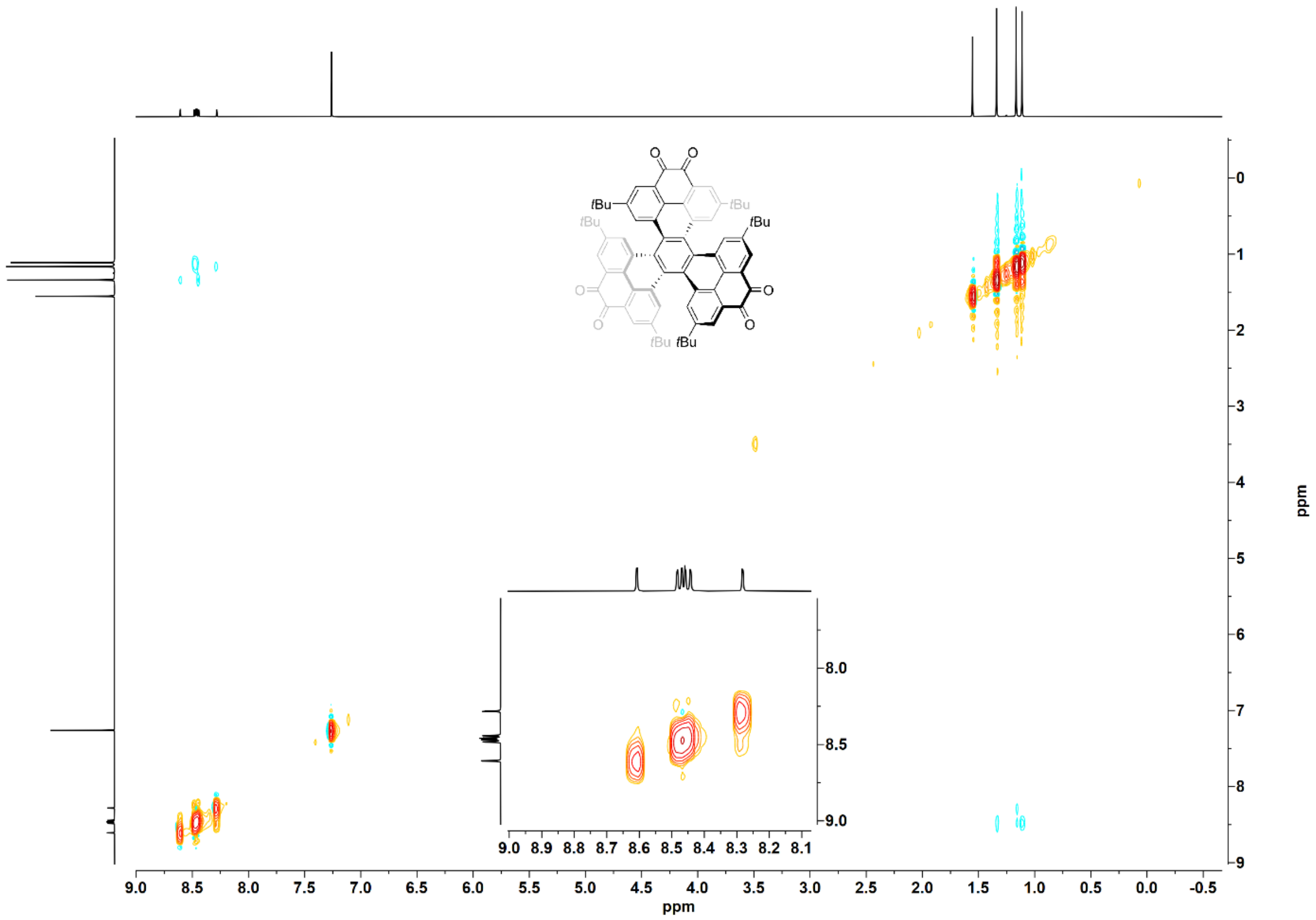

Figure S10: ^{1}H-^{1}H NOESY NMR spectrum ($CDCl_3$, 700 / 700 MHz, r.t.) of ***rac*-*C*$_2$-3**.

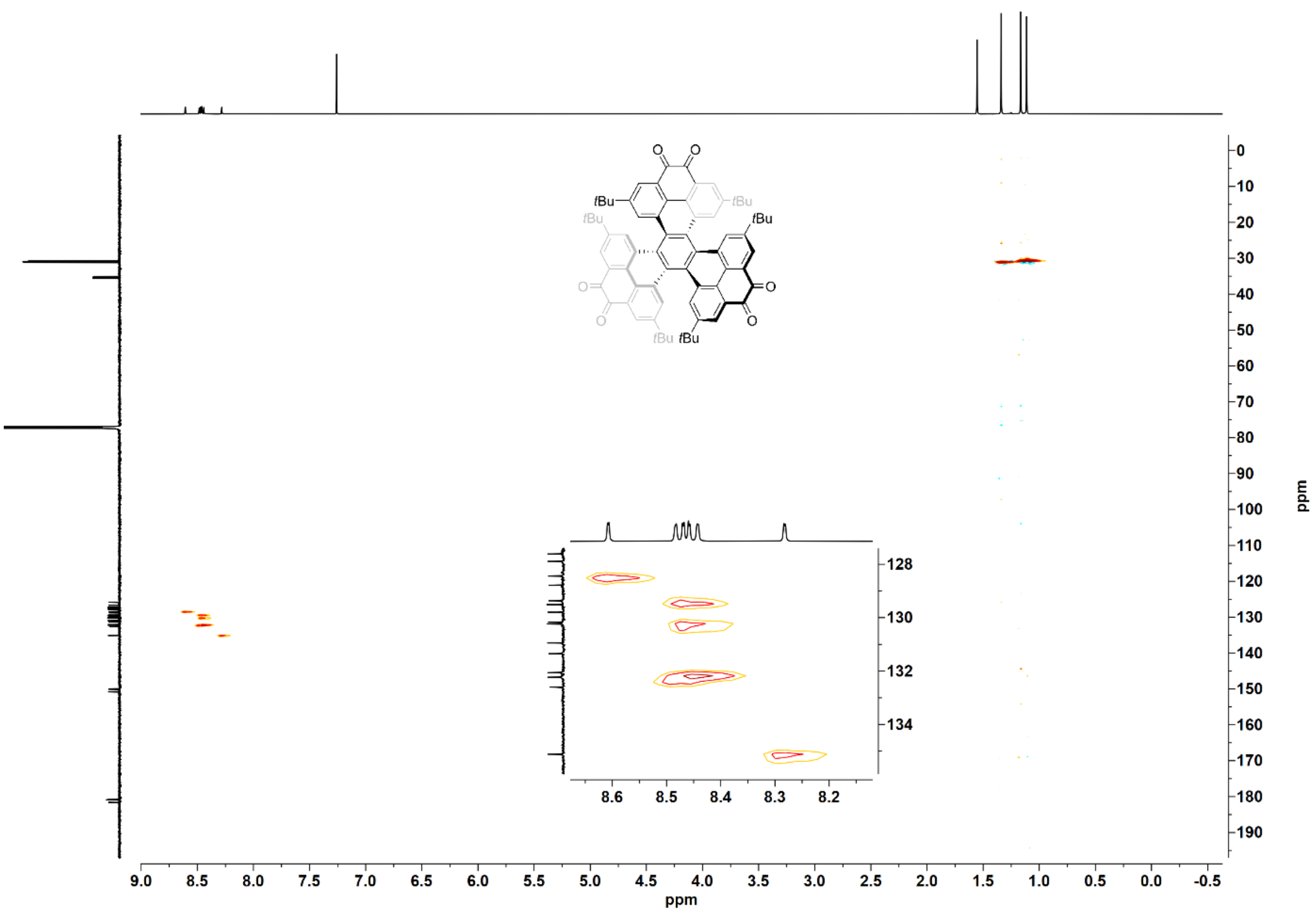

Figure S11: ^{1}H-^{13}C HSQC NMR spectrum ($CDCl_3$, 700 / 176 MHz, r.t.) of ***rac*-*C*$_2$-3**.

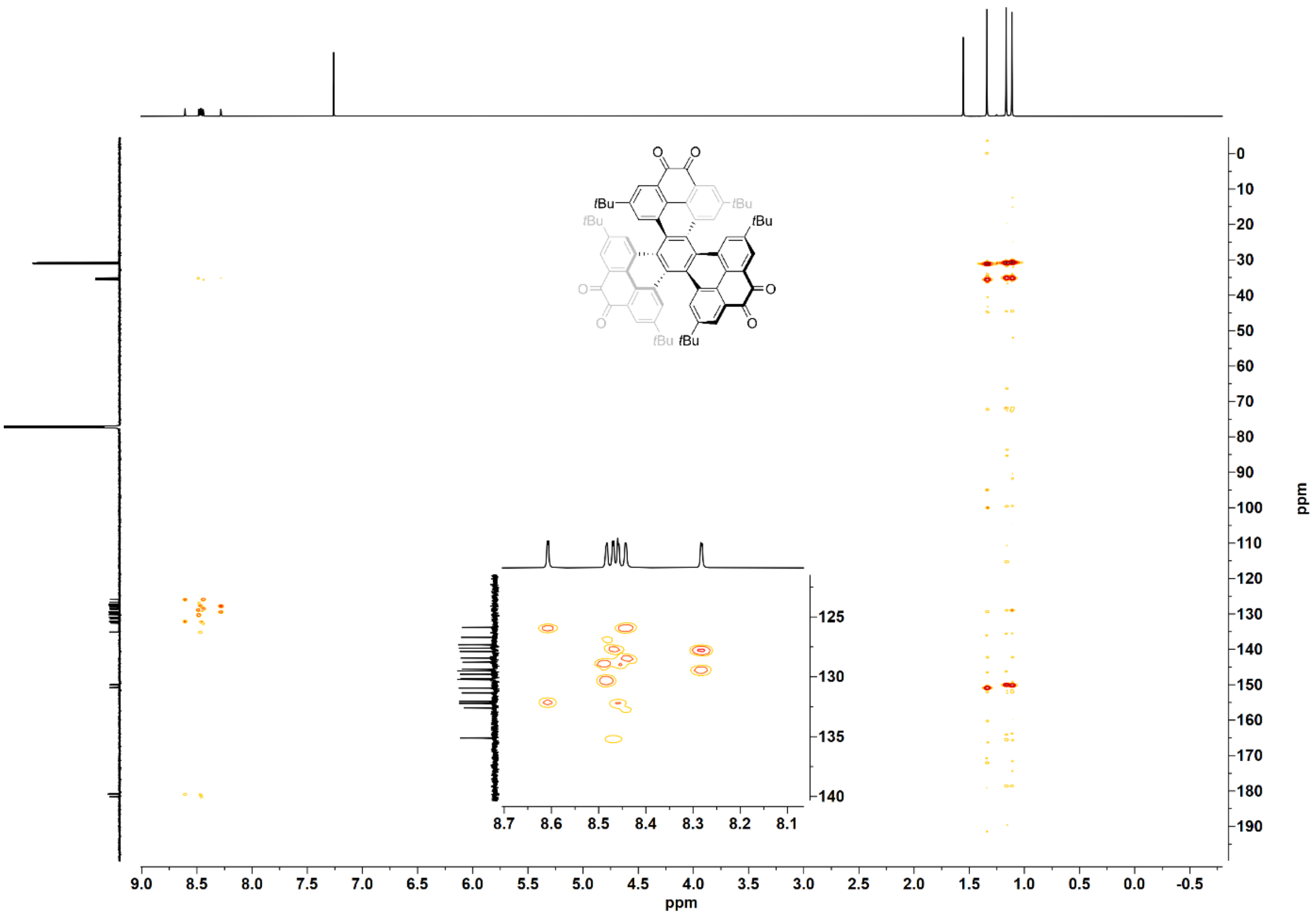

Figure S12: ^{1}H-^{13}C HMBC NMR spectrum ($CDCl_3$, 700 / 176 MHz, r.t.) of ***rac*-*C*$_2$-3**.

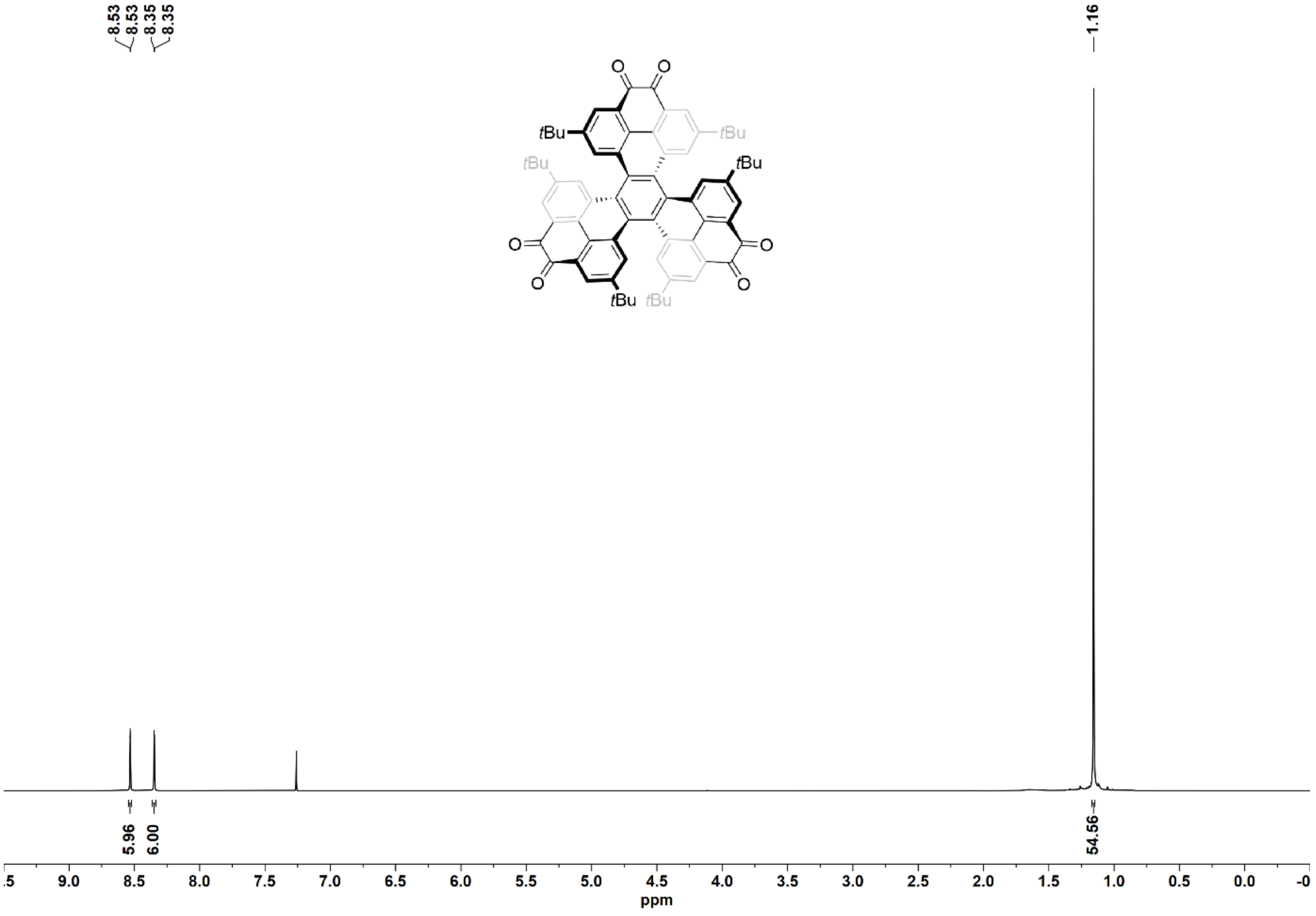

Figure S13: ^{1}H NMR spectrum ($CDCl_3$, 600 MHz, r.t.) of ***rac-D*$_3$-3**.

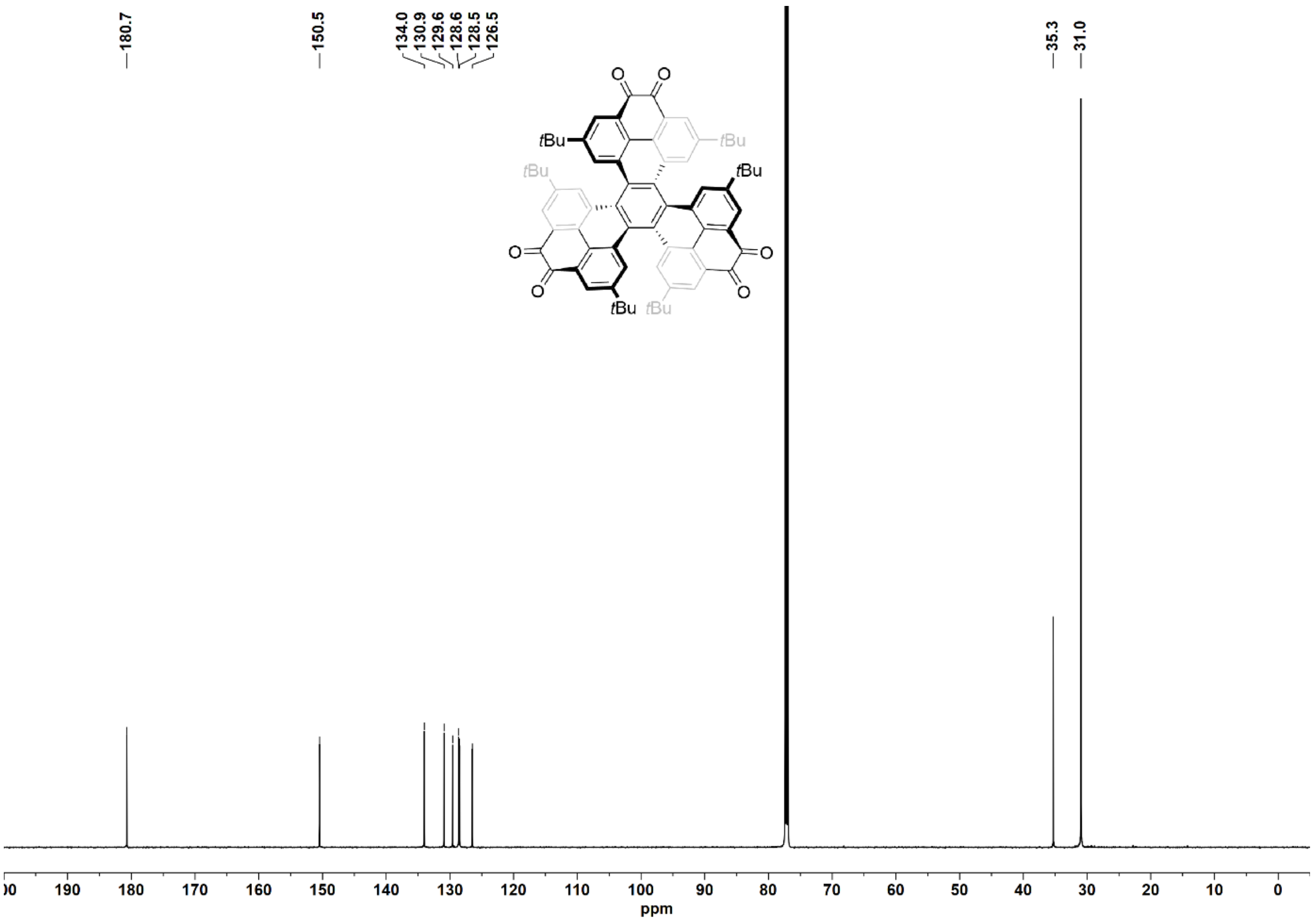

Figure S14: ^{13}C NMR spectrum ($CDCl_3$, 151 MHz, r.t.) of ***rac-D*$_3$-3**.

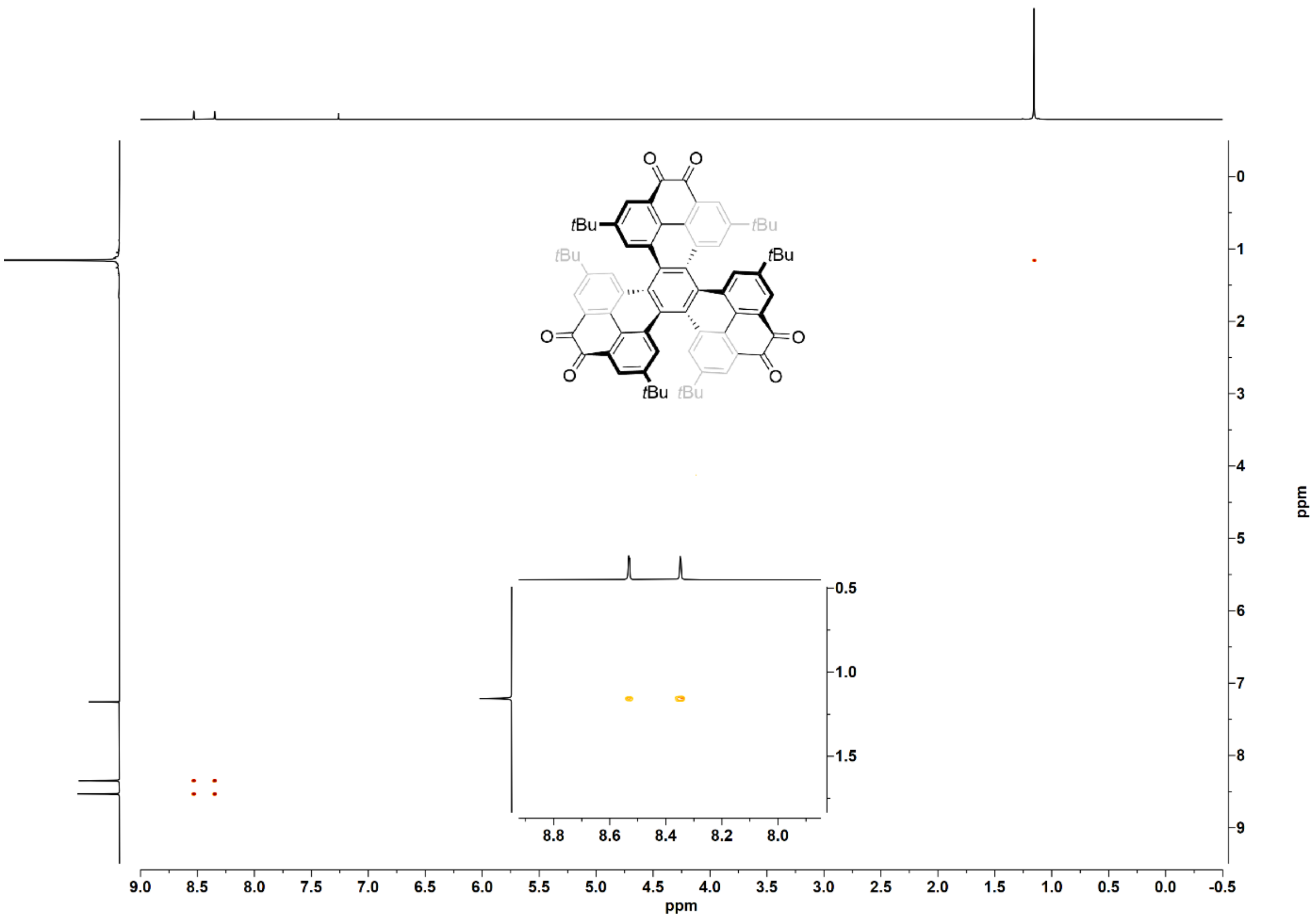

Figure S15: ^{1}H-^{1}H COSY NMR spectrum ($CDCl_3$, 600 / 600 MHz, r.t.) of ***rac-D$_3$-3***.

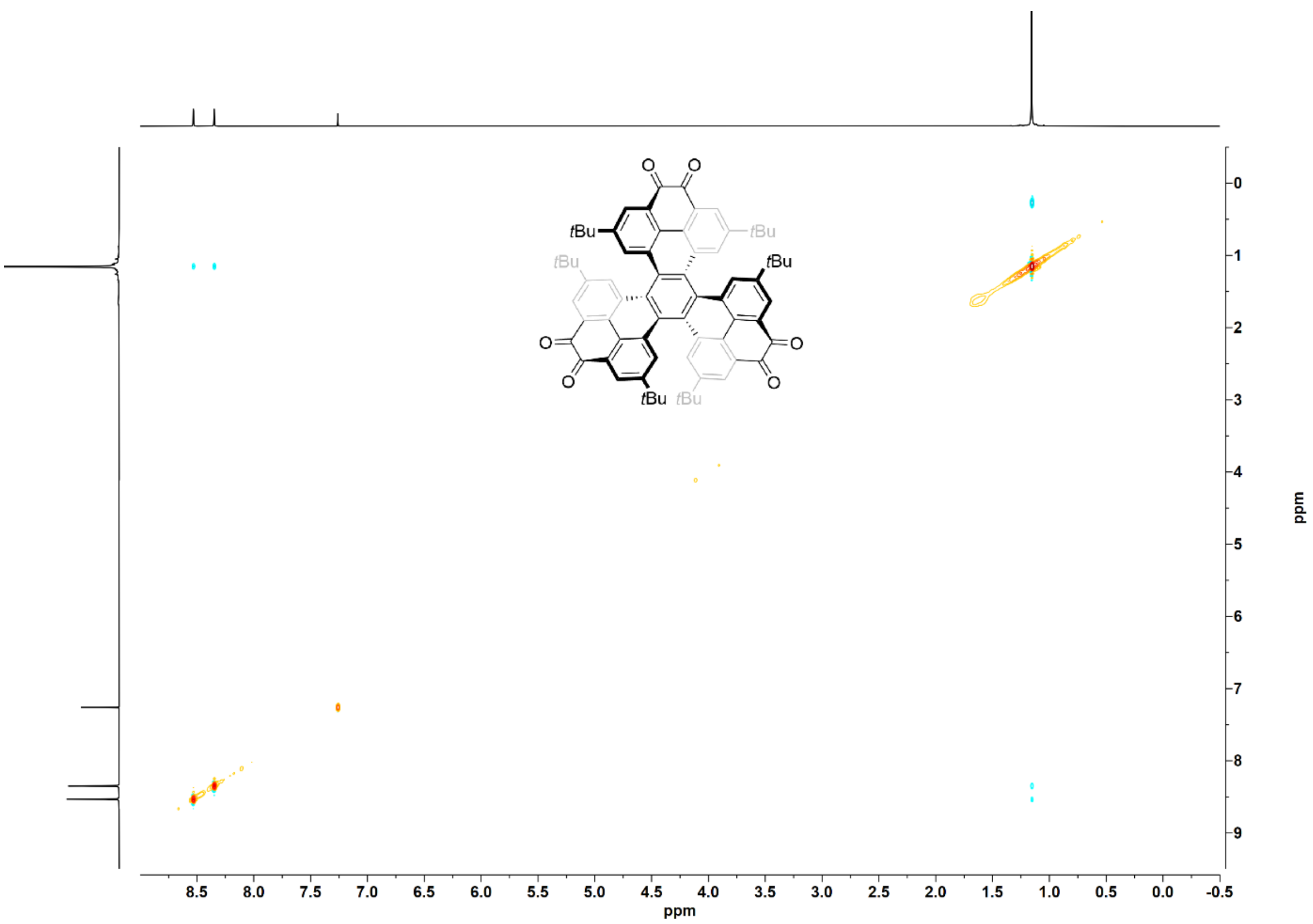

Figure S16: ^{1}H-^{1}H NOESY NMR spectrum ($CDCl_3$, 600 / 600 MHz, r.t.) of ***rac-D$_3$-3***.

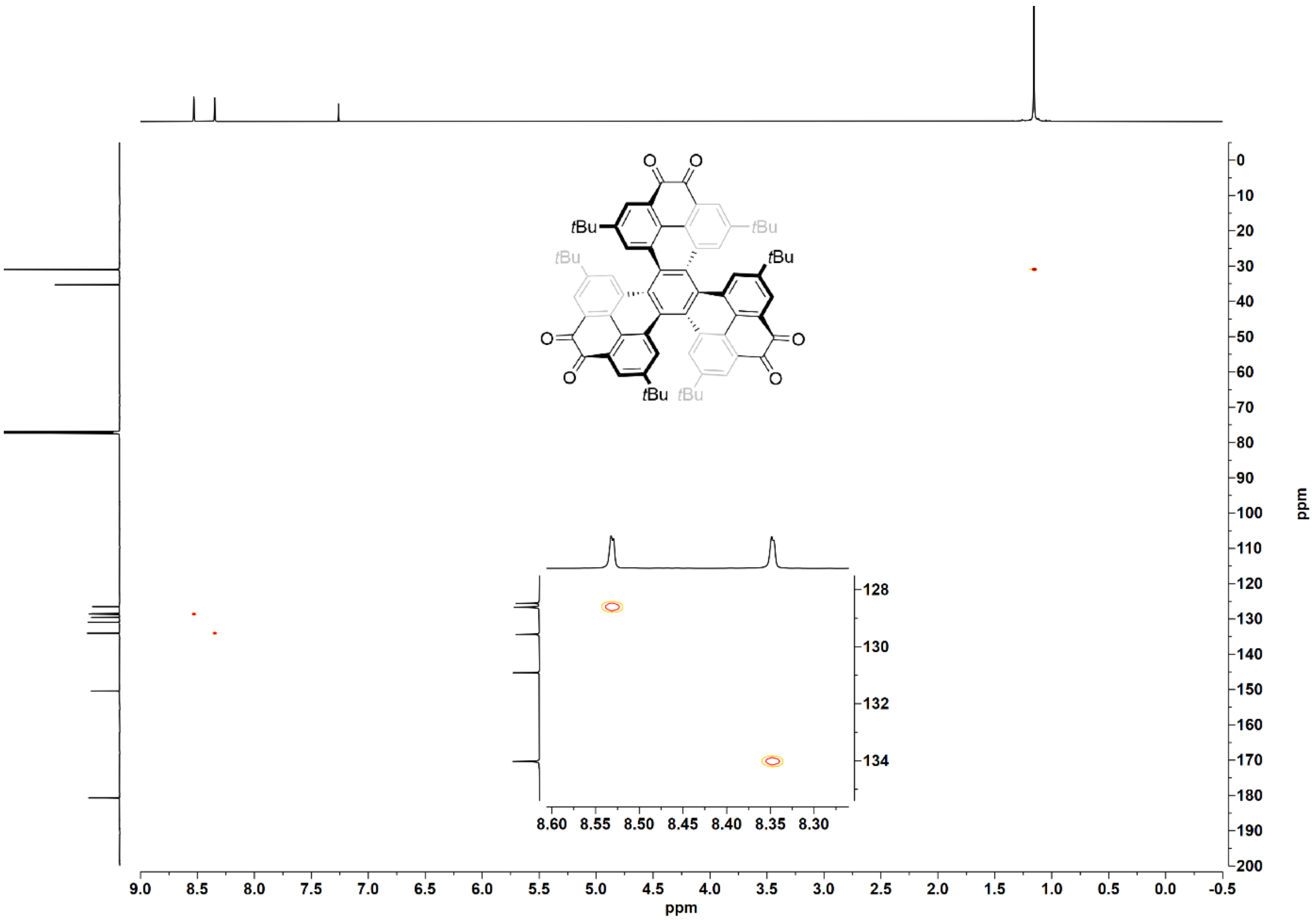

Figure S17: ^{1}H-^{13}C HSQC NMR spectrum ($CDCl_3$, 600 / 151 MHz, r.t.) of ***rac*-*D*$_3$-3**.

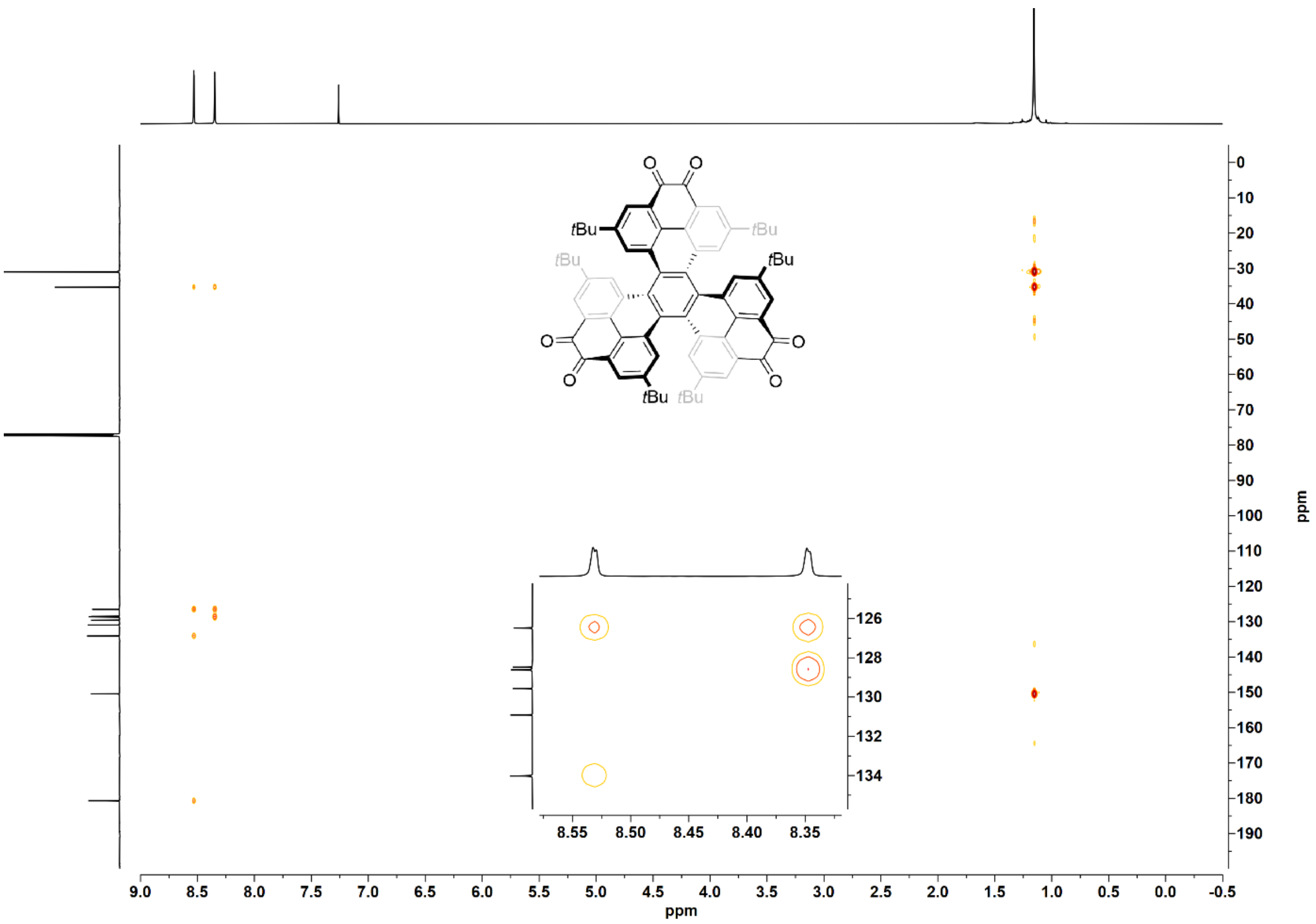

Figure S18: ^{1}H-^{13}C HMBC NMR spectrum ($CDCl_3$, 600 / 151 MHz, r.t.) of ***rac*-*D*$_3$-3**.

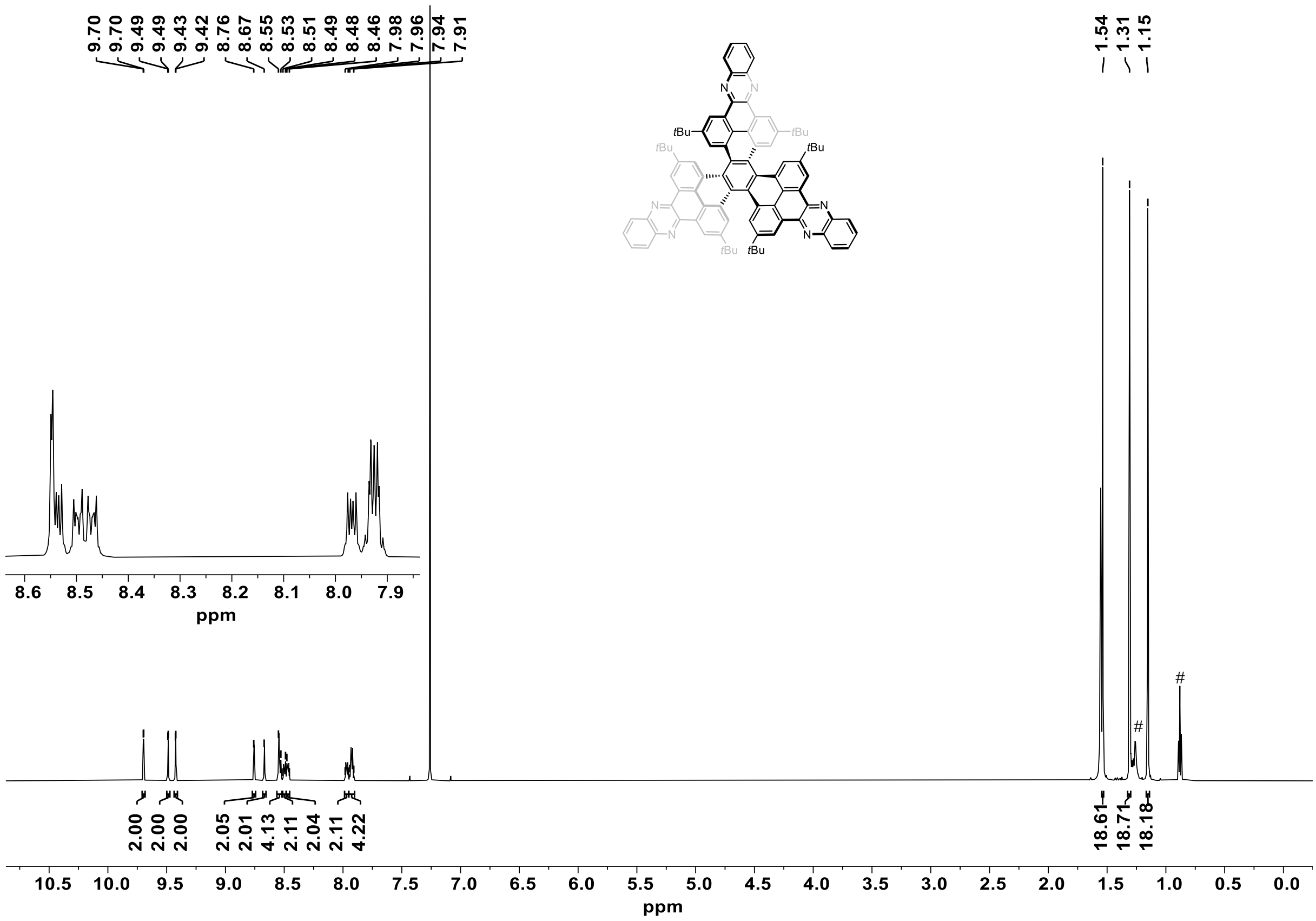

Figure S19: ^{1}H NMR spectrum ($CDCl_3$, 600 MHz, r.t.) of ***rac*-*C*₂-4**. #: *n*-heptane.

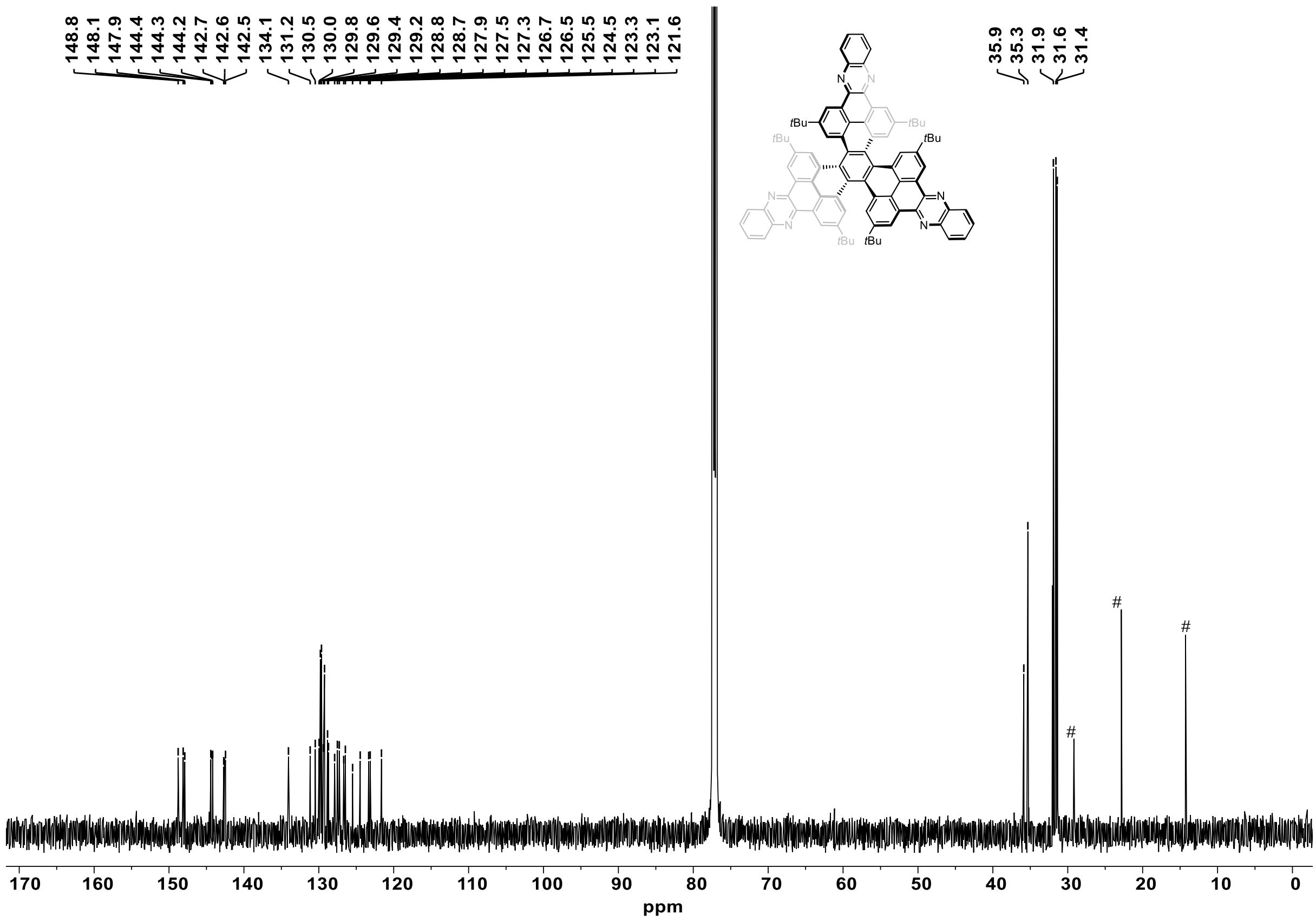

Figure S20: ^{13}C NMR spectrum ($CDCl_3$, 151 MHz, r.t.) of ***rac*-*C*₂-4**. #: *n*-heptane.

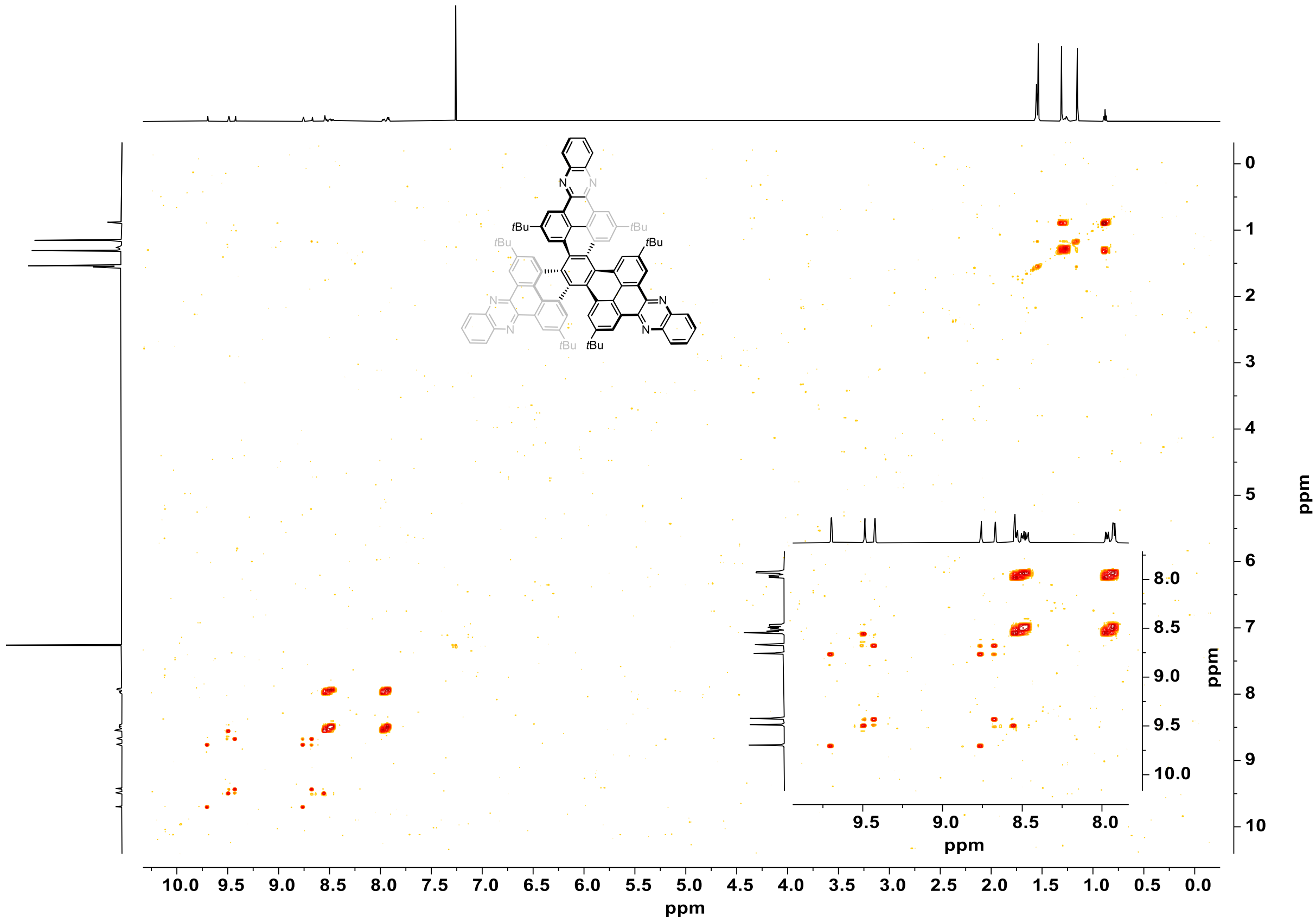

Figure S21: ^{1}H-^{1}H COSY NMR spectrum ($CDCl_3$, 600 / 600 MHz, r.t.) of ***rac-C*₂-4**.

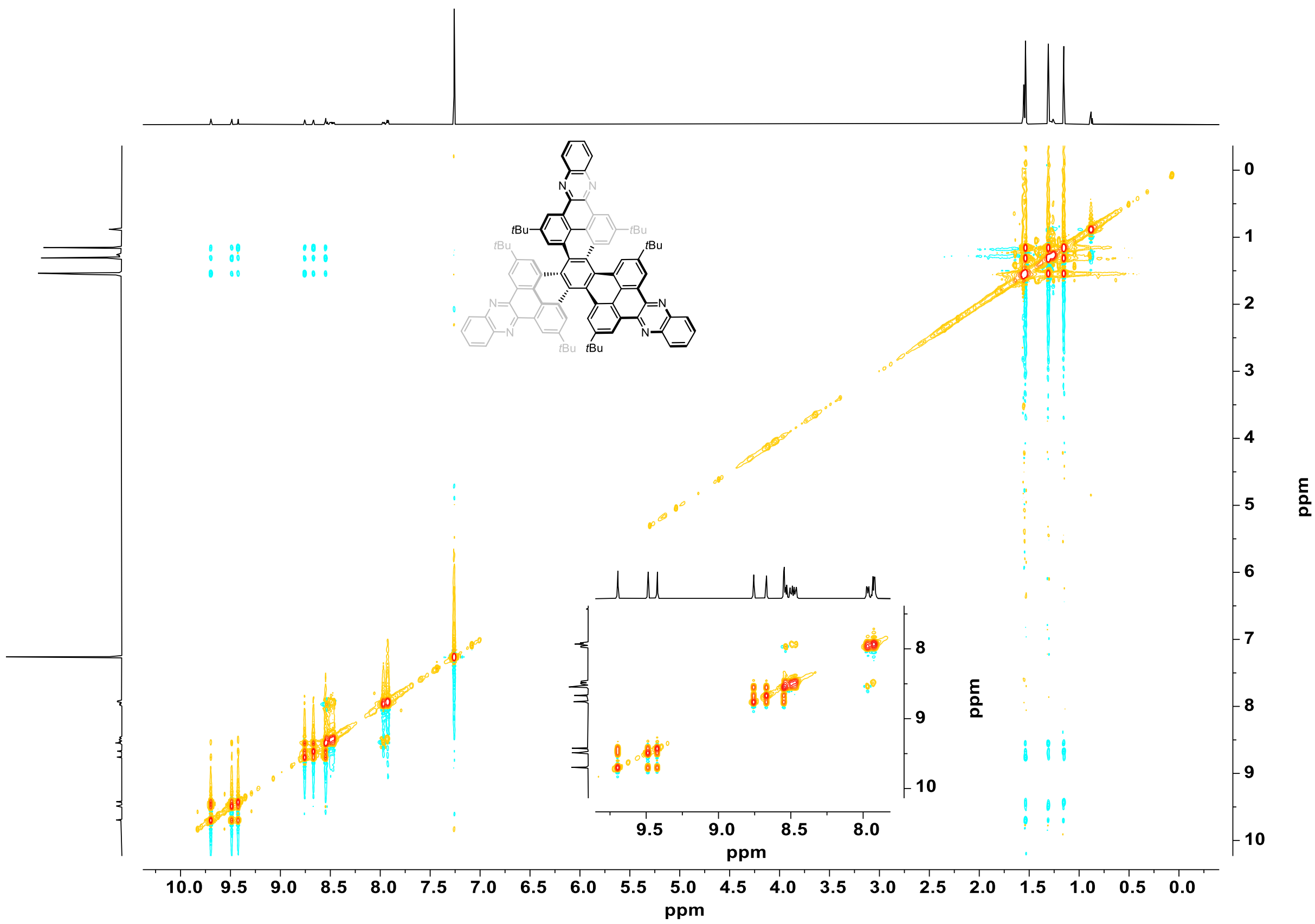

Figure S22: ^{1}H-^{1}H NOESY NMR spectrum ($CDCl_3$, 600 / 151 MHz, r.t.) of ***rac-C*₂-4**.

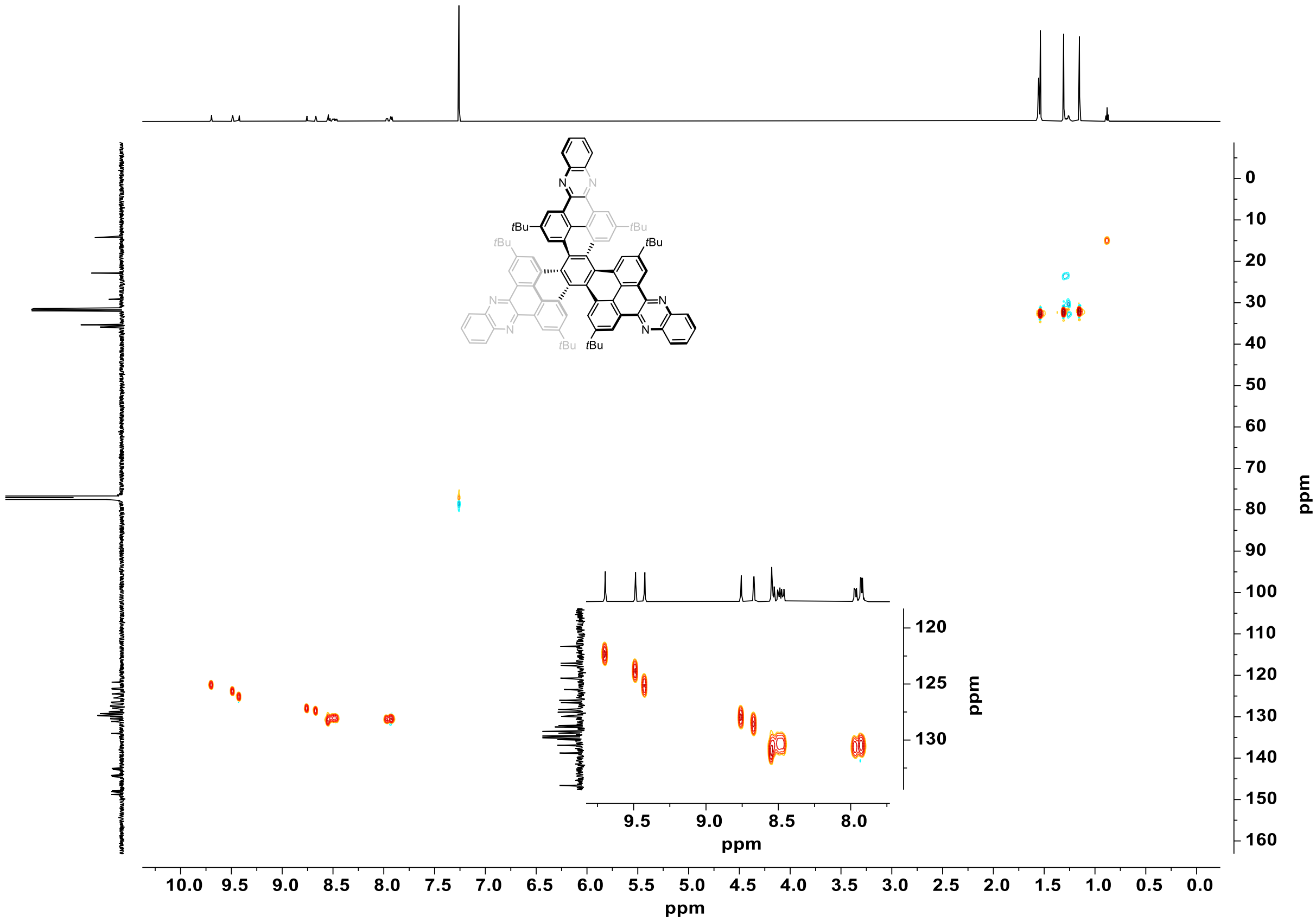

Figure S23: ^{1}H-^{13}C HSQC NMR spectrum ($CDCl_3$, 600 / 151 MHz, r.t.) of ***rac*-*C*$_2$-4**.

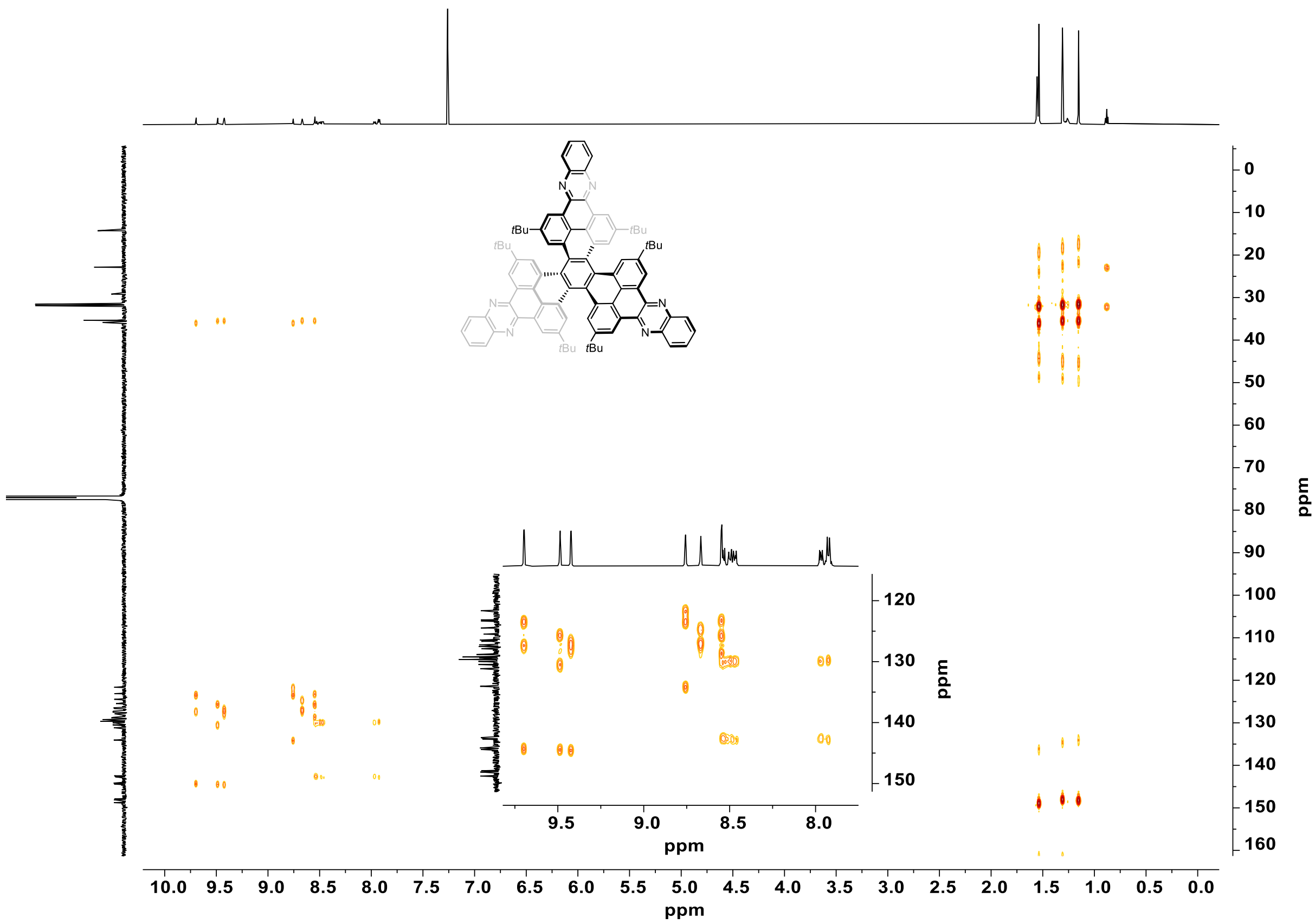

Figure S24: ^{1}H-^{13}C HMBC NMR spectrum ($CDCl_3$, 600 / 151 MHz, r.t.) of ***rac*-*C*$_2$-4**.

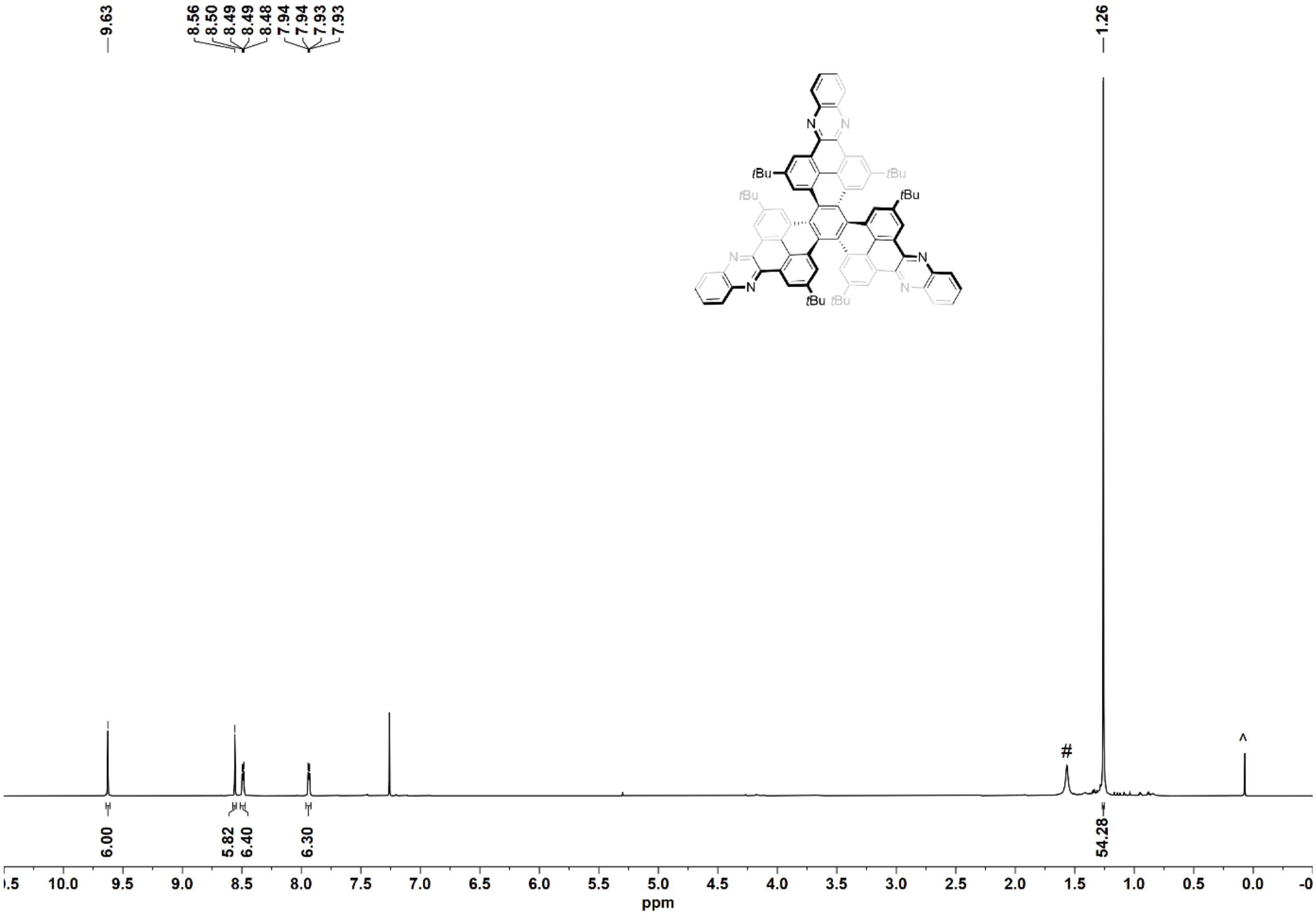

Figure S25: ^{1}H NMR spectrum ($CDCl_3$, 700 MHz, r.t.) of ***rac-D*₃-4**. #: water. ^: silicon grease.

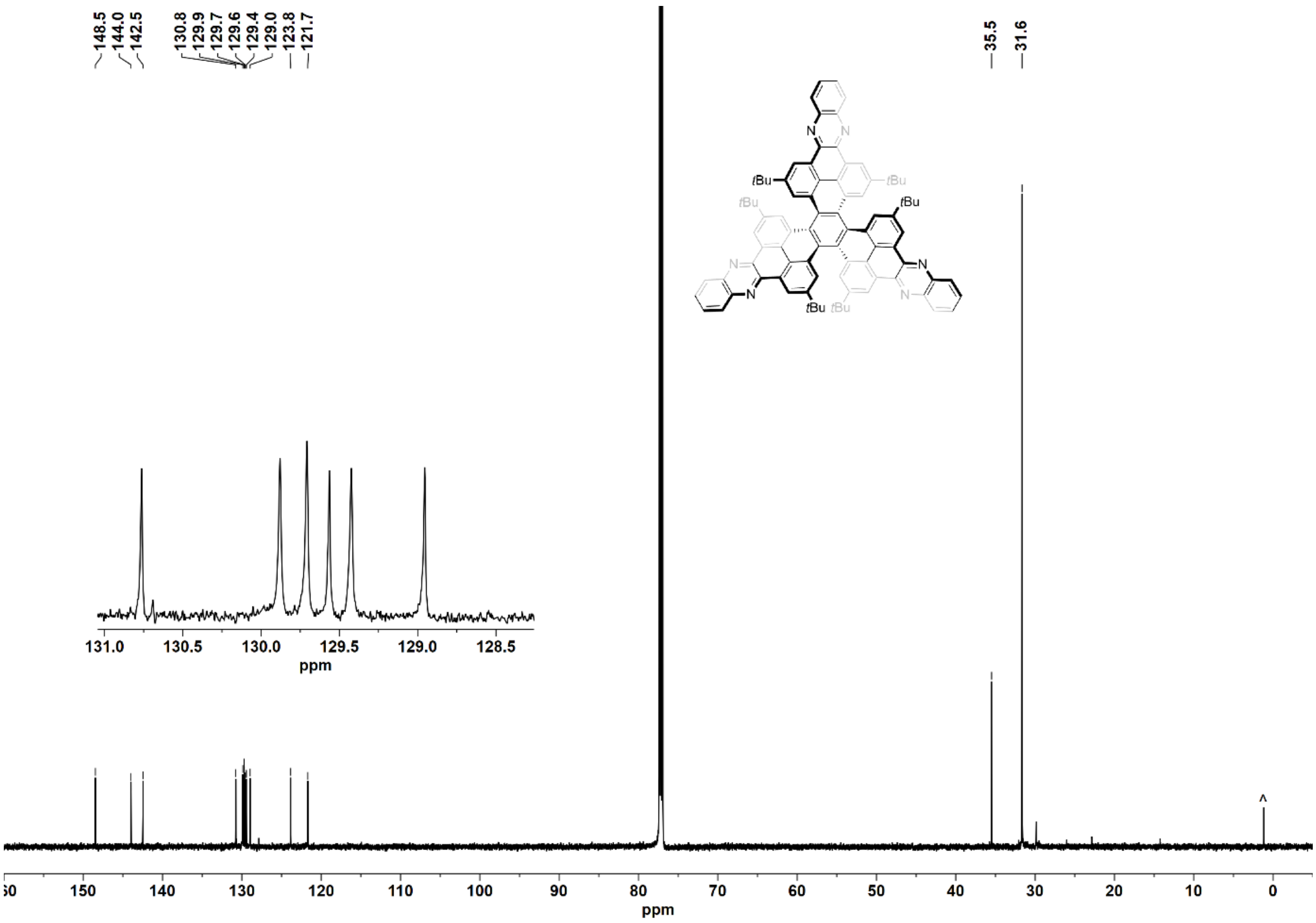

Figure S26: ^{13}C NMR spectrum ($CDCl_3$, 176 MHz, r.t.) of ***rac-D*₃-4**. ^: silicon grease.

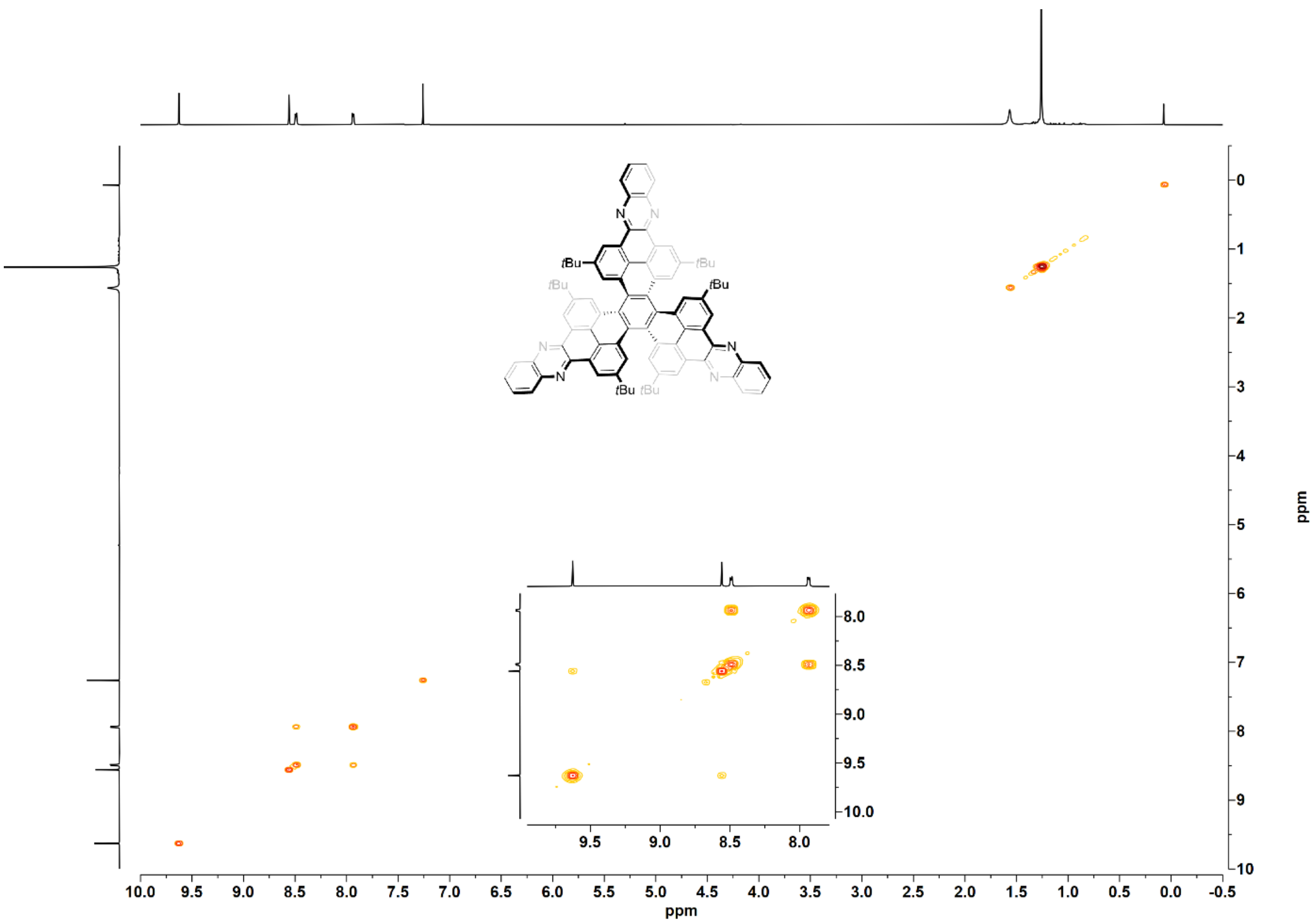

Figure S27: ^{1}H-^{1}H COSY NMR spectrum ($CDCl_3$, 700 / 700 MHz, r.t.) of ***rac-D*$_3$-4**.

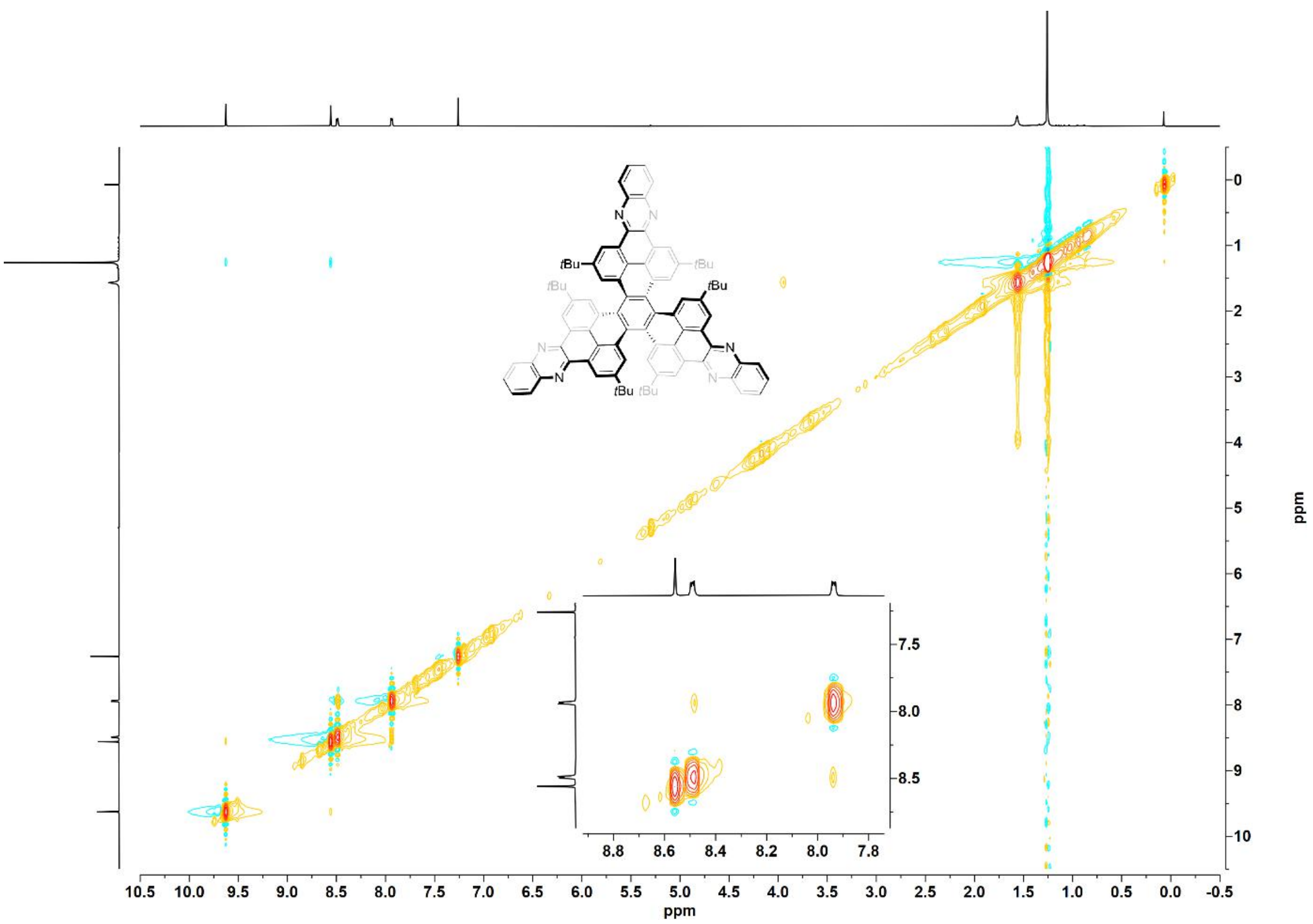

Figure S28: ^{1}H-^{1}H NOESY NMR spectrum ($CDCl_3$, 700 / 700 MHz, r.t.) of ***rac-D*$_3$-4**.

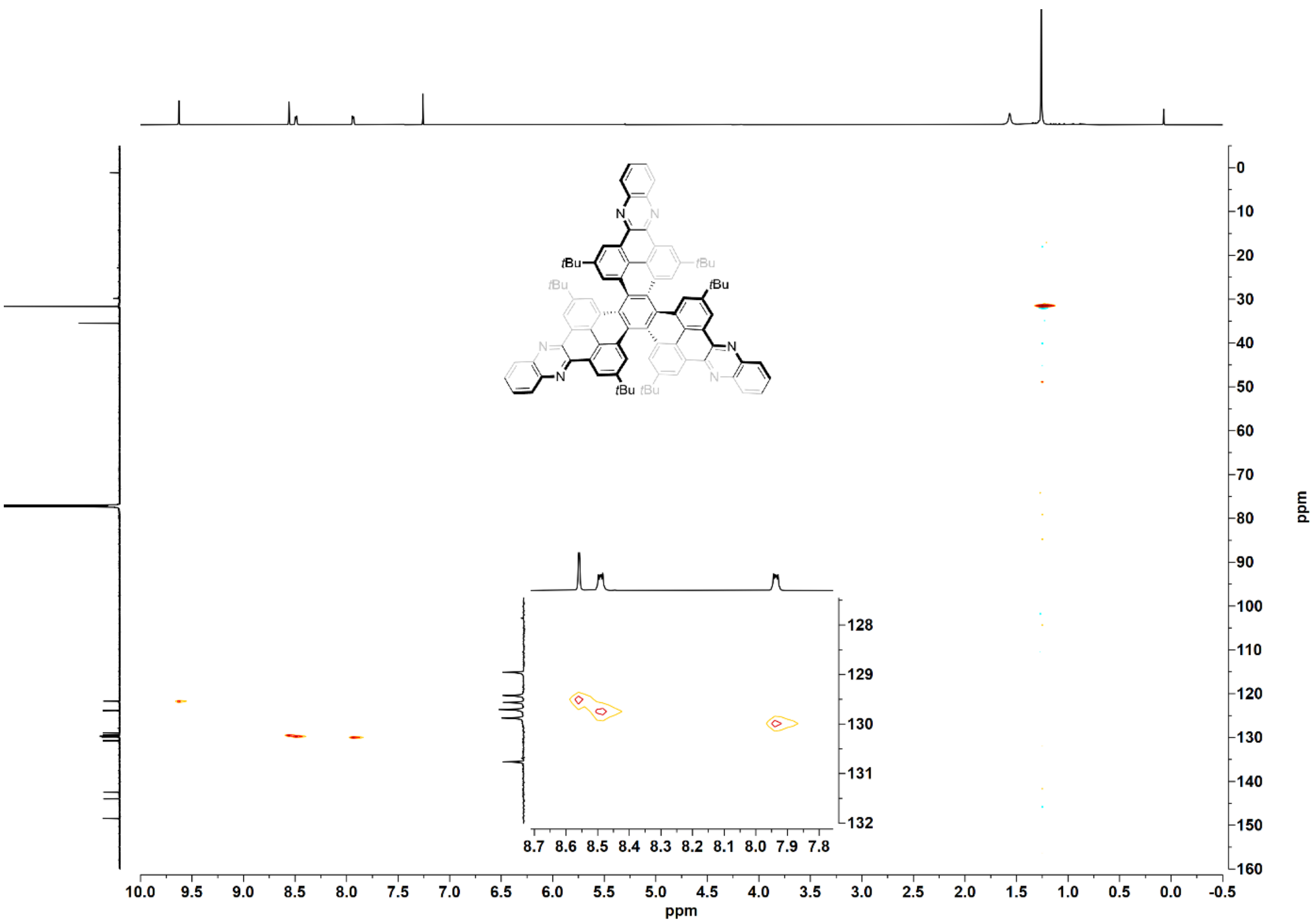

Figure S29: ^{1}H-^{13}C HSQC NMR spectrum ($CDCl_3$, 700 / 176 MHz, r.t.) of ***rac-D*₃-4**.

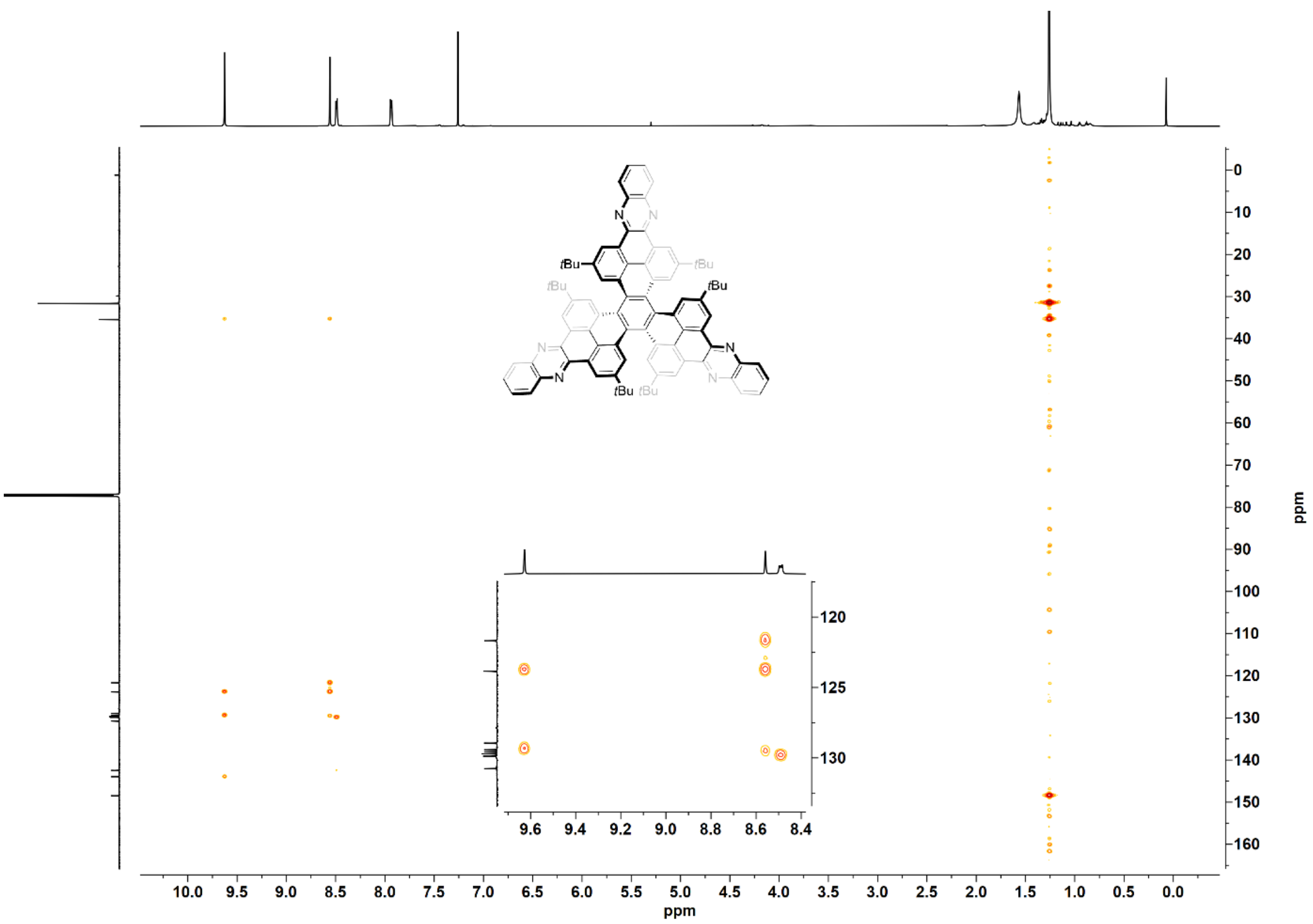

Figure S30: ^{1}H-^{13}C HMBC NMR spectrum ($CDCl_3$, 700 / 176 MHz, r.t.) of ***rac-D*₃-4**.

3.2 VT NMR spectra

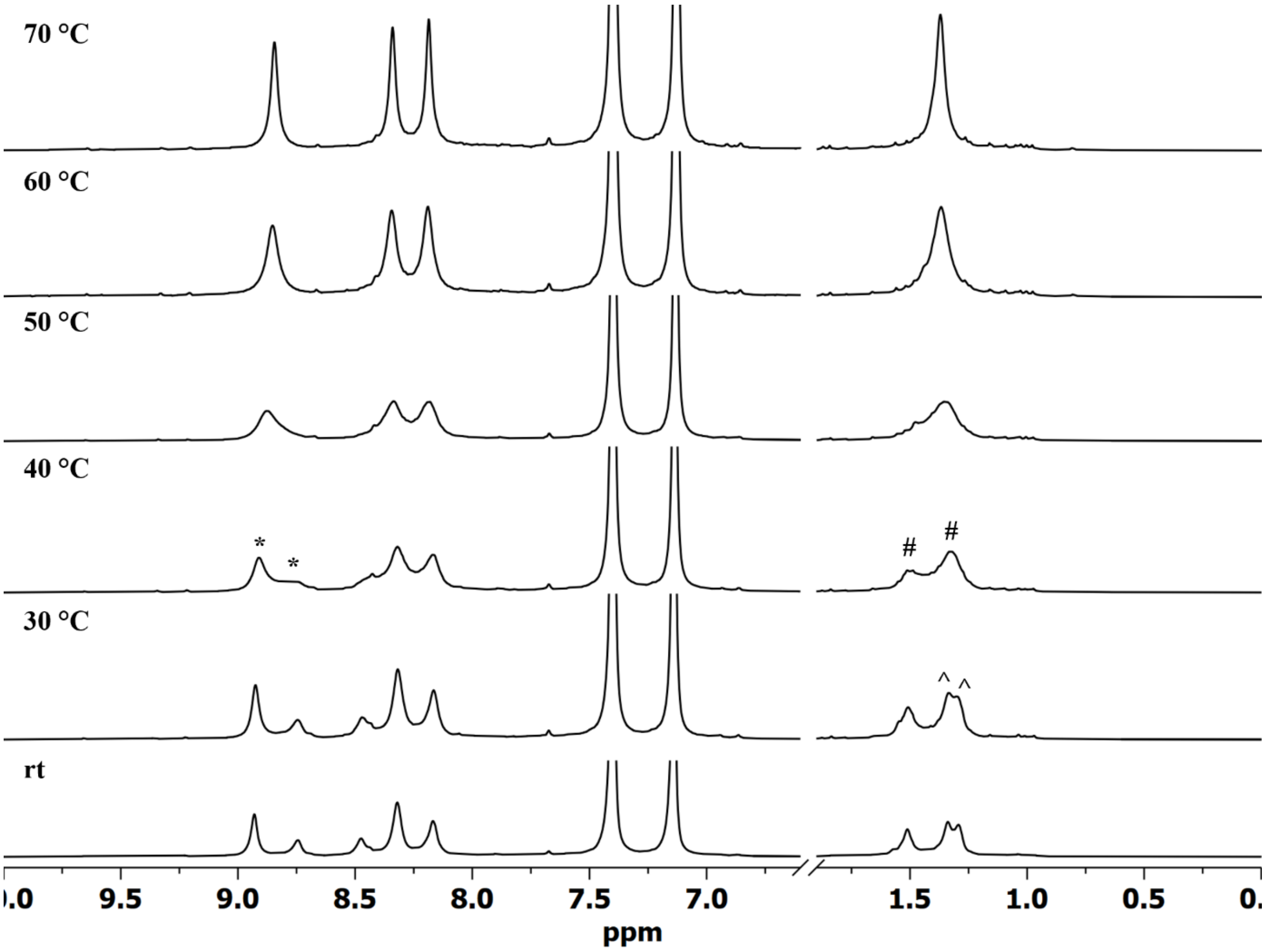

Figure S31: ^{1}H-NMR spectra (o-DCB, 300 MHz) of ***C*₂-2** at various temperatures. Signals marked with *, # or ^ were used in the calculation of the racemization barrier. Note: intensities of peaks in aliphatic and aromatic regions are different, but depicted at similar heights for simplicity reasons.

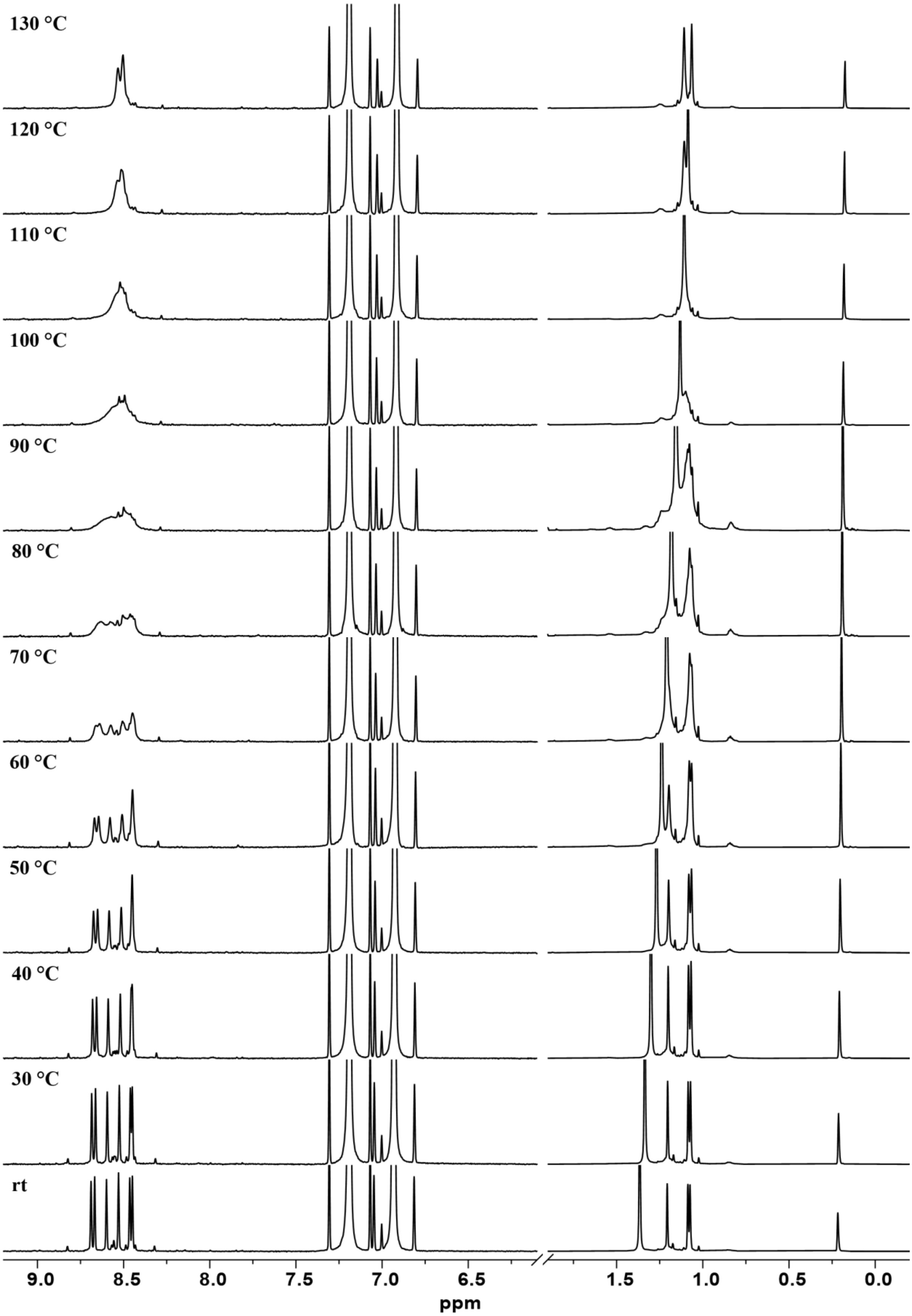

Figure S32: ^{1}H-NMR spectra (*o*-DCB, 300 MHz) of ***C***$_2$**-3** at various temperatures. Note: intensities of peaks in aliphatic and aromatic regions are different, but depicted at similar heights for simplicity reasons.

4. IR Spectra

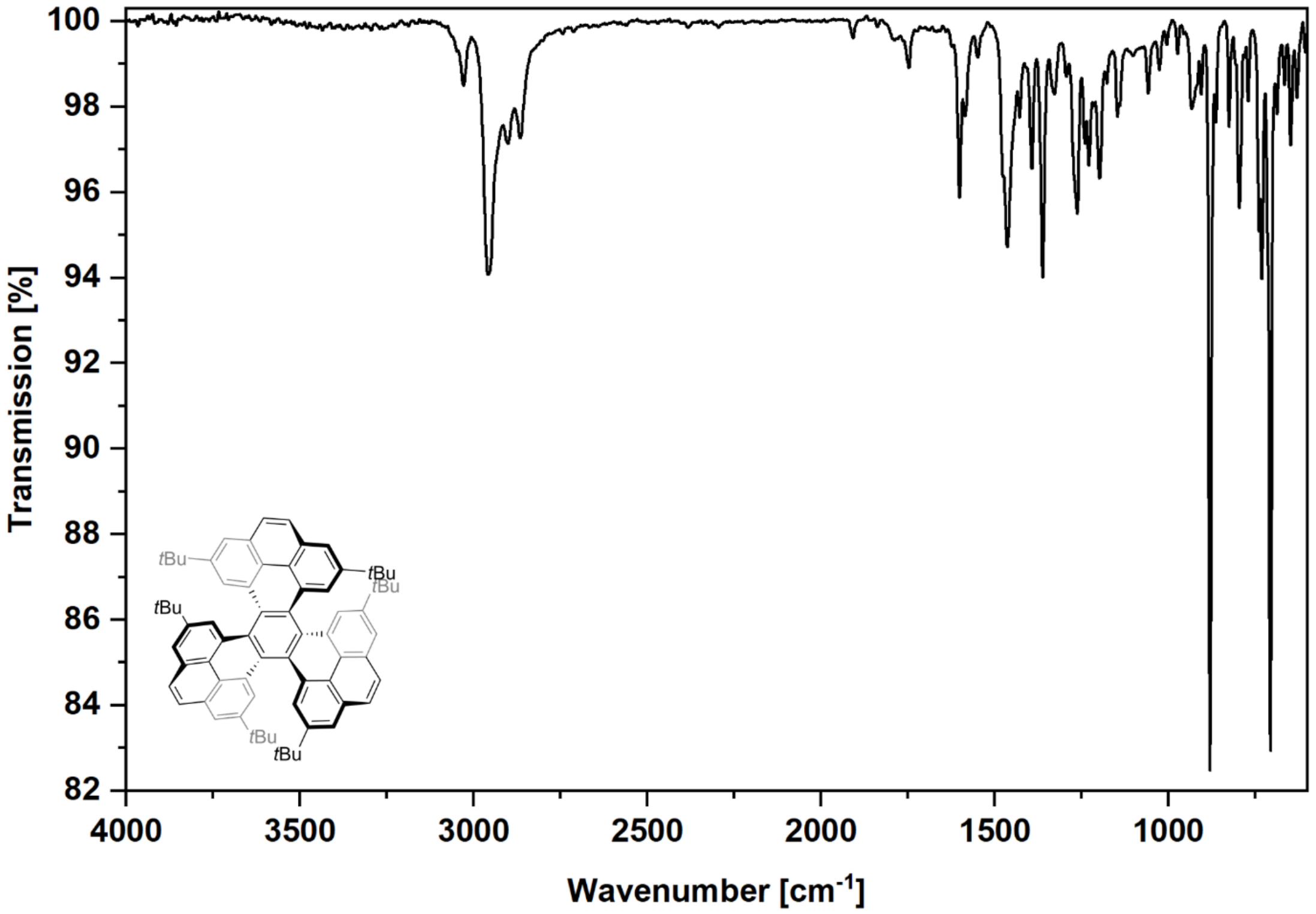

Figure S33: IR-spectrum (ATR, r.t.) of ***rac*-*D*$_3$-2**.

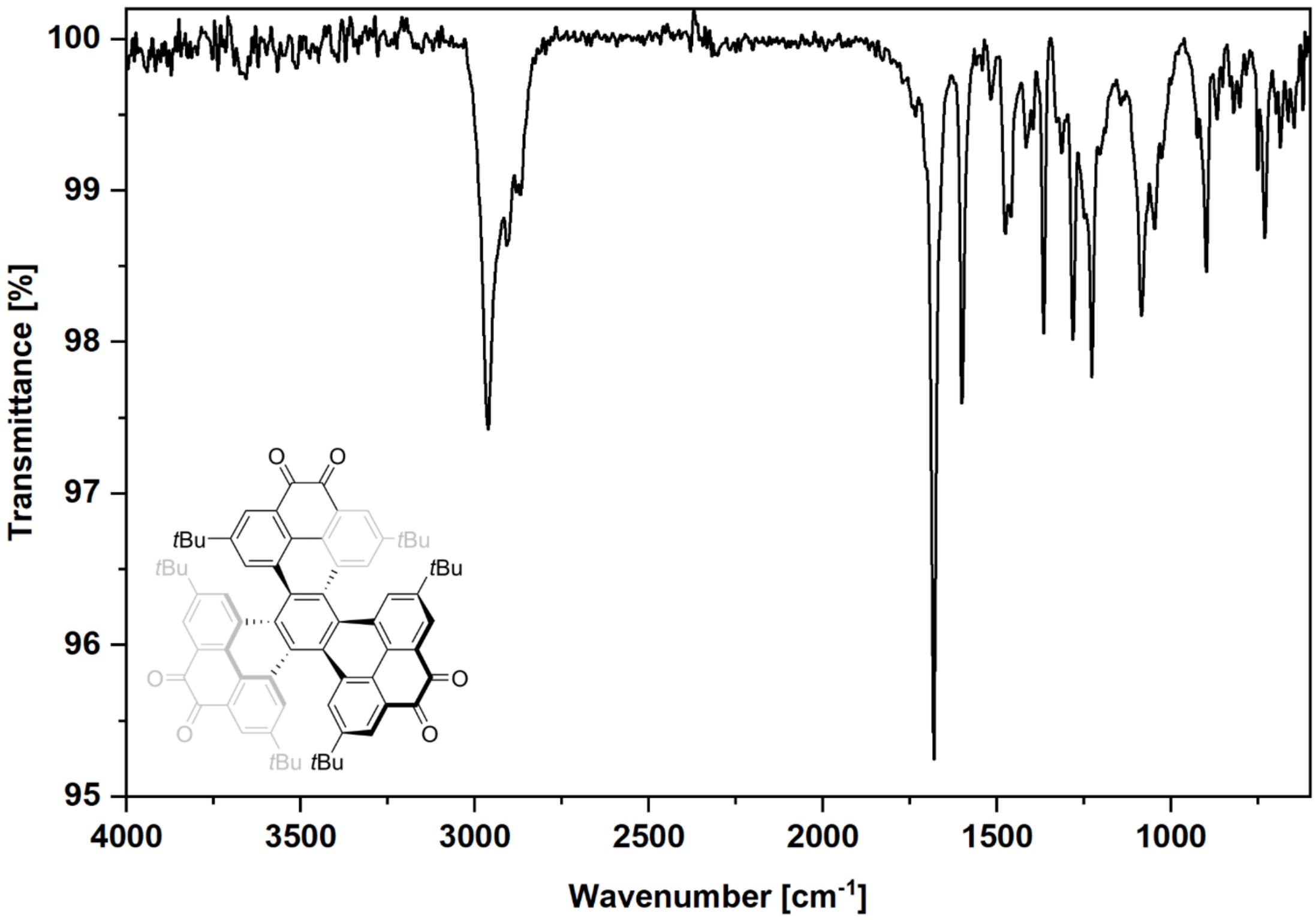

Figure S34: IR-spectrum (ATR, r.t.) of ***rac*-*C*$_2$-3**.

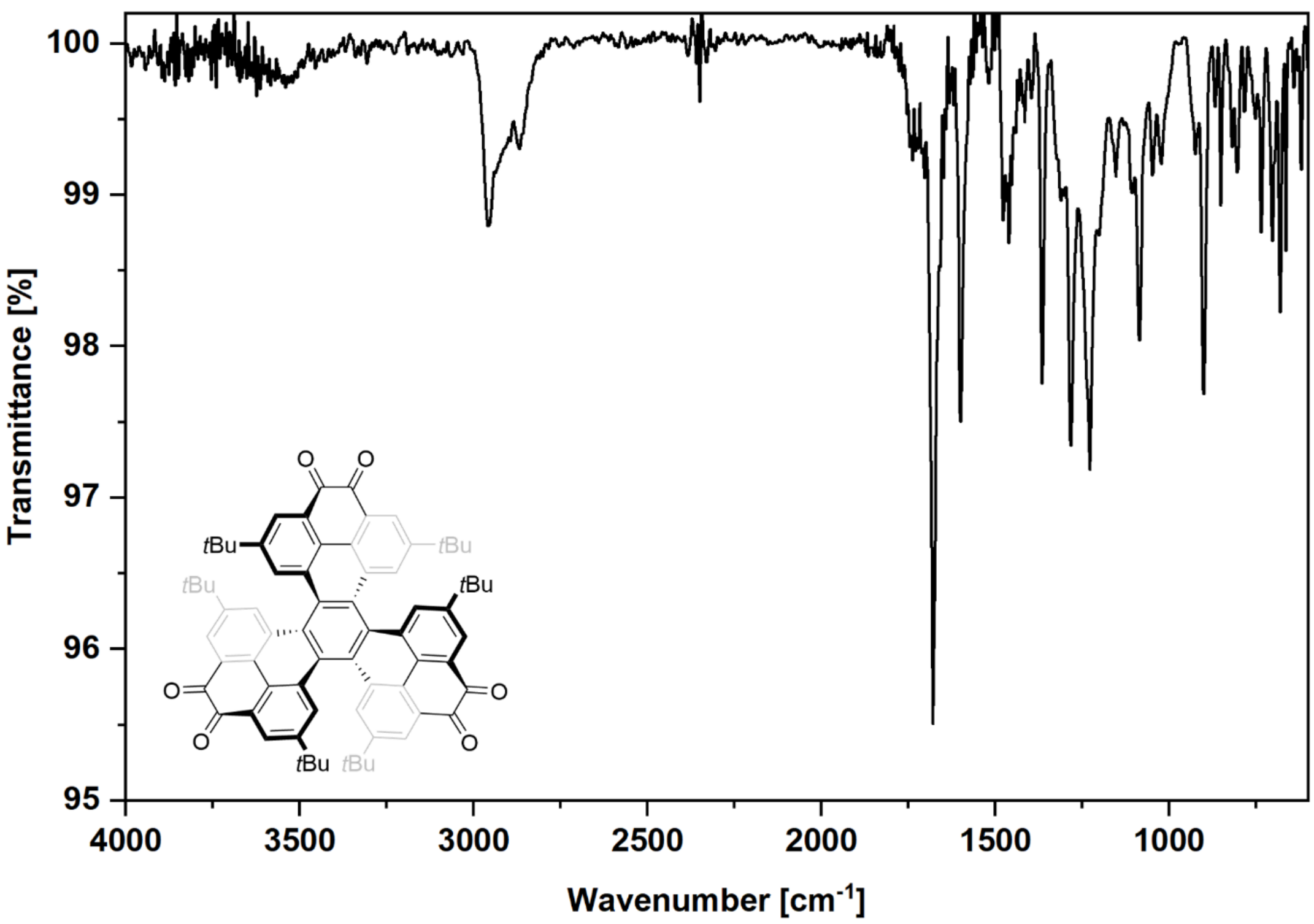

Figure S35: IR-spectrum (ATR, r.t.) of ***rac*-*D*$_3$-3**.

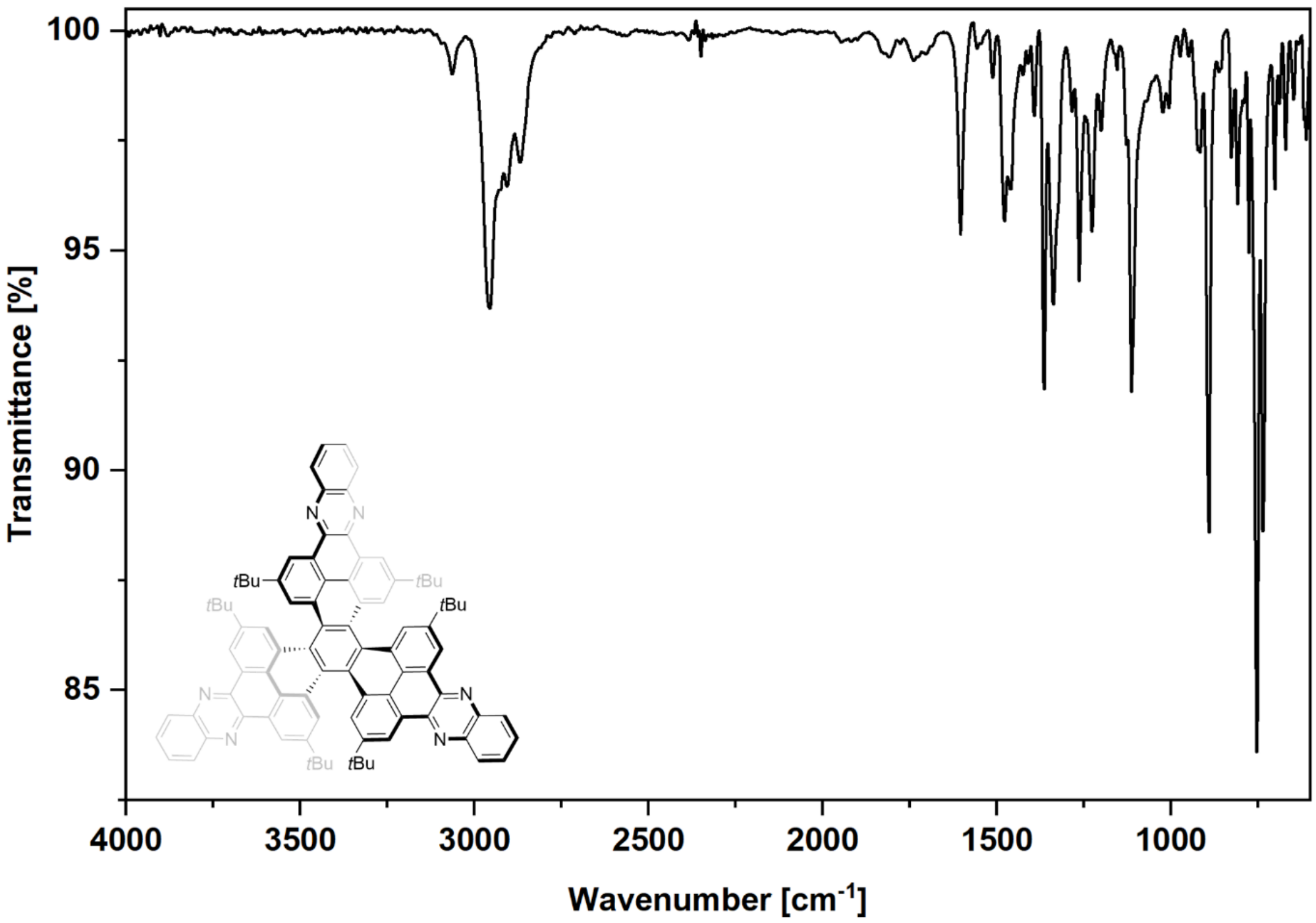

Figure S36: IR-spectrum (ATR, r.t.) of ***rac*-*C*$_2$-4**.

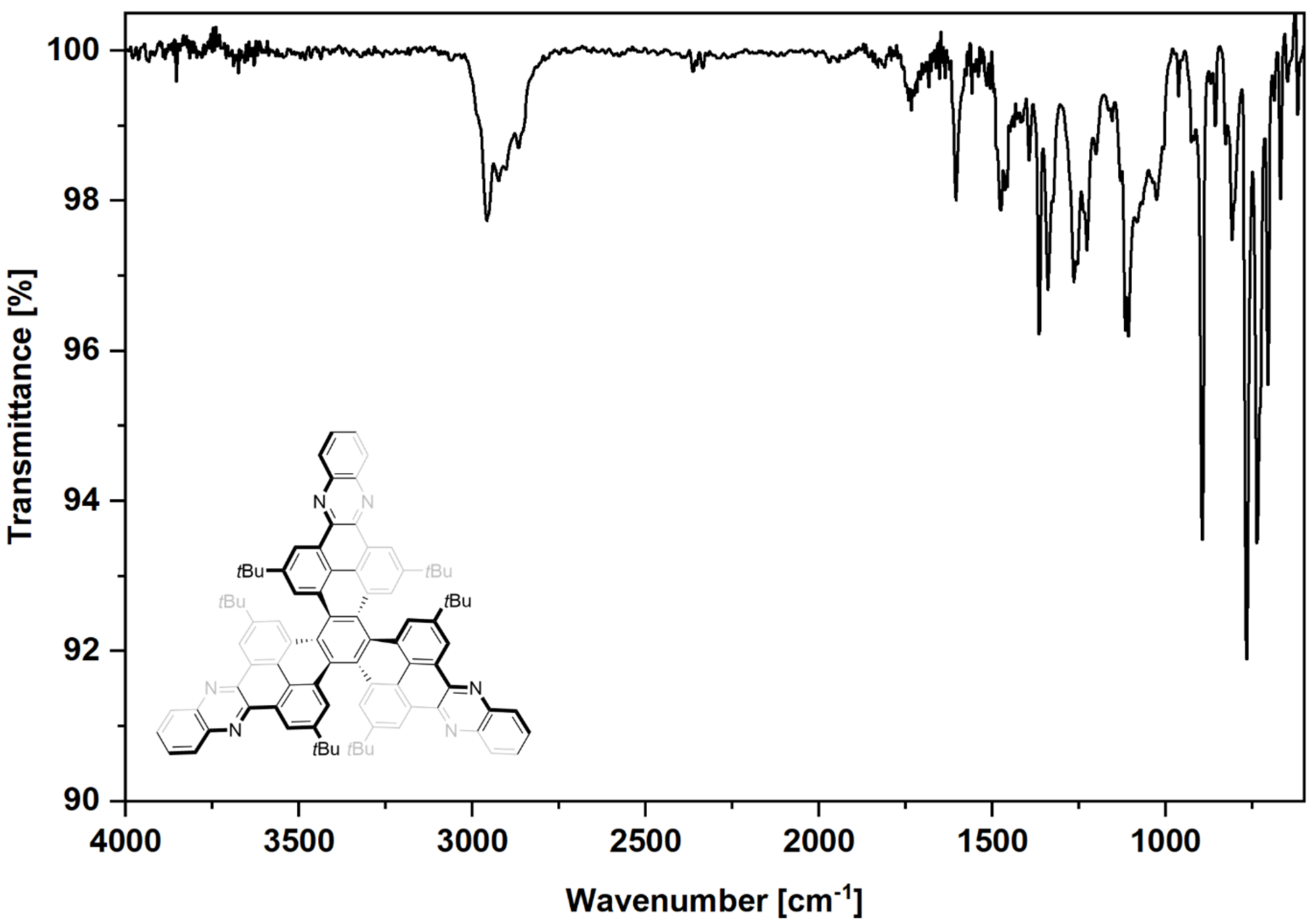

Figure S37: IR-spectrum (ATR, r.t.) of ***rac*-*D*$_3$-4**.

5. UV/Vis Absorption & Fluorescence Spectra

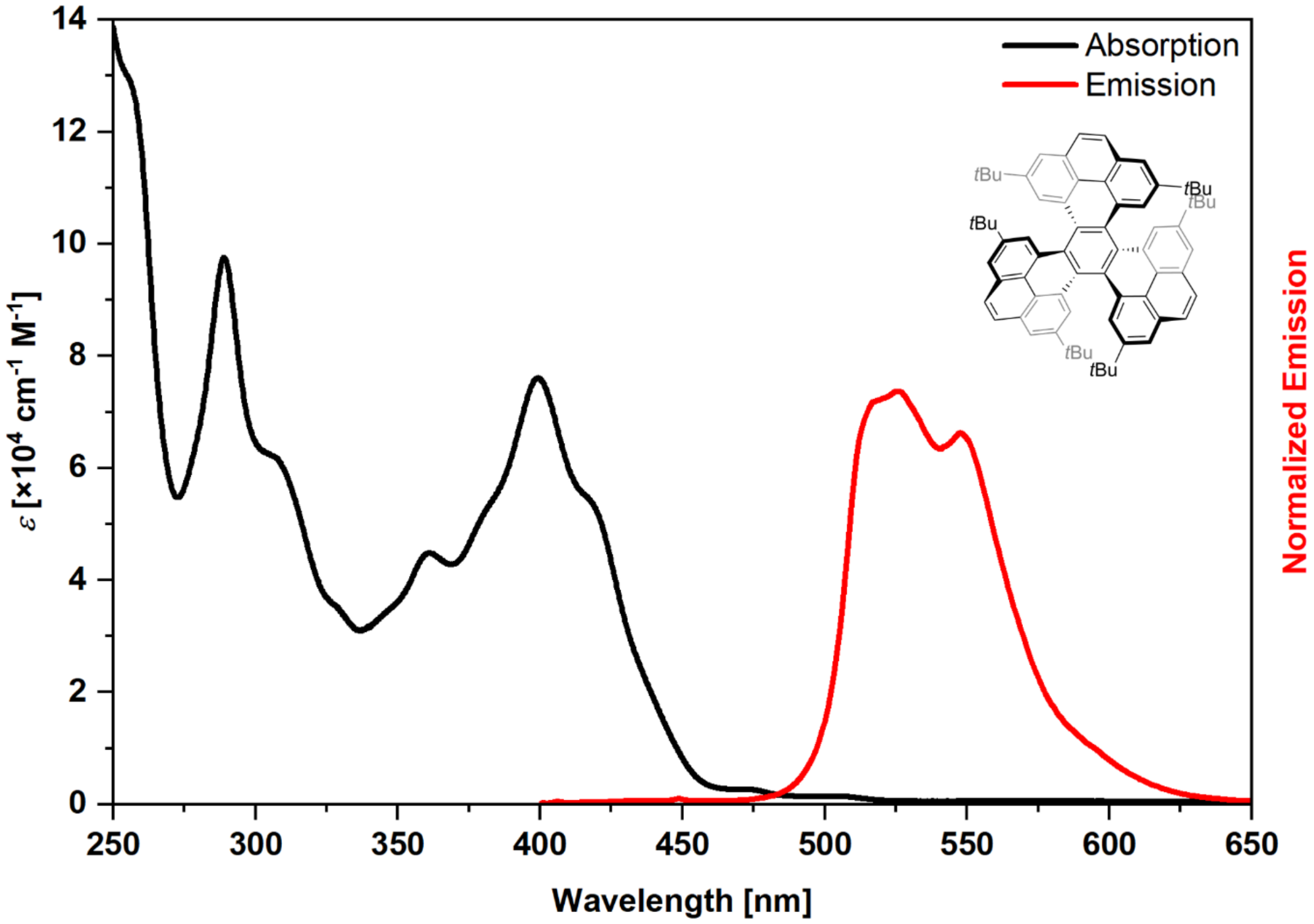

Figure S38: UV-Vis and fluorescence spectrum (DCM, r.t., λ_{ex} = 395 nm) of ***rac*-*D*$_3$-2**.

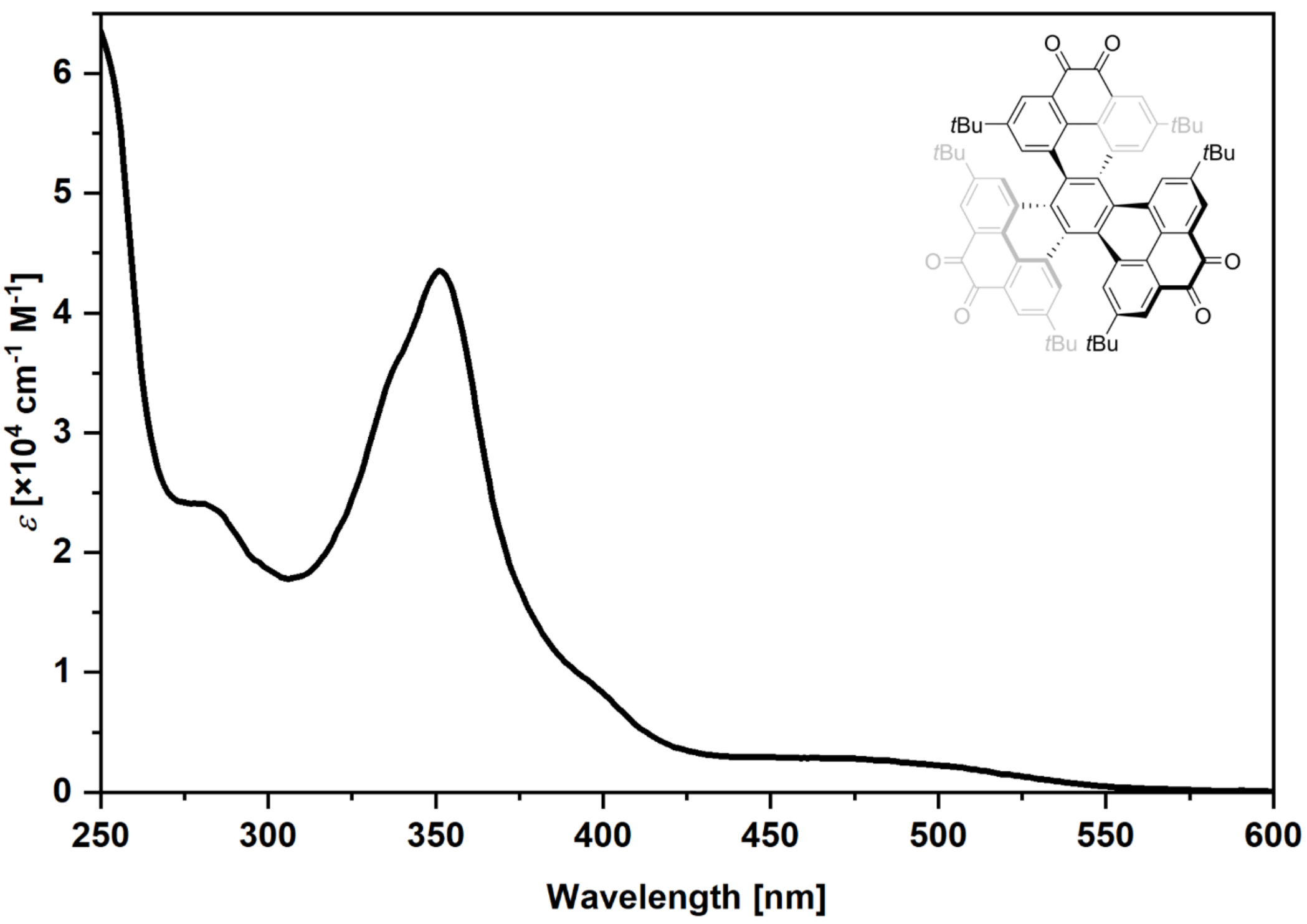

Figure S39: UV-Vis spectrum (DCM, r.t.) of ***rac-C*$_2$-3**.

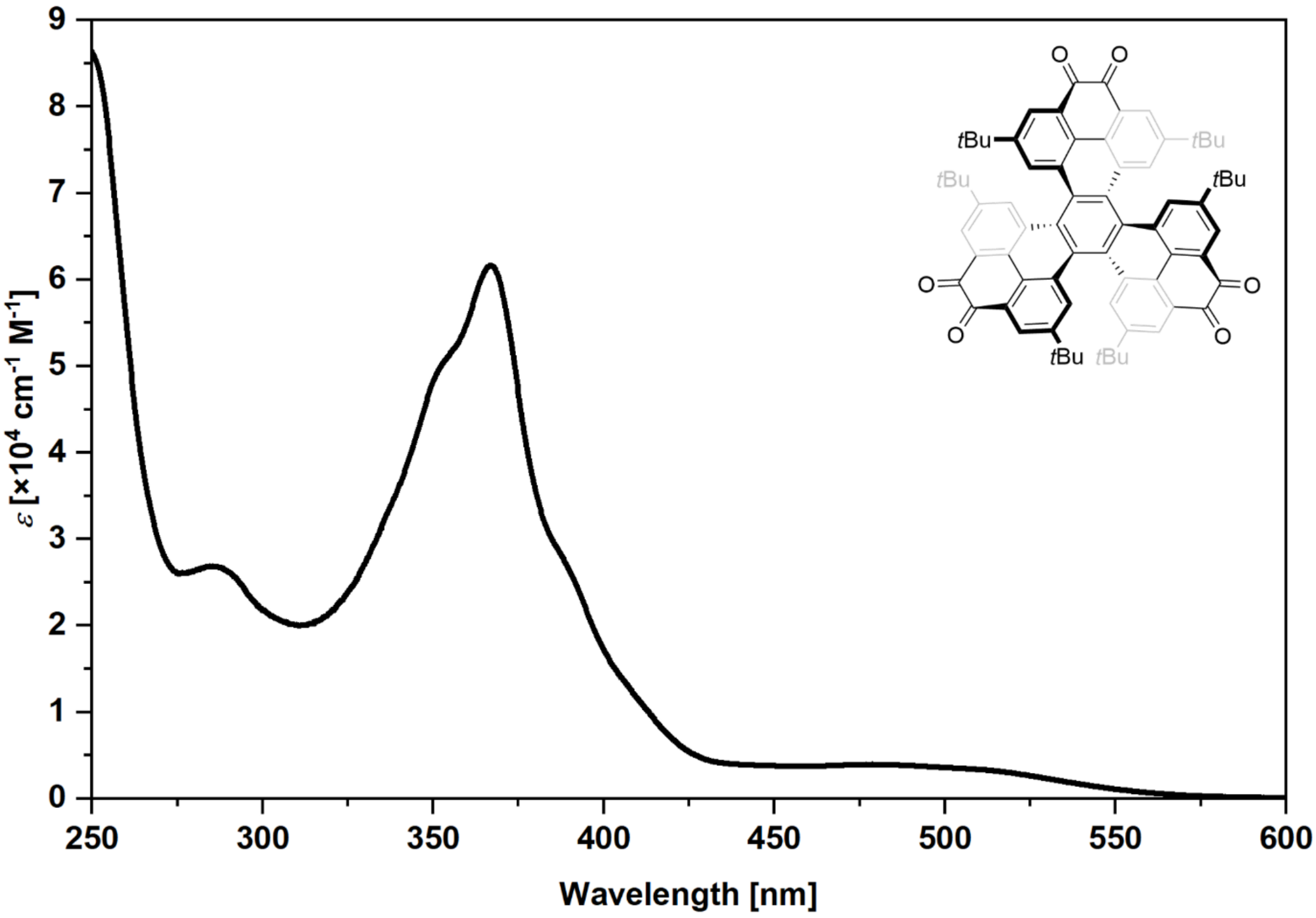

Figure S40: UV-Vis spectrum ($CHCl_3$, r.t.) of ***D*$_3$-3**.

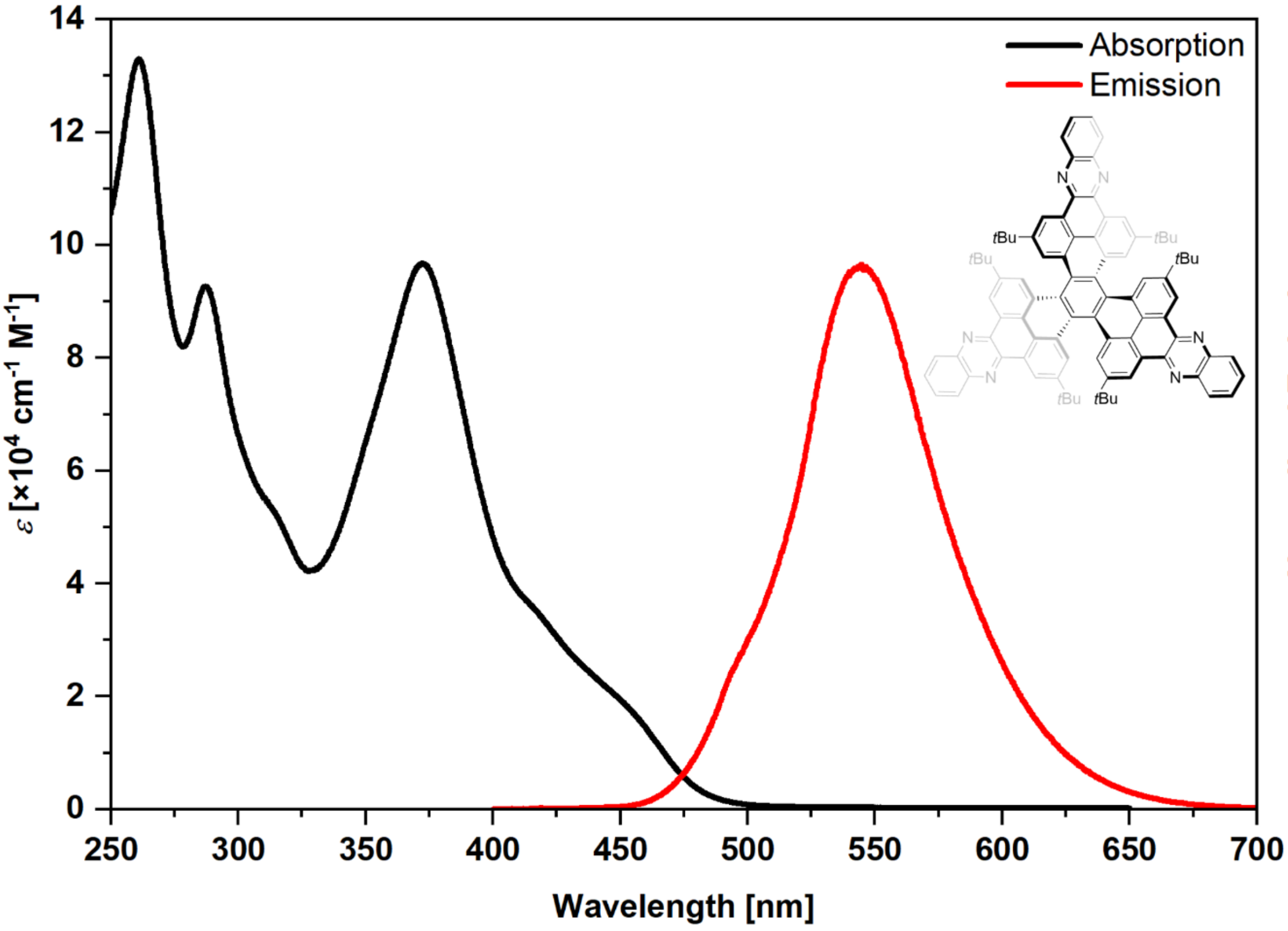

Figure S41: UV-Vis and fluorescence spectrum ($CHCl_3$, r.t., λ_{ex} = 287 nm) of ***C*₂-4**.

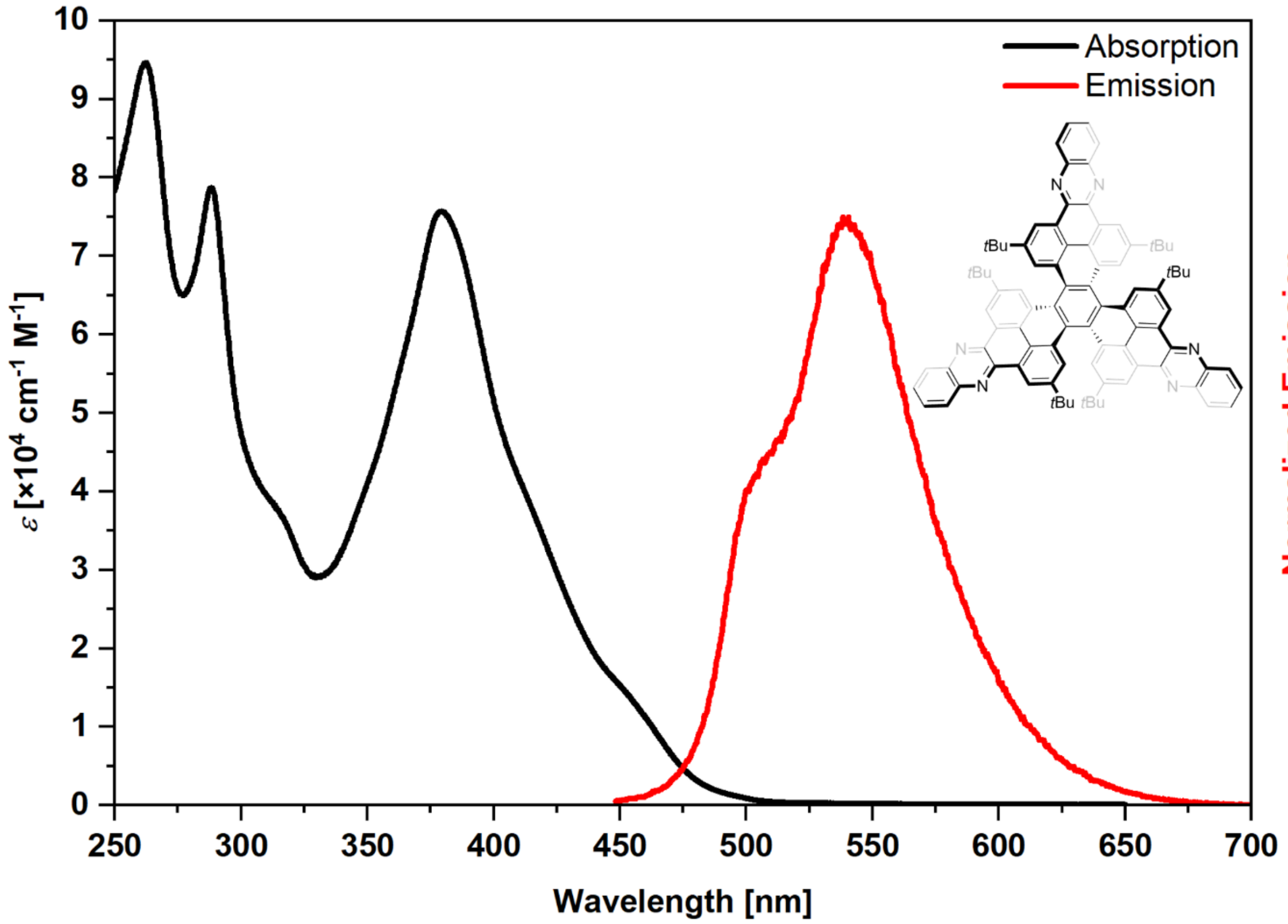

Figure S42: UV-Vis and fluorescence spectrum ($CHCl_3$, r.t., λ_{ex} = 288 nm) of ***D*₃-4**.

6. CD spectra

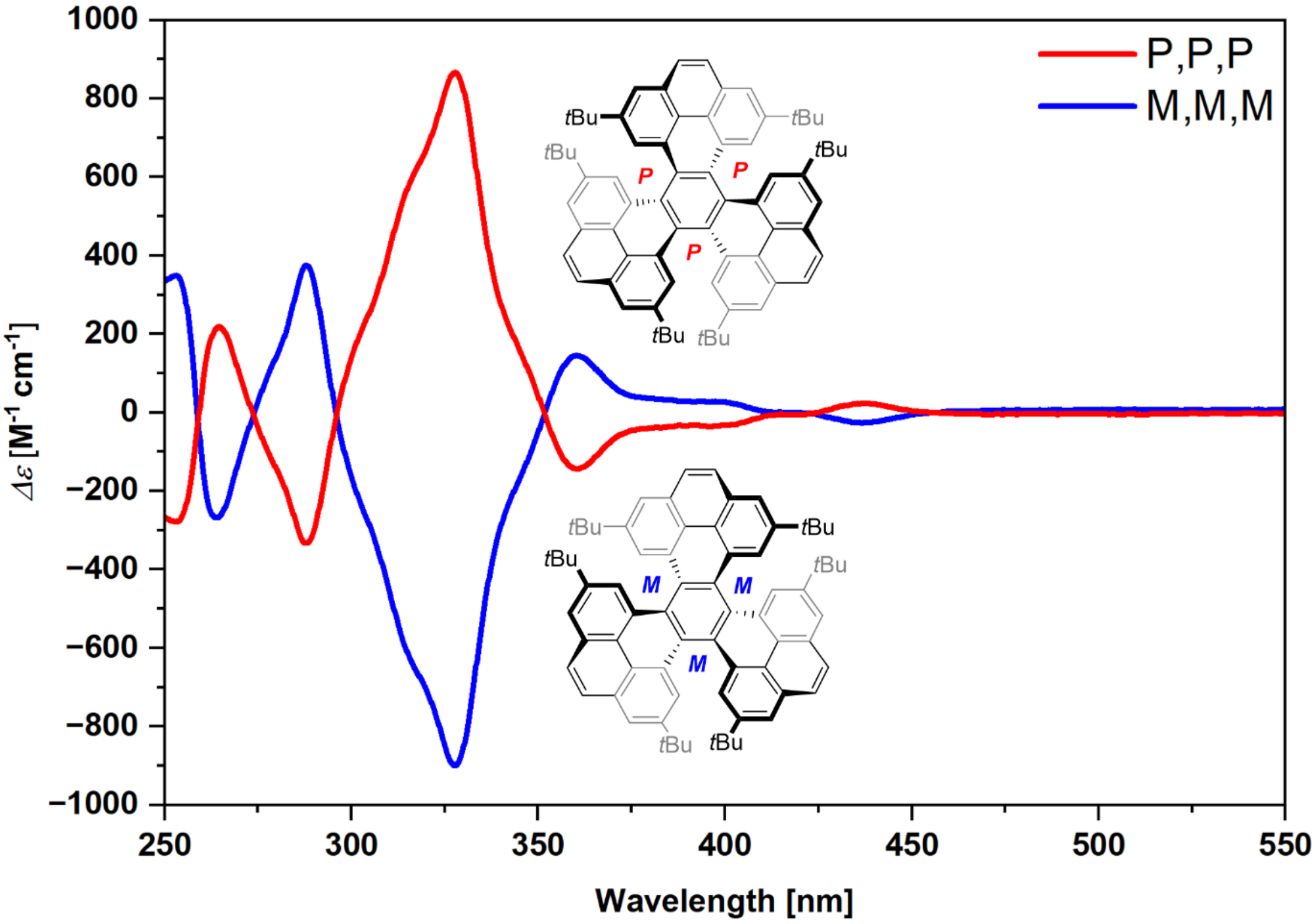

Figure S43: CD-spectrum (DCM, r.t.) of enantiopure ***D*₃-2**. The enantiomers were separated by chiral-HPLC (CHIRALPAK® IA column, 20 mL/min) using *n*-heptane as eluent to give ***(M,M,M)-D*₃-2** (t_{Ret} = 4.48 min.) and ***(P,P,P)-D*₃-2** (t_{Ret} = 4.87 min.) as yellow powders.

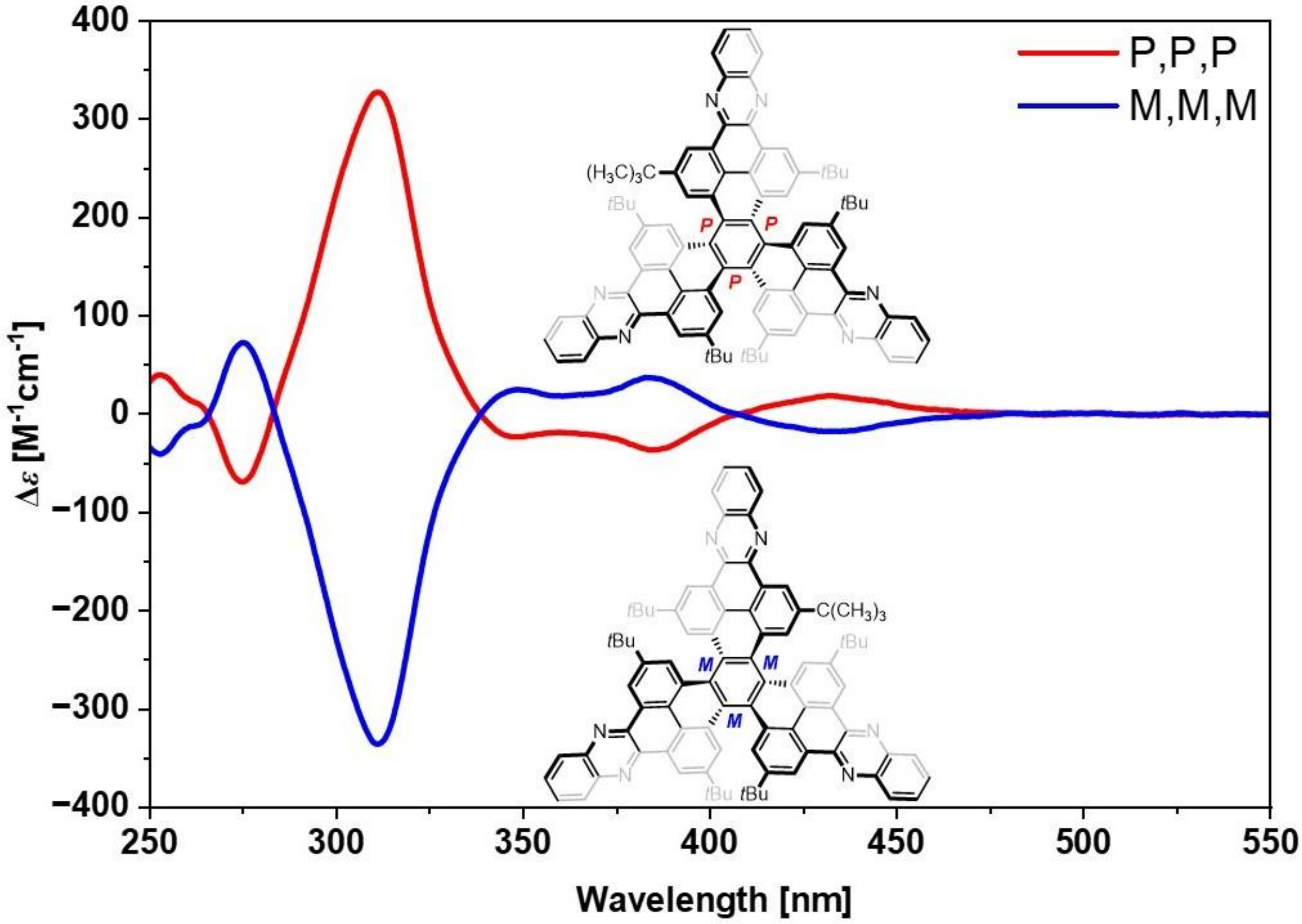

Figure S44: CD-spectrum (DCM, r.t.) of enantiopure ***D*₃-4**. The enantiomers were separated by chiral-HPLC (CHIRALPAK® IA column, 6 mL/min) using *n*-heptane/*iso*-propanol (99.5/0.5) as eluent to give ***(P,P,P)-D*₃-4** (t_{Ret} = 14.4 min.) and ***(M,M,M)-D*₃-4** (t_{Ret} = 15.7 min.) as yellow powders.

7. Mass Spectra

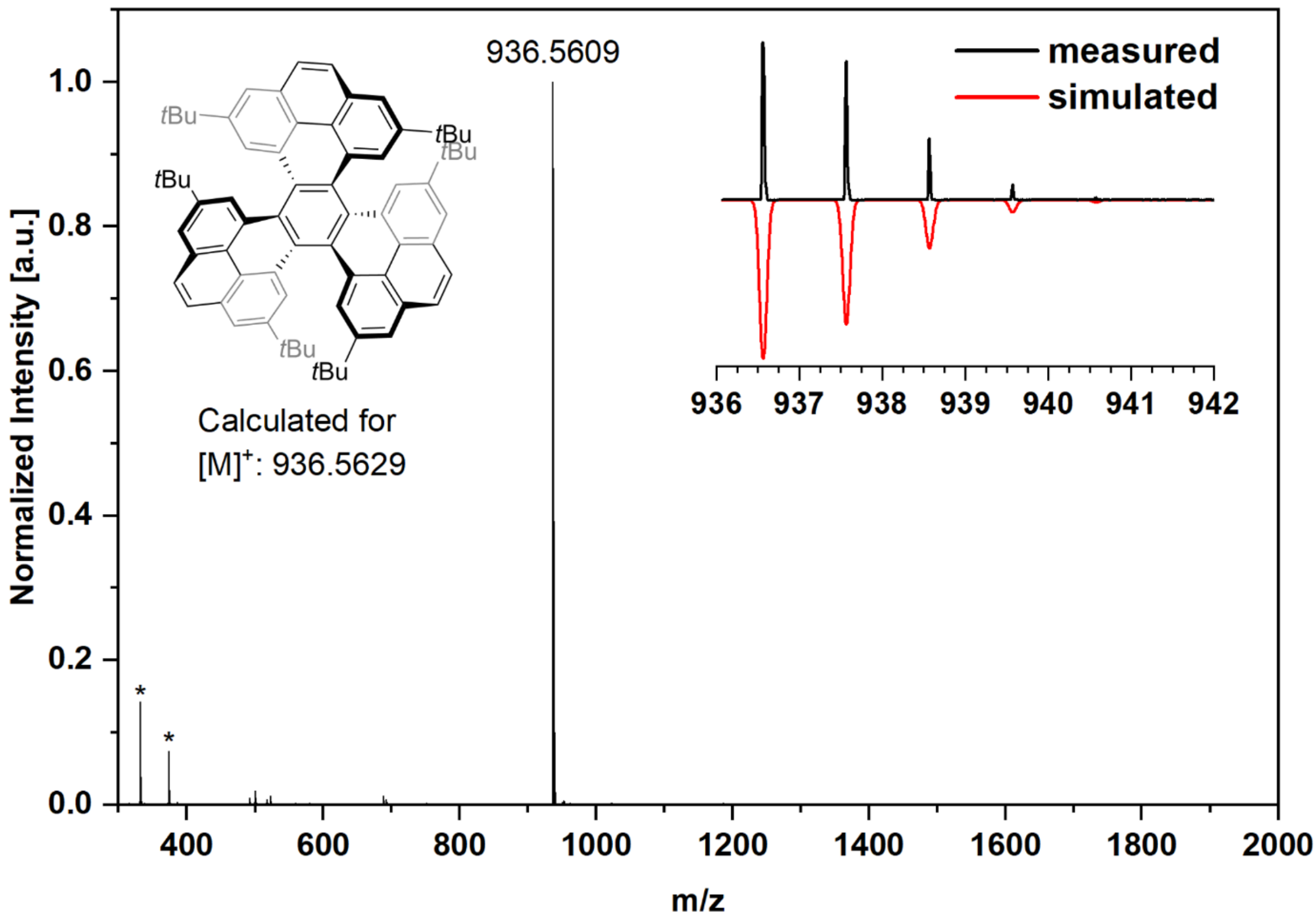

Figure S45: High resolution mass spectrum (MALDI+, DCTB) and simulated isotopic pattern of $\boldsymbol{D_3}$-**2**. *: Machine artifacts.

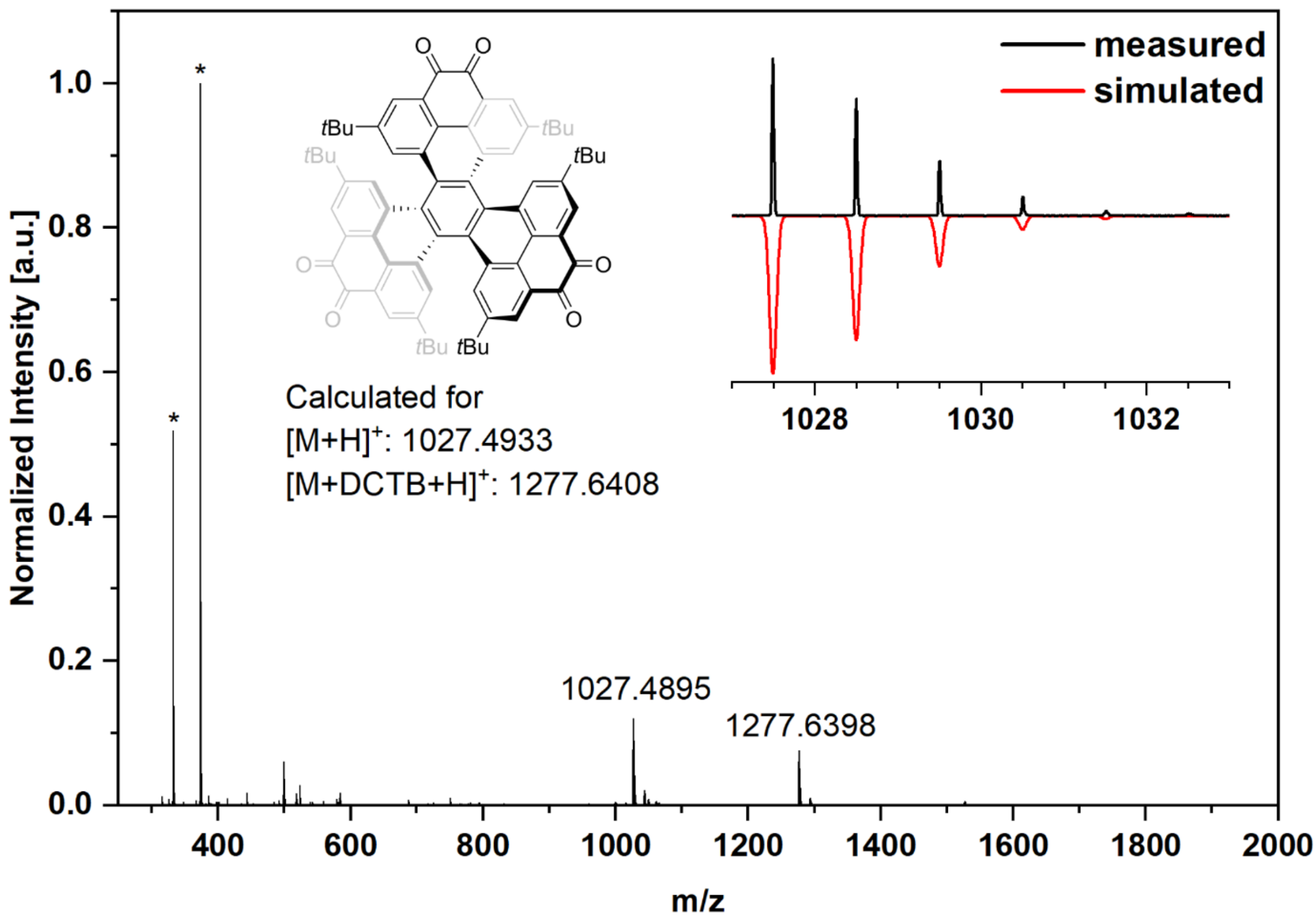

Figure S46: High resolution mass spectrum (MALDI+, DCTB) and simulated isotopic pattern of $\boldsymbol{C_2}$-**3**. *: Machine artifacts.

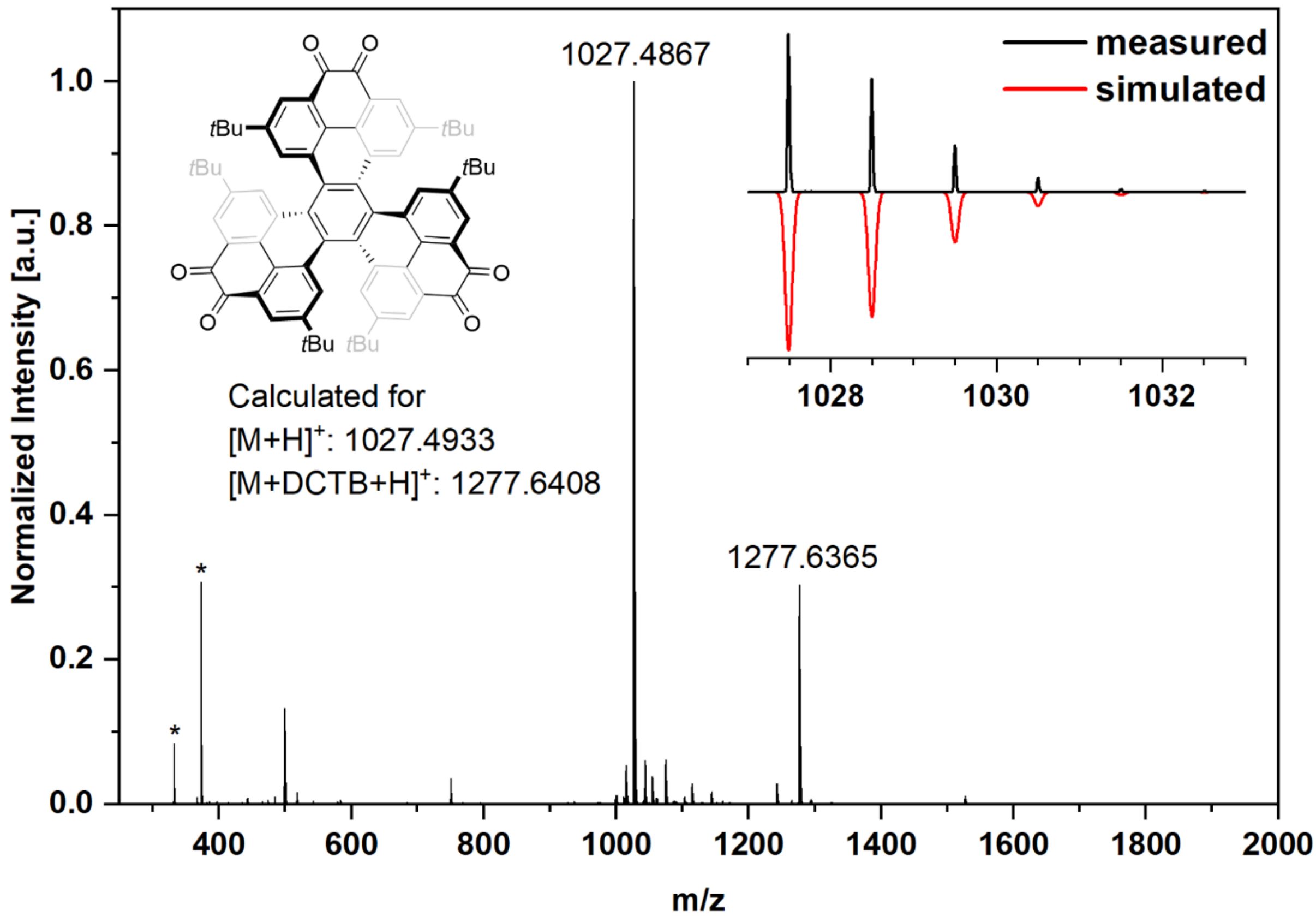

Figure S47: High resolution mass spectrum (MALDI+, DCTB) and simulated isotopic pattern of ***D*₃-3**. *: Machine artifacts.

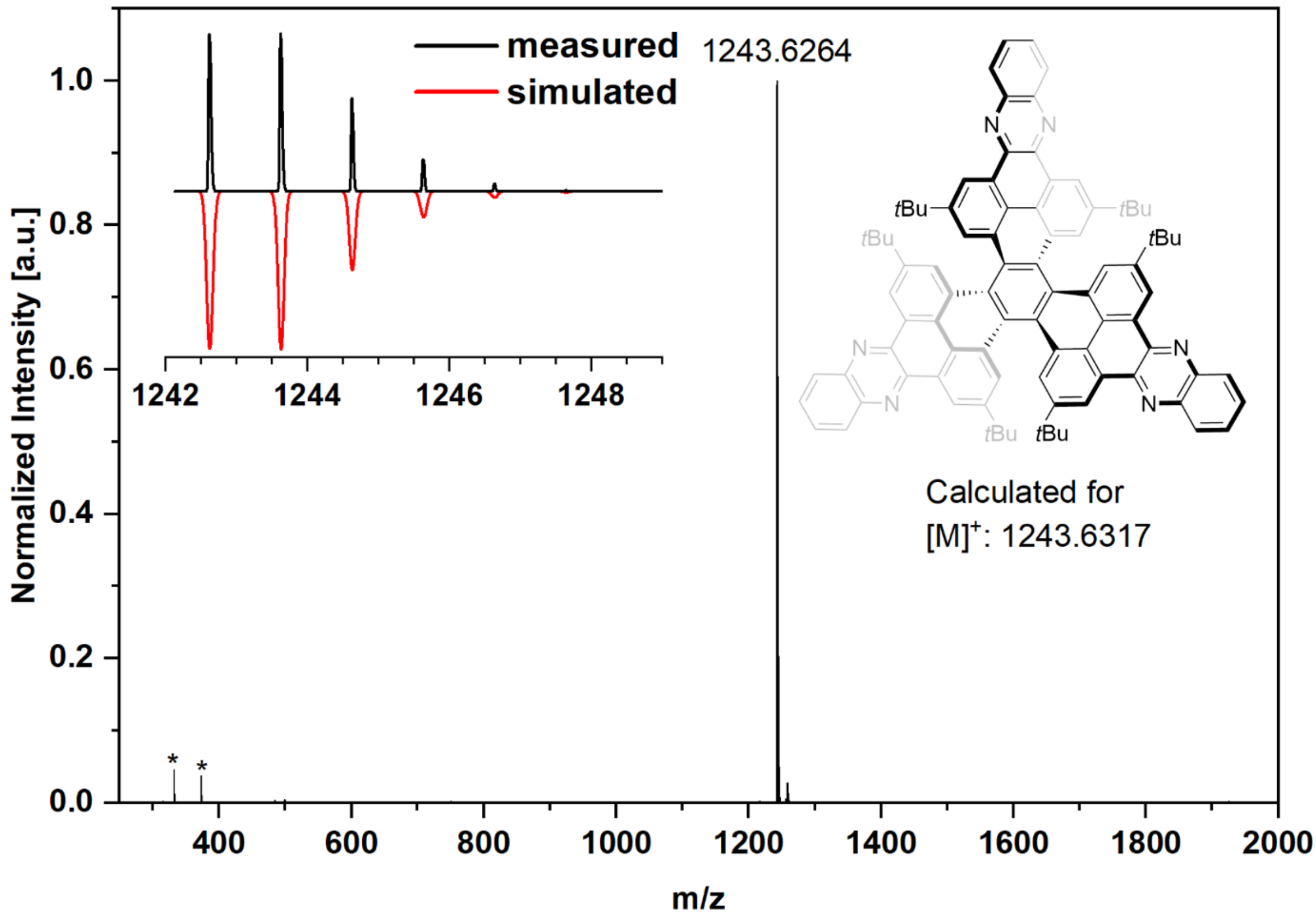

Figure S48: High resolution mass spectrum (MALDI+, DCTB) and simulated isotopic pattern of ***C*₂-4**. *: Machine artifacts.

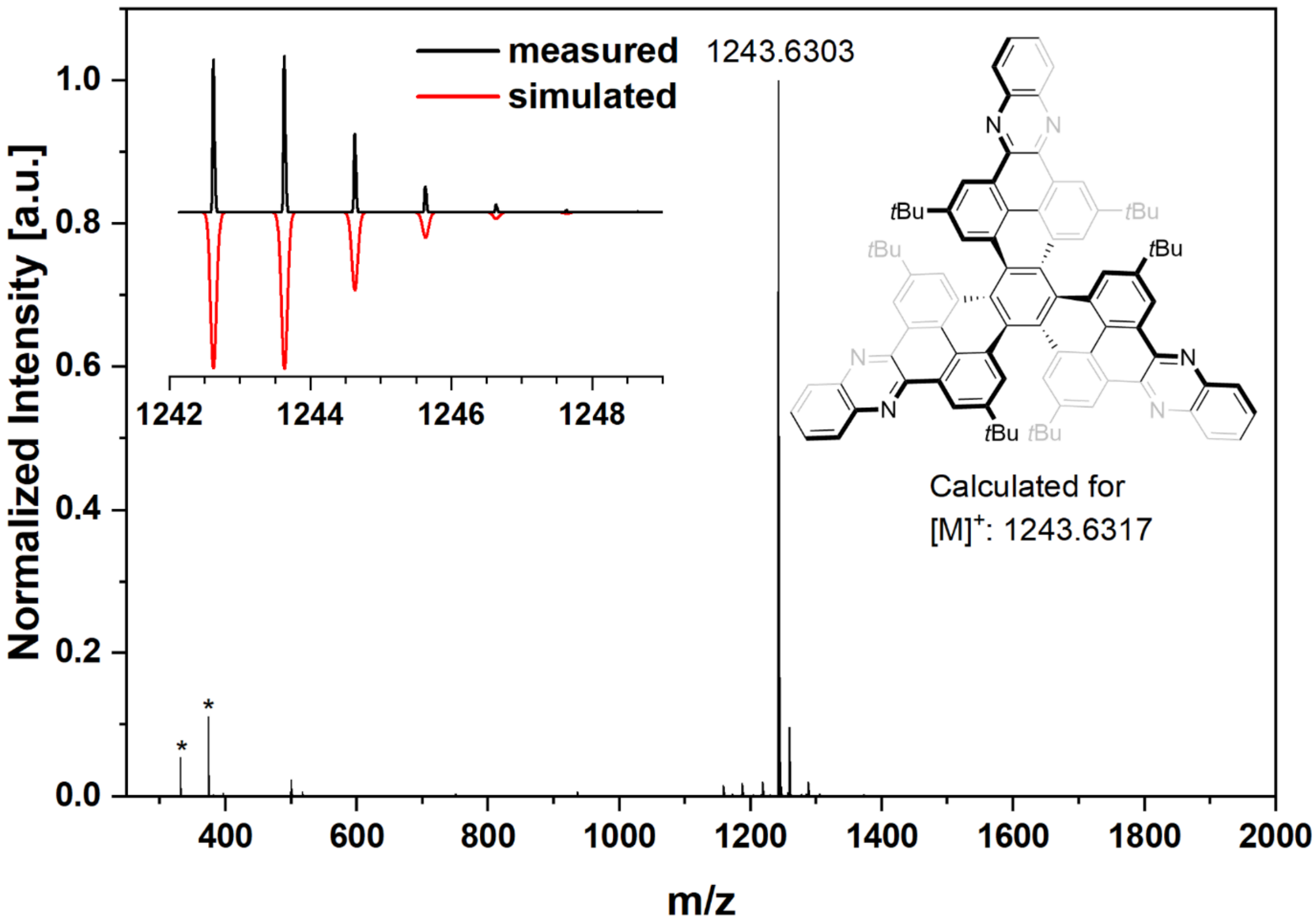

Figure S49: High resolution mass spectrum (MALDI$^+$, DCTB) and simulated isotopic pattern of ***D*$_3$-4**. *: Machine artifacts.

8. Crystallographic Data

2,5,10,13,18,21-Hexa-tert-butyltripyrenylene *rac-D*$_3$-2

Crystals suitable for single crystal X-ray diffraction of ***rac-D*$_3$-2** were obtained by slow evaporation of solvent of a concentrated solution of ***rac-D*$_3$-2** in $CDCl_3$.

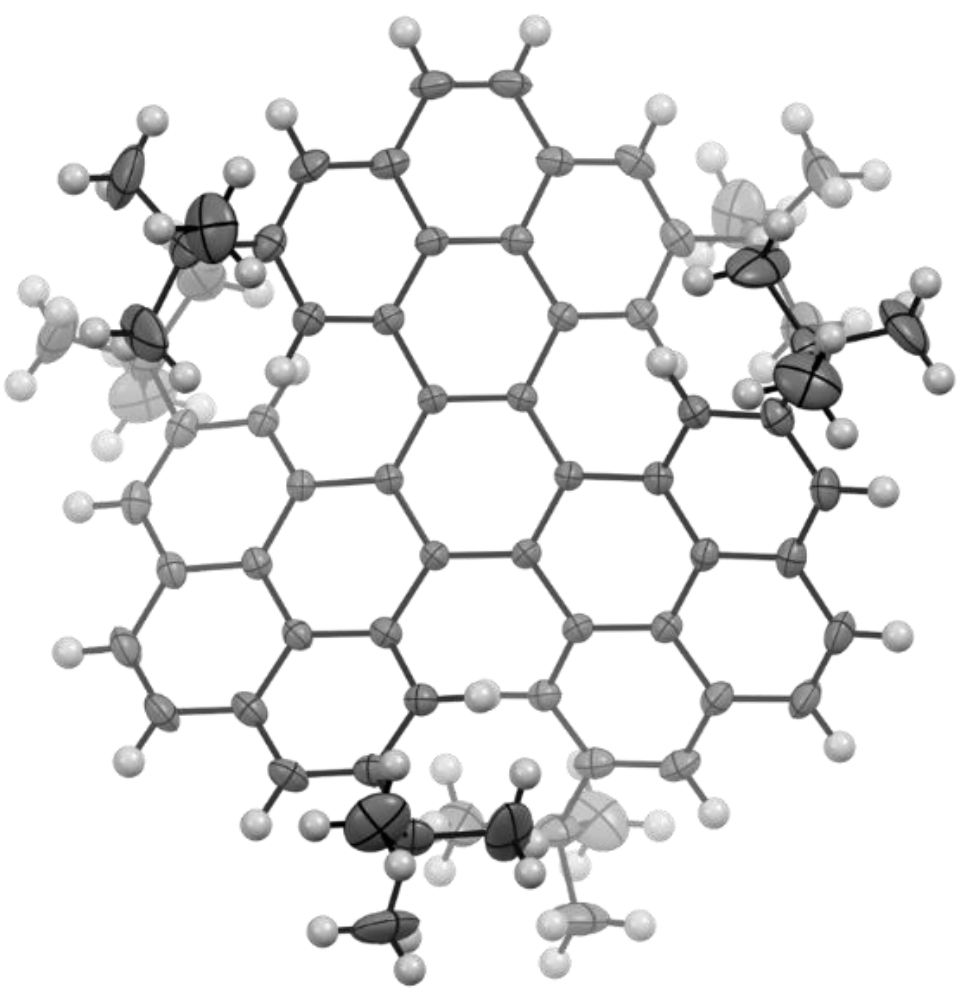

Figure S50: Single crystal structure of ***rac-D*$_3$-2** shown as thermal ellipsoids with a probability of 50%. Light grey: hydrogen, dark grey: carbon.

CCDC-number	2517636	
Empirical formula	$C_{75}H_{75}Cl_9$	
Formula weight	1295.40	
Temperature	200(2) K	
Wavelength	0.71073 Å	
Crystal system	trigonal	
Space group	$P\bar{3}$	
Z	2	
Unit cell dimensions	a = 16.2711(6) Å	α = 90°
	b = 16.2711(6) Å	β = 90°
	c = 14.8272(8) Å	γ = 120°
Volume	3399.6(3) $Å^3$	
Density (calculated)	1.26 g/cm^3	
Absorption coefficient	0.41 mm^{-1}	
Crystal shape	polyhedron	
Crystal size	0.101 x 0.091 x 0.076 mm^3	
Crystal colour	yellow	
Theta range for data collection	1.4 to 29.1°	
Index ranges	$-21 \leq h \leq 22$, $-22 \leq k \leq 22$, $-20 \leq l \leq 20$	
Reflections collected	55476	
Independent reflections	6062 (R(int) = 0.0636)	
Observed reflections	3813 (I > 2σ(I))	
Absorption correction	Semi-empirical from equivalents	
Max. and min. transmission	0.96 and 0.93	
Refinement method	Full-matrix least-squares on F^2	
Data/restraints/parameters	6062 / 528 / 359	
Goodness-of-fit on F^2	1.02	
Final R indices (I>2σ(I))	R1 = 0.075, wR2 = 0.211	
Largest diff. peak and hole	0.81 and -0.66 $eÅ^{-3}$	

2,5,10,13,18,21-Hexa-*tert*-butyltripyrenylene-7,8,15,16,23,24-hexaone *rac*-*C*$_2$-3

Suitable crystals for single crystal X-ray diffraction of ***rac*-*C*$_2$-3** were obtained by slow diffusion of liquid methanol into a solution of ***rac*-*C*$_2$-3** in DCM.

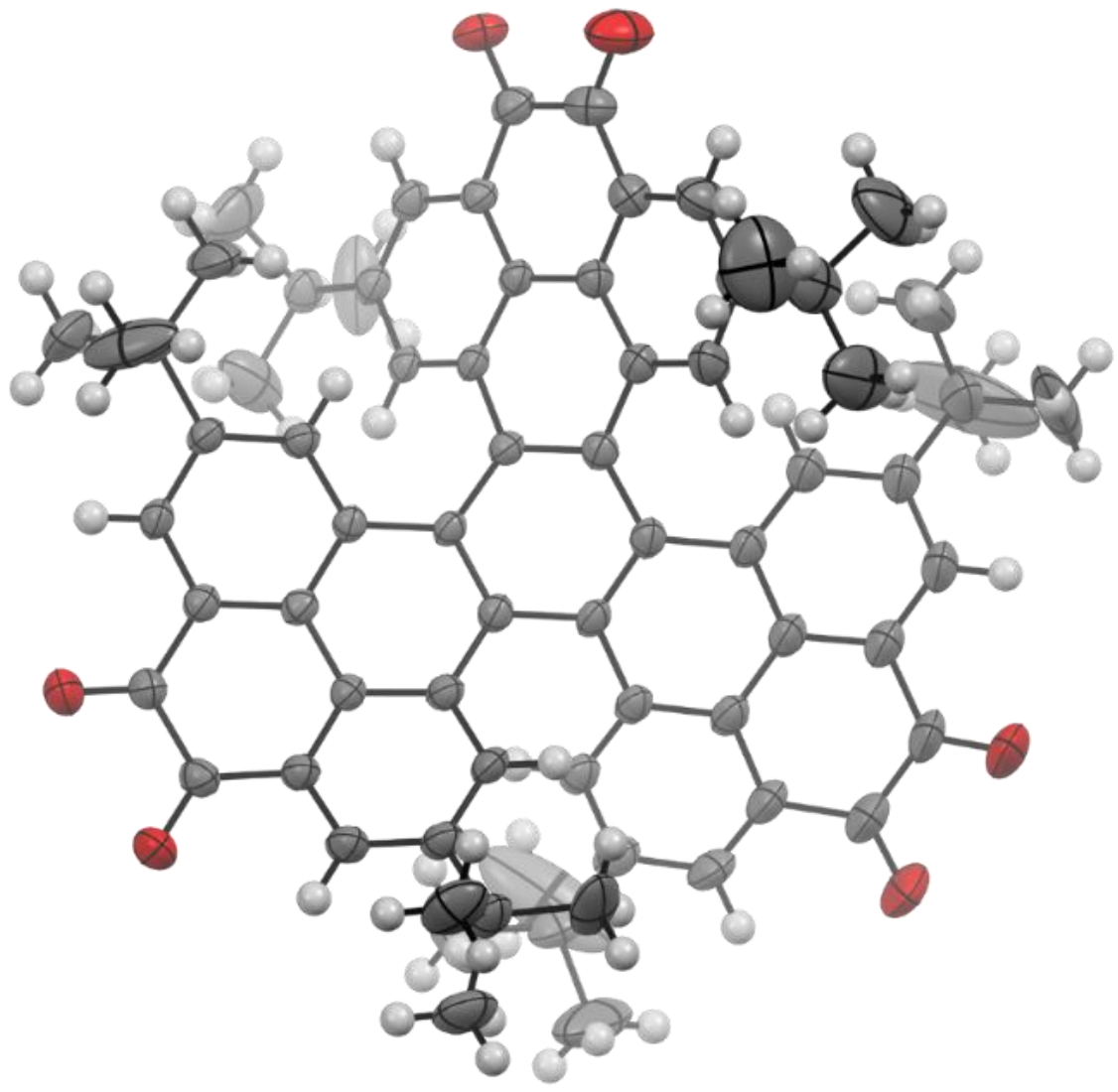

Figure S51: Single crystal structure of ***C*$_2$-3** shown as thermal ellipsoids with a probability of 50%. Light grey: hydrogen, dark grey: carbon, red: oxygen.

CCDC-number	2517637	
Empirical formula	$C_{72.50}H_{67}ClO_6$	
Formula weight	1069.71	
Temperature	200(2) K	
Wavelength	0.71073 Å	
Crystal system	triclinic	
Space group	$P\bar{1}$	
Z	4	
Unit cell dimensions	a = 13.9925(10) Å	α = 75.7246(15)°
	b = 18.3910(12) Å	β = 82.5811(17)°
	c = 27.2269(18) Å	γ = 89.9622(17)°
Volume	6729.7(8) Å^3	
Density (calculated)	1.06 g/cm^3	
Absorption coefficient	0.10 mm^{-1}	
Crystal shape	plank	
Crystal size	0.564 x 0.095 x 0.036 mm^3	
Crystal colour	orange	
Theta range for data collection	0.8 to 22.7°	
Index ranges	$-15 \le h \le 15$, $-19 \le k \le 19$, $-29 \le l \le 29$	
Reflections collected	87392	
Independent reflections	18074 (R(int) = 0.0889)	
Observed reflections	10788 ($I > 2\sigma(I)$)	
Absorption correction	Semi-empirical from equivalents	
Max. and min. transmission	0.96 and 0.69	
Refinement method	Full-matrix least-squares on F^2	
Data/restraints/parameters	18074 / 5780 / 1460	
Goodness-of-fit on F^2	1.03	
Final R indices ($I>2\sigma(I)$)	R1 = 0.075, wR2 = 0.184	
Largest diff. peak and hole	0.43 and -0.48 eÅ^{-3}	

2,5,10,13,18,21-Hexa-*tert*-butyltripyrenylene-7,8,15,16,23,24-hexaone *rac-D*$_3$-3

Suitable crystals for single crystal X-ray diffraction of ***rac-D*$_3$-3** were obtained by slow diffusion of liquid methanol into a solution of ***rac-D*$_3$-3** in DCM.

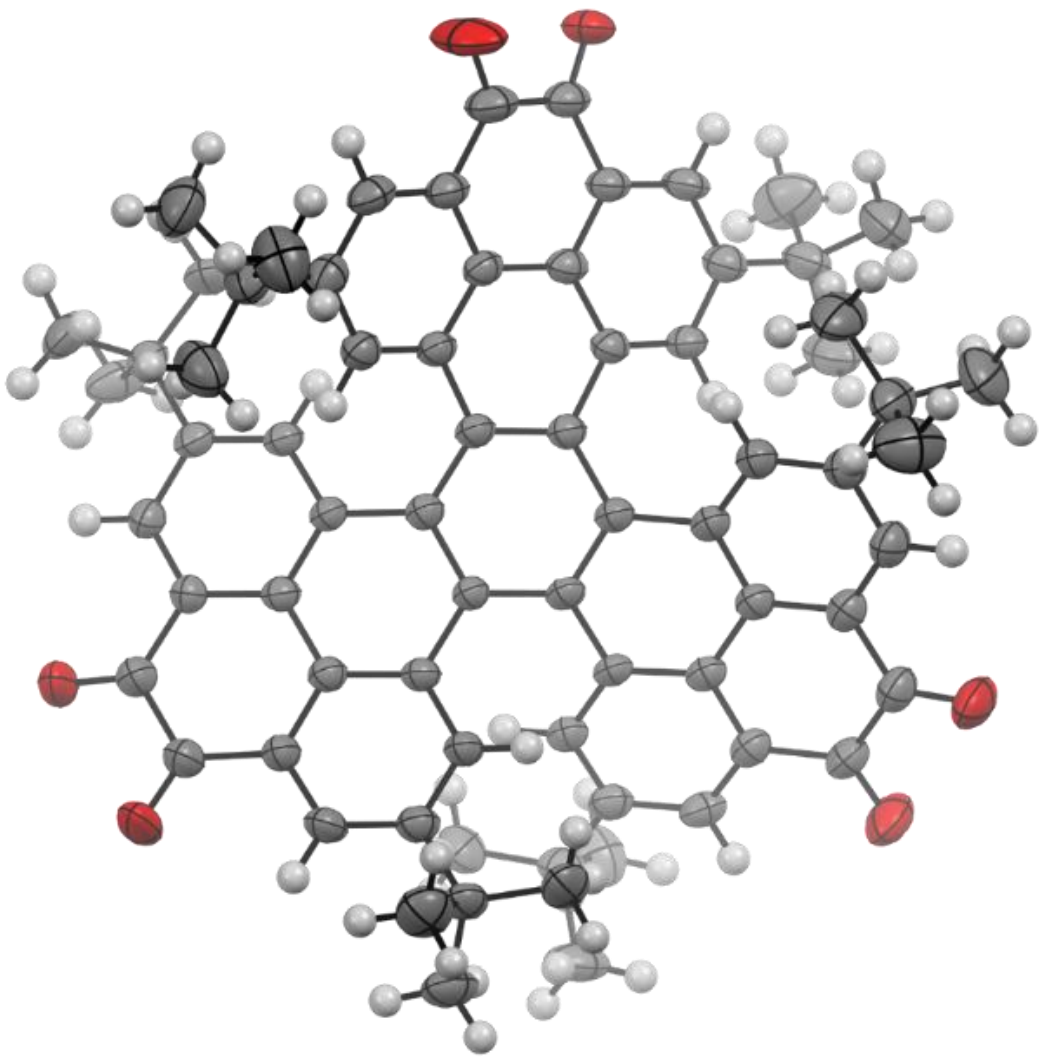

Figure S52: Single crystal structure of ***rac-D*$_3$-3** shown as thermal ellipsoids with a probability of 50%. Light grey: hydrogen, dark grey: carbon, blue: nitrogen.

CCDC-number	2517638	
Empirical formula	$C_{73}H_{67}Cl_3O_6$	
Formula weight	1146.61	
Temperature	200(2) K	
Wavelength	1.54178 Å	
Crystal system	triclinic	
Space group	$P\bar{1}$	
Z	2	
Unit cell dimensions	a = 14.5474(5) Å	α =95.445(3)°
	b = 16.5714(5) Å	β =104.322(3)°
	c = 17.0130(5) Å	γ =112.559(3)°
Volume	3586.3(2) Å^3	
Density (calculated)	1.06 g/cm^3	
Absorption coefficient	1.51 mm^{-1}	
Crystal shape	plank	
Crystal size	0.111 x 0.105 x 0.038 mm^3	
Crystal colour	orange	
Theta range for data collection	2.7 to 68.7°	
Index ranges	$-13 \leq h \leq 17$, $-19 \leq k \leq 16$, $-20 \leq l \leq 16$	
Reflections collected	43926	
Independent reflections	12764 (R(int) = 0.0480)	
Observed reflections	7352 ($I > 2\sigma(I)$)	
Absorption correction	Semi-empirical from equivalents	
Max. and min. transmission	0.99 and 0.83	
Refinement method	Full-matrix least-squares on F^2	
Data/restraints/parameters	12764 / 798 / 809	
Goodness-of-fit on F^2	1.03	
Final R indices (I>2sigma(I))	R1 = 0.063, wR2 = 0.154	
Largest diff. peak and hole	0.21 and -0.22 eÅ^{-3}	

Trisphenanthrophenazine *rac- D*$_3$-4

Suitable crystals for single crystal X-ray diffraction of ***rac- D*$_3$-4** were obtained by slow evaporation of solvent of a concentrated solution of ***rac-D*$_3$-4** in $CHCl_3$.

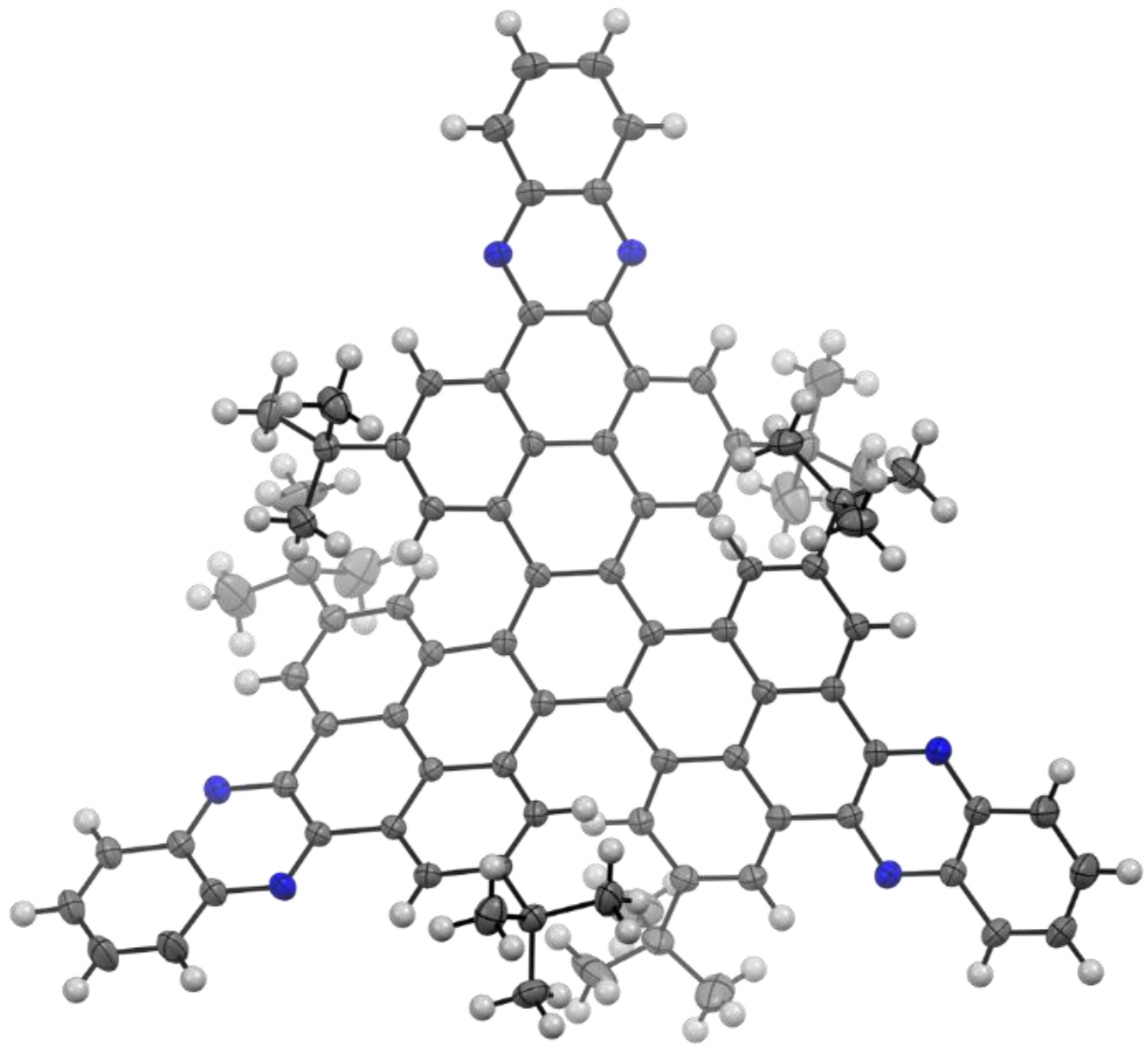

Figure S53: Single crystal structure of ***rac-D*$_3$-4** shown as thermal ellipsoids with a probability of 50%. Light grey: hydrogen, dark grey: carbon, blue: nitrogen.

CCDC-number	2517639	
Empirical formula	$C_{95.50}H_{83}Cl_{16.50}N_6$	
Formula weight	1899.60	
Temperature	200(2) K	
Wavelength	1.54178 Å	
Crystal system	trigonal	
Space group	$R\bar{3}$	
Z	6	
Unit cell dimensions	a = 19.1108(4) Å	α = 90°
	b = 19.1108(4) Å	β = 90°
	c = 43.8514(14) Å	γ = 120°
Volume	13869.8(7) $Å^3$	
Density (calculated)	1.37 g/cm^3	
Absorption coefficient	4.87 mm^{-1}	
Crystal shape	prism	
Crystal size	0.105 x 0.086 x 0.053 mm^3	
Crystal colour	yellow	
Theta range for data collection	2.9 to 68.4°	
Index ranges	$-16\leq h\leq 23$, $-22\leq k\leq 21$, $-31\leq l\leq 52$	
Reflections collected	22512	
Independent reflections	5555 (R(int) = 0.0503)	
Observed reflections	3314 ($I > 2\sigma(I)$)	
Absorption correction	Semi-empirical from equivalents	
Max. and min. transmission	0.98 and 0.81	
Refinement method	Full-matrix least-squares on F^2	
Data/restraints/parameters	5555 / 561 / 448	
Goodness-of-fit on F^2	1.02	
Final R indices ($I>2\sigma(I)$)	R1 = 0.064, wR2 = 0.153	
Largest diff. peak and hole	0.49 and -0.39 $eÅ^{-3}$	

(*P*,*P*,*P*)-2,5,10,13,18,21-Hexa-tert-butyltripyrenylene (*P*,*P*,*P*)-*D*$_3$-2

Crystals suitable for single crystal X-ray diffraction of **(*P*,*P*,*P*)-*D*$_3$-2** could be obtained by gas vapor diffusion of ethanol into a concentrated solution of **(*P*,*P*,*P*)-*D*$_3$-2** in $CHCl_3$.

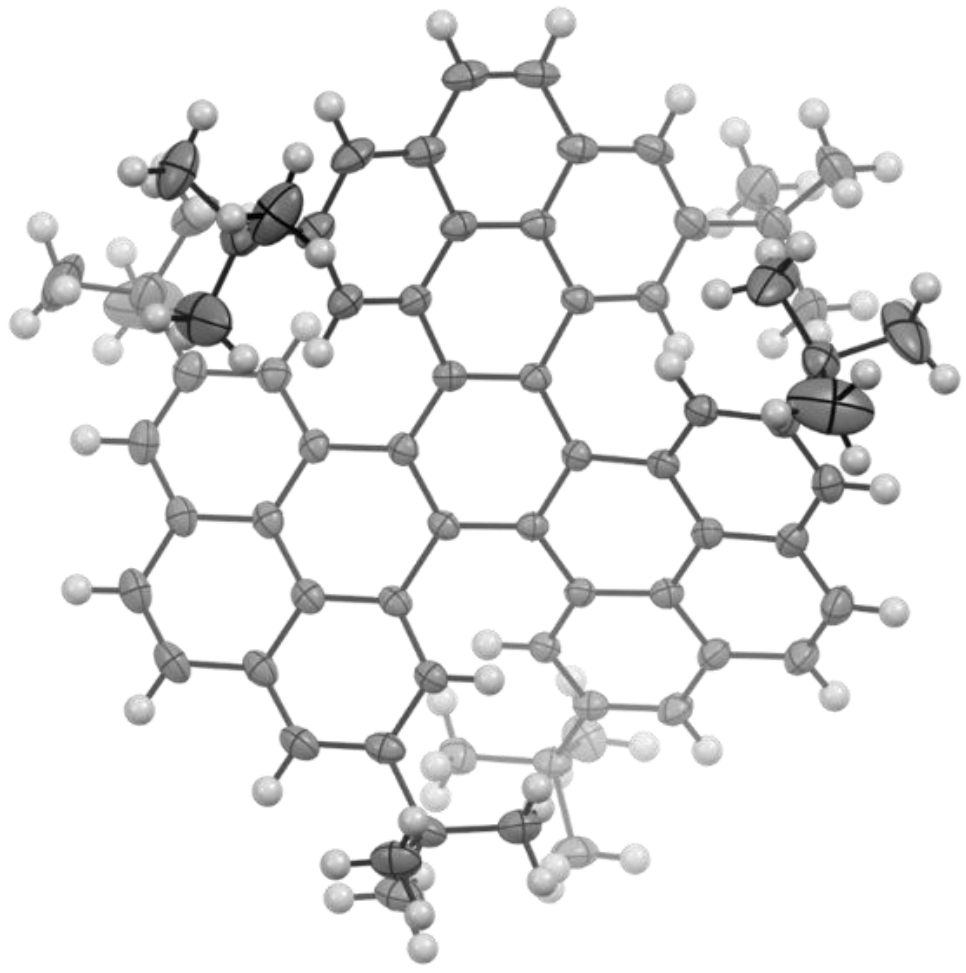

Figure S54: Single crystal structure of **(*P*,*P*,*P*)-*D*$_3$-2** portrayed as thermal ellipsoids with a probability of 50%. Light grey: hydrogen, dark grey: carbon.

CCDC-number	2517640	
Empirical formula	$C_{74}H_{78}O$	
Formula weight	983.36	
Temperature	200(2) K	
Wavelength	1.54178 Å	
Crystal system	orthorhombic	
Space group	$P2_12_12_1$	
Z	4	
Unit cell dimensions	a = 14.7343(3) Å	α = 90°
	b = 15.7207(3) Å	β = 90°
	c = 25.2507(4) Å	γ = 90°
Volume	5848.91(19) Å^3	
Density (calculated)	1.12 g/cm^3	
Absorption coefficient	0.48 mm^{-1}	
Crystal shape	column	
Crystal size	0.128 x 0.044 x 0.042 mm^3	
Crystal colour	orange	
Theta range for data collection	3.3 to 68.2°	
Index ranges	$-17 \leq h \leq 15$, $-15 \leq k \leq 18$, $-30 \leq l \leq 17$	
Reflections collected	34937	
Independent reflections	10445 (R(int) = 0.0198)	
Observed reflections	8952 ($I > 2\sigma(I)$)	
Absorption correction	Semi-empirical from equivalents	
Max. and min. transmission	0.99 and 0.89	
Refinement method	Full-matrix least-squares on F^2	
Data/restraints/parameters	10445 / 1191 / 743	
Goodness-of-fit on F^2	1.05	
Final R indices ($I>2\sigma(I)$)	R1 = 0.042, wR2 = 0.097	
Absolute structure parameter	0.99(19)	
Largest diff. peak and hole	0.16 and -0.29 eÅ^{-3}	

Trisphenanthrophenazine *M,M,M-D*$_3$-4

Suitable crystals for single crystal X-ray strudiffraction of ***M,M,M-D*$_3$-4** weree obtained by slow evaporation of solvent of a concentrated solution of ***M,M,M-D*$_3$-4** in $CDCl_3$.

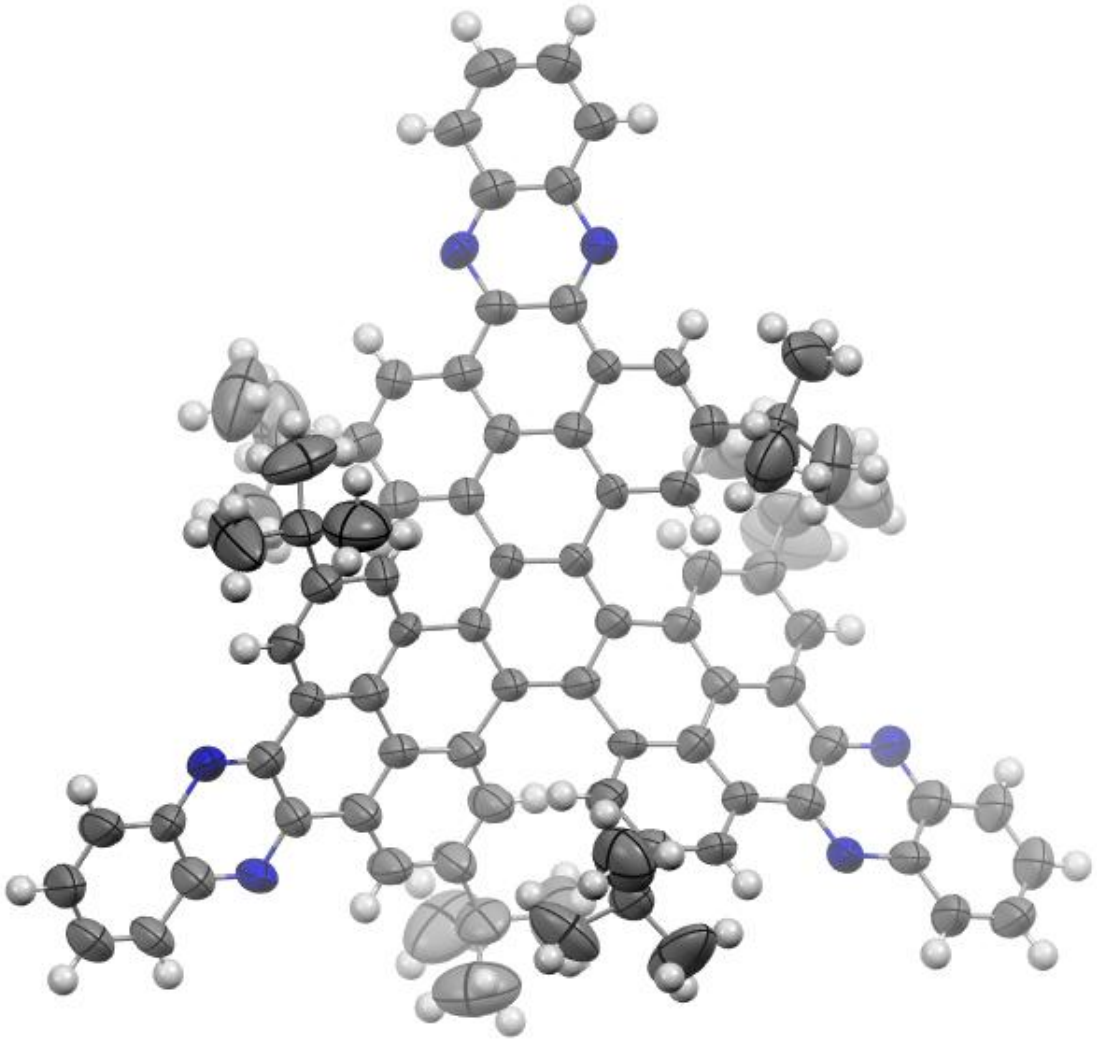

Figure S55: Single crystal structure of ***M,M,M-D*$_3$-4** portrayed as thermal ellipsoids with a probability of 50%. Hydrogen atoms drawn as fixed-sized spheres with a radius of 0.30 Å. Light grey: hydrogen, dark grey: carbon, blue: nitrogen.

CCDC-number	2517641	
Empirical formula	$C_{90}H_{78}N_6$	
Formula weight	1243.58	
Temperature	200(2) K	
Wavelength	1.54178 Å	
Crystal system	hexagonal	
Space group	$P6_322$	
Z	4	
Unit cell dimensions	a = 19.2330(3) Å	α = 90°
	b = 19.2330(3) Å	β = 90°
	c = 30.8475(9) Å	γ = 120°
Volume	9882.0(4) Å^3	
Density (calculated)	0.84 g/cm^3	
Absorption coefficient	0.37 mm^{-1}	
Crystal shape	brick	
Crystal size	0.060 x 0.040 x 0.020 mm^3	
Crystal colour	orange	
Theta range for data collection	4.8 to 48.4°	
Index ranges	$-18\leq h\leq 12$, $-15\leq k\leq 18$, $-29\leq l\leq 29$	
Reflections collected	46689	
Independent reflections	3175 (R(int) = 0.0918)	
Observed reflections	1961 ($I > 2\sigma(I)$)	
Absorption correction	Semi-empirical from equivalents	
Max. and min. transmission	0.99 and 0.70	
Refinement method	Full-matrix least-squares on F^2	
Data/restraints/parameters	3175 / 595 / 296	
Goodness-of-fit on F^2	1.46	
Final R indices ($I>2\sigma(I)$)	R1 = 0.091, wR2 = 0.223	
Largest diff. peak and hole	0.34 and -0.22 eÅ^{-3}	

9. Computational Details

9.1 General Remarks

The Gaussian09 program package was employed for quantum chemical calculations with a Kohn-Scham density functional methodologies theoretical approach.[S6] Geometries obtained after UFF-optimization using Avogadro, were visualized in GaussView 6 and optimized using PM6[S7] and afterwards B3LYP as functional.[S8] For this, either the triple-ζ basis-set 6-311G(d,p)[S9] or the doubleζ- basis-set 631G(d,p-) was used.[S10] After optimization of the regarded species, ground states were confirmed by using frequency calculations to not exhibit any imaginary frequency. Transition states were confirmed when only one virtual frequency was obtained, that represented the reaction coordinate. Geometries optimized with the intent of comparing energies and calculating energy barriers were calculated including the GD3BJ empirical dispersion[S11] and the SMD solvent model using either chloroform, dichloromethane or o-DCB as solvent.[S12] By using GoodVibes, the Grimme correction was applied to the energies.[S13] NICS(0) and NICS(1) values were calculated by adding a ghost atom in the centroid or 1 Å above/below of the corresponding ring of the optimized geometries and performing a single-point calculation based on Hartree-Fock methods[S14] using the augmented double-ζ-basis 6-31+G(d)[S10, S15] with the GIAO method.[S16] Ring-current analysis was accomplished by performing a single-point calculation with the CSGT method[S16a, S17] on the HF/6-31+G(d) level, orbital analysis using Multiwfn[S18] and visualized using the AICD program package of the Herges group.[S19] To calculate optical properties, excited state calculations are performed using the TD-DFT method[S20] and either B3LYP or PBE[S21] as functionals and either 6-31G(d,p) or 6-311G(d,p) as basis-sets. Visualization was performed by converting the calculation results into spectral data by using GaussSum[S22] and plotted using Origin. For the correct citation of literature for basis-sets, Basis Set Exchange was used.[S23]

9.2 Coordinates – Ground States

9.2.1 *C*$_2$-2

C	-6.12790	0.49310	-0.52500
C	-6.12760	-0.49800	0.52430
C	-4.85840	-1.03320	1.02130
C	-3.65320	-0.54600	0.46610
C	-3.65350	0.54290	-0.46650
C	-4.85910	1.02930	-1.02190
C	-2.43080	1.15660	-0.81590
C	-2.43100	2.12930	-1.83040
C	-3.60340	2.55390	-2.44800
C	-4.81860	2.01450	-2.00240
C	-4.81730	-2.01840	2.00200
C	-3.60180	-2.55660	2.44790
C	-2.42960	-2.13120	1.83040
C	-2.43010	-1.15870	0.81580
C	-1.19210	-0.66750	0.22010
C	-1.19260	0.66630	-0.22010
N	-7.25950	0.95540	-1.02940
C	-8.42010	0.48120	-0.52660

C	-8.41980	-0.48790	0.52560
N	-7.25890	-0.96120	1.02860
C	-9.65660	0.94880	-1.03930
C	-10.83610	0.47480	-0.52380
C	-10.83580	-0.48340	0.52250
C	-9.65610	-0.95650	1.03810
C	3.74730	4.92500	0.74150
C	2.54900	5.67370	0.46890
C	1.26940	4.97200	0.36070
C	1.25870	3.55540	0.29140
C	2.46030	2.81160	0.54490
C	3.66780	3.47240	0.88540
C	2.40570	1.40250	0.62370
C	3.47610	0.72650	1.23760
C	4.64210	1.37120	1.62990
C	4.73870	2.74930	1.39580
C	0.07520	5.67920	0.40130
C	-1.15510	5.01460	0.45770
C	-1.15480	3.62980	0.34840
C	0.02020	2.87530	0.16880
C	0.02270	1.42390	-0.03680
C	1.22060	0.71060	0.13530
N	4.91560	5.52670	0.88980
C	4.95480	6.87120	0.76220
C	3.76430	7.61550	0.48810
N	2.57370	6.99080	0.35360
C	6.18680	7.55930	0.90170
C	6.22880	8.92440	0.77290
C	5.04830	9.66240	0.50030
C	3.84140	9.02560	0.36040
C	2.55420	-5.67170	-0.46880
C	3.75180	-4.92180	-0.74160
C	3.67090	-3.46930	-0.88550
C	2.46280	-2.80960	-0.54490
C	1.26190	-3.55450	-0.29130
C	1.27390	-4.97110	-0.36060
C	0.02270	-2.87560	-0.16850
C	-1.15150	-3.63130	-0.34790
C	-1.15050	-5.01600	-0.45720
C	0.08040	-5.67940	-0.40100
C	4.74110	-2.74520	-1.39600
C	4.64310	-1.36710	-1.63000
C	3.47660	-0.72360	-1.23760
C	2.40690	-1.40050	-0.62360
C	1.22120	-0.70980	-0.13510
C	0.02390	-1.42410	0.03700
N	2.58010	-6.98870	-0.35360
C	3.77130	-7.61230	-0.48810
C	4.96110	-6.86690	-0.76230
N	4.92070	-5.52240	-0.88990
C	3.84970	-9.02230	-0.36050
C	5.05720	-9.65800	-0.50040
C	6.23700	-8.91890	-0.77310
C	6.19370	-7.55390	-0.90190
C	-3.59650	3.56010	-3.60590
C	-4.61340	4.68910	-3.33900

C	-3.99550	2.81510	-4.89780
C	-2.21190	4.19550	-3.81110
C	-3.59430	-3.56270	3.60600
C	-4.61030	-4.69240	3.33900
C	-3.99420	-2.81780	4.89760
C	-2.20930	-4.19700	3.81150
C	5.77660	0.62970	2.34770
C	5.63950	-0.89700	2.21700
C	5.72870	1.00830	3.84360
C	7.14320	1.04650	1.76750
C	-2.44280	5.81890	0.67250
C	-2.35590	6.54870	2.02980
C	-3.69320	4.92530	0.68120
C	-2.58770	6.86070	-0.45610
C	-2.43740	-5.82180	-0.67150
C	-2.35020	-6.55160	-2.02870
C	-3.68870	-4.92940	-0.68000
C	-2.58080	-6.86350	0.45730
C	5.77700	-0.62470	-2.34780
C	5.73000	-1.00390	-3.84360
C	7.14380	-1.03980	-1.76710
C	5.63820	0.90190	-2.21770
H	-1.48150	2.54470	-2.13020
H	-5.76180	2.34300	-2.41560
H	-5.76040	-2.34760	2.41490
H	-1.47980	-2.54590	2.13030
H	-9.62900	1.68130	-1.83640
H	-11.78110	0.83260	-0.91520
H	-11.78060	-0.84190	0.91370
H	-9.62790	-1.68900	1.83520
H	3.37670	-0.33320	1.40130
H	5.63120	3.30210	1.65190
H	0.13640	6.75710	0.45890
H	-2.09150	3.10900	0.41590
H	7.07290	6.97220	1.10860
H	7.17040	9.45000	0.87930
H	5.10690	10.74000	0.40260
H	2.92580	9.56500	0.15190
H	-2.08880	-3.11150	-0.41520
H	0.14270	-6.75730	-0.45880
H	5.63410	-3.29720	-1.65210
H	3.37630	0.33610	-1.40130
H	2.93460	-9.56260	-0.15190
H	5.11680	-10.73560	-0.40280
H	7.17920	-9.44360	-0.87950
H	7.07930	-6.96590	-1.10890
H	-4.37760	5.22190	-2.41610
H	-4.59410	5.40900	-4.16170
H	-5.63290	4.30910	-3.25610
H	-3.28890	2.00970	-5.11350
H	-4.00240	3.50360	-5.74810
H	-4.99090	2.37430	-4.80830
H	-2.26520	4.93910	-4.60990
H	-1.85810	4.69740	-2.90720
H	-1.46420	3.45420	-4.10310
H	-4.37400	-5.22540	2.41630

H	-4.59080	-5.41220	4.16200
H	-5.63010	-4.31320	3.25580
H	-3.28830	-2.01180	5.11340
H	-4.00060	-3.50610	5.74810
H	-4.99000	-2.37780	4.80780
H	-2.26220	-4.94060	4.61030
H	-1.85480	-4.69860	2.90770
H	-1.46230	-3.45500	4.10380
H	5.59270	-1.21260	1.17260
H	6.50140	-1.38330	2.68020
H	4.74620	-1.27020	2.72320
H	5.85020	2.08550	3.98060
H	6.52830	0.50100	4.39190
H	4.77190	0.71850	4.28520
H	7.94130	0.47690	2.25090
H	7.19130	0.85570	0.69390
H	7.35130	2.10540	1.92920
H	-1.50430	7.23130	2.06470
H	-3.26470	7.13170	2.20530
H	-2.24590	5.83130	2.84700
H	-3.80600	4.36740	-0.24990
H	-4.58420	5.54460	0.81030
H	-3.66960	4.20770	1.50530
H	-1.75010	7.56100	-0.46870
H	-2.63140	6.37480	-1.43290
H	-3.50630	7.43880	-0.32100
H	-1.49800	-7.23340	-2.06400
H	-3.25860	-7.13560	-2.20370
H	-2.24140	-5.83430	-2.84610
H	-3.80190	-4.37160	0.25110
H	-4.57920	-5.54950	-0.80880
H	-3.66600	-4.21180	-1.50410
H	-3.49890	-7.44250	0.32260
H	-1.74250	-7.56290	0.46970
H	-2.62470	-6.37750	1.43410
H	4.77300	-0.71520	-4.28550
H	6.52910	-0.49580	-4.39180
H	5.85260	-2.08090	-3.98030
H	7.19150	-0.84850	-0.69350
H	7.94150	-0.46960	-2.25050
H	7.35310	-2.09860	-1.92830
H	6.49960	1.38900	-2.68100
H	4.74450	1.27400	-2.72410
H	5.59100	1.21790	-1.17340

9.2.2 D_3-2

C	-5.53680	-2.84110	0.33860
C	-5.99920	-1.65530	-0.33690
C	-5.05590	-0.57980	-0.64570
C	-3.69610	-0.73230	-0.28110
C	-3.21630	-1.96310	0.28010
C	-4.11420	-2.99520	0.64550
C	-1.82880	-2.15480	0.46110
C	-1.40170	-3.30190	1.15310
C	-2.27770	-4.30120	1.55980
C	-3.63780	-4.14380	1.26710

C	-5.48230	0.58780	-1.26820
C	-4.58740	1.62350	-1.56240
C	-3.26610	1.48080	-1.15570
C	-2.80430	0.34770	-0.46290
C	-1.40070	0.20480	-0.06380
C	-0.89220	-1.09950	0.06240
N	-6.37260	-3.81210	0.66760
C	-7.67560	-3.66530	0.34430
C	-8.13220	-2.49420	-0.33840
N	-7.27210	-1.50530	-0.66390
C	-8.60680	-4.68130	0.67890
C	-9.92930	-4.53640	0.34540
C	-10.38170	-3.37610	-0.33450
C	-9.50580	-2.37540	-0.67050
C	5.23010	-3.37280	0.33600
C	4.43410	-4.36660	-0.33830
C	3.03030	-4.08860	-0.64490
C	2.48220	-2.83500	-0.28010
C	3.30820	-1.80360	0.28020
C	4.65150	-2.06440	0.64440
C	2.78000	-0.50640	0.46180
C	3.55970	0.43710	1.15410
C	4.86340	0.17850	1.56030
C	5.40780	-1.07740	1.26620
C	2.23200	-5.04270	-1.26550
C	0.88700	-4.78660	-1.55760
C	0.34990	-3.57080	-1.15150
C	1.10070	-2.60320	-0.46050
C	0.52260	-1.31600	-0.06250
C	1.39780	-0.22330	0.06300
N	6.48980	-3.60990	0.66250
C	7.01480	-4.81100	0.33760
C	6.22860	-5.79260	-0.34380
N	4.94120	-5.54340	-0.66670
C	8.36130	-5.10810	0.66910
C	8.89770	-6.32510	0.33400
C	8.11870	-7.29770	-0.34450
C	6.81320	-7.04080	-0.67750
C	0.30680	6.21530	0.33720
C	1.56640	6.02270	-0.33530
C	2.02690	4.66800	-0.64270
C	1.21440	3.56660	-0.27950
C	-0.09250	3.76650	0.27930
C	-0.53820	5.06030	0.64330
C	-0.95240	2.66070	0.45940
C	-2.16050	2.86450	1.14950
C	-2.58880	4.12300	1.55480
C	-1.77220	5.22210	1.26280
C	3.25260	4.45350	-1.26270
C	3.70260	3.16070	-1.55610
C	2.91750	2.08770	-1.15110
C	1.70400	2.25430	-0.46040
C	0.87790	1.11010	-0.06270
C	-0.50610	1.32190	0.06170
N	-0.11740	7.42480	0.66430
C	0.66160	8.47980	0.34190

C	1.90580	8.28950	-0.33770
N	2.33320	7.05000	-0.66130
C	0.24600	9.79430	0.67430
C	1.03320	10.86720	0.34170
C	2.26600	10.67870	-0.33490
C	2.69590	9.41970	-0.66870
C	-1.79810	-5.53950	2.32690
C	-2.34370	-6.81550	1.65270
C	-2.33230	-5.46100	3.77250
C	-0.26390	-5.63530	2.36990
C	-5.07190	2.85860	-2.33150
C	-6.33830	3.42900	-1.65990
C	-5.40980	2.43720	-3.77710
C	-4.00770	3.96770	-2.37440
C	5.69560	1.21260	2.32840
C	5.89430	0.70950	3.77370
C	5.01110	2.58880	2.37230
C	7.07350	1.37890	1.65480
C	0.05860	-5.82550	-2.32340
C	0.59070	-5.91110	-3.76940
C	-1.43410	-5.45840	-2.36520
C	0.19850	-7.20580	-1.64850
C	-3.90230	4.32710	2.31970
C	-3.56970	4.75170	3.76550
C	-4.75210	3.04620	2.36220
C	-4.73360	5.43680	1.64310
C	5.01620	2.96280	-2.32230
C	6.14200	3.77350	-1.64740
C	4.82380	3.46720	-3.76800
C	5.44420	1.48650	-2.36520
H	-0.35290	-3.40000	1.37490
H	-4.36160	-4.90140	1.53250
H	-6.52810	0.65590	-1.53290
H	-2.56020	2.26240	-1.37820
H	-8.23540	-5.55700	1.19650
H	-10.64080	-5.31280	0.60070
H	-11.43140	-3.28520	-0.58790
H	-9.82530	-1.47950	-1.18810
H	3.11980	1.39400	1.37660
H	6.42620	-1.32490	1.53040
H	2.69610	-5.98220	-1.53040
H	-0.68030	-3.35110	-1.37300
H	8.93430	-4.34820	1.18580
H	9.92670	-6.55200	0.58700
H	8.56530	-8.25160	-0.59920
H	6.19670	-7.76600	-1.19410
H	-2.76990	2.00530	1.37110
H	-2.06710	6.22780	1.52710
H	3.83480	5.32510	-1.52630
H	3.24190	1.08570	-1.37340
H	-0.69940	9.91070	1.18950
H	0.71550	11.87170	0.59540
H	2.86990	11.54220	-0.58760
H	3.63290	9.24820	-1.18390
H	-2.01620	-6.87970	0.61300
H	-1.98030	-7.70170	2.18010

H	-3.43450	-6.84770	1.66260
H	-1.95600	-4.56640	4.27520
H	-2.01300	-6.33680	4.34540
H	-3.42380	-5.42240	3.78950
H	0.03060	-6.55650	2.87820
H	0.17050	-5.65000	1.36740
H	0.18020	-4.80230	2.92020
H	-6.14240	3.69930	-0.62030
H	-6.67000	4.32660	-2.18900
H	-7.16300	2.71430	-1.67030
H	-4.52660	2.03280	-4.27800
H	-5.76700	3.29700	-4.35170
H	-6.18700	1.66960	-3.79400
H	-4.41420	4.84470	-2.88390
H	-3.69880	4.27350	-1.37190
H	-3.11640	3.65500	-2.92380
H	6.40700	-0.25490	3.78990
H	6.49250	1.42360	4.34740
H	4.93130	0.58720	4.27590
H	4.80670	2.97330	1.37010
H	5.66130	3.30430	2.88150
H	4.06750	2.55620	2.92250
H	7.65910	2.13630	2.18320
H	7.64700	0.45040	1.66400
H	6.96570	1.69570	0.61540
H	1.64390	-6.20030	-3.78700
H	0.02390	-6.65180	-4.34130
H	0.49840	-4.94520	-4.27270
H	-1.85210	-5.34180	-1.36240
H	-1.99100	-6.24990	-2.87240
H	-1.60960	-4.53130	-2.91620
H	1.22980	-7.56260	-1.65920
H	-0.13210	-7.16860	-0.60850
H	-0.41370	-7.94320	-2.17490
H	-2.99080	5.67790	3.78270
H	-4.48870	4.91320	4.33670
H	-2.98370	3.97910	4.26980
H	-4.98020	2.67650	1.35950
H	-5.69810	3.25190	2.86870
H	-4.25360	2.24560	2.91400
H	-5.68350	5.56570	2.16910
H	-4.21610	6.39770	1.65280
H	-4.95150	5.18420	0.60340
H	6.27550	3.46790	-0.60770
H	7.08650	3.61220	-2.17430
H	5.93560	4.84510	-1.65740
H	4.03310	2.90510	-4.27120
H	5.74840	3.34680	-4.34040
H	4.54800	4.52410	-3.78480
H	6.40800	1.40000	-2.87260
H	5.55220	1.06550	-1.36270
H	4.72870	0.87150	-2.91640

9.2.3 C_2-3

C	-3.83910	4.87181	-1.04102
C	-2.55008	5.69225	-0.78137

C	-1.27828	4.93943	-0.66433
C	-1.28252	3.53089	-0.51146
C	-2.49164	2.77097	-0.71112
C	-3.70360	3.39781	-1.09278
C	-2.43422	1.36325	-0.70231
C	-3.50736	0.64621	-1.27749
C	-4.67341	1.25477	-1.71271
C	-4.77015	2.64813	-1.55887
C	-0.08812	5.63748	-0.76702
C	1.14384	4.96546	-0.80590
C	1.13115	3.59434	-0.60757
C	-0.04906	2.85630	-0.35987
C	-0.04893	1.42276	-0.06043
C	-1.24819	0.69990	-0.17970
O	-2.61405	6.89988	-0.72528
O	-4.88775	5.45102	-1.21683
C	-2.54996	-5.69229	0.78123
C	-3.83898	-4.87188	1.04102
C	-3.70352	-3.39788	1.09279
C	-2.49158	-2.77101	0.71110
C	-1.28244	-3.53090	0.51142
C	-1.27817	-4.93944	0.66426
C	-0.04900	-2.85628	0.35983
C	1.13123	-3.59430	0.60755
C	1.14394	-4.96542	0.80587
C	-0.08801	-5.63747	0.76695
C	-4.77007	-2.64823	1.55891
C	-4.67335	-1.25487	1.71275
C	-3.50733	-0.64628	1.27751
C	-2.43419	-1.36329	0.70230
C	-1.24817	-0.69991	0.17968
C	-0.04890	-1.42275	0.06041
O	-4.88762	-5.45112	1.21681
O	-2.61389	-6.89993	0.72526
C	6.13317	0.54405	0.55722
C	6.13319	-0.54405	-0.55713
C	4.82231	-1.10152	-0.97132
C	3.62776	-0.57910	-0.42831
C	3.62775	0.57914	0.42831
C	4.82229	1.10155	0.97135
C	2.40639	1.21477	0.72416
C	2.40122	2.26234	1.66997
C	3.56155	2.73094	2.26990
C	4.78011	2.14967	1.87650
C	4.78014	-2.14964	-1.87647
C	3.56159	-2.73087	-2.26993
C	2.40125	-2.26226	-1.67003
C	2.40640	-1.21472	-0.72420
C	1.16693	-0.68113	-0.17053
C	1.16692	0.68118	0.17050
O	7.18854	-0.91287	-1.02065
O	7.18852	0.91292	1.02071
C	-5.80303	0.47043	-2.38860
C	-5.66167	-1.04498	-2.16726
C	-5.75014	0.76257	-3.90376
C	-7.17112	0.91953	-1.83645

C	2.42922	5.74551	-1.09935
C	2.59064	6.87221	-0.05773
C	2.32210	6.36632	-2.50861
C	3.67593	4.84833	-1.05397
C	2.42934	-5.74543	1.09936
C	3.67604	-4.84826	1.05383
C	2.59073	-6.87226	0.05787
C	2.32226	-6.36607	2.50870
C	-5.80298	-0.47056	2.38867
C	-5.75001	-0.76265	3.90383
C	-7.17108	-0.91974	1.83660
C	-5.66170	1.04485	2.16729
C	3.54721	3.81779	3.35030
C	2.16207	4.46771	3.49543
C	4.57026	4.92041	3.00694
C	3.93520	3.16743	4.69567
C	3.54724	-3.81770	-3.35035
C	4.56989	-4.92062	-3.00674
C	3.93576	-3.16743	-4.69560
C	2.16196	-4.46725	-3.49584
H	-3.39969	-0.42063	-1.38168
H	-5.66355	3.18745	-1.84404
H	-0.15112	6.71170	-0.88347
H	2.06214	3.06072	-0.66203
H	2.06220	-3.06066	0.66202
H	-0.15097	-6.71170	0.88336
H	-5.66345	-3.18757	1.84410
H	-3.39968	0.42057	1.38169
H	1.44826	2.69395	1.93483
H	5.72385	2.49588	2.27709
H	5.72389	-2.49585	-2.27704
H	1.44828	-2.69385	-1.93492
H	-5.62336	-1.29763	-1.10534
H	-6.51853	-1.56070	-2.60634
H	-4.76426	-1.44540	-2.64482
H	-5.87140	1.82973	-4.10437
H	-6.54925	0.22534	-4.42249
H	-4.79322	0.44614	-4.32645
H	-7.96636	0.31815	-2.28385
H	-7.22103	0.79654	-0.75305
H	-7.38381	1.96492	-2.06580
H	2.64322	6.46602	0.95439
H	3.51046	7.43134	-0.24881
H	1.75899	7.57857	-0.09199
H	2.20215	5.58798	-3.26638
H	3.22767	6.93358	-2.74077
H	1.47040	7.04549	-2.58507
H	4.56725	5.45162	-1.24021
H	3.79770	4.36466	-0.08297
H	3.64163	4.07003	-1.82070
H	3.64174	-4.06983	1.82042
H	4.56736	-5.45152	1.24018
H	3.79782	-4.36476	0.08275
H	1.75905	-7.57859	0.09220
H	3.51052	-7.43140	0.24905
H	2.64336	-6.46619	-0.95429

H	3.22784	-6.93327	2.74092
H	2.20229	-5.58764	3.26638
H	1.47058	-7.04526	2.58526
H	-4.79308	-0.44616	4.32647
H	-6.54911	-0.22544	4.42259
H	-5.87120	-1.82981	4.10448
H	-7.22104	-0.79676	0.75320
H	-7.96633	-0.31839	2.28404
H	-7.38369	-1.96513	2.06597
H	-4.76427	1.44533	2.64477
H	-5.62347	1.29748	1.10535
H	-6.51856	1.56054	2.60640
H	1.40985	3.75101	3.83404
H	2.20925	5.26669	4.23858
H	1.81826	4.90486	2.55458
H	5.58990	4.53407	2.96420
H	4.54524	5.70039	3.77222
H	4.34511	5.38277	2.04420
H	3.22387	2.38325	4.96680
H	4.92985	2.71848	4.64741
H	3.93967	3.91727	5.49174
H	4.34438	-5.38291	-2.04405
H	4.54483	-5.70060	-3.77202
H	5.58964	-4.53458	-2.96377
H	3.22470	-2.38306	-4.96690
H	3.94024	-3.91725	-5.49168
H	4.93051	-2.71874	-4.64707
H	2.20913	-5.26630	-4.23893
H	1.81774	-4.90423	-2.55507
H	1.41005	-3.75036	-3.83474

9.2.4 *D_3*-3

C	-0.73642	-6.21464	0.39597
C	-2.03998	-5.91644	-0.39244
C	-2.36495	-4.49620	-0.66737
C	-1.47389	-3.46733	-0.28147
C	-0.18077	-3.76318	0.28408
C	0.17414	-5.07712	0.67017
C	0.75401	-2.72386	0.45772
C	1.94916	-3.01200	1.15294
C	2.28952	-4.28852	1.57251
C	1.38648	-5.32647	1.29191
C	-3.56465	-4.19355	-1.28995
C	-3.92589	-2.86626	-1.57173
C	-3.06444	-1.86471	-1.15195
C	-1.86346	-2.12495	-0.45580
C	-0.95698	-1.04353	-0.06095
C	0.40781	-1.35577	0.06314
O	-2.74910	-6.83735	-0.73143
O	-0.49863	-7.35213	0.73571
C	5.74942	2.46922	0.39746
C	6.14280	1.19216	-0.39266
C	5.07531	0.20094	-0.66873
C	3.73893	0.45754	-0.28195
C	3.34868	1.72459	0.28534
C	4.30908	2.68856	0.67242

C	1.98121	2.01424	0.45927
C	1.63304	3.19245	1.15596
C	2.56838	4.12483	1.57706
C	3.91881	3.86219	1.29594
C	5.41288	-0.98844	-1.29316
C	4.44404	-1.96470	-1.57569
C	3.14613	-1.72026	-1.15469
C	2.77123	-0.55098	-0.45691
C	1.38160	-0.30720	-0.06124
C	0.96960	1.03076	0.06356
O	7.29469	1.03929	-0.73271
O	6.61559	3.24393	0.73718
C	-5.01494	3.74403	0.38709
C	-4.10436	4.72279	-0.40209
C	-2.71125	4.29441	-0.67412
C	-2.26593	3.00892	-0.28593
C	-3.16898	2.03765	0.28037
C	-4.48464	2.38762	0.66496
C	-2.73599	0.70893	0.45700
C	-3.58263	-0.18056	1.15470
C	-4.85833	0.16377	1.57375
C	-5.30641	1.46367	1.28905
C	-1.84850	5.18184	-1.29598
C	-0.51772	4.83129	-1.57473
C	-0.08157	3.58490	-1.15331
C	-0.90826	2.67530	-0.45775
C	-0.42526	1.34989	-0.06147
C	-1.37809	0.32415	0.06296
O	-4.54740	5.79655	-0.74357
O	-6.11971	4.10682	0.72430
C	3.58375	-4.58206	2.33825
C	4.52215	-3.36479	2.37328
C	4.32980	-5.75281	1.66472
C	3.22074	-4.97494	3.78589
C	-5.21782	-2.56804	-2.33942
C	-6.40003	-3.29653	-1.66677
C	-5.06000	-3.08103	-3.78635
C	-5.53255	-1.06368	-2.37600
C	2.17554	5.39104	2.34513
C	0.65214	5.59432	2.38171
C	2.81537	6.62390	1.67320
C	2.69821	5.27082	3.79224
C	4.83151	-3.23167	-2.34518
C	6.05324	-3.89241	-1.67326
C	5.19711	-2.83660	-3.79154
C	3.68589	-4.25612	-2.38352
C	-5.75862	-0.80774	2.34385
C	-5.17140	-2.22790	2.38703
C	-7.14497	-0.87416	1.66976
C	-5.91915	-0.28953	3.78866
C	0.38820	5.80049	-2.34118
C	0.34867	7.18846	-1.66833
C	-0.13308	5.92098	-3.78874
C	1.84810	5.31979	-2.37614
H	2.61505	-2.19496	1.37296
H	1.59823	-6.35060	1.57012

H	-4.20064	-5.02374	-1.56817
H	-3.30833	-0.83938	-1.37255
H	0.59236	3.36019	1.37575
H	4.69992	4.55723	1.57498
H	6.44974	-1.12360	-1.57200
H	2.38003	-2.44388	-1.37578
H	-3.20757	-1.16503	1.37744
H	-6.29934	1.79282	1.56621
H	-2.24929	6.14729	-1.57594
H	0.92863	3.28347	-1.37223
H	4.08193	-2.52646	2.91891
H	5.44991	-3.63358	2.88316
H	4.77926	-3.02101	1.36825
H	3.74507	-6.67408	1.68122
H	5.26858	-5.94759	2.18960
H	4.56388	-5.52243	0.62331
H	4.12611	-5.20201	4.35572
H	2.69496	-4.15926	4.28862
H	2.57613	-5.85653	3.80896
H	-6.51216	-2.98544	-0.62610
H	-7.32918	-3.06431	-2.19355
H	-6.27430	-4.38047	-1.68127
H	-4.23097	-2.57592	-4.28842
H	-5.97319	-2.89205	-4.35762
H	-4.86318	-4.15528	-3.80813
H	-6.48438	-0.90200	-2.88671
H	-5.61491	-0.64155	-1.37128
H	-4.77073	-0.50114	-2.92145
H	0.14709	4.79236	2.92588
H	0.42102	6.53089	2.89397
H	0.22503	5.64731	1.37715
H	3.90562	6.57864	1.68888
H	2.51450	7.53332	2.19969
H	2.49804	6.71280	0.63217
H	2.44181	6.16716	4.36391
H	2.25560	4.40636	4.29355
H	3.78409	5.15414	3.81459
H	5.83956	-4.14681	-0.63308
H	6.31683	-4.81233	-2.20148
H	6.92909	-3.24151	-1.68635
H	4.34537	-2.36997	-4.29285
H	5.48944	-3.72130	-4.36411
H	6.02945	-2.12949	-3.81246
H	2.81806	-3.87657	-2.92864
H	4.02175	-5.16059	-2.89548
H	3.36119	-4.53989	-1.37929
H	-4.22554	-2.26101	2.93347
H	-5.86718	-2.89527	2.90030
H	-5.00093	-2.62780	1.38433
H	-7.65175	0.09230	1.68038
H	-7.78243	-1.58767	2.19824
H	-7.06120	-1.19784	0.63025
H	-4.95012	-0.23684	4.29139
H	-6.36287	0.70849	3.80619
H	-6.56696	-0.95862	4.36193
H	0.67300	7.12971	-0.62730

H	1.01537	7.87667	-2.19427
H	-0.65259	7.62223	-1.68377
H	-0.11064	4.95041	-4.29074
H	0.48871	6.61659	-4.35926
H	-1.16138	6.28884	-3.81187
H	2.46509	6.06263	-2.88650
H	2.25377	5.18010	-1.37099
H	1.95423	4.37848	-2.92113

9.2.5 *(P,P,P)-C*$_2$-4

C	-6.12790	0.49310	-0.52500
C	-6.12760	-0.49800	0.52430
C	-4.85840	-1.03320	1.02130
C	-3.65320	-0.54600	0.46610
C	-3.65350	0.54290	-0.46650
C	-4.85910	1.02930	-1.02190
C	-2.43080	1.15660	-0.81590
C	-2.43100	2.12930	-1.83040
C	-3.60340	2.55390	-2.44800
C	-4.81860	2.01450	-2.00240
C	-4.81730	-2.01840	2.00200
C	-3.60180	-2.55660	2.44790
C	-2.42960	-2.13120	1.83040
C	-2.43010	-1.15870	0.81580
C	-1.19210	-0.66750	0.22010
C	-1.19260	0.66630	-0.22010
N	-7.25950	0.95540	-1.02940
C	-8.42010	0.48120	-0.52660
C	-8.41980	-0.48790	0.52560
N	-7.25890	-0.96120	1.02860
C	-9.65660	0.94880	-1.03930
C	-10.83610	0.47480	-0.52380
C	-10.83580	-0.48340	0.52250
C	-9.65610	-0.95650	1.03810
C	3.74730	4.92500	0.74150
C	2.54900	5.67370	0.46890
C	1.26940	4.97200	0.36070
C	1.25870	3.55540	0.29140
C	2.46030	2.81160	0.54490
C	3.66780	3.47240	0.88540
C	2.40570	1.40250	0.62370
C	3.47610	0.72650	1.23760
C	4.64210	1.37120	1.62990
C	4.73870	2.74930	1.39580
C	0.07520	5.67920	0.40130
C	-1.15510	5.01460	0.45770
C	-1.15480	3.62980	0.34840
C	0.02020	2.87530	0.16880
C	0.02270	1.42390	-0.03680
C	1.22060	0.71060	0.13530
N	4.91560	5.52670	0.88980
C	4.95480	6.87120	0.76220
C	3.76430	7.61550	0.48810
N	2.57370	6.99080	0.35360
C	6.18680	7.55930	0.90170
C	6.22880	8.92440	0.77290

C	5.04830	9.66240	0.50030
C	3.84140	9.02560	0.36040
C	2.55420	-5.67170	-0.46880
C	3.75180	-4.92180	-0.74160
C	3.67090	-3.46930	-0.88550
C	2.46280	-2.80960	-0.54490
C	1.26190	-3.55450	-0.29130
C	1.27390	-4.97110	-0.36060
C	0.02270	-2.87560	-0.16850
C	-1.15150	-3.63130	-0.34790
C	-1.15050	-5.01600	-0.45720
C	0.08040	-5.67940	-0.40100
C	4.74110	-2.74520	-1.39600
C	4.64310	-1.36710	-1.63000
C	3.47660	-0.72360	-1.23760
C	2.40690	-1.40050	-0.62360
C	1.22120	-0.70980	-0.13510
C	0.02390	-1.42410	0.03700
N	2.58010	-6.98870	-0.35360
C	3.77130	-7.61230	-0.48810
C	4.96110	-6.86690	-0.76230
N	4.92070	-5.52240	-0.88990
C	3.84970	-9.02230	-0.36050
C	5.05720	-9.65800	-0.50040
C	6.23700	-8.91890	-0.77310
C	6.19370	-7.55390	-0.90190
C	-3.59650	3.56010	-3.60590
C	-4.61340	4.68910	-3.33900
C	-3.99550	2.81510	-4.89780
C	-2.21190	4.19550	-3.81110
C	-3.59430	-3.56270	3.60600
C	-4.61030	-4.69240	3.33900
C	-3.99420	-2.81780	4.89760
C	-2.20930	-4.19700	3.81150
C	5.77660	0.62970	2.34770
C	5.63950	-0.89700	2.21700
C	5.72870	1.00830	3.84360
C	7.14320	1.04650	1.76750
C	-2.44280	5.81890	0.67250
C	-2.35590	6.54870	2.02980
C	-3.69320	4.92530	0.68120
C	-2.58770	6.86070	-0.45610
C	-2.43740	-5.82180	-0.67150
C	-2.35020	-6.55160	-2.02870
C	-3.68870	-4.92940	-0.68000
C	-2.58080	-6.86350	0.45730
C	5.77700	-0.62470	-2.34780
C	5.73000	-1.00390	-3.84360
C	7.14380	-1.03980	-1.76710
C	5.63820	0.90190	-2.21770
H	-1.48150	2.54470	-2.13020
H	-5.76180	2.34300	-2.41560
H	-5.76040	-2.34760	2.41490
H	-1.47980	-2.54590	2.13030
H	-9.62900	1.68130	-1.83640
H	-11.78110	0.83260	-0.91520

H	-11.78060	-0.84190	0.91370
H	-9.62790	-1.68900	1.83520
H	3.37670	-0.33320	1.40130
H	5.63120	3.30210	1.65190
H	0.13640	6.75710	0.45890
H	-2.09150	3.10900	0.41590
H	7.07290	6.97220	1.10860
H	7.17040	9.45000	0.87930
H	5.10690	10.74000	0.40260
H	2.92580	9.56500	0.15190
H	-2.08880	-3.11150	-0.41520
H	0.14270	-6.75730	-0.45880
H	5.63410	-3.29720	-1.65210
H	3.37630	0.33610	-1.40130
H	2.93460	-9.56260	-0.15190
H	5.11680	-10.73560	-0.40280
H	7.17920	-9.44360	-0.87950
H	7.07930	-6.96590	-1.10890
H	-4.37760	5.22190	-2.41610
H	-4.59410	5.40900	-4.16170
H	-5.63290	4.30910	-3.25610
H	-3.28890	2.00970	-5.11350
H	-4.00240	3.50360	-5.74810
H	-4.99090	2.37430	-4.80830
H	-2.26520	4.93910	-4.60990
H	-1.85810	4.69740	-2.90720
H	-1.46420	3.45420	-4.10310
H	-4.37400	-5.22540	2.41630
H	-4.59080	-5.41220	4.16200
H	-5.63010	-4.31320	3.25580
H	-3.28830	-2.01180	5.11340
H	-4.00060	-3.50610	5.74810
H	-4.99000	-2.37780	4.80780
H	-2.26220	-4.94060	4.61030
H	-1.85480	-4.69860	2.90770
H	-1.46230	-3.45500	4.10380
H	5.59270	-1.21260	1.17260
H	6.50140	-1.38330	2.68020
H	4.74620	-1.27020	2.72320
H	5.85020	2.08550	3.98060
H	6.52830	0.50100	4.39190
H	4.77190	0.71850	4.28520
H	7.94130	0.47690	2.25090
H	7.19130	0.85570	0.69390
H	7.35130	2.10540	1.92920
H	-1.50430	7.23130	2.06470
H	-3.26470	7.13170	2.20530
H	-2.24590	5.83130	2.84700
H	-3.80600	4.36740	-0.24990
H	-4.58420	5.54460	0.81030
H	-3.66960	4.20770	1.50530
H	-1.75010	7.56100	-0.46870
H	-2.63140	6.37480	-1.43290
H	-3.50630	7.43880	-0.32100
H	-1.49800	-7.23340	-2.06400
H	-3.25860	-7.13560	-2.20370

H	-2.24140	-5.83430	-2.84610
H	-3.80190	-4.37160	0.25110
H	-4.57920	-5.54950	-0.80880
H	-3.66600	-4.21180	-1.50410
H	-3.49890	-7.44250	0.32260
H	-1.74250	-7.56290	0.46970
H	-2.62470	-6.37750	1.43410
H	4.77300	-0.71520	-4.28550
H	6.52910	-0.49580	-4.39180
H	5.85260	-2.08090	-3.98030
H	7.19150	-0.84850	-0.69350
H	7.94150	-0.46960	-2.25050
H	7.35310	-2.09860	-1.92830
H	6.49960	1.38900	-2.68100
H	4.74450	1.27400	-2.72410
H	5.59100	1.21790	-1.17340

***(M,M,M)-C*₂-4**

C	-3.750032	-4.922960	-0.741719
C	-2.552148	-5.672434	-0.468977
C	-1.272095	-4.971457	-0.360702
C	-1.260549	-3.554829	-0.291442
C	-2.461743	-2.810344	-0.544962
C	-3.669612	-3.470447	-0.885520
C	-2.406299	-1.401257	-0.623640
C	-3.476260	-0.724665	-1.237597
C	-4.642603	-1.368565	-1.629938
C	-4.740089	-2.746642	-1.395909
C	-0.078338	-5.679337	-0.401175
C	1.152317	-5.015514	-0.457498
C	1.152880	-3.630749	-0.348173
C	-0.021645	-2.875512	-0.168686
C	-0.023353	-1.424056	0.036904
C	-1.220888	-0.710144	-0.135179
N	-4.918669	-5.523983	-0.889989
C	-4.958660	-6.868493	-0.762451
C	-3.768591	-7.613493	-0.488242
N	-2.577608	-6.989522	-0.353674
C	-6.191010	-7.555895	-0.902020
C	-6.233850	-8.920901	-0.773243
C	-5.053804	-9.659626	-0.500518
C	-3.846537	-9.023524	-0.360572
C	-2.551252	5.672756	0.468932
C	-3.749264	4.923482	0.741677
C	-3.669077	3.470962	0.885552
C	-2.461307	2.810655	0.545053
C	-1.259991	3.554939	0.291526
C	-1.271308	4.971574	0.360717
C	-0.021196	2.875423	0.168823
C	1.153444	3.630473	0.348337
C	1.153108	5.015244	0.457581
C	-0.077436	5.679266	0.401170
C	-4.739677	2.747347	1.395955
C	-4.642404	1.369270	1.630065
C	-3.476154	0.725165	1.237771

C	-2.406086	1.401559	0.623788
C	-1.220783	0.710246	0.135346
C	-0.023138	1.423968	-0.036763
N	-2.576502	6.989843	0.353577
C	-3.767391	7.614007	0.488084
C	-4.957584	6.869206	0.762290
N	-4.917808	5.524694	0.889892
C	-3.845111	9.024045	0.360355
C	-5.052283	9.660342	0.500233
C	-6.232454	8.921815	0.772950
C	-6.189831	7.556809	0.901790
C	6.127859	-0.495997	0.524565
C	6.127898	0.495143	-0.524725
C	4.858859	1.030943	-1.021609
C	3.653468	0.544185	-0.466319
C	3.653420	-0.544775	0.466232
C	4.858776	-1.031666	1.021478
C	2.430480	-1.157961	0.815731
C	2.430300	-2.130578	1.830264
C	3.602539	-2.555713	2.447711
C	4.817990	-2.016969	2.002000
C	4.818144	2.016272	-2.002106
C	3.602737	2.555175	-2.447752
C	2.430476	2.130147	-1.830283
C	2.430589	1.157517	-0.815762
C	1.192490	0.666759	-0.220082
C	1.192410	-0.667034	0.220138
N	7.259227	-0.958888	1.028826
C	8.420006	-0.485107	0.525931
C	8.420045	0.484021	-0.526135
N	7.259304	0.957923	-1.029005
C	9.656423	-0.953372	1.038475
C	10.836050	-0.479799	0.522985
C	10.836088	0.478474	-0.523232
C	9.656499	0.952163	-1.038702
C	-5.776707	-0.626400	-2.347606
C	-7.143448	-1.042200	-1.767016
C	-5.729445	-1.005280	-3.843488
C	-5.638557	0.900208	-2.217198
C	2.439576	-5.820687	-0.671928
C	2.352517	-6.550632	-2.029139
C	2.583523	-6.862342	0.456909
C	3.690493	-4.927825	-0.680648
C	2.440508	5.820199	0.672004
C	3.691241	4.927088	0.681011
C	2.353447	6.550394	2.029087
C	2.584812	6.861619	-0.457000
C	-5.776629	0.627331	2.347767
C	-5.729206	1.006161	3.843658
C	-5.638822	-0.899305	2.217305
C	-7.143305	1.043456	1.767258
C	3.595307	-3.561870	3.605740
C	2.210434	-4.196704	3.811000
C	3.994687	-2.816907	4.897500
C	4.611777	-4.691226	3.338927
C	3.595596	3.561443	-3.605682

C	3.995109	2.816621	-4.897481
C	4.612035	4.690771	-3.338630
C	2.210737	4.196296	-3.811008
H	-3.376278	0.335066	-1.401237
H	-5.632925	-3.298932	-1.652027
H	-0.140295	-6.757229	-0.458822
H	2.089931	-3.110571	-0.415612
H	-7.076834	-6.968285	-1.108997
H	-7.175831	-9.445930	-0.879641
H	-5.113059	-10.737241	-0.402873
H	-2.931244	-9.563473	-0.151974
H	2.090408	3.110151	0.415819
H	-0.139222	6.757172	0.458740
H	-5.632432	3.299791	1.652034
H	-3.376328	-0.334569	1.401484
H	-2.929727	9.563842	0.151763
H	-5.111367	10.737962	0.402542
H	-7.174357	9.446995	0.879294
H	-7.075754	6.969346	1.108762
H	1.480633	-2.545605	2.130123
H	5.761135	-2.345851	2.414973
H	5.761311	2.345049	-2.415115
H	1.480849	2.545314	-2.130075
H	9.628496	-1.685909	1.835572
H	11.780942	-0.838025	0.914273
H	11.781009	0.836606	-0.914536
H	9.628630	1.684704	-1.835798
H	-7.191223	-0.851186	-0.693450
H	-7.941335	-0.472165	-2.250378
H	-7.352304	-2.101037	-1.928487
H	-4.772526	-0.716155	-4.285298
H	-6.528774	-0.497464	-4.391668
H	-5.851697	-2.082381	-3.980335
H	-6.500232	1.387038	-2.680307
H	-5.591442	1.216000	-1.172801
H	-4.745106	1.272775	-2.723567
H	2.243010	-5.833373	-2.846491
H	3.261165	-7.134038	-2.204332
H	1.500683	-7.232966	-2.064150
H	2.627640	-6.376262	1.433582
H	3.501632	-7.441246	0.321941
H	1.745388	-7.561874	0.469548
H	4.581149	-5.547626	-0.809732
H	3.667346	-4.210192	-1.504696
H	3.803760	-4.370062	0.250519
H	3.804448	4.369077	-0.250016
H	4.582013	5.546742	0.810004
H	3.667905	4.209655	1.505229
H	1.501730	7.232884	2.063881
H	3.262182	7.133665	2.204289
H	2.243718	5.833290	2.846544
H	3.503003	7.440384	-0.321999
H	2.628991	6.375347	-1.433578
H	1.746808	7.561302	-0.469903
H	-5.851195	2.083288	3.980537
H	-6.528629	0.498521	4.391862

H	-4.772335	0.716795	4.285414
H	-5.591860	-1.215077	1.172895
H	-6.500566	-1.385960	2.680469
H	-4.745412	-1.272087	2.723591
H	-7.351904	2.102341	1.928749
H	-7.191187	0.852461	0.693694
H	-7.941296	0.473605	2.250662
H	1.856206	-4.698195	2.907026
H	2.263463	-4.940446	4.609609
H	1.463235	-3.454976	4.103252
H	4.990319	-2.376544	4.807889
H	4.001261	-3.505253	5.747881
H	3.288504	-2.011133	5.113150
H	4.592210	-5.411062	4.161760
H	4.375944	-5.224109	2.416069
H	5.631494	-4.311605	3.256113
H	3.288965	2.010848	-5.113263
H	4.001723	3.505058	-5.747787
H	4.990748	2.376281	-4.807841
H	4.376028	5.223609	-2.415788
H	4.592633	5.410652	-4.161426
H	5.631734	4.311135	-3.255636
H	2.263822	4.940078	-4.609576
H	1.463548	3.454590	-4.103338
H	1.856459	4.697758	-2.907035

9.2.6 (***P*,*P*,*P***)-D_3-4

C	-5.53680	-2.84110	0.33860
C	-5.99920	-1.65530	-0.33690
C	-5.05590	-0.57980	-0.64570
C	-3.69610	-0.73230	-0.28110
C	-3.21630	-1.96310	0.28010
C	-4.11420	-2.99520	0.64550
C	-1.82880	-2.15480	0.46110
C	-1.40170	-3.30190	1.15310
C	-2.27770	-4.30120	1.55980
C	-3.63780	-4.14380	1.26710
C	-5.48230	0.58780	-1.26820
C	-4.58740	1.62350	-1.56240
C	-3.26610	1.48080	-1.15570
C	-2.80430	0.34770	-0.46290
C	-1.40070	0.20480	-0.06380
C	-0.89220	-1.09950	0.06240
N	-6.37260	-3.81210	0.66760
C	-7.67560	-3.66530	0.34430
C	-8.13220	-2.49420	-0.33840
N	-7.27210	-1.50530	-0.66390
C	-8.60680	-4.68130	0.67890
C	-9.92930	-4.53640	0.34540
C	-10.38170	-3.37610	-0.33450
C	-9.50580	-2.37540	-0.67050
C	5.23010	-3.37280	0.33600
C	4.43410	-4.36660	-0.33830
C	3.03030	-4.08860	-0.64490

C	2.48220	-2.83500	-0.28010
C	3.30820	-1.80360	0.28020
C	4.65150	-2.06440	0.64440
C	2.78000	-0.50640	0.46180
C	3.55970	0.43710	1.15410
C	4.86340	0.17850	1.56030
C	5.40780	-1.07740	1.26620
C	2.23200	-5.04270	-1.26550
C	0.88700	-4.78660	-1.55760
C	0.34990	-3.57080	-1.15150
C	1.10070	-2.60320	-0.46050
C	0.52260	-1.31600	-0.06250
C	1.39780	-0.22330	0.06300
N	6.48980	-3.60990	0.66250
C	7.01480	-4.81100	0.33760
C	6.22860	-5.79260	-0.34380
N	4.94120	-5.54340	-0.66670
C	8.36130	-5.10810	0.66910
C	8.89770	-6.32510	0.33400
C	8.11870	-7.29770	-0.34450
C	6.81320	-7.04080	-0.67750
C	0.30680	6.21530	0.33720
C	1.56640	6.02270	-0.33530
C	2.02690	4.66800	-0.64270
C	1.21440	3.56660	-0.27950
C	-0.09250	3.76650	0.27930
C	-0.53820	5.06030	0.64330
C	-0.95240	2.66070	0.45940
C	-2.16050	2.86450	1.14950
C	-2.58880	4.12300	1.55480
C	-1.77220	5.22210	1.26280
C	3.25260	4.45350	-1.26270
C	3.70260	3.16070	-1.55610
C	2.91750	2.08770	-1.15110
C	1.70400	2.25430	-0.46040
C	0.87790	1.11010	-0.06270
C	-0.50610	1.32190	0.06170
N	-0.11740	7.42480	0.66430
C	0.66160	8.47980	0.34190
C	1.90580	8.28950	-0.33770
N	2.33320	7.05000	-0.66130
C	0.24600	9.79430	0.67430
C	1.03320	10.86720	0.34170
C	2.26600	10.67870	-0.33490
C	2.69590	9.41970	-0.66870
C	-1.79810	-5.53950	2.32690
C	-2.34370	-6.81550	1.65270
C	-2.33230	-5.46100	3.77250
C	-0.26390	-5.63530	2.36990
C	-5.07190	2.85860	-2.33150
C	-6.33830	3.42900	-1.65990
C	-5.40980	2.43720	-3.77710
C	-4.00770	3.96770	-2.37440
C	5.69560	1.21260	2.32840
C	5.89430	0.70950	3.77370
C	5.01110	2.58880	2.37230

C	7.07350	1.37890	1.65480
C	0.05860	-5.82550	-2.32340
C	0.59070	-5.91110	-3.76940
C	-1.43410	-5.45840	-2.36520
C	0.19850	-7.20580	-1.64850
C	-3.90230	4.32710	2.31970
C	-3.56970	4.75170	3.76550
C	-4.75210	3.04620	2.36220
C	-4.73360	5.43680	1.64310
C	5.01620	2.96280	-2.32230
C	6.14200	3.77350	-1.64740
C	4.82380	3.46720	-3.76800
C	5.44420	1.48650	-2.36520
H	-0.35290	-3.40000	1.37490
H	-4.36160	-4.90140	1.53250
H	-6.52810	0.65590	-1.53290
H	-2.56020	2.26240	-1.37820
H	-8.23540	-5.55700	1.19650
H	-10.64080	-5.31280	0.60070
H	-11.43140	-3.28520	-0.58790
H	-9.82530	-1.47950	-1.18810
H	3.11980	1.39400	1.37660
H	6.42620	-1.32490	1.53040
H	2.69610	-5.98220	-1.53040
H	-0.68030	-3.35110	-1.37300
H	8.93430	-4.34820	1.18580
H	9.92670	-6.55200	0.58700
H	8.56530	-8.25160	-0.59920
H	6.19670	-7.76600	-1.19410
H	-2.76990	2.00530	1.37110
H	-2.06710	6.22780	1.52710
H	3.83480	5.32510	-1.52630
H	3.24190	1.08570	-1.37340
H	-0.69940	9.91070	1.18950
H	0.71550	11.87170	0.59540
H	2.86990	11.54220	-0.58760
H	3.63290	9.24820	-1.18390
H	-2.01620	-6.87970	0.61300
H	-1.98030	-7.70170	2.18010
H	-3.43450	-6.84770	1.66260
H	-1.95600	-4.56640	4.27520
H	-2.01300	-6.33680	4.34540
H	-3.42380	-5.42240	3.78950
H	0.03060	-6.55650	2.87820
H	0.17050	-5.65000	1.36740
H	0.18020	-4.80230	2.92020
H	-6.14240	3.69930	-0.62030
H	-6.67000	4.32660	-2.18900
H	-7.16300	2.71430	-1.67030
H	-4.52660	2.03280	-4.27800
H	-5.76700	3.29700	-4.35170
H	-6.18700	1.66960	-3.79400
H	-4.41420	4.84470	-2.88390
H	-3.69880	4.27350	-1.37190
H	-3.11640	3.65500	-2.92380
H	6.40700	-0.25490	3.78990

H	6.49250	1.42360	4.34740
H	4.93130	0.58720	4.27590
H	4.80670	2.97330	1.37010
H	5.66130	3.30430	2.88150
H	4.06750	2.55620	2.92250
H	7.65910	2.13630	2.18320
H	7.64700	0.45040	1.66400
H	6.96570	1.69570	0.61540
H	1.64390	-6.20030	-3.78700
H	0.02390	-6.65180	-4.34130
H	0.49840	-4.94520	-4.27270
H	-1.85210	-5.34180	-1.36240
H	-1.99100	-6.24990	-2.87240
H	-1.60960	-4.53130	-2.91620
H	1.22980	-7.56260	-1.65920
H	-0.13210	-7.16860	-0.60850
H	-0.41370	-7.94320	-2.17490
H	-2.99080	5.67790	3.78270
H	-4.48870	4.91320	4.33670
H	-2.98370	3.97910	4.26980
H	-4.98020	2.67650	1.35950
H	-5.69810	3.25190	2.86870
H	-4.25360	2.24560	2.91400
H	-5.68350	5.56570	2.16910
H	-4.21610	6.39770	1.65280
H	-4.95150	5.18420	0.60340
H	6.27550	3.46790	-0.60770
H	7.08650	3.61220	-2.17430
H	5.93560	4.84510	-1.65740
H	4.03310	2.90510	-4.27120
H	5.74840	3.34680	-4.34040
H	4.54800	4.52410	-3.78480
H	6.40800	1.40000	-2.87260
H	5.55220	1.06550	-1.36270
H	4.72870	0.87150	-2.91640

(*M*,*M*,*M*)-D_3-4

C	-6.153183	-0.930882	-0.337465
C	-6.213763	0.340398	0.337881
C	-4.977163	1.060173	0.645193
C	-3.736722	0.482970	0.280037
C	-3.673813	-0.836723	-0.280756
C	-4.853747	-1.529495	-0.645437
C	-2.419528	-1.460003	-0.462299
C	-2.380060	-2.683340	-1.154523
C	-3.528844	-3.351894	-1.560647
C	-4.767896	-2.769901	-1.267266
C	-5.009700	2.303272	1.266714
C	-3.831652	3.000932	1.559154
C	-2.624570	2.445066	1.152254
C	-2.547493	1.223159	0.460657
C	-1.262406	0.640589	0.062005
C	-1.195691	-0.757798	-0.063677
N	-7.254737	-1.585752	-0.665401
C	-8.443060	-1.032254	-0.340865

C	-8.502886	0.223219	0.342033
N	-7.372579	0.887372	0.666219
C	-9.649277	-1.699399	-0.674105
C	-10.856756	-1.141565	-0.339215
C	-10.916028	0.102269	0.340882
C	-9.767068	0.772508	0.675547
C	3.883043	-4.863354	-0.335802
C	2.811636	-5.551812	0.337960
C	1.569757	-4.840857	0.644442
C	1.449737	-3.477828	0.279744
C	2.561562	-2.763334	-0.280113
C	3.751872	-3.438706	-0.643725
C	2.474395	-1.365401	-0.461593
C	3.514619	-0.719541	-1.153138
C	4.668372	-1.380127	-1.558254
C	4.783763	-2.744073	-1.264412
C	0.508989	-5.490806	1.264906
C	-0.684293	-4.819451	1.557075
C	-0.805883	-3.495777	1.151133
C	0.214131	-2.817939	0.460116
C	0.076435	-1.413604	0.061721
C	1.254153	-0.656610	-0.063476
N	5.001604	-5.489529	-0.662224
C	5.116247	-6.795448	-0.337870
C	4.057950	-7.475485	0.343068
N	2.917066	-6.829006	0.665890
C	6.297883	-7.506038	-0.669335
C	6.418304	-8.830750	-0.334706
C	5.369740	-9.504533	0.343313
C	4.214077	-8.845048	0.676268
C	2.270726	5.794257	-0.338239
C	3.402630	5.210746	0.335709
C	3.407523	3.780024	0.643412
C	2.286969	2.994553	0.279286
C	1.112244	3.600134	-0.280509
C	1.102005	4.968519	-0.644827
C	-0.054968	2.825696	-0.461016
C	-1.135056	3.403710	-1.151355
C	-1.140197	4.733111	-1.556581
C	-0.016019	5.514845	-1.264720
C	4.500581	3.186579	1.264438
C	4.515301	1.817764	1.558030
C	3.429816	1.050547	1.152325
C	2.333152	1.594629	0.460466
C	1.185964	0.773040	0.062038
C	-0.058448	1.414507	-0.063180
N	2.254343	7.075698	-0.666108
C	3.328513	7.827721	-0.342958
C	4.446433	7.251403	0.338397
N	4.456407	5.940474	0.662638
C	3.353874	9.205914	-0.676197
C	4.441467	9.972298	-0.342827
C	5.549061	9.401297	0.335674
C	5.555007	8.071146	0.670331
C	-3.468679	-4.678308	-2.327984
C	-3.950628	-4.433448	-3.773370

C	-4.391867	-5.714355	-1.653697
C	-2.044807	-5.257311	-2.371503
C	-3.897776	4.327202	2.326225
C	-4.350881	4.037511	3.772642
C	-4.917800	5.269388	1.653813
C	-2.536092	5.040481	2.366661
C	5.787824	-0.665084	-2.324677
C	5.577952	0.857551	-2.368351
C	5.817585	-1.204781	-3.770119
C	7.146053	-0.947048	-1.649424
C	-1.800414	-5.540465	2.322806
C	-2.106188	-6.894208	1.648902
C	-3.099018	-4.717953	2.363104
C	-1.323993	-5.789441	3.769314
C	-2.320595	5.345119	-2.320745
C	-3.535000	4.402672	-2.360965
C	-1.871314	5.639909	-3.767322
C	-2.753245	6.662803	-1.644924
C	5.697162	1.212159	2.325052
C	5.676035	1.753575	3.769972
C	7.022684	1.620295	1.649326
C	5.631789	-0.323521	2.370106
H	-1.417200	-3.110128	-1.376803
H	-5.695327	-3.257735	-1.532128
H	-5.979292	2.700390	1.532076
H	-1.706709	2.961989	1.373664
H	-9.576221	-2.647753	-1.191858
H	-11.778460	-1.651534	-0.593522
H	-11.882040	0.522194	0.595369
H	-9.784519	1.723528	1.193273
H	3.403014	0.327758	-1.375472
H	5.670314	-3.303294	-1.528154
H	0.649484	-6.529255	1.529702
H	-1.712448	-2.959264	1.372590
H	7.083397	-6.968199	-1.185549
H	7.321382	-9.373654	-0.587655
H	5.488907	-10.551159	0.597584
H	3.398442	-9.336034	1.192466
H	-1.986477	2.783493	-1.372854
H	0.024833	6.562120	-1.529038
H	5.329751	3.827548	1.528808
H	3.418187	-0.002578	1.374665
H	2.495471	9.617140	-1.192743
H	4.460696	11.025501	-0.597140
H	6.396327	10.027612	0.588951
H	6.387891	7.610434	1.186886
H	-3.309244	-3.705227	-4.276243
H	-3.927284	-5.365136	-4.346469
H	-4.973055	-4.049084	-3.789747
H	-4.101577	-5.879787	-0.614178
H	-4.329588	-6.670040	-2.181399
H	-5.436233	-5.397766	-1.663322
H	-2.059088	-6.224961	-2.878792
H	-1.359147	-4.609596	-2.923150
H	-1.636893	-5.408251	-1.369111
H	-3.641279	3.374623	4.274243

H	-4.416220	4.967301	4.345586
H	-5.331476	3.556429	3.791158
H	-4.647406	5.461079	0.613438
H	-4.946190	6.227034	2.180872
H	-5.926931	4.854017	1.666348
H	-2.642178	6.002251	2.874254
H	-1.789991	4.461711	2.916505
H	-2.146858	5.230020	1.363387
H	5.505210	1.286530	-1.366004
H	6.423236	1.328518	-2.875879
H	4.674210	1.127804	-2.919883
H	5.994911	-2.282573	-3.786449
H	6.613808	-0.719402	-4.342355
H	4.866906	-1.012447	-4.273881
H	7.943281	-0.415988	-2.176781
H	7.143684	-0.612571	-0.610017
H	7.393287	-2.009962	-1.658544
H	-1.241744	-7.560237	1.660331
H	-2.921040	-7.398469	2.175607
H	-2.407823	-6.754727	0.608808
H	-2.971289	-3.782787	2.913667
H	-3.879106	-5.291173	2.869846
H	-3.457185	-4.475078	1.359761
H	-0.416952	-6.397957	3.787929
H	-1.105284	-4.843955	4.272081
H	-2.096887	-6.311676	4.341093
H	-3.868444	4.126683	-1.357633
H	-4.366165	4.899080	-2.867557
H	-3.318805	3.484002	-2.911688
H	-1.026941	6.332750	-3.786018
H	-2.691182	6.085944	-4.338196
H	-1.563167	4.720183	-4.271017
H	-3.612647	7.088060	-2.170552
H	-3.039649	6.493884	-0.604890
H	-1.955951	7.407928	-1.655813
H	4.747421	1.473602	4.273807
H	6.514069	1.345347	4.342756
H	5.751727	2.843241	3.785345
H	7.051452	1.286722	0.610031
H	7.866394	1.166657	2.176636
H	7.168974	2.701732	1.657980
H	6.517446	-0.712263	2.878427
H	4.757256	-0.677269	2.921511
H	5.600231	-0.758450	1.368188

9.3 Coordinates – Transition States

9.3.1 C_2-C_2-Transition State of 2

C	-3.49050	4.54950	2.09530
C	-2.31430	4.84490	2.70350
C	-1.11070	4.14670	2.35180
C	-1.19930	3.07570	1.42090
C	-2.45170	2.68280	0.87620
C	-3.58900	3.49370	1.12570
C	-2.55910	1.53960	0.02710

C	-3.71420	1.44810	-0.75850
C	-4.78470	2.35520	-0.65300
C	-4.74490	3.30020	0.36450
C	0.14300	4.51600	2.85010
C	1.31210	3.86430	2.44870
C	1.20790	2.84280	1.50580
C	-0.02180	2.45540	0.95050
C	-0.13670	1.33080	0.04070
C	-1.37520	0.66050	-0.09450
C	-5.98060	2.31810	-1.61800
C	-7.06540	1.39840	-1.02310
C	-6.58840	3.72270	-1.81220
C	-5.55480	1.82700	-3.01480
C	-1.77030	-4.99050	2.84340
C	-3.00410	-4.77740	2.32430
C	-3.24010	-3.74750	1.35030
C	-2.17550	-2.87550	0.99340
C	-0.86620	-3.17330	1.46020
C	-0.64410	-4.22040	2.39790
C	0.23370	-2.47030	0.92290
C	1.51790	-2.75970	1.41380
C	1.75220	-3.76320	2.35070
C	0.65900	-4.49460	2.82280
C	-4.46430	-3.63350	0.69270
C	-4.64650	-2.71110	-0.33160
C	-3.64760	-1.74810	-0.54470
C	-2.42050	-1.76010	0.13540
C	-1.31190	-0.80240	-0.07880
C	-0.01540	-1.36120	0.02170
C	3.16430	-4.07570	2.86390
C	4.23750	-3.22140	2.16950
C	3.22470	-3.80030	4.38060
C	3.48660	-5.56160	2.59930
C	5.45080	0.92500	-2.73950
C	5.49930	-0.42980	-2.77060
C	4.39800	-1.22670	-2.30500
C	3.27200	-0.56690	-1.73270
C	3.22050	0.85660	-1.70050
C	4.29600	1.61970	-2.24070
C	2.06970	1.52450	-1.21150
C	1.97930	2.91370	-1.38260
C	3.02710	3.67540	-1.89460
C	4.18830	3.01110	-2.30000
C	4.39000	-2.61850	-2.42400
C	3.28250	-3.38240	-2.04410
C	2.18460	-2.72110	-1.49870
C	2.17470	-1.33670	-1.27270
C	1.06980	-0.65520	-0.61660
C	1.01550	0.73530	-0.59030
C	2.92860	5.20100	-2.02790
C	1.62970	5.75360	-1.41800
C	4.11860	5.85880	-1.29830
C	2.97110	5.58080	-3.52260
C	2.66480	4.27990	3.04290
C	2.65970	3.99180	4.55850
C	2.88780	5.78890	2.80890

C	3.83800	3.51810	2.40480
C	-5.90690	-2.82890	-1.20190
C	-7.15080	-2.49520	-0.35250
C	-6.03000	-4.27550	-1.73030
C	-5.85760	-1.90310	-2.42270
C	3.29490	-4.90410	-2.24210
C	4.54340	-5.50330	-1.56150
C	3.33660	-5.21490	-3.75270
C	2.05210	-5.57710	-1.63640
H	-4.38520	5.12030	2.31950
H	-2.25440	5.64640	3.43160
H	-3.73490	0.71680	-1.54620
H	-5.58220	3.96350	0.53580
H	0.18960	5.33300	3.56090
H	2.09580	2.32170	1.19130
H	-7.90750	1.28800	-1.71320
H	-6.66750	0.40900	-0.80570
H	-7.44240	1.81300	-0.08480
H	-7.37580	3.67710	-2.56910
H	-5.83320	4.43760	-2.14770
H	-7.04340	4.10910	-0.89860
H	-4.79330	2.48740	-3.43750
H	-5.15330	0.81530	-3.01210
H	-6.41790	1.82940	-3.68550
H	-1.60490	-5.77390	3.57500
H	-3.84340	-5.39550	2.62440
H	2.34550	-2.17360	1.05480
H	0.80520	-5.29730	3.53620
H	-5.24790	-4.33840	0.94340
H	-3.79350	-1.02500	-1.32370
H	5.22530	-3.50150	2.54380
H	4.23560	-3.36580	1.08640
H	4.09880	-2.15610	2.36960
H	4.22350	-4.02100	4.76880
H	2.50630	-4.41440	4.92810
H	2.99840	-2.75200	4.59110
H	3.43050	-5.78720	1.53200
H	4.49710	-5.79670	2.94590
H	2.79280	-6.22610	3.11810
H	6.27990	1.51410	-3.11660
H	6.36780	-0.94030	-3.17270
H	1.06990	3.40410	-1.08090
H	5.02340	3.57050	-2.70570
H	5.26130	-3.09810	-2.85470
H	1.31490	-3.28720	-1.21120
H	1.62320	6.84330	-1.49950
H	0.74530	5.37960	-1.93960
H	1.53480	5.49430	-0.36080
H	4.05160	6.94780	-1.37370
H	5.07650	5.55390	-1.72410
H	4.12240	5.59000	-0.23960
H	2.13600	5.12450	-4.06000
H	2.90490	6.66630	-3.64260
H	3.89710	5.24430	-3.99420
H	2.50280	2.92690	4.74770
H	3.61470	4.28330	5.00560

H	1.86580	4.54280	5.06750
H	2.87450	6.02340	1.74210
H	3.85680	6.09440	3.21390
H	2.11870	6.39350	3.29340
H	4.77890	3.87080	2.83460
H	3.77240	2.44350	2.59160
H	3.88380	3.67250	1.32400
H	-7.08220	-1.49150	0.07040
H	-8.05740	-2.55030	-0.96250
H	-7.26020	-3.19680	0.47740
H	-5.14560	-4.55150	-2.31010
H	-6.90640	-4.36230	-2.37910
H	-6.14110	-5.00060	-0.92260
H	-6.75870	-2.03580	-3.02640
H	-5.81220	-0.85700	-2.13400
H	-4.99350	-2.12400	-3.05490
H	4.54970	-5.27810	-0.49280
H	4.55220	-6.59040	-1.68150
H	5.46860	-5.11410	-1.99090
H	2.45960	-4.80000	-4.25580
H	3.34890	-6.29620	-3.91970
H	4.22570	-4.78960	-4.22350
H	2.12480	-6.66000	-1.76420
H	1.95850	-5.36920	-0.56770
H	1.13290	-5.24910	-2.12780

9.3.2 C_2-D_3-Transition State of 2

C	1.70940	5.67600	-1.43410
C	2.96960	5.17550	-1.46950
C	3.27570	3.90800	-0.86060
C	2.21240	3.03780	-0.51460
C	0.89580	3.57620	-0.46690
C	0.65890	4.93880	-0.78940
C	-0.10890	2.82590	0.17870
C	-1.18280	3.51800	0.74470
C	-1.40500	4.87900	0.50570
C	-0.51700	5.55020	-0.33600
C	4.56190	3.62870	-0.38870
C	4.77210	2.59580	0.51570
C	3.74550	1.64650	0.68620
C	2.48400	1.77190	0.09490
C	1.36760	0.80060	0.17780
C	0.06030	1.37320	0.23350
C	-2.53630	5.63900	1.21340
C	-3.61900	4.68850	1.75530
C	-1.91860	6.41010	2.40070
C	-3.20860	6.64740	0.26120
C	-5.56840	-1.33360	2.02850
C	-5.89510	-0.27310	1.24350
C	-4.89470	0.47370	0.53070
C	-3.53010	0.13040	0.72970
C	-3.20560	-1.04610	1.46400
C	-4.20990	-1.78930	2.13060
C	-1.88690	-1.55510	1.40050
C	-1.57520	-2.72950	2.08400
C	-2.54130	-3.44590	2.80430

C	-3.85130	-2.97020	2.80330
C	-5.21490	1.48520	-0.38160
C	-4.22430	2.14040	-1.11440
C	-2.88550	1.85970	-0.82240
C	-2.50720	0.91250	0.13630
C	-1.10010	0.54710	0.39190
C	-0.88830	-0.80150	0.66720
C	-2.12120	-4.72450	3.54110
C	-3.29630	-5.38750	4.27550
C	-1.55320	-5.73690	2.52410
C	-1.03540	-4.37410	4.58020
C	3.17200	-4.04250	-3.19100
C	1.90100	-4.42010	-3.47220
C	0.80530	-3.93790	-2.68480
C	1.06080	-2.96390	-1.68050
C	2.37780	-2.47250	-1.46340
C	3.45430	-3.11600	-2.13230
C	2.63960	-1.43920	-0.51080
C	3.95150	-1.38690	-0.01550
C	4.99640	-2.18330	-0.52140
C	4.75510	-2.92990	-1.66510
C	-0.49480	-4.43480	-2.86030
C	-1.53930	-3.99900	-2.05630
C	-1.25850	-3.06620	-1.05310
C	0.01740	-2.54100	-0.82390
C	0.26860	-1.38640	0.05700
C	1.46790	-0.64580	-0.03010
C	6.36230	-2.34640	0.16440
C	7.48090	-1.82050	-0.75830
C	6.42660	-1.62680	1.51440
C	6.60510	-3.84850	0.43930
C	6.08250	2.55550	1.31890
C	6.56460	3.98350	1.65720
C	7.16950	1.86770	0.47090
C	5.88860	1.83850	2.66810
C	-4.61630	3.09790	-2.24930
C	-5.66930	4.11340	-1.76060
C	-5.21950	2.26080	-3.39820
C	-3.40810	3.87580	-2.79700
C	-2.99080	-4.46700	-2.22380
C	-3.47260	-5.12000	-0.91110
C	-3.14130	-5.48890	-3.36010
C	-3.88260	-3.24680	-2.54190
H	1.49790	6.67350	-1.80340
H	3.78910	5.76860	-1.86070
H	-1.86920	2.97390	1.37090
H	-0.66820	6.59650	-0.57110
H	5.34980	4.33540	-0.61380
H	3.89460	0.84400	1.38560
H	-4.44090	5.27220	2.17760
H	-4.02400	4.04480	0.97270
H	-3.23530	4.04770	2.55210
H	-2.69240	6.96150	2.94400
H	-1.16650	7.12370	2.05580
H	-1.43410	5.72170	3.09750
H	-2.51890	7.42840	-0.06410

H	-3.60160	6.15340	-0.62890
H	-4.04020	7.14080	0.77120
H	-6.34000	-1.88570	2.55410
H	-6.93200	0.02470	1.13070
H	-0.55610	-3.09020	2.03690
H	-4.63220	-3.51710	3.31490
H	-6.26100	1.71390	-0.54640
H	-2.11020	2.37610	-1.36210
H	-2.94660	-6.28810	4.78600
H	-3.72800	-4.72430	5.02950
H	-4.08820	-5.68570	3.58370
H	-1.22240	-6.64370	3.03890
H	-0.70060	-5.32960	1.97760
H	-2.31390	-6.01600	1.79200
H	-1.41410	-3.65060	5.30670
H	-0.72460	-5.27320	5.12020
H	-0.15020	-3.94320	4.10830
H	4.00910	-4.46030	-3.73970
H	1.69570	-5.13350	-4.26280
H	4.15410	-0.77960	0.84410
H	5.55540	-3.48000	-2.14520
H	-0.65850	-5.16820	-3.63890
H	-2.08620	-2.72030	-0.46290
H	8.45440	-1.90740	-0.26660
H	7.52430	-2.38980	-1.68930
H	7.32120	-0.77350	-1.01980
H	7.40060	-1.79560	1.98040
H	5.65850	-1.99640	2.19910
H	6.30040	-0.55520	1.40430
H	6.63960	-4.43340	-0.48100
H	7.55840	-3.98350	0.95890
H	5.81070	-4.25880	1.06770
H	5.78840	4.54770	2.17960
H	7.44270	3.92720	2.30640
H	6.85630	4.54640	0.76930
H	6.84490	0.88610	0.13330
H	8.09700	1.74950	1.03950
H	7.38790	2.46260	-0.41970
H	6.83250	1.82870	3.21910
H	5.14680	2.36450	3.27470
H	5.56280	0.80550	2.56960
H	-5.29470	4.69750	-0.91850
H	-5.92390	4.80480	-2.56860
H	-6.59270	3.62440	-1.44450
H	-4.49500	1.52850	-3.76300
H	-5.50500	2.90790	-4.23310
H	-6.10810	1.71780	-3.06770
H	-3.74420	4.58300	-3.55940
H	-2.89510	4.43900	-2.01500
H	-2.67880	3.21160	-3.26660
H	-3.42910	-4.42360	-0.07170
H	-4.50830	-5.45710	-1.01440
H	-2.85300	-5.98540	-0.66230
H	-2.84100	-5.06810	-4.32310
H	-4.18710	-5.79510	-3.44280
H	-2.54530	-6.38670	-3.17610

H	-4.92120	-3.56320	-2.67660
H	-3.86080	-2.50800	-1.73830
H	-3.55150	-2.75460	-3.45990

9.3.3 *C*2-*C*2-Transition State of 3*

*: Calculated at the PM6-level of theory.

C	5.53610	-1.49450	-2.51930
C	5.87770	-0.05070	-2.06420
C	4.70350	0.83710	-1.85360
C	3.46240	0.26970	-1.46330
C	3.25660	-1.17100	-1.48300
C	4.24910	-2.04030	-2.01210
C	2.02330	-1.71440	-1.06840
C	1.78300	-3.10150	-1.25870
C	2.75570	-3.95150	-1.76090
C	4.00860	-3.40080	-2.12930
C	4.84820	2.20940	-1.99700
C	3.75020	3.07370	-1.77760
C	2.54780	2.52250	-1.35070
C	2.40160	1.13210	-1.11300
C	1.18530	0.55960	-0.55000
C	1.00790	-0.82470	-0.51680
O	7.01970	0.28890	-1.88490
O	6.27780	-2.09730	-3.25420
C	-4.05800	-4.32120	1.68170
C	-2.98410	-4.37070	2.80450
C	-1.63970	-3.89460	2.38110
C	-1.55030	-2.91730	1.36050
C	-2.73310	-2.43580	0.66050
C	-3.93230	-3.18460	0.72880
C	-2.64830	-1.30290	-0.18380
C	-3.69680	-1.10390	-1.10970
C	-4.82070	-1.92730	-1.14790
C	-4.96890	-2.92030	-0.15470
C	-0.49470	-4.37550	2.99830
C	0.78290	-3.89730	2.61670
C	0.86650	-2.93450	1.61870
C	-0.28950	-2.43190	0.96860
C	-0.23310	-1.30380	0.04370
C	-1.39570	-0.52860	-0.17830
O	-3.25300	-4.76110	3.91080
O	-4.90440	-5.17560	1.60150
C	-1.72680	5.06320	2.73190
C	-2.80870	5.26390	1.63510
C	-2.99900	4.10880	0.71560
C	-2.01560	3.09490	0.62400
C	-0.72940	3.28770	1.27960
C	-0.55100	4.27070	2.28230
C	0.36240	2.49540	0.87700
C	1.61910	2.71120	1.49540
C	1.80500	3.69860	2.45780
C	0.69200	4.46820	2.86700
C	-4.11310	4.07700	-0.11020
C	-4.25570	3.05290	-1.07150
C	-3.35180	1.99190	-1.05970

C	-2.23910	1.95780	-0.18920
C	-1.21210	0.90210	-0.19630
C	0.11280	1.36270	-0.01040
O	-3.41440	6.30210	1.54680
O	-1.86320	5.51670	3.83840
C	2.53670	-5.46050	-1.91680
C	1.05660	-5.85080	-1.75600
C	3.36590	-6.16790	-0.82780
C	3.00670	-5.90500	-3.31520
C	3.85620	4.58320	-2.02480
C	3.15720	4.88500	-3.36400
C	5.32060	5.05160	-2.09650
C	3.16280	5.34620	-0.88000
C	-5.89860	-1.82240	-2.23340
C	-7.05470	-0.97880	-1.67210
C	-6.41580	-3.22830	-2.60400
C	-5.34180	-1.18210	-3.51640
C	2.02710	-4.47750	3.29920
C	2.26800	-5.87920	2.70680
C	1.78980	-4.57750	4.81810
C	3.27350	-3.60520	3.06320
C	3.17320	3.90530	3.11870
C	4.30930	3.58180	2.13090
C	3.24570	2.95950	4.33190
C	3.34490	5.36380	3.58320
C	-5.39510	3.16950	-2.09110
C	-5.64000	4.64970	-2.45250
C	-6.66280	2.58020	-1.45120
C	-5.05620	2.42690	-3.39470
H	0.79310	-3.49440	-1.00720
H	4.79270	-4.04950	-2.53690
H	5.82370	2.61070	-2.29020
H	1.68280	3.17450	-1.19170
H	-3.58550	-0.30520	-1.85310
H	-5.88560	-3.51860	-0.09890
H	-0.58600	-5.12280	3.79330
H	1.84230	-2.53080	1.33300
H	2.45930	2.06340	1.22640
H	0.79770	5.21680	3.65920
H	-4.85930	4.87630	-0.03290
H	-3.47000	1.17640	-1.78280
H	0.42470	-5.35940	-2.50390
H	0.92030	-6.93130	-1.88270
H	0.66760	-5.59770	-0.76350
H	4.43000	-5.91990	-0.89440
H	3.28160	-7.25680	-0.91160
H	3.02370	-5.88900	0.18400
H	2.79670	-6.96670	-3.48560
H	2.49920	-5.34060	-4.10540
H	4.08480	-5.76860	-3.45470
H	2.10160	4.59390	-3.34760
H	3.19650	5.95400	-3.59970
H	3.63110	4.34880	-4.19360
H	5.86770	4.81690	-1.17680
H	5.37880	6.13740	-2.23700
H	5.85990	4.59810	-2.93550

H	3.30410	6.42750	-0.97880
H	2.08260	5.16740	-0.85450
H	3.56920	5.04680	0.10190
H	-6.71680	0.03170	-1.38550
H	-7.85750	-0.86090	-2.40740
H	-7.49490	-1.43720	-0.77960
H	-5.59890	-3.88620	-2.92010
H	-7.13510	-3.18000	-3.42900
H	-6.92870	-3.71770	-1.76790
H	-6.08670	-1.19970	-4.32050
H	-5.06460	-0.12650	-3.36690
H	-4.45880	-1.71690	-3.88250
H	2.47430	-5.82930	1.62360
H	3.12680	-6.36680	3.18040
H	1.40390	-6.53660	2.84620
H	1.51280	-3.60640	5.24400
H	2.69130	-4.91760	5.33950
H	0.99230	-5.28560	5.06920
H	3.53320	-3.53260	2.00140
H	3.13290	-2.58920	3.44840
H	4.14790	-4.02420	3.57480
H	4.21750	4.17870	1.20680
H	5.28960	3.80170	2.56700
H	4.32150	2.52540	1.84140
H	2.45340	3.17400	5.05800
H	4.20330	3.05610	4.85460
H	3.13870	1.91110	4.03390
H	3.21910	6.06860	2.75450
H	2.62790	5.63620	4.36620
H	4.34430	5.52840	4.00210
H	-4.72730	5.12900	-2.82350
H	-6.40060	4.74500	-3.23520
H	-5.99530	5.23420	-1.59580
H	-6.53720	1.50970	-1.21500
H	-7.52620	2.67020	-2.11870
H	-6.91910	3.08990	-0.51580
H	-5.82860	2.59330	-4.15460
H	-4.10640	2.77180	-3.81770
H	-4.98890	1.33720	-3.24780

9.3.4 C_2-D_3-Transition State of 3

C	-3.14770	-4.44350	-2.78850
C	-1.70500	-4.69140	-3.28300
C	-0.61480	-4.06900	-2.51090
C	-0.90000	-3.12180	-1.50090
C	-2.25160	-2.75810	-1.15170
C	-3.33240	-3.52290	-1.64910
C	-2.50400	-1.74310	-0.19440
C	-3.74980	-1.81230	0.47310
C	-4.75600	-2.71610	0.12410
C	-4.57050	-3.47270	-1.03590
C	0.69350	-4.45430	-2.78470
C	1.76270	-3.91390	-2.06730
C	1.46830	-2.98910	-1.07190
C	0.15910	-2.58170	-0.74460
C	-0.11250	-1.43490	0.13610

C	-1.38430	-0.82660	0.15830
O	-1.52680	-5.41000	-4.24630
O	-4.06610	-5.01510	-3.34100
C	-2.37320	5.38660	-1.51310
C	-3.73150	4.63570	-1.46200
C	-3.77500	3.39470	-0.66280
C	-2.58350	2.69450	-0.38140
C	-1.32190	3.39440	-0.43960
C	-1.24890	4.76070	-0.79510
C	-0.21320	2.79570	0.17700
C	0.79730	3.63620	0.68640
C	0.83380	5.00350	0.43490
C	-0.17020	5.53490	-0.39010
C	-4.96210	3.03680	-0.04300
C	-4.96300	2.05420	0.95320
C	-3.83530	1.23220	1.04590
C	-2.65320	1.45540	0.30830
C	-1.43050	0.62080	0.33830
C	-0.19800	1.33670	0.28860
O	-4.70090	5.12170	-2.00620
O	-2.30820	6.45840	-2.07910
C	5.79110	-0.65860	2.01440
C	5.99970	0.52280	1.02460
C	4.81060	1.07030	0.33990
C	3.52630	0.54870	0.62410
C	3.37510	-0.67480	1.37280
C	4.45230	-1.28150	2.05500
C	2.14200	-1.34870	1.32020
C	1.99450	-2.57580	2.00080
C	3.03780	-3.16120	2.70440
C	4.27440	-2.49130	2.71250
C	4.96580	2.08220	-0.59550
C	3.86310	2.60300	-1.29070
C	2.59900	2.16200	-0.91950
C	2.39210	1.18020	0.07440
C	1.05350	0.64610	0.38010
C	1.01970	-0.72180	0.64970
O	7.12480	0.94950	0.86060
O	6.73580	-1.04670	2.67020
C	-6.00480	-2.99880	0.96870
C	-7.26780	-2.63200	0.16360
C	-5.99520	-2.23230	2.29230
C	-6.04370	-4.50740	1.30140
C	3.22850	-4.27000	-2.34380
C	3.98740	-2.99440	-2.76630
C	3.36410	-5.31410	-3.46060
C	3.86220	-4.83730	-1.05680
C	1.87300	5.92640	1.08200
C	3.08240	5.14390	1.61910
C	1.18910	6.65470	2.25920
C	2.37560	6.97500	0.07090
C	-6.15020	1.97310	1.92410
C	-7.28930	1.17480	1.26320
C	-6.67510	3.38940	2.24640
C	-5.73320	1.34460	3.26500
C	2.89010	-4.48830	3.45560

C	1.49360	-5.10110	3.27950
C	3.13730	-4.23980	4.95770
C	3.93360	-5.49290	2.92460
C	4.08480	3.58150	-2.44990
C	4.75690	2.81060	-3.60590
C	5.01240	4.73020	-2.00540
C	2.76640	4.18260	-2.95950
H	-3.88140	-1.22010	1.35830
H	-5.35440	-4.11030	-1.42170
H	0.84840	-5.17830	-3.57160
H	2.29930	-2.54170	-0.55750
H	1.55870	3.19370	1.30760
H	-0.17090	6.58140	-0.66310
H	-5.84010	3.63360	-0.24540
H	-3.81430	0.46840	1.80290
H	1.02780	-3.05670	1.96680
H	5.12650	-2.91420	3.22880
H	5.96990	2.42630	-0.80400
H	1.73700	2.54790	-1.43720
H	-7.34530	-3.23060	-0.74710
H	-8.16290	-2.81740	0.76430
H	-7.26300	-1.57950	-0.12610
H	-5.12540	-2.49420	2.90150
H	-6.89280	-2.47470	2.86650
H	-5.98880	-1.15940	2.12890
H	-6.91320	-4.72320	1.92910
H	-6.11830	-5.12410	0.40360
H	-5.14560	-4.80870	1.84790
H	3.96280	-2.23110	-1.98490
H	5.03550	-3.23260	-2.96890
H	3.55310	-2.56500	-3.67340
H	2.85640	-6.24810	-3.20500
H	4.42130	-5.54120	-3.61900
H	2.95590	-4.95020	-4.40730
H	4.90810	-5.10120	-1.23750
H	3.83620	-4.11230	-0.24090
H	3.33480	-5.73700	-0.72840
H	3.55980	4.55170	0.83580
H	3.82250	5.84530	2.01220
H	2.80520	4.47200	2.43490
H	0.33670	7.24600	1.91450
H	1.89770	7.32990	2.74840
H	0.82810	5.93910	3.00320
H	3.15540	7.58560	0.53370
H	2.79730	6.50120	-0.81720
H	1.57910	7.64920	-0.25000
H	-6.95950	0.18080	0.96710
H	-8.13340	1.06750	1.95110
H	-7.64420	1.68810	0.36530
H	-5.87790	4.02790	2.63690
H	-7.45690	3.31890	3.00750
H	-7.11260	3.87980	1.37530
H	-5.33840	0.33530	3.16760
H	-4.96940	1.95680	3.75320
H	-6.60000	1.29380	3.92870
H	1.27180	-5.30590	2.22860

H	1.44350	-6.04830	3.82210
H	0.71020	-4.44950	3.67590
H	4.13680	-3.83660	5.13730
H	3.04630	-5.17760	5.51310
H	2.40790	-3.53140	5.36020
H	3.77990	-5.68870	1.86060
H	4.95400	-5.12760	3.05920
H	3.84350	-6.44060	3.46310
H	4.12080	1.98950	-3.94830
H	4.93570	3.48110	-4.45170
H	5.71720	2.39050	-3.29640
H	4.59800	5.26410	-1.14850
H	5.13390	5.44370	-2.82510
H	6.00640	4.37030	-1.73200
H	2.97910	4.90100	-3.75500
H	2.10460	3.41860	-3.37530
H	2.22710	4.70740	-2.16810

9.4 Frontier Molecular Orbitals

9.4.1 (*P,P,P*)-*D*$_3$-2

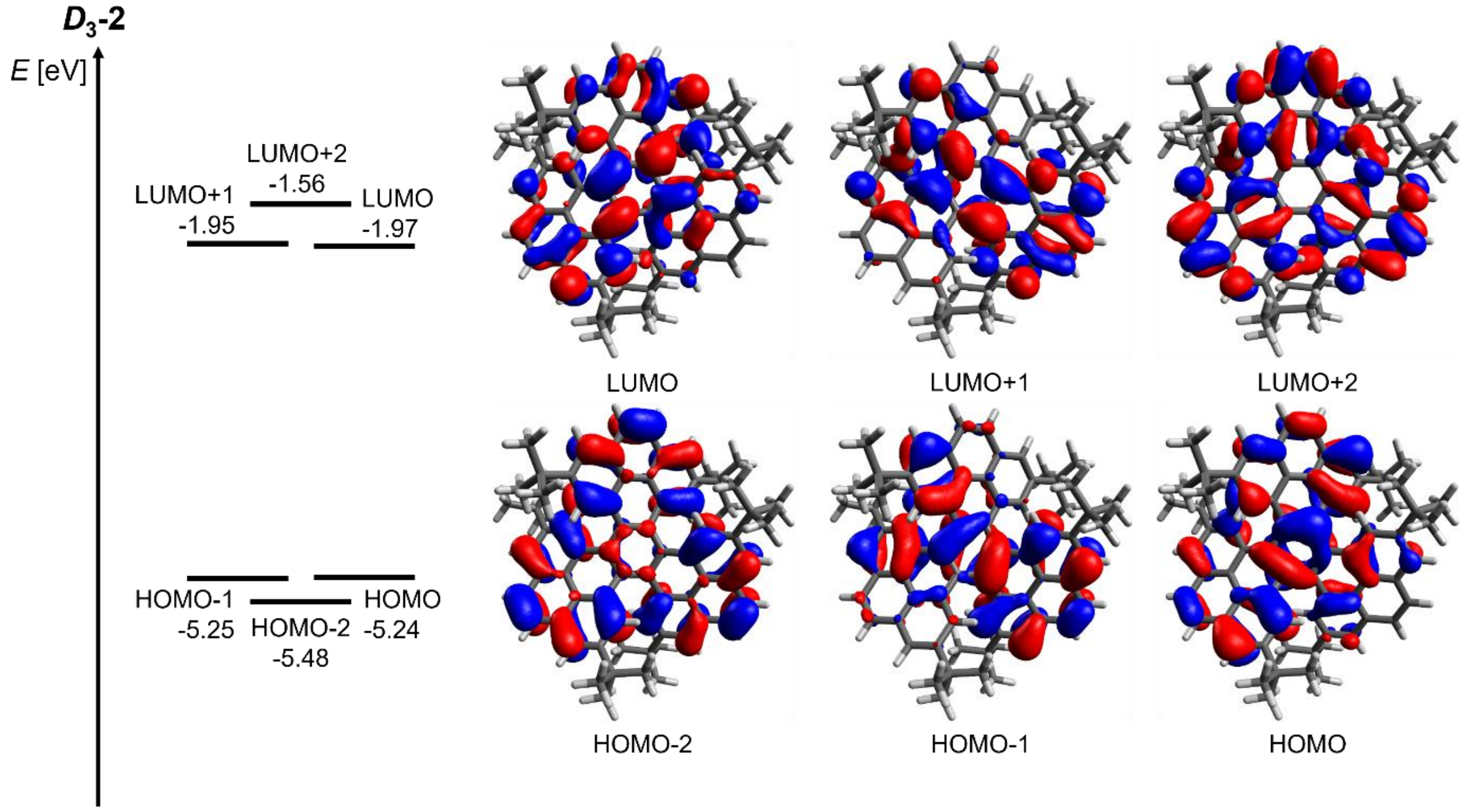

Figure S56: Calculated Frontier Molecular Orbitals of **(*P,P,P*)-*D*$_3$-2** and their energy levels in eV. Plotted with an isosurface value of 0.02 calculated at the B3LYP/6-311G(d,p) level of theory.

9.4.2 (*P*,*P*,*M*)-*C*$_2$-3

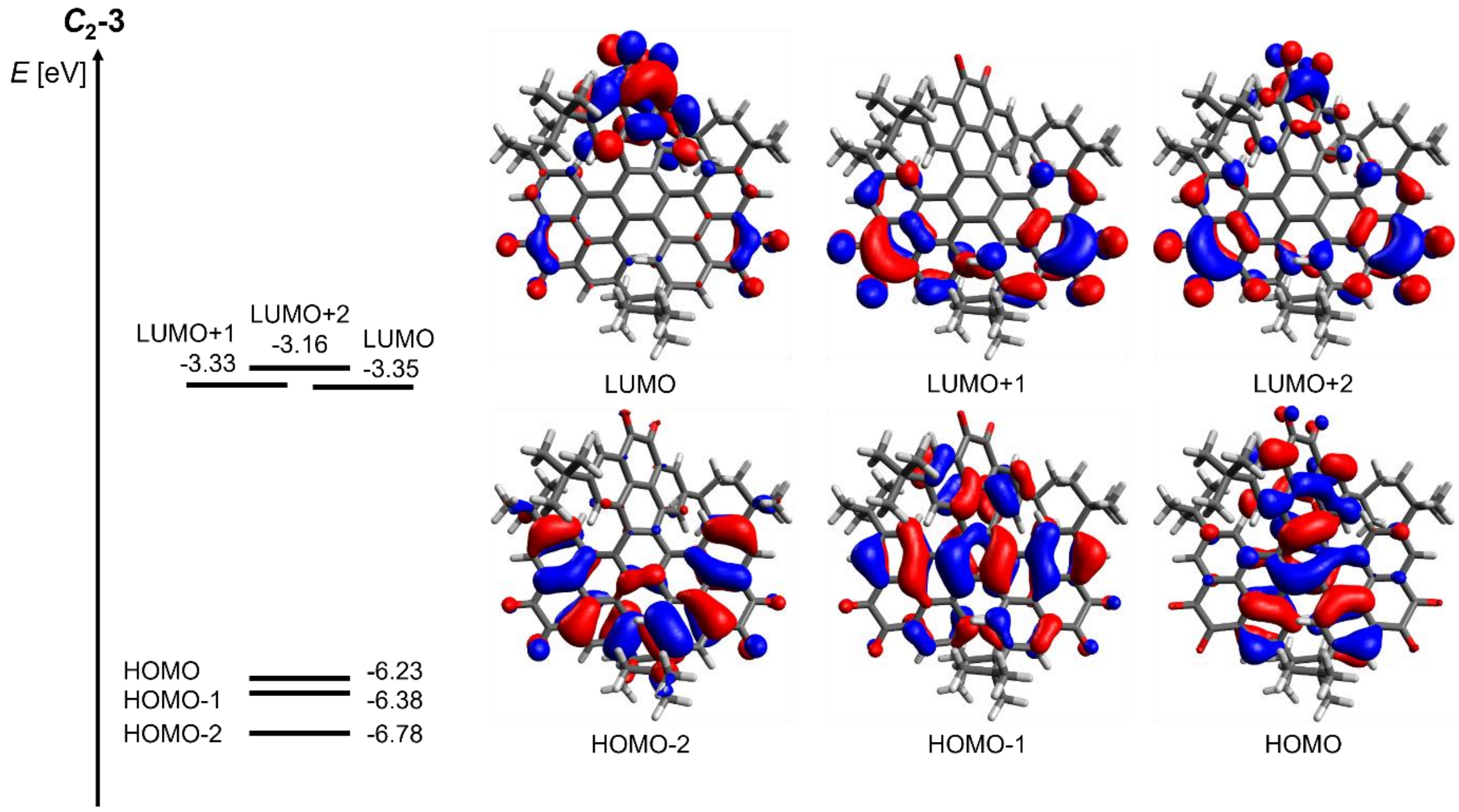

Figure S57: Calculated Frontier Molecular Orbitals of **(*P*,*P*,*P*)-*C*$_2$-3** and their energy levels in eV. Plotted with an isosurface value of 0.02 calculated at the B3LYP/6-311G(d,p) level of theory.

9.4.3 (*P*,*P*,*P*)-*D*$_3$-3

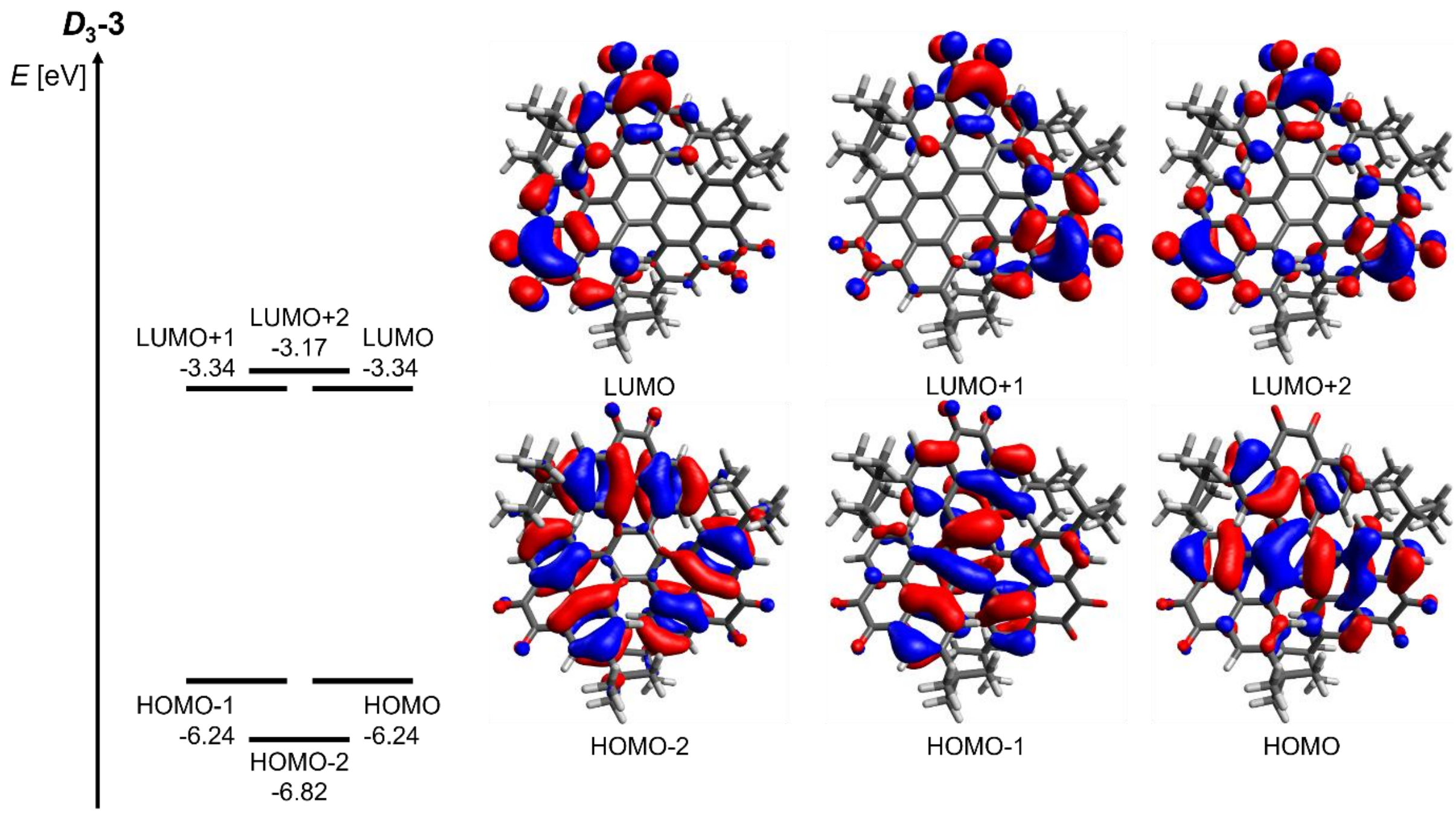

Figure S58: Calculated Frontier Molecular Orbitals of **(*P*,*P*,*P*)-*D*$_3$-3** and their energy levels in eV. Plotted with an isosurface value of 0.02 calculated at the B3LYP/6-311G(d,p) level of theory.

9.4.4 (*P*,*P*,*M*)-C_2-4

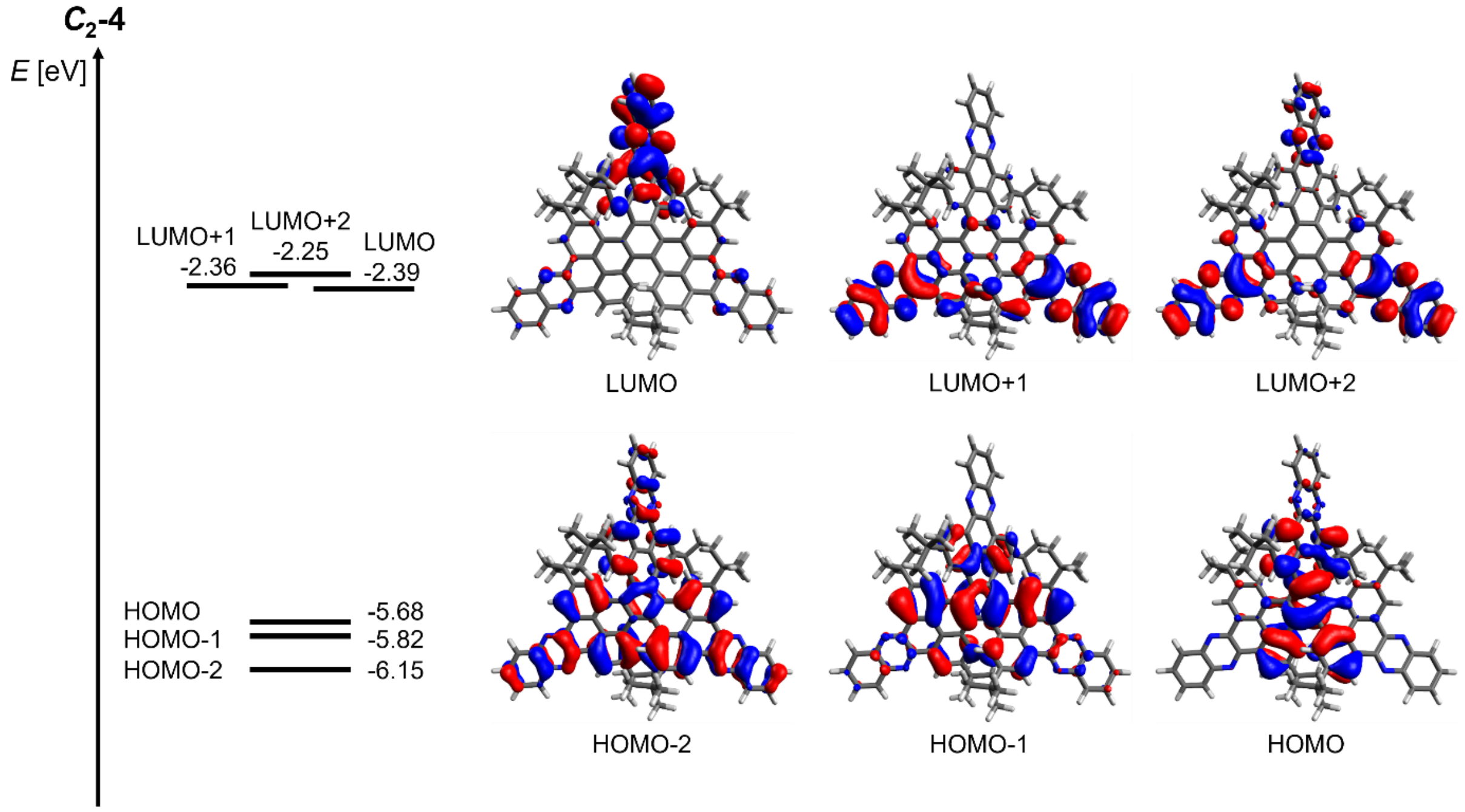

Figure S59: Calculated Frontier Molecular Orbitals of **(*P*,*P*,*P*)-*C*₂-4** and their energy levels in eV. Plotted with an isosurface value of 0.02 calculated at the B3LYP/6-311G(d,p) level of theory.

9.4.5 (*P*,*P*,*P*)-D_3-4

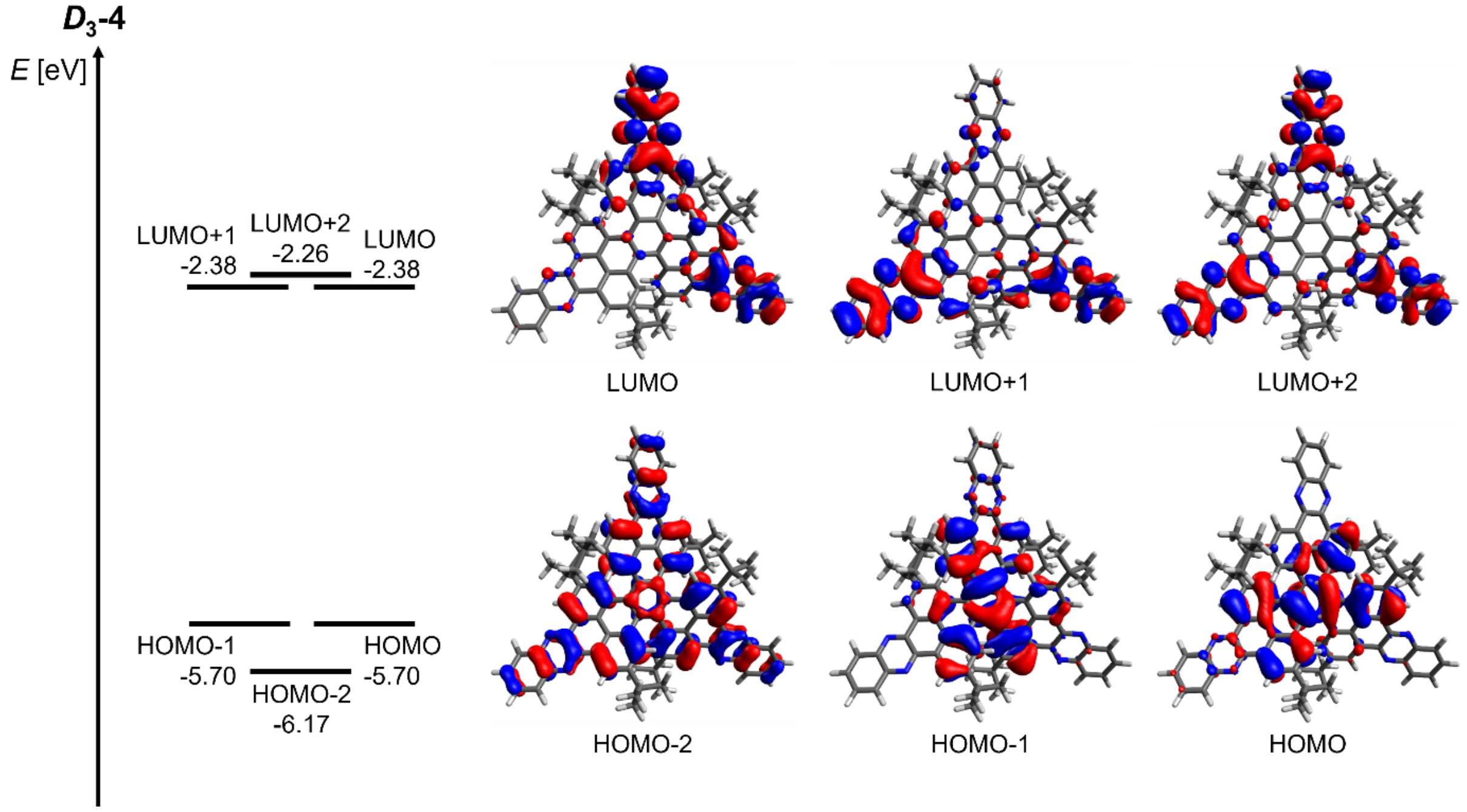

Figure S60: Calculated Frontier Molecular Orbitals of ***D*₃-4** and their energy levels in eV. Plotted with an isosurface value of 0.02 calculated at the B3LYP/6-311G(d,p) level of theory.

9.4.6 Band Gap Comparison

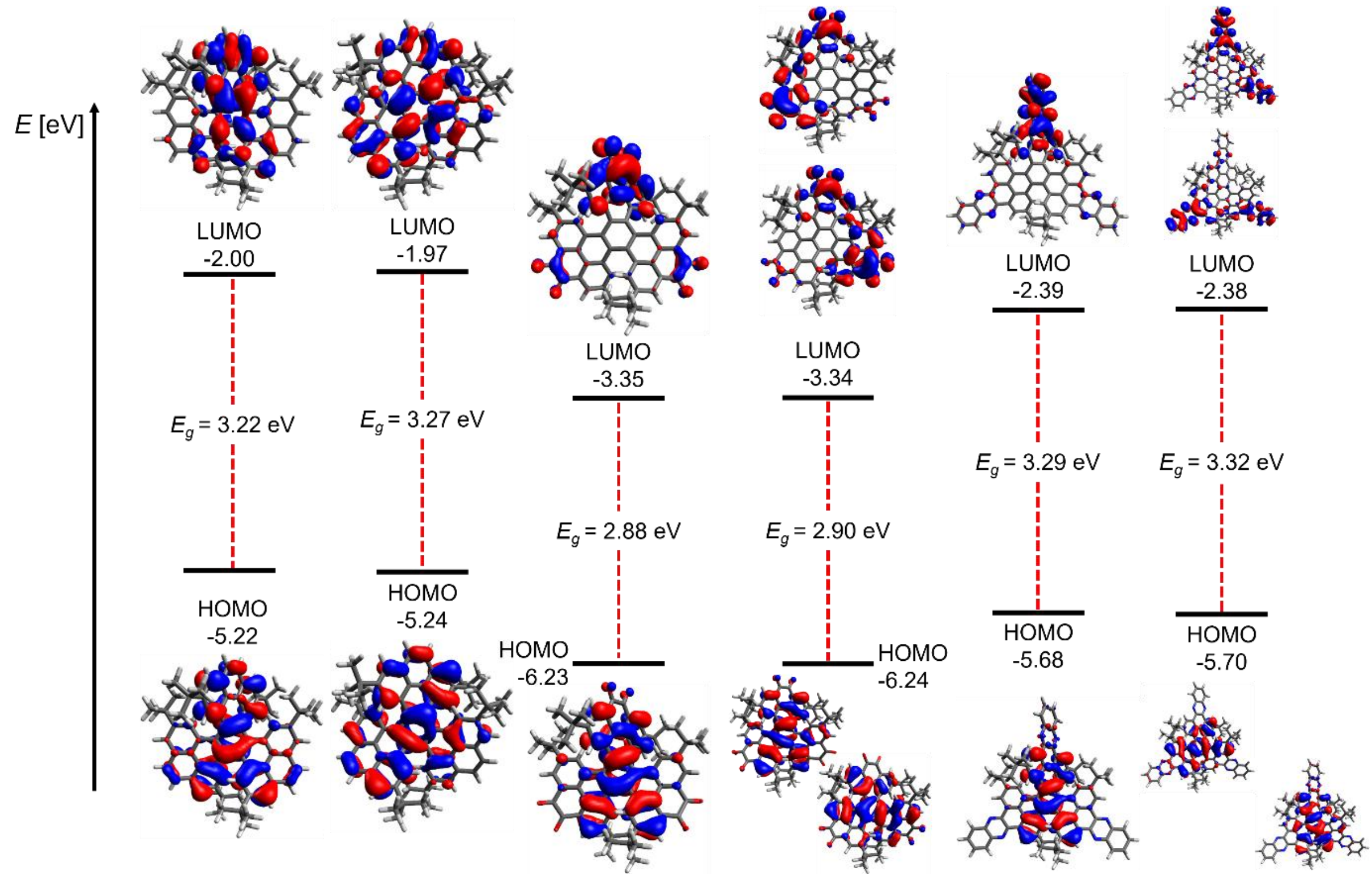

Figure S61: Comparison of the calculated HOMO and LUMO energies for **(*P*,*P*,*P*)-*D*$_3$-2**, **(*P*,*P*,*M*)-*C*$_2$-3**, **(*P*,*P*,*P*)-*D*$_3$-3**, **(*P*,*P*,*M*)-*C*$_2$-4** and **(*P*,*P*,*P*)-*D*$_3$-4** with plotted FMOs with an isosurface value of 0.02 calculated at the B3LYP/6-311G(d,p) level of theory. When two orbitals were degenerate, both orbitals were visualized.

9.5 TD-DFT

9.5.1 (*P*,*P*,*P*)-*D*$_3$-2 and (*P*,*P*,*P*)-*D*$_3$-2

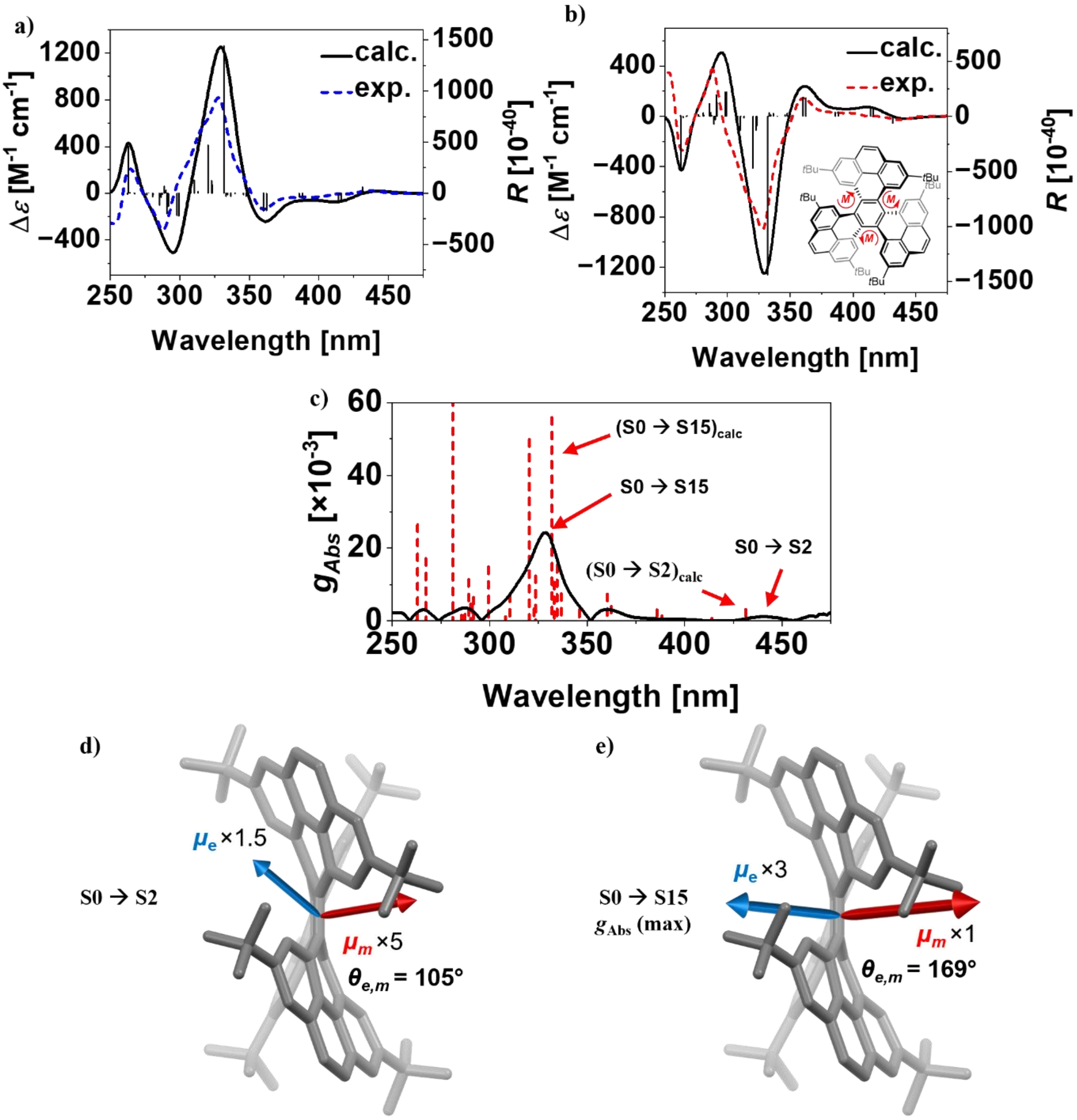

Figure S62: **a)-b)** Comparison between experimental (dashed curve) and calculated (continuous curve, σ = 0.5 eV) CD spectra of **(*P*,*P*,*P*)-*D*$_3$-2** and **(*M*,*M*,*M*)-*D*$_3$-2**. **c)** Absolute value of experimental g_{Abs} (black curve) and calculated g_{Abs}-values (red lines). Local and global maximum was labelled with the appropriate transition. **d)-e)** Electronic (μ_e, blue) and magnetic (μ_m, red) transition dipole moments and their corresponding angles θ for the S_0 to S_2 (**d)**) and S_0 to S_{15} (**e)**) transition. Calculated at the B3LYP/6-311G(d,p) level of theory. Vector lengths were scaled by the displayed factor for better visibility.

9.5.2 (*P*,*P*,*P*)-*D*$_3$-4 and (*P*,*P*,*P*)-*D*$_3$-4

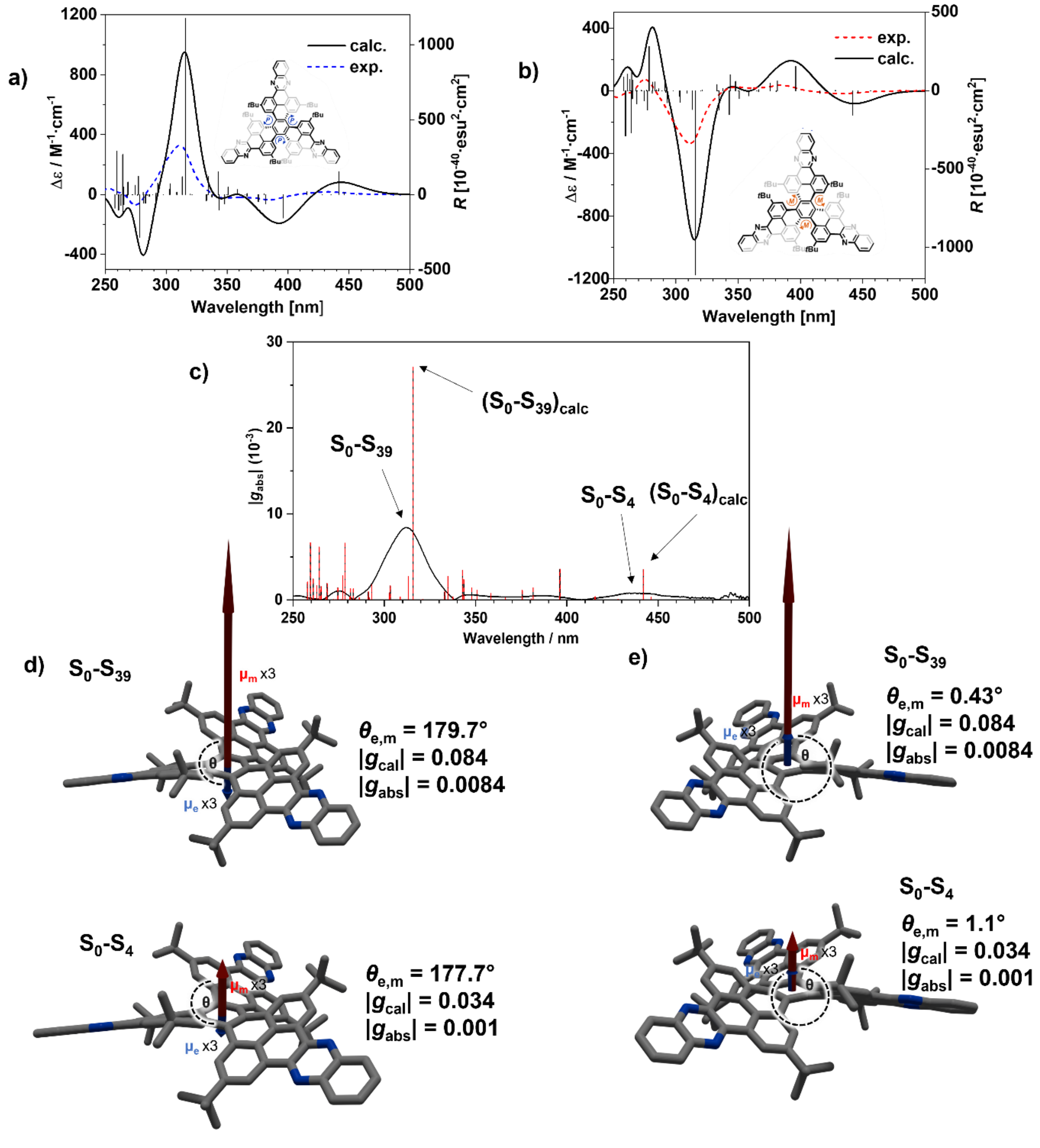

Figure S63: **a)-b)** Comparison between experimental (dashed curve) and calculated (continuous curve, σ = 0.3 eV) CD spectra of **(*P*,*P*,*P*)-*D*$_3$-4** (**a**)) and **(*M*,*M*,*M*)-*D*$_3$-4** (**b**)). **c)** Absolute value of experimental g_{Abs} (black curve) and calculated electronic transitions (Rotatory strength, red lines) with the calculated g_{abs} shown in **d)** & **e)**. Local and global maximum were labelled with the appropriate transition. **d)-e)** Electronic (μ_e, blue) and magnetic (μ_m, red) transition dipole moments and their corresponding angles θ for the S_0 to S_4 and S_0 to S_{39} transition. Calculated at the PBE0/6-311G(d,p) level of theory. Vector lengths were scaled by the displayed factor for better visibility.

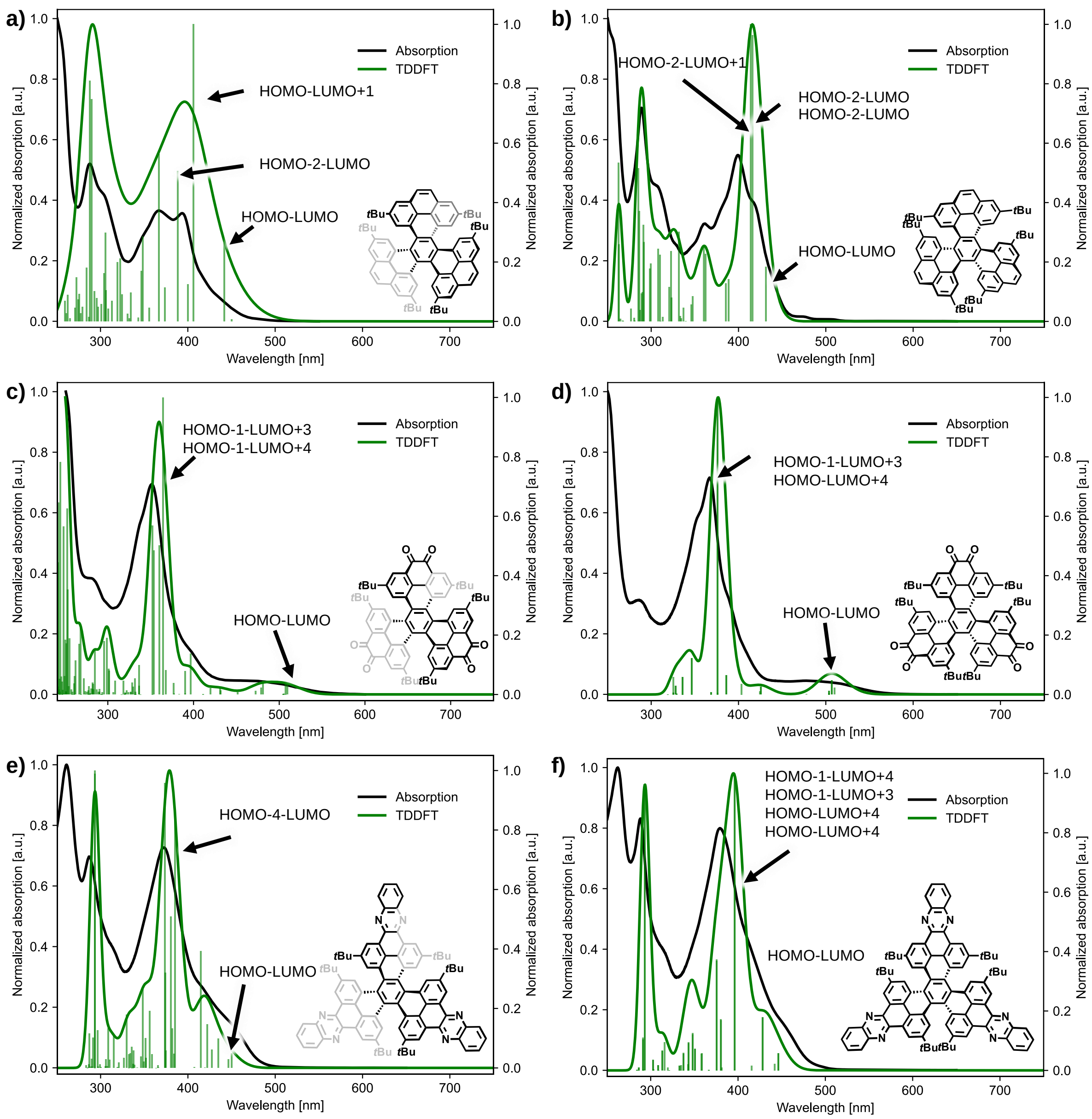

Figure S64: Comparison between experimental (black curves) and calculated (green curves) UV-vis spectra of ***(P,P,M)*-*C*$_2$-2** (a), ***(P,P,P)*-*D*$_3$-2** (b), ***(P,P,M)*-*C*$_2$-3** (c), ***(P,P,P)*-*D*$_3$-3** (d), ***(P,P,M)*-*C*$_2$-4** (e) and ***(P,P,P)*-*D*$_3$-2** (f). TD-DFT spectra have been calculated at the B3LYP/6-311G(d,p) level of theory. UV-vis spectra have been measured in dichloromethane (a-c) or chloroform (d-e).

10. References

[S1] D. Popp, S. M. Elbert, C. Barwig, J. Petry, F. Rominger, M. Mastalerz, *Angew. Chem. Int. Ed.* **2023**, *62*, e202219277.

[S2] G. Sheldrick, *Bruker Analytical X-ray Division* **2012**.

[S3] G. Sheldrick, *Acta Cryst.* **2015**, *A71*, 3–8.

[S4] G. Sheldrick, *Acta Cryst.* **2015**, *C71*, 3–8.

[S5] T. H. El-Assaad, K. N. Parida, M. F. Cesario, D. V. McGrath, *Green Chemistry* **2020**, *22*, 5966–5971.

[S6] a) M. J. Frisch, G. W. Trucks, H. B. Schlegel, G. E. Scuseria, M. A. Robb, J. R. Cheeseman, G. Scalmani, V. Barone, B. Mennucci, G. A. Petersson, H. Nakatsuji, M. Caricato, X. Li, H. P. Hratchian, A. F. Izmaylov, J. Bloino, G. Zheng, J. L. Sonnenberg, M. Hada, M. Ehara, K. Toyota, R. Fukuda, J. Hasegawa, M. Ishida, T. Nakajima, Y. Honda, O. Kitao, H. Nakai, T. Vreven, J. A. Montgomery, Jr., J. E. Peralta, F. Ogliaro, M. Bearpark, J. J. Heyd, E. Brothers, K. N. Kudin, V. N. Staroverov, R. Kobayashi, J. Normand, K. Raghavachari, A. Rendell, J. C. Burant, S. S. Iyengar, J. Tomasi, M. Cossi, N. Rega, M. J. Millam, M. Klene, J. E. Knox, J. B. Cross, V. Bakken, C. Adamo, J. Jaramillo, R. Gomperts, R. E. Stratmann, O. Yazyev, A. J. Austin, R. Cammi, C. Pomelli, J. W. Ochterski, R. L. Martin, K. Morokuma, V. G. Zakrzewski, G. A. Voth, P. Salvador, J. J. Dannenberg, S. Dapprich, A. D. Daniels, Ö. Farkas, J. B. Foresman, J. V. Ortiz, J. Cioslowski, D. J. Fox, Wallingford, CT, **2016**; b) P. Hohenberg, W. Kohn, *Phys. Rev* **1964**, *136*, B864; c) W. Kohn, L. J. Sham, *Phys. Rev.* **1965**, *140*, A1133–A1138; d) R. G. Parr, W. Yang, *Density-functional theory of atoms and molecules*, 1. Ed ed., Oxford University Press, New York, **1994**; e) W. Koch, *A Chemist's Guide to Density Functional Theory*, 2 ed. ed., Wiley-VCH, Weinheim, **2001**.

[S7] J. J. P. Stewart, *J. Mol. Model.* **2007**, *13*, 1173–1213.

[S8] a) A. D. Becke, *J. Chem. Phys.* **1993**, *98*, 5648–5652; b) P. J. Stephens, F. J. Devlin, C. F. Chabalowski, M. J. Frisch, *J. Phys. Chem.* **1994**, *98*, 11623–11627; c) C. Lee, W. Yang, R. G. Parr, *Phys. Rev. B* **1988**, *37*, 785–789; d) S. H. Vosko, L. Wilk, M. Nusair, *Can. J. Phys.* **1980**, *58*, 1200–1211.

[S9] R. Krishnan, J. S. Binkley, R. Seeger, J. A. Pople, *J. Chem. Phys.* **1980**, *72*, 650–654.

[S10] a) R. Ditchfield, W. J. Hehre, J. A. Pople, *J. Chem. Phys.* **1971**, *54*, 724–728; b) P. C. Hariharan, J. A. Pople, *Theor. Chim. Acta* **1973**, *28*, 213–222; c) W. J. Hehre, R. Ditchfield, J. A. Pople, *J. Chem. Phys.* **1972**, *56*, 2257–2261.

[S11] S. Grimme, S. Ehrlich, L. Goerigk, *J. Comput. Chem.* **2011**, *32*, 1456–1465.

[S12] A. V. Marenich, C. J. Cramer, D. G. Truhlar, *J. Phys. Chem. B* **2009**, *113*, 6378–6396.

[S13] a) G. Luchini, J. V. Alegre‐Requena, I. Funes‐Ardoiz, R. S. Paton, *F1000Research* **2020**, *9*, 291; bS. Grimme,*Chem. Eur. J.* **2012**, *18*, 9955‐9964.

[S14] C. C. J. Roothaan, *Rev. Mod. Phys.* **1951**, *23*, 69–89.

[S15] T. Clark, J. Chandrasekhar, G. W. Spitznagel, P. V. R. Schleyer, **1983**, *4*, 294–301.

[S16] a) J. R. Cheeseman, G. W. Trucks, T. A. Keith, M. J. Frisch, *J. Chem. Phys.* **1996**, *104*, 5497–5509; b) K. Wolinski, J. F. Hinton, P. Pulay, *J. Am. Chem. Soc.* **1990**, *112*, 8251–8260; c) R. McWeeny, *Phys. Rev.* **1962**, *126*, 1028–1034; d) R. Ditchfield, *Mol. Phys.* **1974**, *27*, 789–807; e) F. London, *J. Phys. Radium.* **1937**, *8*, 397–409.

[S17] a) T. A. Keith, R. F. W. Bader, *Chem. Phys. Lett.* **1993**, *210*, 223–231; b) T. A. Keith, R. F. W. Bader, *Chem. Phys. Lett.* **1992**, *194*, 1–8.

[S18] a) T. Lu, F. Chen, *J. Comput. Chem.* **2012**, *33*, 580–592; b) T. Lu, *J. Chem. Phys.* **2024**, *161*, 082503; c) T. Lu, Q. Chen, *Theor. Chem. Acc.* **2020**, *139*, 25.

[S19] a) D. Geuenich, K. Hess, F. Köhler, R. Herges, *Chem. Rev.* **2005**, *105*, 3758–3772; b) R. Herges, D. Geuenich, *J. Phys. Chem. A* **2001**, *105*, 3214–3220.

[S20] a) R. Bauernschmitt, R. Ahlrichs, *Chem. Phys. Lett.* **1996**, *256*, 454–464; b) M. E. Casida, C. Jamorski, K. C. Casida, D. R. Salahub, *J. Chem. Phys.* **1998**, *108*, 4439–4449; c) R. E. Stratmann, G. E. Scuseria, M. J. Frisch, *J. Chem. Phys.* **1998**, *109*, 8218–8224; d) C. Van Caillie, R. D. Amos, *Chem. Phys. Lett.* **1999**, *308*, 249–255; e) C. Van Caillie, R. D. Amos, Chem. Phys. Lett. **2000**, *317*, 159–164; f) F. Furche, R. Ahlrichs, *J. Chem. Phys.* **2002**, *117*, 7433–7447; g) G. Scalmani, M. J. Frisch, B. Mennucci, J. Tomasi, R. Cammi, V. Barone, *J. Chem. Phys.* **2006**, *124*, 94107.

[S21] C. Adamo, V. Barone, *J. Chem. Phys.* **1999**, *110*, 6158–6170.
[S22] N. M. O'Boyle, A. L. Tenderholt, K. M. Langner, *J. Comput. Chem.* **2008**, *29*, 839–845.
[S23] a) B. P. Pritchard, D. Altarawy, B. Didier, T. D. Gibson, T. L. Windus, *J. Chem. Inf. Model.* **2019**, *59*, 4814–4820; b) D. Feller, *J. Comput. Chem.* **1996**, *17*, 1571–1586; c) K. L. Schuchardt, B. T. Didier, T. Elsethagen, L. Sun, V. Gurumoorthi, J. Chase, J. Li, T. L. Windus, *J. Chem. Inf. Model.* **2007**, *47*, 1045–1052.
